# Comparative proteomic study of dog and human saliva

**DOI:** 10.1371/journal.pone.0208317

**Published:** 2018-12-04

**Authors:** Phutsa Sanguansermsri, Howard F. Jenkinson, Jitkamol Thanasak, Kongthawat Chairatvit, Sittiruk Roytrakul, Suthathip Kittisenachai, Duangchewan Puengsurin, Rudee Surarit

**Affiliations:** 1 Department of Oral Biology, Faculty of Dentistry, Mahidol University, Bangkok, Thailand; 2 Department of Clinical Medicine and Public Health, Faculty of Veterinary Science, Mahidol University, Nakhon Pathom, Thailand; 3 Bristol Dental School, University of Bristol, Bristol, United Kingdom; 4 Proteomics Research Laboratory, National Center for Genetic Engineering and Biotechnology, Pathumthani, Thailand; Virginia Commonwealth University, UNITED STATES

## Abstract

Saliva contains many proteins that have an important role in biological process of the oral cavity and is closely associated with many diseases. Although the dog is a common companion animal, the composition of salivary proteome and its relationship with that of human are unclear. In this study, shotgun proteomics was used to compare the salivary proteomes of 7 Thai village dogs and 7 human subjects. Salivary proteomes revealed 2,532 differentially expressed proteins in dogs and humans, representing various functions including cellular component organization or biogenesis, cellular process, localization, biological regulation, response to stimulus, developmental process, multicellular organismal process, metabolic process, immune system process, apoptosis and biological adhesion. The oral proteomes of dogs and humans were appreciably different. Proteins related to apoptosis processes and biological adhesion were predominated in dog saliva. Drug-target network predictions by STITCH Version 5.0 showed that dog salivary proteins were found to have potential roles in tumorigenesis, anti-inflammation and antimicrobial processes. In addition, proteins related to regeneration and healing processes such as fibroblast growth factor and epidermal growth factor were also up-regulated in dogs. These findings provide new information on dog saliva composition and will be beneficial for the study of dog saliva in diseased and health conditions in the future.

## Introduction

Saliva is an important fluid that maintains homeostasis in the oral cavity. It contains many kinds of proteins and peptides including immunoglobulins, enzymes and cytokines [1]. Saliva has numerous functions such as moistening food and bolus formation, lubrication of the oral mucosa, maintaining the mineralization of teeth, tissue defense and buffering system of the oral cavity [2]. Human saliva has been well studied and in terms of human medicine has been employed in diagnostic tests for oral diseases, cancer, and systemic diseases, since saliva constituents provide information on health status [3,4]. In the veterinary field, a variety of techniques such as immunofluorometric assay, enzyme-linked immunosorbent assay and radioimmunoassay, have been utilized in developing salivary protein detection [5–8].

Dogs are a major reservoir for zoonotic infections. The resident pathogenic oral bacteria or viruses can be transmitted to humans mainly by infected saliva. Therefore, saliva becomes more important for public health considerations. However, the composition of the dog salivary proteome, which may also be associated with human pathogenic organisms, and its relationship with that of their owners remain unclear. Alteration of dog or human saliva protein composition by age, food consumption, environmental changes, and health condition may increase the risk of dog-associated zoonotic infection. Major proteins identified by a proteomics approach in dog saliva were involved in metabolism, however, major proteins in human saliva were cytoskeletal and inflammation-related [9]. Dog saliva has a more basic pH and higher buffering capacity than human saliva, and has different electrolyte composition in calcium, potassium and sodium [10]. The aim of this study was to identify proteins present in dog or human salivary samples utilizing shotgun proteomics. Better understanding of salivary functions as a result of proteome information will further studies of pathophysiological mechanisms of diseases in dogs.

## Materials and methods

### Dogs

Saliva was obtained from 7 healthy Thai village dogs (1–3 years old) at the Veterinary Teaching Hospital of Mahidol University, Thailand. The study was approved by the Faculty of Veterinary Science-Animal Care and Use Committee (FVS-ACUC) (Protocol No. MUVS-2015-19). Written informed consent forms were obtained from all dog owners. Health status of the dogs was determined by veterinarians. The dogs were included in this study according to the following criteria: none of the dogs had received antibiotics within the 3-month period before sample collection, no signs of oral diseases (clinically healthy, probing depth < 3 mm and no gingival inflammation) or systemic diseases. Dog saliva was allowed to drip from the mouth into a collecting vessel, or was collected using a syringe at the buccal area from healthy dogs under anesthesia.

### Human subjects

Seven subjects were recruited from the Dental Hospital, Faculty of Dentistry, Mahidol University, Thailand. Whole, unstimulated saliva was collected following informed consent from healthy volunteers by Navazesh’s method [11] between 07:00 and 10:00. All subjects showed no sign of periodontitis (probing depth < 3 mm and no attachment loss). The study protocol was approved by the Ethics Committee of the Faculty of Dentistry/Faculty of Pharmacy, Mahidol University Institutional Review Board (COA.No.MU-DT/PY-IRB 2011/012.3103). Written informed consent forms were obtained from all subjects.

### Saliva preparation

**Fig 1:**
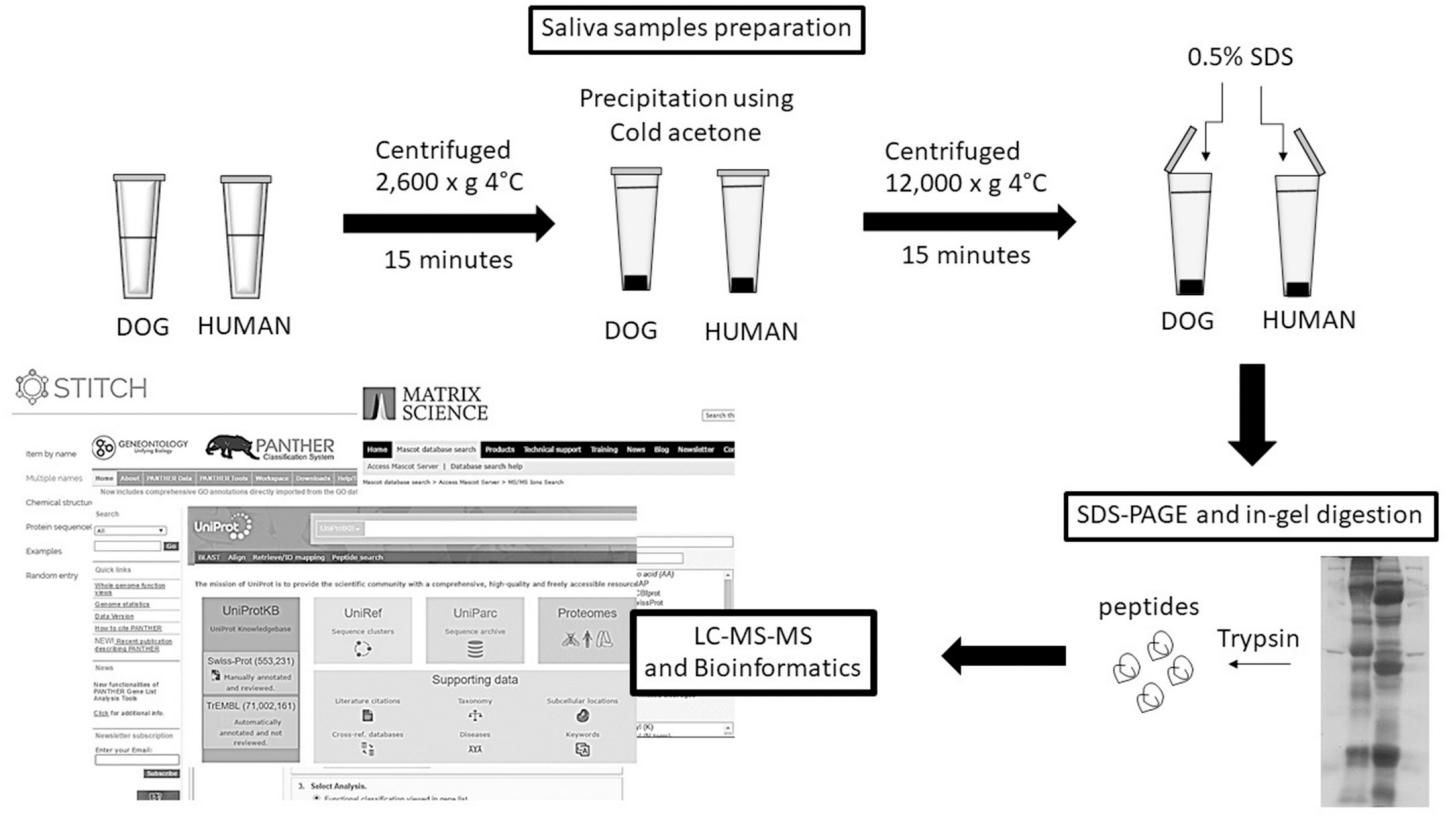
Workflow of shotgun proteomics analysis.

Protease inhibitor cocktail (Roche, Mannheim, Germany) was added to the saliva samples immediately after collection and they were stored at -80 °C until use. Saliva was centrifuged at 2,600 × g at 4 °C for 15 min and the supernatant was collected. Protein concentrations of samples were estimated using Bradford Protein assay [12]. Portions of saliva containing 10 μg protein from each dog were pooled and proteins were precipitated with 3 volumes of ice-cold acetone at -20 °C for 16 h. The precipitant was collected by centrifugation at 12,000 × g at 4 °C for 15 min. The supernatant was discarded and protein pellet was allowed to air dry. Human salivary proteins were prepared using same procedure. The workflow of shotgun proteomics analysis is shown in Fig 1.

### Sodium Dodecyl Sulfate Polyacrylamide Gel Electrophoresis (SDS-PAGE) for shotgun proteomics

Protein pellets from dog or human samples were suspended in 0.5% SDS and heated at 60 °C for 30 min. Samples containing 50 μg protein were mixed with SDS-sample buffer containing 0.5 M dithiothreitol (DTT), 10% w/v SDS, 0.4 M Tris-HCl pH 6.8 and 50% v/v glycerol and proteins were separated by SDS-PAGE (12.5% acrylamide gel) at 90 volts. Proteins were stained with Coomassie Brilliant Blue R-250.

### Protein identification by LC-MS/MS

Protein bands on SDS-PAGE gels were excised manually according to molecular size using a scalpel and subjected to in-gel digestion followed by High-performance liquid chromatography-Tandem mass spectrometry (LC/MS/MS) using an in-house method as described previously [13]. Briefly, gel plugs were dehydrated with 100% acetonitrile (ACN), reduced with 10 mM DTT and alkylated in 100 mM IAA solution. After that, proteins in gel plugs were digested by trypsin at 37 °C overnight. The peptides were extracted by 50% ACN in 0.1% trifluoroacetic acid followed by drying at 40 °C overnight. The peptides were analyzed by nanoLC-MS/MS (nanoACQUITY UPLC/ SYNAPT HDMS, Waters, Milford, USA) coupled to mass spectrometry (Electrospray ionization/Quadrupole-Time of flight (ESIQ-TOF), Waters, Milford, USA). Finally, the raw MS/MS data were subjected to DecyderMS [14,15] for quantitation, and the analyzed MS/MS data were sent to identify by Mascot MS/MS Ions Search [16] using NCBI protein database. Gene ontology annotations included biological process, cellular component and molecular function were performed using Panther database [17]. Protein-chemical interactions were analyzed according to STITCH 5.0 database [18].

### Immunoblotting analysis

Immunoblotting analysis were performed using 10 μg protein from each group. Samples were separated by 7.5% or 10% SDS-PAGE and transferred to nitrocellulose membranes and subsequently probed with antibodies. Antibodies to Amylase, Baculoviral IAP repeat-containing protein 2 (BIRC2) and Carbonic anhydrase 6 were from Abcam, Cambridge, MA, USA., MyBioSource, Inc., San Diego, CA, USA. and Santa Cruz Biotechnology, Inc., USA. Immunoreactive protein bands were visualized using an ECL detection reagent (PerkinElmer, Inc., Waltham, MA, USA).

### Statistical analysis

Data were analyzed using an Analysis of Variance (ANOVA) to determine differences between groups at a significance level of 95%.

## Results

**Fig 2:**
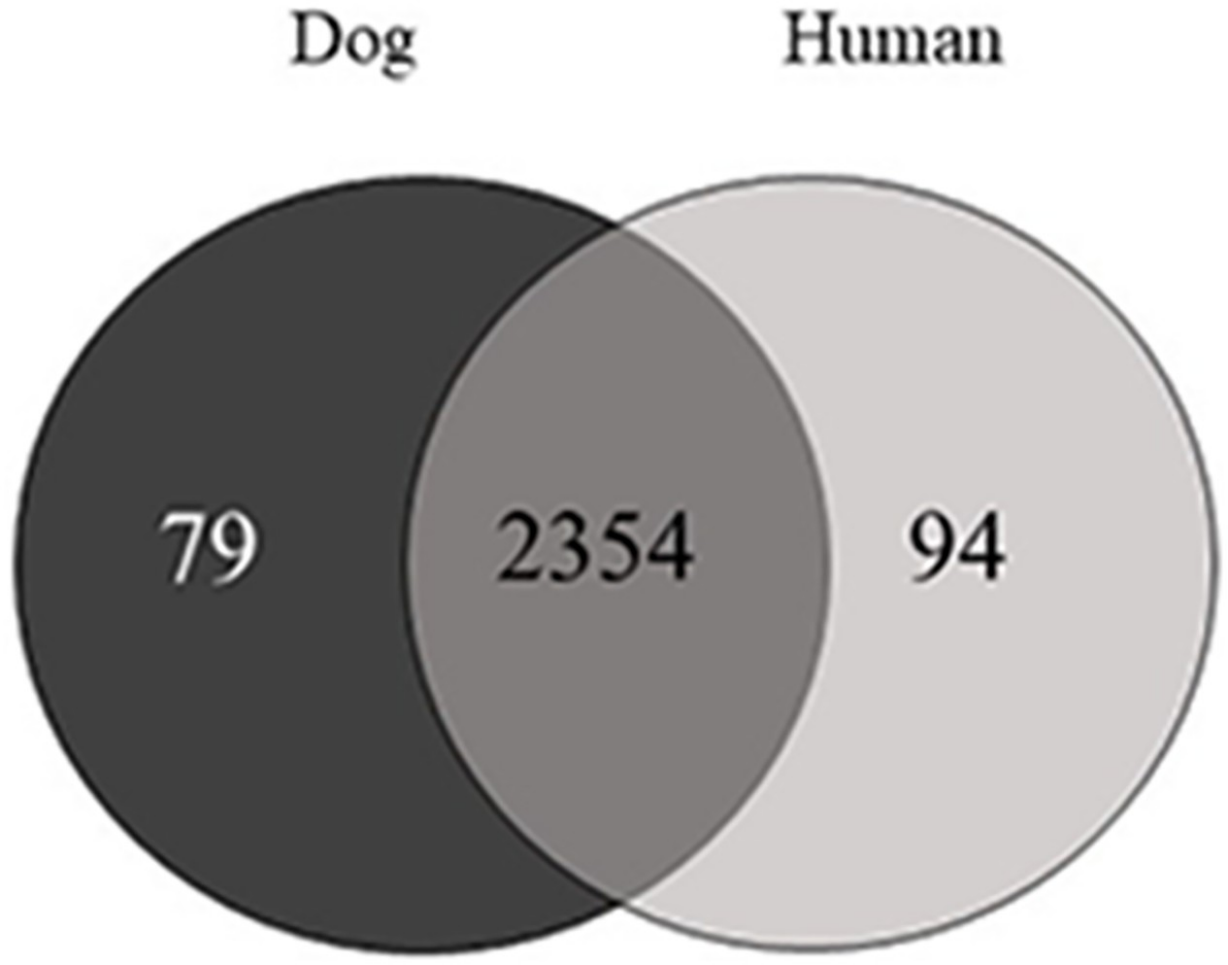
Venn diagram identifying the overlapped and unique proteins of dog and human saliva.

**Fig 3:**
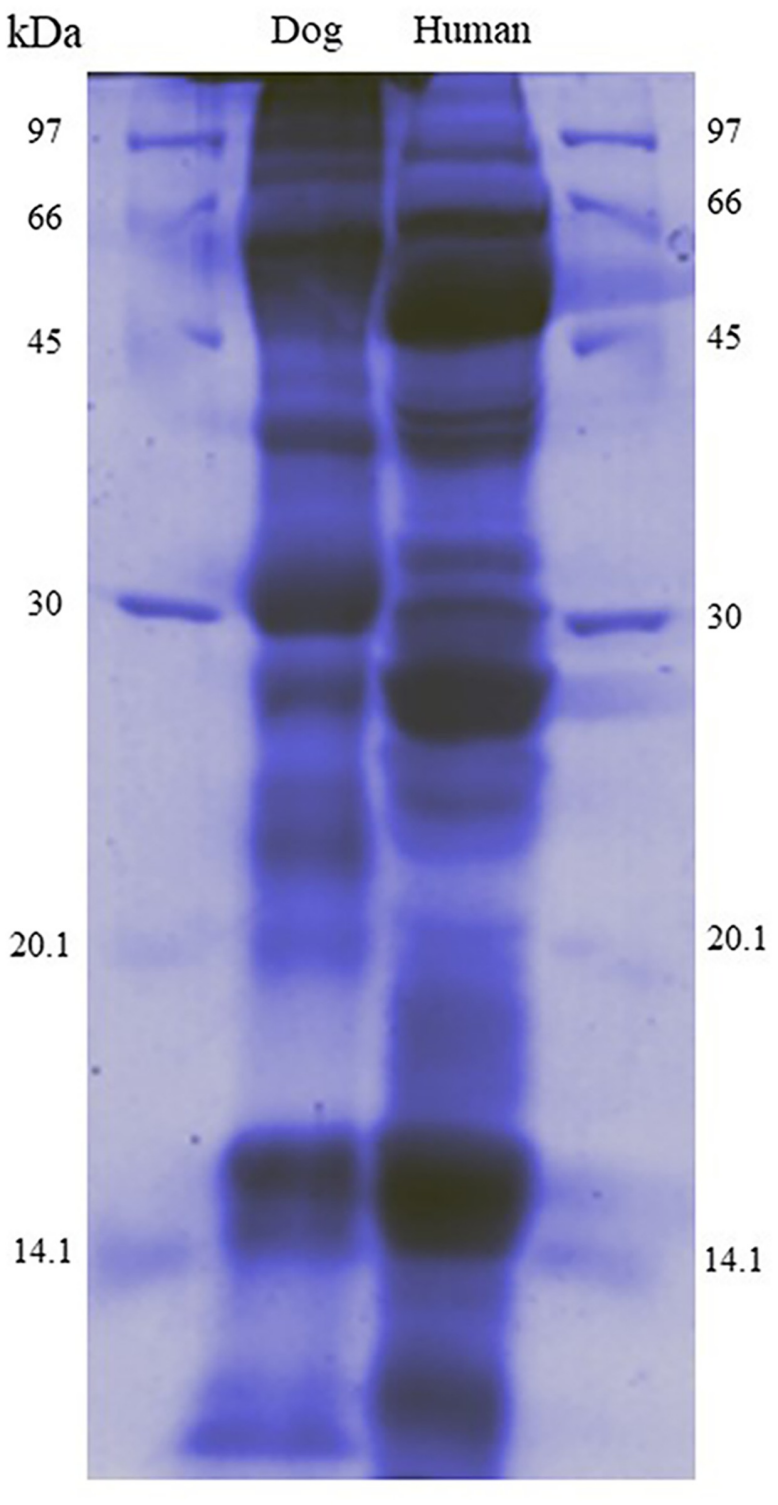
Representative SDS-PAGE of 50 μg protein from dog saliva pool and human saliva pool.

A total of 2,532 proteins (S1, S2 and S3 Tables) showed significantly different expression in dogs when compared to humans, with 79 proteins being found only in dog saliva (Fig 2). The salivary protein profiles from SDS-PAGE showed different banding patterns between dog and human saliva (Fig 3). Predominant bands at high molecular weight were observed in dog saliva.

**Fig 4:**
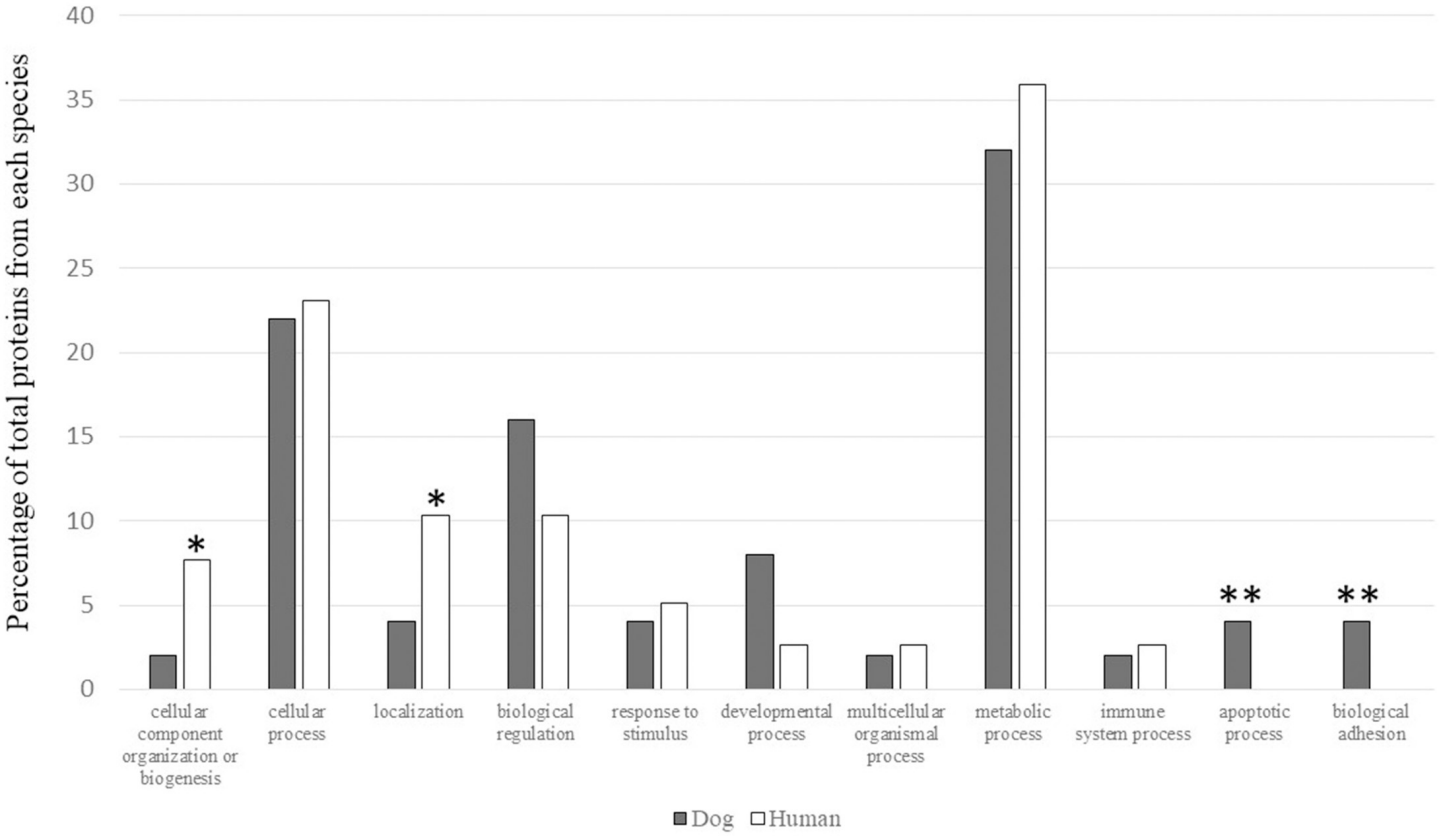
Comparison of functional characteristic of proteins found only in dogs and human. *: Dominant functions found in human. **: Dominant functions found only in dogs.

According to the functional annotation of salivary proteins using Gene Ontology Annotation Database, the identified proteins were classified as cellular component organization or biogenesis, cellular process, localization, biological regulation, response to stimulus, developmental process, multicellular organismal process, metabolic process, immune system process, apoptosis and biological adhesion as shown in Fig 4.

The most abundant proteins found only in dog saliva were proteins with metabolic process (e.g. mitochondrial 10-formyltetrahydrofolate dehydrogenase precursor) (32%) followed by proteins with cellular process (e.g. 60S ribosomal protein L13) (22%) and biological regulation (e.g. histone H2A type 2-A) (16%). Unique proteins with dominant functions found in each species were presented in Table 1.

**Table 1:** Unique proteins from dominant functions found only in dog or human.

Dogs: Apoptotic process and Biological adhesion		Human: Cellular component organization or biogenesis and Localization	
Protein name	Known functions	Protein name	Known functions
**Baculoviral IAP repeat-containing protein 2 (BIRC2)**	Apoptosis inhibitor and inflammatory signaling and immunity, cell proliferation, cell invasion and metastasis [19,20]	**5-methylcytosine rRNA methyltransferase NSUN4 isoform a (NSUN4)**	Cell proliferation and differentiation, protein biosynthesis and cancer [29]
**Dexamethasone-induced Ras-related protein 1 isoform X1 (RASD1)**	Intracellular signaling process related to cell morphology, cell growth and cell-extracellular matrix interaction in variety of cell types including heart, liver, brain and immune cells [21–24]	**Actin-related protein 8 isoform X1 (ACTR8)**	Processes of vesicle motility, mitosis, actin filament dynamics, and modulation of chromatin structure [30]
**E3 ubiquitin-protein ligase XIAP isoform X1 (XIAP)**	Apoptosis inhibition process inflammatory signaling pathway, cell proliferation and metastasis and cell invasion [25–27]	**DNA repair protein RAD50 (RAD50)**	DNA repairing process [31]
**Notch-related protein or Neurogenic locus notch homolog protein 4 (NOTCH4)**	Acts as a receptor involved in developmental process such as differentiation, proliferation and program cell death [28]	**Dynein heavy chain 6-axonemal isoform X1 (DNAH6)**	Cellular movement [32]
		**H chain H-crystal structure of the Dbl and pleckstrin homology domains of Dbs in complex with RhoA (RHOA***)*	Actin cytoskeleton organization, cell cycle progression, cell transformation and invasion [33,34]
		**Rabenosyn-5 isoform X1 (ZFYVE20)**	Vesicle transportation [35]

When compared with dog saliva, proteins involved in cellular component organization or biogenesis and localization were more prevalent in human saliva as shown in Fig 4. These proteins included 5-methylcytosine rRNA methyltransferase NSUN4 isoform a (NSUN4), actin-related protein 8 isoform X1 (ACTR8), DNA repair protein RAD50 (RAD50), dynein heavy chain 6-axonemal isoform X1 (DNAH6), H chain H-crystal structure of the Dbl and pleckstrin homology domains of Dbs in complex with RhoA (RHOA) and rabenosyn-5 isoform X1 (ZFYVE20) (Table 1).

Interestingly, proteins involved in apoptosis processes and in biological adhesion function were detected only in dog saliva (Fig 4). Apoptosis-related proteins included E3 ubiquitin-protein ligase XIAP isoform X1 (XIAP) and Baculoviral IAP repeat-containing protein 2 (BIRC2), while biological adhesion-related proteins included notch-related protein (Neurogenic locus notch homolog protein 4 or NOTCH4) and dexamethasone-induced Ras-related protein 1 isoform X1 (RASD1) (Table 1). Other proteins that were only present in dog saliva are sirtuin and suppressin. Sirtuin is an important protein related to cellular homeostasis such as carbohydrate, protein and lipid metabolism, aging and may play a role in type II diabetes pathogenesis [36,37]. Suppressin is the other protein that was only present in dog saliva. This protein plays a role in immunological regulation by controlling the functions of lymphocytes [38]. Moreover, proteins related to angiogenesis, cell regeneration and wound healing such as fibroblast growth factor 12 isoform X3, fibroblast growth factor receptor substrate 3 isoform X1, epidermal growth factor receptor kinase substrate 8-like protein 1 isoform X4, and growth hormone-inducible transmembrane protein, were found to up-regulate in dog saliva (Table 2).

**Table 2:** Expression level of proteins in dog compared to human.

Protein name	Accession number	Peptide sequence	Score	Fold change in dog
Amylase	gi|578798954	TGSGDIENYNDATQVR	91.58	-4.6
Carbonic anhydrase 6	gi|28812184	SYDIAQHEPDGLAVLAALVK	62.64	4.76
Cystatin S	gi|4503109	IIPGGIYDADLNDEWVQR	86.73	-7.6
Cystatin SA	gi|4503105	IIEGGIYDADLNDER	32.31	-3.88
Cystatin SN	gi|19882251	QLCSFEIYEVPWENRR	19.36	-9.54
Epidermal growth factor receptor kinase substrate 8-like protein 1 isoform X4	gi|545487378	QRDVLEVLDDRR	14.84	3.78
Fibroblast growth factor 12 isoform X3	gi|578807164	SSGTPTMNGGK	15.71	1.78
Fibroblast growth factor receptor substrate 3 isoform X1	gi|578811367	RHGGGTR	15.56	0.04
Growth hormone-inducible transmembrane protein	gi|118200356	TRIGIR	16.39	0.86
Histatin	gi|4504529	EFPFYGDYGSNYLYDN	18.43	-16.74
Ig H-chain, partial	gi|185315	GITGTT	17.42	11.27
IgGFc-binding protein-like	gi|545488815	VAGLCGNFNRDPADDVDGPDPR	10.28	8.02
Immunoglobulin alpha-2 heavy chain, partial	gi|184761	QEPSQGTTTFAVTSILR	93.14	-9.1
Immunoglobulin epsilon variable region, partial	gi|288189027	DDSKNMLYLHMNR	20.57	-7.44
Immunoglobulin heavy chain constant region CH2, partial	gi|124390009	QISVSWFR	42.53	6.2
Immunoglobulin heavy chain variable region, partial	gi|112694973	VQCEVQVLASGGGLAQPGGSLR	19.26	6.18
Immunoglobulin heavy chain VDJC region, partial	gi|553426	STXGGTAALGCLVK	23.24	-6.28
Immunoglobulin heavy chain, partial	gi|219566253	SPSLESRLTINK	9.84	-10.76
Immunoglobulin heavy variable 3–49*03, partial	gi|371570901	GLIQPGRSLR	4.67	-8.6
Immunoglobulin kappa light chain, partial	gi|3169770	DSTYSLSSTLTXSK	55.61	9.32
Immunoglobulin L/VH3-9/N13/DH2-15/N5/JH4 heavy chain variable region, partial	gi|209165484	SYVVTAEYYFD	14.22	-11.08
Immunoglobulin variable region, partial	gi|323432812	TVAXPSVFIFPPSDEQLK	19.22	-9.54
Immunoglobulin VH_3c kappa chain, partial	gi|339272251	SGTASVLCLLNNFYPR	99.69	-6.56
Interferon alpha-1/2-like	gi|345806671	RDPPGSPR	27.01	5.88
Interferon alpha-8 precursor	gi|42476083	ALILLAQMRR	21.42	11.42
Interferon regulatory factor 6	gi|343131263	HATRHSPQQEEENTIFK	9.88	6.88
Interferon-induced protein with tetratricopeptide repeats 3	gi|545494542	AKSTEEGK	11.18	3.44
Interleukin 12 receptor, beta 1, isoform CRA_c, partial	gi|119605061	SGDGVAEPR	34.84	-6.32
Interleukin 17 receptor, isoform CRA_c	gi|119578144	GAEGVCGIQNGSLRWAEVKR	24.61	-12.26
Interleukin-18 receptor accessory protein isoform X1	gi|530368833	TETTGR	7.63	-6.16
Lactoperoxidase	gi|345805633	FWWENPGVFTEK	44.89	-2.62
Lactoperoxidase isoform 3 preproprotein	gi|231569458	NGFPLPLAR	43.32	3.32
Mucin-5AC	gi|545531528	ALSGVVEGTAAAFANTWK	65.56	1.1
Mucin-7-like	gi|545560548	NNVQQYTVDR	47.9	2.66
Mucin-16	gi|545535651	VALGLAGISMDPK	10.52	-3.54
Mucin-19-like	gi|545547107	DCLCTIFGNYVK	19.42	5.9
Olfactory marker protein	gi|545536254	MAEDGPKQPQLSMPLVLDPDLTK	10.05	9.34
Olfactory receptor 10T2	gi|52218846	VLGMPVATK	9.43	-16.52
Olfactory receptor 5AP2	gi|50979290	DVKKALK	6.95	-6.58
Olfactory receptor 5B12	gi|52317108	MRSPEGR	6.31	8.08
Otolin	gi|359323777	TGLKGEAGDMGIPGPPGVVGPQGPK	3.27	4.72
Prolactin-induced protein, partial	gi|116642859	TYLISSIPLXGAFNYK	51.56	-6.44
Sirtuin	gi|119581643	EAGAGR	7.81	22.6
Suppressin	gi|3293442	IHADAKR	7.51	11.18

**Fig 5:**
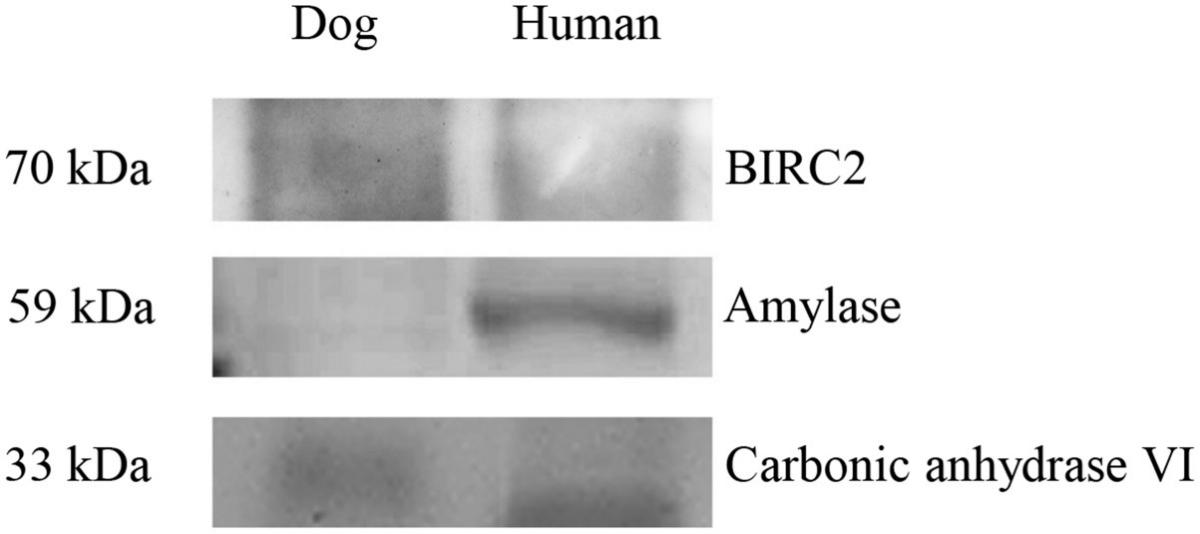
Immunoblotting analysis of salivary proteins (BIRC2, amylase and carbonic anhydrase 6) was performed in dog and human samples.

Besides these, proteins with antimicrobial properties were also found in dog saliva such as cystatin S, SA and SN, histatin, interferon alpha-1/2-like, lactoperoxidase and prolactin-inducible protein. Interestingly, levels of these proteins in dog saliva were much lower than in human saliva (Table 2). However, immunoglobulins, interferon and interleukin detected may play more of a role in the oral defense mechanism. Increased levels of carbonic anhydrase 6 with high buffering capacity were demonstrated in dog saliva. Various mucins were identified in both species, such as mucin-5AC, mucin-7-like, mucin-16 and mucin-19-like, but only mucin-16 was present in dogs at lower amounts than in human saliva. In addition, a much lower level of amylase was present in dog saliva. Validation of proteins expression were performed by immunoblotting analysis (Fig 5). The expression of BIRC2 and carbonic anhydrase 6 were detected in dog saliva whereas amylase protein was detected in human saliva. These results were consistent with shotgun proteomics.

## Discussion

From protein profiles on SDS-PAGE analysis, dominant bands in dogs were found at high molecular weight while dominant bands in human saliva were found at both high and low molecular weight. The difference between the saliva proteins of dog and human may indicate the possibility of zoonotic pathogen reservoir and/or transfer. Shotgun proteome analysis showed that levels of proteins involved in metabolic processes were highest in dog saliva including apolipoprotein, histone, PHD finger protein, RNA-binding motif protein, transcription elongation factor A protein and Zinc finger protein. The level of apolipoprotein A-I in saliva was found to correlate with the levels of high-density lipoprotein (HDL) in plasma [39]. This protein has been used as a biomarker in coronary heart disease and Parkinson disease in human [40]. The presence of higher amount of apolipoprotein in dog saliva compared to human may be related to low incident of atherosclerosis in dog [41]. The different amount of amylase in each species may explain the difference between the food consumption behaviour [42]. From our results, dog saliva had lower amounts of alpha-amylase than human saliva which is possibly due to the natural selection associated with digestion capability and food appreciation. The study of diet and evolution of amylase gene revealed that high salivary amylase expression was observed in human populations with high-starch diets which may from natural selection and evolution [42]. Since the dog is carnivorous animal, amylase activity in this species is not as dominant as omnivorous species as humans. However, dogs in Thailand are often fed with rice and starch so amylase may present in their saliva.

Our study showed no lactoferrin in dog saliva which is consistent with a report by Sousa‐Pereira et al. [9]. Cystatin A, B, C, D, E/M and S were previously identified in dog saliva [9,43], but only cystatin S, SA and SN were detected in our study. The different results between the present study and other studies may due to the different methods for sample collection or preparation, and to different breeds of dogs since there are reports showing inter-individual variations and differences between breeds [9,43,44]. Numerous studies of cystatins in dogs have been reported, however, only serum cystatin C is evaluated as a biomarker in dog’s glomerular disease [45]. In humans, cystatin S, SA and SN in both serum and saliva were reported for their potential uses as biomarkers for cancer diseases and autoimmune disorders, and antibacterial properties against periodontal pathogens [46–48]. A future study of these proteins in dog saliva would be of interest in biomarkers and antimicrobial research. Interestingly, fibroblast growth factor and growth hormone were present in higher amounts in dog saliva. These might indicate that these proteins are protective factors in defense and repair after fighting. Other wild animals may also have similar salivary composition, however, salivary protein compositions in most wild animals have not yet been reported. Keratins were down-regulated in dog saliva, which may be from the difference in turnover rate of the oral epithelium between dogs and human [49–51].

**Fig 6:**
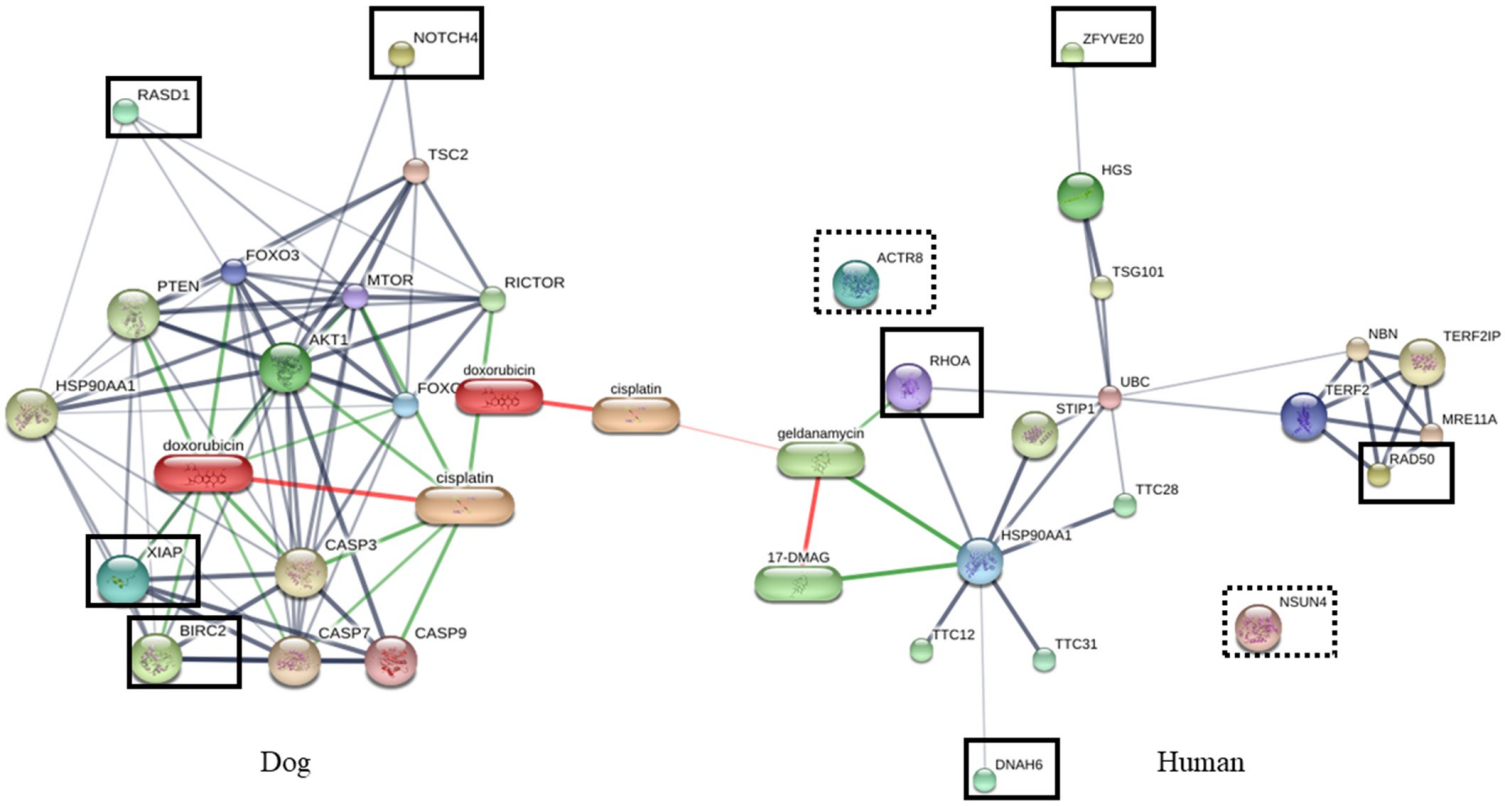
Protein-drug interaction network of proteins dominate in human and dogs. Interaction network was predicted by STITCH version 5.0 (http://stitch.embl.de). Proteins network related to anticancer agents. Bold box: proteins related to the drugs, Dashed box: proteins non-related to the drugs.

**Fig 7:**
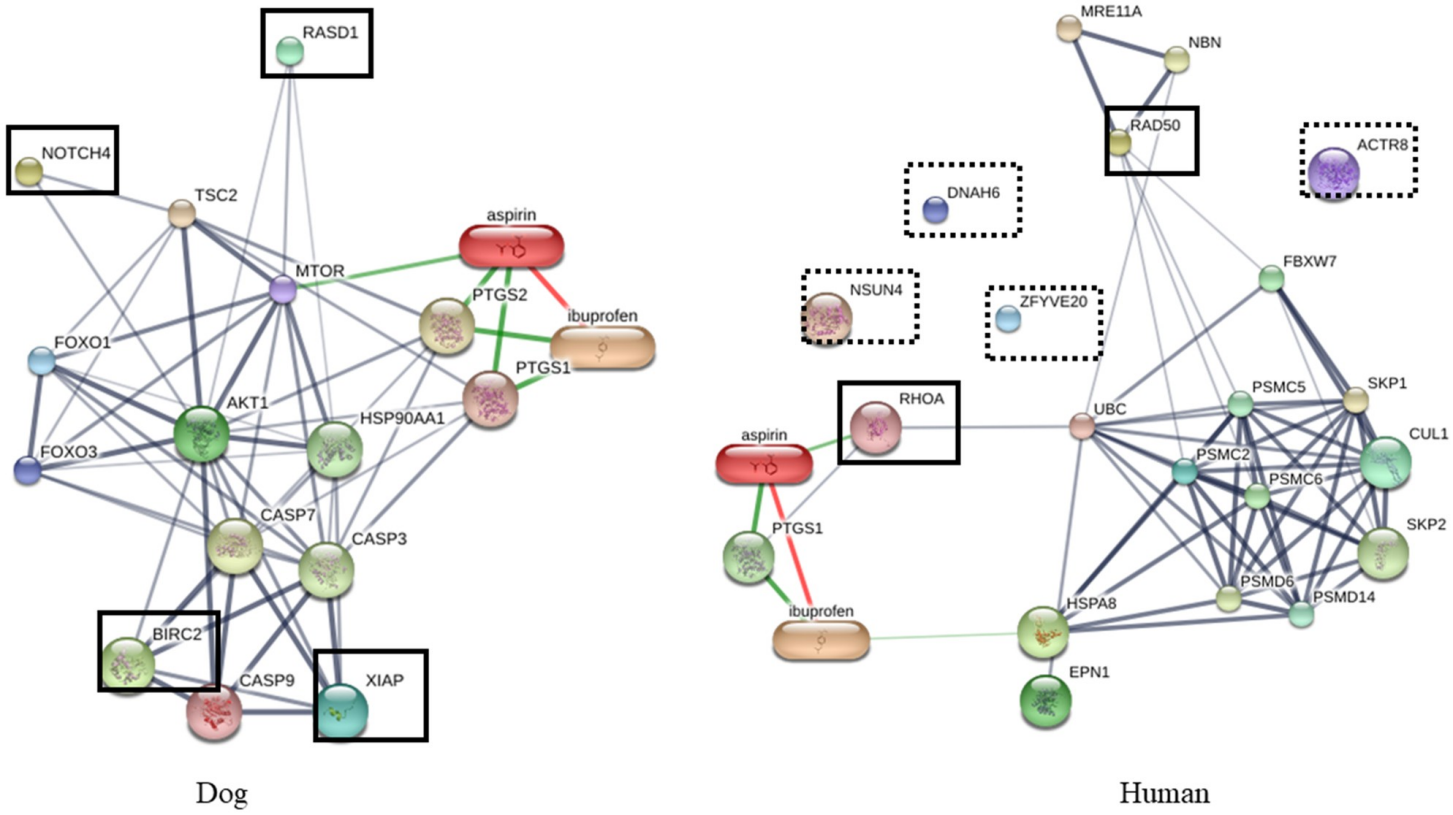
Protein-drug interaction network of proteins dominate in human and dogs. Interaction network was predicted by STITCH version 5.0 (http://stitch.embl.de). Proteins network related to anti-inflammatory agents. Bold box: proteins related to the drugs, Dashed box: proteins non-related to the drugs.

**Fig 8:**
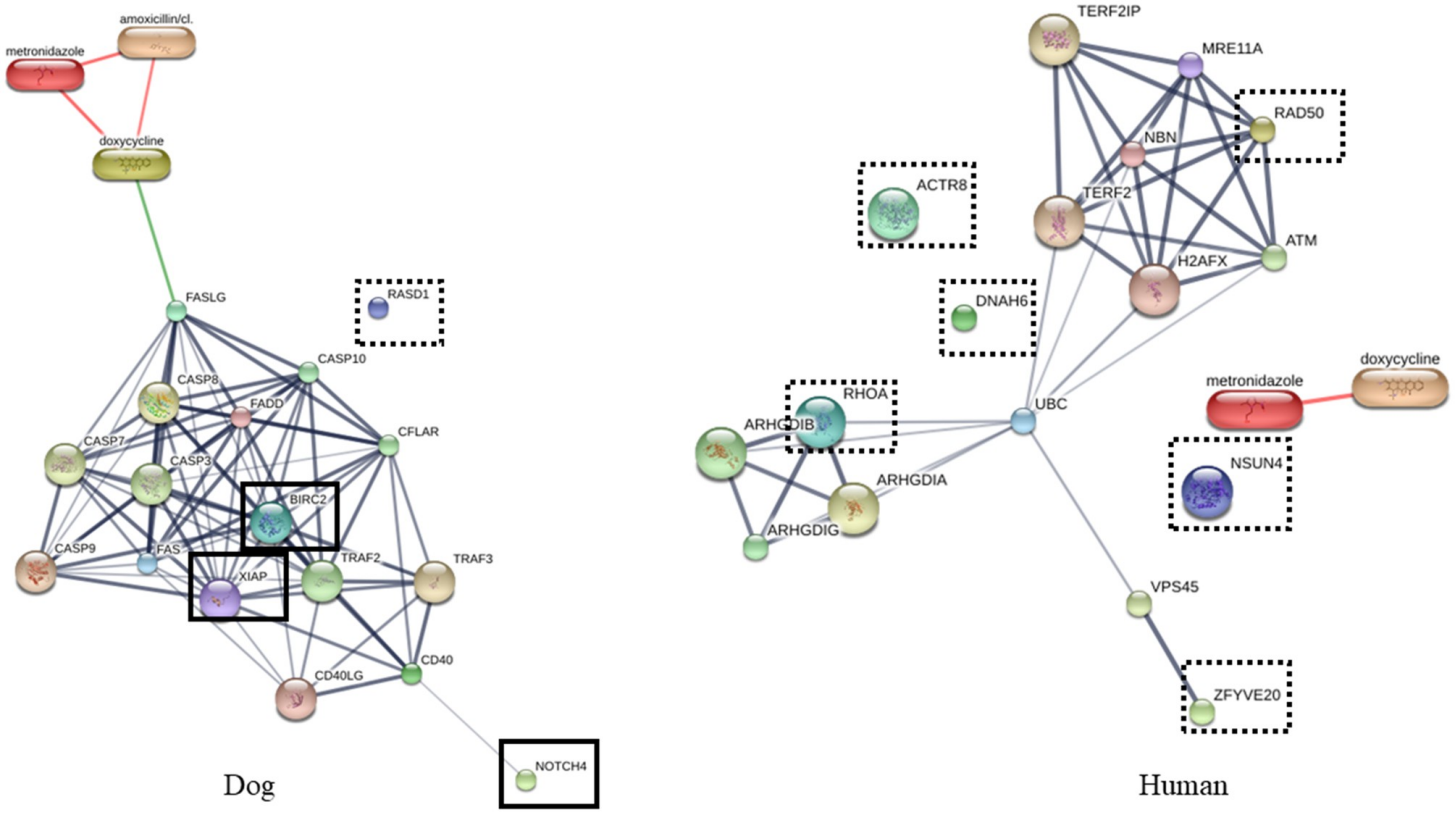
Protein-drug interaction network of proteins dominate in human and dogs. Interaction network was predicted by STITCH version 5.0 (http://stitch.embl.de). Proteins network related to antimicrobial agents. Bold box: proteins related to the drugs, Dashed box: proteins non-related to the drugs.

To determine the roles of XIAP, BIRC2, NOTCH4 and RASD1 in dogs and the proteins dominating in humans, which are NSUN4, ACTR8, RAD50, DNAH6, RHOA and ZFYVE20, interactions of the proteins were predicted by STITCH Version 5.0 (http://stitch.embl.de) (Figs 6–8). Proteins playing roles in apoptosis processes and biological adhesion were dominant in dog saliva. These functions are mainly associated with cancer. Therefore, these proteins were filtered and constructed with interaction networks using anti-cancer and anti-inflammatory agents, since inflammation always occurs with tumorigenesis. The proteins in dog were found to be more associated with cancer pathways (Fig 6) and inflammatory pathways than in human (Fig 7). Pathogenesis of cancer is related to dysregulation of apoptosis and the main problem in cancer treatment is chemotherapeutic resistance. Inhibitor of apoptosis proteins or IAPs (XIAP and BIRC2), and NOTCH signaling proteins are known to be involved in anti-apoptosis process and are elevated in many cancer cells, especially breast cancer [52–54]. The popular anti-cancer agents, doxorubicin and cisplatin, are associated with XIAP via protein kinase B (Akt) (Fig 6) [55]. Moreover, IAPs and NOTCH signaling proteins were also related to inflammatory pathway via Akt as shown in Fig 7. These proteins are related to inflammatory pathways by controlling homeostasis of many cells such as endothelial cells, smooth muscle cells, fibroblasts, lymphocytes and dendritic cells [56]. Since apoptosis resistance is one of the most important facets of tumorigenesis, alteration of these pathways might improve efficiency of chemotherapeutic drugs. Mammary gland tumor is the most common tumor in dogs, so higher level of these proteins in dog saliva may be related to the higher incidence rate of breast cancer in dogs than in human [52,57].

In addition, the antimicrobial properties of saliva were reported [58–61], and the interaction networks of some proteins obtained from shotgun proteomics and anti-bacterial agents including doxycycline, amoxicillin-clavulanic acid and metronidazole were analyzed by STITCH Version 5.0 (http://stitch.embl.de). The salivary proteins in the dog were found to be more associated with antimicrobial drugs than human as shown in Fig 8. The association between antimicrobial agents and the proteins was predicted via FAS/FAS ligand (FASLG) and Casp7. Anti-inflammatory and antimicrobial pathway of FASLG and Casp7 were reported [61,62]. Moreover, Torres et al. also reported that the proteins serve immune functions and show relations to antimicrobial mechanisms were the most abundant proteins in dog saliva [43]. Higher antimicrobial activities of dog salivary proteins than human was proposed. The ability of zoonotic pathogens in dog oral cavity to grow and transfer to humans may be regulated by these proteins.

Many methods have been reported for collecting dog saliva, however, no standard operating procedure has been set up [63]. Different salivary compositions have been obtained but these may be influenced by material collection methods such as cotton, polyester and polyethylene [64,65]. Since our study collected saliva directly from rom the dog’s mouth, from dripping or using a syringe to gently draw saliva out of the buccal cavity, there was no interference from collecting devices. Saliva volume and protein concentration differed considerably between dogs even though they had similar weights.

This study is one of the first to identify dog salivary proteins by using a shotgun proteomic technique. The main advantages of this proteomic technique are that low levels of a specific protein can be detected, and salivary proteins can be collected easily and noninvasively. However, the importance of saliva preparation has to be considered for downstream protein analysis. The inclusion of protease inhibitor and application of strict temperature control are recommended during saliva preparation, and separation of tissue material and debris is effected by centrifugation techniques. Proteins in samples were separated by molecular weight on one-dimensional gel electrophoresis, then bands were digested by trypsin and analyzed by tandem mass spectrometry. Overall, this technique can produce abundant protein information from small amounts of samples and has high sensitivity.

Proteome information from dog saliva has been limited, resulting in difficulties in applying to veterinary medicine. Here we have established novel dog saliva proteome data for Thai village dogs. The use of saliva as a non-invasive technique for developing disease diagnostics, therapeutics and prevention in the veterinary field has a promising future. Our findings are not only useful for animal health science but also have potential of utilizing dogs as an animal model. Since this study used only Thai village dogs, it should be of interest to extend the study to more varieties of breed to be able to better understand about behavior and salivary composition.

## Supporting information

S1 Table 2,532 differentially expressed proteins found in dogs and human (log 2 value). (PDF)

S2 Table Proteins found only in dogs. (PDF)

S3 Table Proteins found only in human. (PDF)
