# Supplementary material for: Comparative proteomic study of dog and human saliva
Source: PLoS One. 2018 Dec 4;13(12):e0208317. doi: 10.1371/journal.pone.0208317 (PMC6279226; doi:10.1371/journal.pone.0208317)
Supplement: S1 Table — (PDF) [file pone.0208317.s001.pdf]

Supplementary data 1 : 2,532 differentially expressed proteins found in dogs and human

**Protein name**	**Accession number**	**ID Score**	**Peptide**	**DOG**	**HUMAN**
0501254A protein Tro alpha1 H,myeloma	gi|223069	61.700001	QQPSQGTTTFAVTSILR	7.260226	7.182228
0506249A Ig Aalpha1 Bur	gi|223099	68.269997	QEPSZGTTTFAVTSILR	8.546066	8.331213
0709220A Ig kappa L I Den	gi|223335	41.599998	SGTASVVCLLDDFYPR	3.822825	6.332503
0808265A dismutase,Cu/Zn superoxide	gi|223480	0.61	KHGGPKBZER	10.47084	7.780565
1004299C T cell antigen receptor	gi|223910	18.25	KSTXVKGK	7.746826	7.135264
14-3-3 protein zeta/delta-like [Canis lupus familiaris]	gi|359321774	19.129999	MVSSSVNIYETGHK	12.84868	10.30434
1807128A tripeptidyl peptidase II	gi|228585	7.8499999	IPLHPASMATAATEEPFPFHGLLI	10.81701	8.559608
182 kDa tankyrase-1-binding protein isoform X2 [Homo sapiens]	gi|530397526	3.1300001	AEPDPGR	8.288301	6.312349
1-acyl-sn-glycerol-3-phosphate acyltransferase epsilon [Homo sapiens]	gi|61743952	15.25	LSIKKTLPSMLILSGLTAGMLMT	7.4379	6.446205
1-phosphatidylinositol 4,5-bisphosphate phosphodiesterase beta-2 isoform X2 [Canis lupus familiaris]	gi|545549407	5.3600001	FVDYNKR	5.662716	5.402674
1-phosphatidylinositol 4,5-bisphosphate phosphodiesterase beta-2 isoform X8 [Homo sapiens]	gi|578827092	23.49	KKRSLPR	10.3463	9.987351
1-phosphatidylinositol 4,5-bisphosphate phosphodiesterase delta-4 isoform X5 [Homo sapiens]	gi|530371087	19.620001	KKAMSLCSTILGS	5.823864	6.544563
2112198A Na channel:SUBUNIT=beta	gi|1096608	12.24	KEKLESMK	0	8.369205
2208436B hepatocyte nuclear factor 4	gi|1588511	18.049999	TMSLTSKCPEAKAMELGGVETC	5.426034	8.518025
26S protease regulatory subunit 4 [Homo sapiens]	gi|24430151	17.129999	KKKYEPPVPTR	8.01456	8.123689
26S proteasome non-ATPase regulatory subunit 1 isoform 2 [Homo sapiens]	gi|300388183	15.13	VSTAVLSITAKAK	8.488806	7.518966
28S ribosomal protein S14, mitochondrial [Homo sapiens]	gi|11559927	16.43	HLADHGQLSGIQR	10.20177	1.745648
28S ribosomal protein S24, mitochondrial precursor [Homo sapiens]	gi|15721937	14.52	AASVCSGLLGPRVLSWSR	6.666831	7.888187
28S ribosomal protein S34, mitochondrial [Canis lupus familiaris]	gi|359319743	4.2600002	SKGTAV	4.795248	3.783552
2-hydroxyacyl-CoA lyase 1 isoform b [Homo sapiens]	gi|548923731	1.34	YIGMR	9.513807	9.462811
2-oxoglutarate dehydrogenase E1 component DHKTD1, mitochondrial [Homo sapiens]	gi|38788380	11.72	SVEVPR	6.451146	6.459344
39S ribosomal protein L11, mitochondrial isoform X1 [Homo sapiens]	gi|578821681	5.3200002	AGLAMPGPPLGPVLGQR	7.77847	0
39S ribosomal protein L33, mitochondrial-like [Canis lupus familiaris]	gi|545531082	16.15	SKTILVKMLSQAGTGYSFNTK	7.079988	3.868963
39S ribosomal protein L45, mitochondrial isoform 2 [Homo sapiens]	gi|507116145	11.28	AAPIPQGFSCLSR	2.333895	9.336543
39S ribosomal protein L48, mitochondrial isoform X1 [Homo sapiens]	gi|530396892	21.91	HLIKAEEPKKK	9.206687	6.839946
5,6-dihydroxyindole-2-carboxylic acid oxidase isoform X1 [Homo sapiens]	gi|530390133	14.04	RNLLDLSKEEK	9.770824	8.613402
5-hydroxytryptamine receptor 1F [Canis lupus familiaris]	gi|545550782	22.700001	AITDAVK	8.500835	11.67793
5-methylcytosine rRNA methyltransferase NSUN4 isoform a [Homo sapiens]	gi|40316918	16.139999	VDLATVPR	0	10.60365
5-phosphohydroxy-L-lysine phospho-lyase isoform X14 [Homo sapiens]	gi|578811260	12.9	ADTLALR	10.77486	7.930489
60S ribosomal protein L13 isoform 2 [Homo sapiens]	gi|341604768	20.120001	TIGISVDPR	9.561173	0
60S ribosomal protein L13a isoform X1 [Canis lupus familiaris]	gi|345786084	17.450001	MAEGQVLVLDGR	9.418341	10.96939
60S ribosomal protein L21-like [Canis lupus familiaris]	gi|545515720	2.3699999	MTNTKVKRR	5.52629	7.200642
60S ribosomal protein L29 [Homo sapiens]	gi|4506629	15.9	EVKPKIPK	7.647213	9.113137
754931A hemoglobin zeta	gi|229556	21.85	VDPVNFR	8.529692	8.16663
85/88 kDa calcium-independent phospholipase A2 isoform X1 [Homo sapiens]	gi|578837422	13.25	GEHGNTPLHLAMSK	10.04343	12.98606
A Chain A, Back Pocket Flexibility Provides Group-ii Pak Selectivity For Type 1 Kinase Inhibitors	gi|584580144	9.5299997	XLVGTPYWMAPELISR	7.919578	9.748495
A Chain A, Crystal Structure Of A Domain Of 26s Proteasome Regulatory Subunit 8 From Homo Sapiens. Northeast Structural Genomics Consortium Target Id Hr3102a	gi|281500993	16.389999	XNLTRGINLR	10.16274	12.97241
A Chain A, Crystal Structure Of A Domain Of Brefeldin A-Inhibited Guanine Nucleotide-Exchange Protein 2 (Brefeldin A-Inhibited Gep 2) From Homo Sapiens (Human), Northeast Structural Genomics Consortium Target Id Hr5562a	gi|344189841	9.5500002	IDRLXEK	8.811718	9.372358
A Chain A, Crystal Structure Of Abba+udp+gal At Ph 9.0 With Mpd As The Cryoprotectant	gi|568786492	25.200001	FMVSLPR	7.641259	8.563803
A Chain A, Crystal Structure Of Dual Specificity Protein Phosphatase 23 From Homo Sapiens In Complex With Ligand Malate Ion	gi|118138468	4.21	TGTXLACYLVK	0	7.140872
A Chain A, Crystal Structure Of Human Catenin-beta-like 1 56 Kda Fragment	gi|528082092	11.5	VHQILNXRGSSIKIVR	9.08635	9.402261
A Chain A, Crystal Structure Of Human Enolase-Phosphatase E1	gi|71042465	8.3599997	EAGXKVYIYSSGSVEAQK	8.281619	6.936657
A Chain A, Crystal Structure Of Human Gtpase Imap Family Member 4	gi|290790349	4.4400001	QTQAX	11.0826	9.023847
A Chain A, Crystal Structure Of Phosphorylated P38 Alpha In Complex With Dp802	gi|306991845	19.09	HTDDEMTGXVATR	12.26716	11.12628
A Chain A, Crystal Structure Of Snx9px-Bar (230-595), C2221	gi|203282517	5.6900001	AXDDGVK	7.363827	8.290828
A Chain A, Crystal Structure Of Synaptotagmin I C2a Domain With Cu(Ii)	gi|257471779	13.03	GSPGIGGGGGGILDSXVEKLGK	7.324289	10.9989
A Chain A, Crystal Structure Of The Catalytic Domain Of Human Diphosphoinositol Pentakisphosphate Kinase 2 (Ppip5k2) In Complex With Adp And In The Absence Of Cadmium At Ph 7.0	gi|359545937	0.9	GSFTDSPPER	7.203394	6.822063
A Chain A, Crystal Structure Of The Complex Between Dosage Compensation Factors Msl1 And Mof	gi|317455089	15.96	YWKGQHVIXVTPK	10.56653	10.00014
A Chain A, Crystal Structure Of The Efc Domain Of Formin-binding Protein 17	gi|149241439	20.639999	QQAQIRHQXAEDSK	7.346663	9.729236
A Chain A, Crystal Structure Of The Paz Domain Of Human Eif2c1 In Complex With A 9-Mer Sirna-Like Duplex	gi|49259004	24.639999	KLTDNQTSTXIKATAR	10.49945	6.960381
A Chain A, Crystal Structure Of The Ubiquitin-Like Domain Of Plexin D1	gi|237824059	5.9400001	GAKPRNLNVSFQGCGXDSLSVR	9.842659	10.01006
A Chain A, Crystal Structure Of Truncated (delta 1-89) Human Methionine Aminopeptidase Type 1 In Complex With 2-((5-chloro-6-methyl-2- (pyridin-2-yl)pyrimidin-4-yl)amino)-3-phenylpropanamide	gi|564731025	9.8599997	GSHMLEDPYR	0	4.47196
A Chain A, Dbl And Pleckstrin Homology Domains From Hsos1	gi|6729800	12.6	KXNEIQK	5.926031	0
A Chain A, Ensemble Refinement Of The Protein Crystal Structure Of Human Phosphomannomutase 2 (Pmm2)	gi|343197792	15.11	TXPGGNDHEIFTDPR	7.032403	9.228109
A Chain A, Human Alpha-Phosphomannomutase 1 With D-Mannose 1-Phosphate And Mg2+ Cofactor Bound	gi|93279753	2.8699999	XAVTAQAARR	5.139057	2.963398
A Chain A, Human Cdc25a Catalytic Domain	gi|157830510	12.01	LIGDFSKGYLFHTVAGKHQDLK	9.164802	9.913884
A Chain A, Human Centromere Protein B (Cenp-B) Dna Bindign Domain Rp1	gi|6729749	5.0799999	RAILASE	8.738173	4.987697

A Chain A, Human Cyclic Gmp-amp Synthase (cgas) In Complex With Sulfate Ion	gi|582045649	7.8699999	MGASK	4.994516	5.434201
A Chain A, Mechanistic Analyses Of Catalysis In Human Pancreatic Alpha-amylase: Detailed Kinetic And Structural Studies Of Mutants Of Three Conserved Carboxylic Acids	gi|20664074	28.5	XYSPNTQQGR	9.482169	9.811908
A Chain A, Molecular Insights Into Pebp2CBF-Smmhc Associated Acute Leukemia Revealed From The Three-Dimensional Structure Of Pebp2CBF BETA	gi|6730180	4.3000002	XVPDQRSK	9.002174	9.008707
A Chain A, Pepck Complex With Nonhydrolyzable Gtp Analog, Mad Data	gi|20150792	12.08	LLGQXEEEGILRR	8.489646	9.847544
A Chain A, Phf19 Links Methylated Lysine 36 Of Histone H3 To Regulation Of Polycomb Activity	gi|410562756	11.66	SKLTEGQYVLCR	4.625334	3.57804
A Chain A, Plk1 In Complex With Bi6727	gi|237640531	15.04	GPAPADPGK	10.33522	10.60798
A Chain A, Rbx1	gi|381352847	6	GGGGTNSGAGK	8.449682	6.633584
A Chain A, Solution Structure Of Human Ubiquitin Fusion Degradation Protein 1 Homolog Ufd1	gi|183448198	18.91	GSSGSSGIPR	9.915663	8.935296
A Chain A, Solution Structure Of The 13th Filamin Domain From Human Filamin-B	gi|159164142	18.870001	VLPTYDAS	10.36027	5.715164
A Chain A, Structural Basis Of The Interaction Of Rbap46RBAP48 WITH Histone H4	gi|190016329	11.17	YXPQNPHIIATK	8.09926	9.157025
A Chain A, The Catalytically Active Fully Closed Conformation Of Human Phosphoglycerate Kinase K219a Mutant In Complex With Amp-Pcp And 3pg	gi|315583375	15.81	VADAIQLINNMLDK	8.10113	9.080448
A Chain A, The Helix-Hinge-Helix Structural Motif In Human Apolipoprotein A-I Determined By Nmr Spectroscopy, 1 Structure	gi|157831263	10.87	SPLGEEMR	7.465414	6.8384
A disintegrin and metalloproteinase with thrombospondin motifs 9 isoform X1 [Homo sapiens]	gi|530372870	10.62	YSGILMK	5.738455	5.672085
A42856 EPF autoantibody-reactive epitope - human (fragment)	gi|345836	10.99	RTQEGGRGDPPPAGR	6.652375	9.897567
abnormal spindle-like microcephaly-associated protein isoform 1 [Homo sapiens]	gi|126116596	16.41	KSTVILQALVR	9.565392	7.498548
AC006020_1 lysine ketoglutarate reductase/saccharopine dehydrogenase [Homo sapiens]	gi|6094681	11.04	ALNGFVK	7.703881	4.195912
AC006036_1 unknown, partial [Homo sapiens]	gi|6624100	16.91	SLLKGLK	7.538052	7.912916
AC007055_3 unknown [Homo sapiens]	gi|4885694	10	MEADR	5.431897	8.968805
AC007075_1 unknown, partial [Homo sapiens]	gi|6094660	9.5799999	LSSSVEYNIMELEQELENVK	5.734864	8.480821
AC007277_1nonhistone chromosomal protein HMG-1 [Homo sapiens]	gi|6624099	8.1499996	DKGKFEDMAKVDK	3.662609	6.483268
acetoacetyl-CoA synthetase [Canis lupus familiaris]	gi|345791123	14.03	ATAEVSLTWPGLR	9.924475	9.693095
acetyl-CoA carboxylase 1 isoform X3 [Homo sapiens]	gi|578840348	19.85	GVISDILDWK	12.336	10.35979
Acid phosphatase, prostate [Homo sapiens]	gi|16740983	14.59	GEYFVEMHYR	9.332995	9.689149
acidic amino acid decarboxylase GADL1 [Homo sapiens]	gi|197383062	19.309999	EKGLSGSPR	12.82002	11.06929
acidic leucine-rich nuclear phosphoprotein 32 family member B [Homo sapiens]	gi|5454088	7.8499999	MDMKRR	0	7.080008
actin cytoskeleton-regulatory complex protein PAN1-like [Homo sapiens]	gi|578813146	20.190001	MGMGARGADTEFPR	8.542169	8.816197
actin, cytoplasmic 2 isoform X2 [Homo sapiens]	gi|578831330	41.919998	VAPEEHPVLLTEAPLNPK	7.716703	10.24218
actin-like protein 7A [Canis lupus familiaris]	gi|545517855	27.82	KETFVGK	5.230852	8.5354
actin-related protein 8 isoform X1 [Homo sapiens]	gi|578806774	10.37	MYSSILVVGGGLMFHK	0	7.903813
activating signal cointegrator 1 complex subunit 1 isoform X6 [Homo sapiens]	gi|578819520	10.43	HIVGK	9.70254	10.38998
active breakpoint cluster region-related protein isoform X2 [Homo sapiens]	gi|578829853	5.79	RWMHLIPGGR	8.491817	8.588332
activity-dependent neuroprotector homeobox protein isoform X6 [Canis lupus familiaris]	gi|545541077	7.3499999	SKPLMLIAPKPQDKKGMGLQSR	5.624545	8.922422
acylamino-acid-releasing enzyme isoform X7 [Homo sapiens]	gi|530372382	12.17	TPLLLMLGQEDR	7.734431	12.86336
acyl-CoA synthetase short-chain family member 2, isoform CRA_a [Homo sapiens]	gi|119596648	11.51	QGMSVSPSGVMPR	9.966078	7.932153
acyl-CoA-binding protein isoform 4 [Homo sapiens]	gi|295849266	24.43	WGKGLHGLEER	6.522265	7.086978
acyl-coenzyme A synthetase ACSM5, mitochondrial isoform X2 [Canis lupus familiaris]	gi|545502024	11.31	KAANVLQGMCGLQPGDR	8.767651	6.933742
acyl-coenzyme A thioesterase 11 isoform 2 [Homo sapiens]	gi|22165400	17.5	AIEMFHFR	5.667646	5.647771
acyl-coenzyme A thioesterase 4 [Canis lupus familiaris]	gi|73964279	13.53	LLNKPVIWGGEPRAHSK	2.650736	6.606323
adenomatous polyposis coli protein isoform a [Homo sapiens]	gi|306922386	11.55	GAATDEK	8.158453	8.823969
adenosine deaminase domain-containing protein 1 isoform X4 [Canis lupus familiaris]	gi|545531803	6.6500001	SGVSMASRLCKAAMLSRFNLLA	12.28548	8.461529
adenosine deaminase domain-containing protein 2 isoform X2 [Homo sapiens]	gi|578828875	5.6199999	GLSLNWSLGDPGIEVVDVATGR	7.121738	4.054497
adenosine receptor A1 isoform X1 [Canis lupus familiaris]	gi|545503862	22	YKTVVTPR	9.598717	9.827391
adenylate cyclase type 5 isoform X1 [Homo sapiens]	gi|530373838	31.83	KFPSDKLER	8.08709	8.006925
adenylate cyclase type 6 isoform X7 [Canis lupus familiaris]	gi|545545120	4.8699999	YMSCLRDAQPPSPTPAAPPR	6.358648	7.984266
adenylate cyclase type 9 isoform X1 [Homo sapiens]	gi|530407541	17.860001	VGQSIMHGK	13.20376	8.666822
adhesion G protein-coupled receptor A3 [Canis lupus familiaris]	gi|545494295	9.3599997	SYGLNLAVPNGPGRGR	6.637468	5.96855
ADP-dependent glucokinase isoform X2 [Homo sapiens]	gi|578827470	12.16	MGKGAAAER	6.11025	4.295895
ADP-ribosylation factor-like protein 6-interacting protein 4 isoform 5 [Homo sapiens]	gi|507834130	2.22	AGLLP	10.06967	1.255498
ADP-ribosylation factor-like protein 6-interacting protein 4 isoform 6 [Homo sapiens]	gi|507834132	10.61	SPRGAMAHVGSRK	6.415981	6.434735
AF063596_1 brain my038 protein [Homo sapiens]	gi|12002024	7.77	EGKGRLVLRYLVAGER	7.849979	12.14842
AF083884_1 mutant beta-globin [Homo sapiens]	gi|4929547	24.530001	SALLPCGAR	9.33185	9.175528
AF107021_1 sex determining region Y protein [Canis lupus familiaris]	gi|5114118	2.3499999	AAHTR	9.466011	10.26732
AF111707_1 SLTP004 [Homo sapiens]	gi|20146522	8.4099998	MTNTIHI	8.769938	8.444674
AF119917_31 PRO2249 [Homo sapiens]	gi|7770175	11.59	MDLPTCGAR	7.994611	0
AF130117_28 PRO2277 [Homo sapiens]	gi|11493445	9.71	ATNYMIPFLGSVQNMQIHGDR	9.636664	11.06007
AF130420_1 serine protease-like protein isoform [Homo sapiens]	gi|4883898	10.09	DMNSEK	5.171869	3.913022
AF132960_1 CGI-26 protein [Homo sapiens]	gi|4680691	15.7	MVTAFR	7.677197	6.380613
AF148646_1 mitofilin, partial [Homo sapiens]	gi|8131894	19.809999	ARAPASLTAGDTLSVPAPAVQPI	6.122319	7.992194
AF151046_1 HSPC212 [Homo sapiens]	gi|7106814	11.64	NLQGRLFISLLMKNLLSEDMR	8.14045	11.77586
AF153062_1 type I collagen pre-pro-alpha1(I) chain [Canis lupus familiaris]	gi|4960163	7.4699998	GETGPAGPAGPIGPVGAR	6.31875	7.946358
AF161491_1 HSPC142 [Homo sapiens]	gi|6841506	7.9200001	GGGVPGGT	6.806076	5.901448
AF163258_1 MSTP067 [Homo sapiens]	gi|33337920	22.85	SLKISGKEKNLK	0	6.739758
AF163261_1 MSTP070 [Homo sapiens]	gi|33337926	12.25	VEENPYR	9.176153	6.491119
AF164613_1 Gag-Pro-Pol protein [Homo sapiens]	gi|5802819	12.34	GPELMGPSESKPR	0	10.50061
AF165917_1 triadin isoform 3 [Canis lupus familiaris]	gi|6002950	10.45	TSPILVISTTCR	6.192982	8.601966
AF192529_1 RPA-binding trans-activator [Homo sapiens]	gi|6180045	7.8499999	EGGLKR	7.258287	7.356907
AF193421_1 ARC [Homo sapiens]	gi|6319152	7.6700001	AEMLEHVR	6.067247	7.388912
AF201378_1 uncoupling protein 3, partial [Canis lupus familiaris]	gi|6425122	10.76	VRFQA	9.545025	8.910919
AF224266_1 four alpha helix cytokine [Homo sapiens]	gi|7109207	14.4	NGFSDIR	6.045823	8.295284

AF230533_1 nuclear receptor coactivator CIA, partial [Homo sapiens]	gi|11526821	11.62	AGSDINK	3.693118	6.127505
AF235777_1 immunoglobulin heavy chain variable region, partial [Homo sapiens]	gi|13171890	7.1300001	YGSGSYTTYYYYGMDVWGQ	6.541272	8.807602
AF235838_1 immunoglobulin heavy chain variable region, partial [Homo sapiens]	gi|13172012	13.07	DVGATLR	10.19453	10.31142
AF276808_1 Z-band alternatively spliced PDZ-motif protein ZASP-5 [Homo sapiens]	gi|33339651	13.33	ASGVGLPGGW	7.43704	6.844321
AF280088_1 L-selectin ligand sulfotransferase GST-3 [Homo sapiens]	gi|12060808	22.1	WSLPYEKVSR	9.148273	9.998397
AF288392_1 C1orf26 [Homo sapiens]	gi|12620194	13.77	EASYSNDNQIILQSPSSNGXKK	3.913747	6.063303
AF311904_1 membrane protein SB87 precursor [Homo sapiens]	gi|22266724	17.370001	GAQVLLAPR	10.5689	8.438426
AF318374_1 unknown [Homo sapiens]	gi|18027840	11.46	TRGTQGLPMTMGPR	8.35708	8.276655
AF322907_1 NSD1 [Homo sapiens]	gi|15213542	20.190001	GWGLRTQEDIR	9.160419	11.36108
AF329839_1 complement-c1q tumor necrosis factor-related protein [Homo sapiens]	gi|13274524	7.5	GTAGLR	8.438944	8.714767
AF351784_1 dopamine receptor interacting protein, partial [Homo sapiens]	gi|14194057	7.9000001	AWDIVSNAEKRK	6.178251	4.900636
AF354265_1 immunoglobulin gamma heavy chain B [Canis lupus familiaris]	gi|17066526	38.790001	NTVSLTCLIK	12.26854	7.553806
AF354267_1 immunoglobulin gamma heavy chain D [Canis lupus familiaris]	gi|17066530	58.060001	VVSVLPIEHQDWLTGK	7.818028	9.540827
AF370406_1 PP11647 [Homo sapiens]	gi|33341742	21.700001	RGLSSMRSIPR	10.47038	6.077522
AF390108_1 glycoprotein receptor SLAM1 [Canis lupus familiaris]	gi|15420786	9.3400002	LGSSLQLSLASEGISKR	5.037005	9.667121
AF460670_1 immunoglobulin heavy chain variable region, partial [Homo sapiens]	gi|22000332	18.98	AIVVVTR	9.508837	11.03689
AF471290_1 Ig heavy chain variable region, VH3 family, partial [Homo sapiens]	gi|33319124	10.1	GQSRDFR	6.903502	8.770599
AF471465_1 Ig heavy chain variable region, VH3 family, partial [Homo sapiens]	gi|33319474	23.459999	QERLVESGGGVVQPGR	5.72323	8.669589
AF493899_1 guanine nucleotide binding protein alpha z [Homo sapiens]	gi|20147691	13.48	KDLLAEKIR	8.600992	6.55899
AF493900_1 guanine nucleotide binding protein alpha 11 [Homo sapiens]	gi|20147693	11.84	RGFTKLVYQNIFTAMQAMIR	7.511877	5.39384
afadin isoform X8 [Homo sapiens]	gi|578812783	12.16	NENDAIPPK	9.477701	10.1511
AFG3-like protein 2 [Homo sapiens]	gi|300192933	28.209999	SSGGGGGGGGKRGGK	7.745628	8.037815
agrin isoform X9 [Canis lupus familiaris]	gi|545499001	18.15	APDVGQALLR	10.80421	11.19715
AIM1 protein, partial [Homo sapiens]	gi|38541016	12.93	MSALGTAFSREGGRK	10.05647	11.92087
A-kinase anchor protein 11 isoform X5 [Homo sapiens]	gi|530402083	17.77	SKNKSLMIK	12.72713	10.55293
alanine--tRNA ligase, mitochondrial isoform X2 [Homo sapiens]	gi|530382281	27.09	AATERLSLGSR	8.073298	13.52918
albumin, partial [Homo sapiens]	gi|332356380	58.639999	DVFLGMFLYEYAR	8.669618	7.773461
alcohol dehydrogenase, iron containing, 1, isoform CRA_a [Homo sapiens]	gi|119607307	18.08	DDLKNMGAK	7.623588	7.981988
aldehyde oxidase [Homo sapiens]	gi|438656	14.62	FPVGLASR	2.372029	5.066715
allergen Fel d 4 isoform X1 [Canis lupus familiaris]	gi|545517938	48.16	ENILDLTQVDR	9.871632	4.860644
allograft inflammatory factor 1 isoform X2 [Homo sapiens]	gi|530429520	9.0900002	KAISELP	3.12919	4.189597
alpha 1 type VII collagen precursor variant, partial [Homo sapiens]	gi|62088870	3.6700001	AGDPGR	10.98496	8.312806
alpha 2 type V collagen preproprotein variant, partial [Homo sapiens]	gi|62087670	24.639999	GLVGPPGSR	8.792179	7.642372
alpha- and gamma-adaptin-binding protein p34 isoform X3 [Homo sapiens]	gi|578827398	18.66	SGLDSVSSWLPLAK	13.63952	13.26892
alpha-1,6-mannosylglycoprotein 6-beta-N-acetylglucosaminyltransferase B isoform 2 [Homo sapiens]	gi|39812197	12.34	QGTMALPALLTR	12.24576	11.46711
alpha-1-syntrophin [Canis lupus familiaris]	gi|545541499	8.5500002	MAYVSR	6.57871	7.830904
alpha-1-syntrophin isoform X1 [Homo sapiens]	gi|530418323	12.68	VKDELQALLAATSTAGSQDIK	12.61007	6.820075
alpha-2-macroglobulin isoform X1 [Homo sapiens]	gi|578822814	3.1600001	SNHVSR	5.342183	7.243071
alpha-amylase 1 isoform X3 [Homo sapiens]	gi|578798954	91.580002	TGSGDIENYNDATQVR	6.359103	8.657125
alpha-catulin isoform X4 [Homo sapiens]	gi|530391734	15.19	KAKMAAARAVLEK	5.514911	4.741921
alpha-fetoprotein [Canis lupus familiaris]	gi|22218072	22.129999	YIYEIAR	8.88273	8.580298
alpha-ketoglutarate-dependent dioxygenase alkB homolog 2 isoform X1 [Homo sapiens]	gi|530401197	9.8100004	RVAVVR	1.574263	7.184875
alpha-ketoglutarate-dependent dioxygenase alkB homolog 6 isoform X5 [Homo sapiens]	gi|578835064	12.86	SICFDR	3.320485	7.048815
alpha-mannosidase 2x isoform X1 [Canis lupus familiaris]	gi|545493501	9.6999998	VDEEQERR	5.632042	7.013686
Alstrom syndrome protein 1 [Homo sapiens]	gi|110349786	2.04	AGDTSK	9.872054	8.861779
Alstrom syndrome protein 1 isoform X6 [Canis lupus familiaris]	gi|73980988	14.56	ALQQSEKYLTK	9.906689	9.233207
alternative protein AVL9 [Homo sapiens]	gi|444738837	9.3000002	HEVKVLTFCSE	5.943213	8.313643
alternative protein DENND1B [Homo sapiens]	gi|444738091	10.41	KLLLISMRVMTLLK	0	6.577109
alternative protein ELF1 [Homo sapiens]	gi|444738113	17.049999	MMMMTSPLQLKLLVMTGMK	10.05722	9.978243
alternative protein FAM171A1 [Homo sapiens]	gi|444737925	11.28	TMMMMTKEK	6.029122	5.201603
alternative protein KCTD3 [Homo sapiens]	gi|440575889	19.129999	KVILQVR	7.658882	9.023906
alternative protein MAPKBP1 [Homo sapiens]	gi|444738065	16.91	RPSMTWMWSPAGSTR	2.474233	5.21016
alternative protein MKL1 [Homo sapiens]	gi|444739169	5.5100002	HPVPR	8.737327	9.229498
alternative protein POLDIP2 [Homo sapiens]	gi|444738905	2.1800001	MMTVGPSMPSQAWTMSAMK	6.238066	8.307273
alternative protein SSRP1 [Homo sapiens]	gi|444738273	10.87	MPMTAAMTQEK	7.242852	8.7857
alternative protein TNK1 [Homo sapiens]	gi|444738357	15.71	CLLPPMLLGF	8.532365	10.01315
alternative protein UBE2R2 [Homo sapiens]	gi|444739013	13.05	KMPTVMMMMILGMR	10.82411	4.947458
alternative protein ZNF644 [Homo sapiens]	gi|440576095	12.25	KSLMPR	4.173955	0
aminopeptidase N, partial [Homo sapiens]	gi|178533	14.16	SLGILG	6.498561	4.510413
AMP deaminase 2 isoform X2 [Homo sapiens]	gi|578798942	3.98	FIKRAMK	7.158061	0
amyloid beta A4 precursor protein-binding family B member 1 isoform X4 [Homo sapiens]	gi|578820490	18.280001	SPGGAVMGR	3.585204	7.746358
amyloid precursor protein-binding protein 1 (APP-B1) [Homo sapiens]	gi|3242733	35.650002	ALKEFVAK	8.669019	7.104792
amyloid-like protein 2 isoform 5 [Homo sapiens]	gi|343478153	13.77	AVMPRWYFDLSKGK	7.748542	6.830237
amyotrophic lateral sclerosis 2 chromosomal region candidate gene 11 protein isoform X7 [Homo sapiens]	gi|578803842	9.1899996	ISINKAVKCTK	10.15625	8.697011
angiopoietin-2 isoform c precursor [Homo sapiens]	gi|169646754	22.41	GLTGTAGKISSISQPGNDFSTK	8.223414	4.024355
angiopoietin-related protein 5 isoform X1 [Homo sapiens]	gi|578822105	10.68	MHLGRYSGNAGDAFR	6.281992	6.76473
angiotensin II type 1b receptor [Homo sapiens]	gi|471121	17.059999	KAYEIQKNNPR	7.23793	7.367325
angiotensin-converting enzyme isoform X2 [Homo sapiens]	gi|578830520	19.24	SEGPLPDSGR	10.02878	9.795457
ankycorbin isoform X3 [Canis lupus familiaris]	gi|545496448	16.120001	REKENIQALFKAK	8.254261	8.176067
ankyrin repeat and SOCS box protein 12 [Homo sapiens]	gi|320461689	13.53	MRIVLQLAKMNLMDITK	8.559687	10.07298
ankyrin repeat and SOCS box protein 18 [Homo sapiens]	gi|157743292	15.37	MSNSDYLPDYPLNSDLVKR	7.471794	9.951761
ankyrin repeat domain 11 variant, partial [Homo sapiens]	gi|62087952	18.290001	KETKSNSFISIPSKMELK	12.51716	9.000116
ankyrin repeat domain-containing protein 11 isoform X5 [Homo sapiens]	gi|578828959	12.97	EKLLGDGDLMMTSFER	13.66286	9.651384

ankyrin repeat domain-containing protein 60 [Homo sapiens]	gi|578846829	16.540001	DDTTLK	7.763856	7.209804
ankyrin repeat domain-containing protein 7 [Homo sapiens]	gi|118150656	12.2	TALILAVSGEPPCLVK	8.743327	9.924299
ankyrin-2 [Canis lupus familiaris]	gi|545552301	13.81	MTAILTTDVSDR	7.313551	9.264164
ankyrin-3 isoform X15 [Homo sapiens]	gi|578819314	6.5900002	SGAIDMSK	7.678816	8.554372
ankyrin-3 isoform X29 [Homo sapiens]	gi|578819342	26.43	GETALHMAAR	9.504522	7.477953
ankyrin-3 isoformX1 [Canis lupus familiaris]	gi|545494924	13.06	GPPKSPK	9.968983	7.469891
annexin A11 isoform X3 [Homo sapiens]	gi|578819367	6.3499999	TAYGK	8.650026	8.417921
anoctamin-2 [Homo sapiens]	gi|326205433	16.200001	ANNTMGKDPSPR	9.609436	12.95751
anoctamin-2 isoform X11 [Homo sapiens]	gi|578822634	19.360001	ITTAAALSLNKATR	8.718766	8.265427
anoctamin-3 isoform 2 [Homo sapiens]	gi|156766084	5.6599998	NTFEKNLRAEGLMLEKEPAIASI	5.790953	6.186257
anoctamin-4 isoform X7 [Homo sapiens]	gi|578823315	14.73	YCPFMR	8.817742	9.838954
anoctamin-8 [Canis lupus familiaris]	gi|359322309	10.22	RLEPQAEEGGGGGGSGGRR	5.585476	5.591959
anti-c-erbB-2 immunoglobulin heavy chain V, partial [Homo sapiens]	gi|1145312	14.31	KPGESLKISXK	11.08547	10.31808
antigen KI-67 isoform X1 [Homo sapiens]	gi|578819493	23.040001	ELSALK	9.553925	4.972924
anti-HIV immunoglobulin heavy chain variable region, partial [Homo sapiens]	gi|344323217	4.3400002	TLHGR	9.47103	8.666526
anti-HIV-1 immunoglobulin heavy chain variable region, partial [Homo sapiens]	gi|343792789	18.389999	QSXLEQSGSAMKKPGASVR	8.189548	9.123112
anti-Rh(D) antibody immunoglobulin heavy chain variable region, partial [Homo sapiens]	gi|93007550	7.0100002	QXQLQESGPGLVKPSGTLSLTCA	8.982313	7.173729
anti-tetanus toxoid immunoglobulin heavy chain variable region, partial [Homo sapiens]	gi|353252936	13.04	NPGTSVR	3.727777	6.966998
anti-tetanus toxoid immunoglobulin light chain variable region, partial [Homo sapiens]	gi|353255046	14.4	APILLIYR	0	6.866848
anti-vaccinia virus immunoglobulin heavy chain variable region, partial [Homo sapiens]	gi|316925301	15.61	QSPSSGLEWLGR	9.213491	12.61854
AP-4 complex accessory subunit tepsin isoform X4 [Homo sapiens]	gi|578830440	0.7	AAAPPLR	9.527461	7.5826
AP-4 complex subunit epsilon-1 isoform X1 [Homo sapiens]	gi|578826761	12.51	ILGLLGK	9.561975	11.32304
AP-5 complex subunit beta-1 [Homo sapiens]	gi|379317153	16.459999	AALDGGPR	8.356732	7.44728
APC membrane recruitment protein 3 [Homo sapiens]	gi|157427661	14.87	CRDRVQDLSWLR	9.717669	11.54286
apolipoprotein A-I [Canis lupus familiaris]	gi|73955106	59.5	LREQIGPVTQEFWDNLEK	12.15795	10.39097
apolipoprotein A-I binding protein, isoform CRA_a, partial [Homo sapiens]	gi|119573326	26.23	AGGARSASWMSR	12.21537	8.443443
apolipoprotein B receptor [Canis lupus familiaris]	gi|545501703	8.9700003	TTSVGKGDDLSGAR	8.769869	8.22165
apolipoprotein H (beta-2-glycoprotein I), isoform CRA_b [Homo sapiens]	gi|119609419	9.0900002	WSPELPVCAR	8.171856	8.462708
apolipoprotein L2 isoform X2 [Homo sapiens]	gi|578837135	17.030001	KALNKLASHMVMK	10.25033	11.5936
apoptosis-stimulating of p53 protein 2 isoform X1 [Homo sapiens]	gi|530366444	29.26	ENGVNSPR	9.163218	9.363591
apoptosis-stimulating of p53 protein 2 isoform X6 [Canis lupus familiaris]	gi|545505242	17.120001	VVSGSRSQDPSVK	3.089194	5.005519
aprataxin and PNK-like factor [Homo sapiens]	gi|27734905	18.27	MSGGFELQPR	7.797244	8.422073
arf-GAP with GTPase, ANK repeat and PH domain-containing protein 2-like [Homo sapiens]	gi|578805484	10.19	GGSGR	3.551703	7.668318
arf-GAP with GTPase, ANK repeat and PH domain-containing protein 3 isoform X4 [Homo sapiens]	gi|578813905	22.120001	TTVKVPGK	9.201468	6.489548
arginine vasopressin receptor 1A [Canis lupus familiaris]	gi|309401190	17.33	MRIPGGPGAPSAGNSSR	7.793005	4.303482
armadillo repeat-containing protein 12 isoform X2 [Canis lupus familiaris]	gi|73972696	19.9	MGMSIPR	8.189094	7.7036
armadillo repeat-containing protein 2 isoform X2 [Homo sapiens]	gi|578812995	4.3699999	IILALK	3.775933	0
armadillo repeat-containing protein 6 isoform X1 [Homo sapiens]	gi|530415367	7.3800001	GNLAP	7.688869	7.010022
armadillo repeat-containing protein 6 isoform X2 [Canis lupus familiaris]	gi|545534082	11.35	AAHRDCEDVAKAALR	6.86864	7.78914
armadillo repeat-containing X-linked protein 1 [Homo sapiens]	gi|7706143	12.03	VGTISGNR	9.158006	6.865617
arylsulfatase G isoform X1 [Canis lupus familiaris]	gi|545509690	9.0100002	EILADVLLDIAGDNTSR	6.464994	8.591739
ashwin isoform X2 [Homo sapiens]	gi|530368805	28.870001	RSSTVDGLR	8.754726	7.892591
astrotactin-2 isoform a precursor [Homo sapiens]	gi|46488915	10	VRSNLYR	6.83268	9.165978
ataxin-7 [Homo sapiens]	gi|3192954	16.209999	VAKVPAMNNVHMK	8.481437	6.469744
ATF4 [Homo sapiens]	gi|47678299	7.4200001	MVAAK	6.305208	9.260595
atherin-like [Canis lupus familiaris]	gi|545544629	8.6400003	SLGSAAAPAAAATAAAAAGAEA	14.29117	10.32066
ATP synthase F0 subunit 6 (mitochondrion) [Homo sapiens]	gi|82493536	18.690001	QMIAMHNTKGR	10.8616	11.71459
ATP synthase mitochondrial F1 complex assembly factor 1, isoform CRA_b, partial [Homo sapiens]	gi|119627302	9.6599998	SAGFR	8.204309	4.886285
ATPase family AAA domain-containing protein 2B isoform X14 [Homo sapiens]	gi|578802954	18.889999	KTVDR	10.0097	9.836513
ATPase family AAA domain-containing protein 5 [Homo sapiens]	gi|26080431	17.290001	KISSPKKVVTSPR	5.868679	4.616278
ATPase, H+ transporting, lysosomal 56/58kD, V1 subunit B, isoform 2 variant, partial [Homo sapiens]	gi|62087192	15.29	MALRAMR	4.812133	3.772758
ATPase, H+ transporting, lysosomal V0 subunit a4 [Homo sapiens]	gi|80478359	17.84	AVERREMLESVNVR	5.414382	5.957548
ATP-binding cassette sub-family A member 10 [Homo sapiens]	gi|153792144	7.0799999	KLSGGQKR	7.644908	7.703842
ATP-binding cassette sub-family A member 13 [Homo sapiens]	gi|31657092	14.46	TLTGMGHSLEALR	6.632823	7.511977
ATP-binding cassette sub-family A member 3 isoform X2 [Canis lupus familiaris]	gi|545502578	33.369999	DMLQAEGQEPR	8.756118	8.620331
ATP-binding cassette sub-family A member 3-like isoform X1 [Canis lupus familiaris]	gi|545501933	10.92	ATWELLQQYK	3.787824	10.27644
ATP-binding cassette sub-family A member 9 isoform X4 [Canis lupus familiaris]	gi|545510050	7.5999999	QQLCAIAKVRFLKLK	7.889495	8.508564
ATP-binding cassette sub-family C member 11 [Canis lupus familiaris]	gi|359319049	11.52	GLTNSKSAAERFK	10.67801	9.285073
ATP-dependent DNA helicase Q1 isoform X6 [Canis lupus familiaris]	gi|545546127	12.22	LGIQAGAYHANMEPEDK	0	5.296707
ATP-dependent RNA helicase DDX18 [Homo sapiens]	gi|38327634	20.559999	GGGGGFGYQKTKK	7.391435	7.594455
ATP-dependent RNA helicase DDX27 isoform X1 [Homo sapiens]	gi|578836047	10.4	TVINFTMPNTIK	5.504877	7.755771
ATP-dependent RNA helicase DDX4 isoform 4 [Homo sapiens]	gi|262118318	7.4899998	DSWKSEAEGGESSDTQGPK	9.426166	10.74172
ATP-dependent RNA helicase DDX46 isoform X1 [Homo sapiens]	gi|530380277	10.56	EKDAGNFDQNK	10.35357	9.53688
ATP-dependent RNA helicase DDX50 isoform X2 [Homo sapiens]	gi|530394340	1.84	TGTGK	6.948859	7.926189
ATP-dependent RNA helicase DHX30 isoform X1 [Homo sapiens]	gi|578805801	11.74	RLMALAAGISPRLQPLGPRAAGI	11.32749	9.032647
ATP-dependent RNA helicase DHX33 isoform 2 [Homo sapiens]	gi|315113911	15.38	NMTLVAEVR	11.45029	9.777155
ATP-dependent RNA helicase DHX35 [Canis lupus familiaris]	gi|359322681	17.68	GLIRAATVR	6.828455	9.548372
ATP-dependent RNA helicase DHX8 isoform X2 [Homo sapiens]	gi|530411702	5.2800002	TGPGK	9.950905	8.088976
ATP-sensitive inward rectifier potassium channel 8 isoform X1 [Homo sapiens]	gi|530399119	11.17	RNNSMR	8.163165	7.85106
atrial natriuretic peptide receptor 2 isoform X2 [Homo sapiens]	gi|578816516	4.1500001	GAGSR	7.776986	6.703217
AT-rich interactive domain-containing protein 1B isoform X6 [Homo sapiens]	gi|578812873	22.469999	KPLDLFR	7.198959	9.470738
AT-rich interactive domain-containing protein 5A isoform X8 [Homo sapiens]	gi|578803517	18.450001	GTHGIMSPLAKKK	7.72594	7.782922

axin interactor, dorsalization-associated protein [Homo sapiens]	gi|124248539	12.32	LPSEPGMTLLTIR	9.485026	10.82977
B cell antibody heavy chain variable region, partial [Homo sapiens]	gi|224808480	19.790001	MAQVQLVQSGAXVK	4.864556	7.68319
B Chain B, Alix V Domain	gi|513137386	13.02	ADLVNRSIAQXR	9.217956	13.38231
B Chain B, Crystal Structure Analysis Of The Fgf10-fgfr2b Complex	gi|28949025	6.5500002	XEKRLHAVPAANTVKFR	0	6.88173
B Chain B, Crystal Structure Of A Ribonucleotide Reductase M2 B (rnrr2) From Homo Sapiens At 2.20 A Resolution	gi|386783388	8.9300003	KRGLXPGLTFSNELISR	9.300611	9.456874
B Chain B, Crystal Structure Of Bar Domain Of Endophilin-Iii	gi|188036176	26.66	LGXLNTVSKIR	8.96693	9.292788
B Chain B, Crystal Structure Of Human Sulfotransferase Sult1c1 In Complex With Pap	gi|161761239	14.65	GSLTSDLGKQIKLK	4.97347	6.814313
B Chain B, Crystal Structure Of Monomeric Variant Of Human Alpha-defensin 5, Hd5 (glu21eme Mutant)	gi|387766361	9.04	ESLSGVCXISGRLYR	0	6.802151
B Chain B, Crystal Structure Of Mutl Protein Homolog 1 Isoform 1 [homo Sapiens]	gi|330689714	11.71	KEXTAACTPR	7.347779	2.441354
B Chain B, Crystal Structure Of Phosphorylated Estrogen Receptor Beta Ligand Binding Domain	gi|312208029	10.1	NVVPVXDLLLEMLNAHVL	9.381882	8.96994
B Chain B, Crystal Structure Of The 3rd Bromodomain Of Human Poly-Bromodomain Containing Protein 1 (Pb1)	gi|261278906	22	ELARRDDIE	10.54292	10.57495
B Chain B, Crystal Structure Of The Cytoplasmic Domain Of The Human Chloride Channel Clc-ka	gi|150261380	8.1599998	PRILGRNIGSHHVR	12.64097	13.44772
B Chain B, Crystal Structure Of The Ubc Domain Of Baculoviral Iap Repeat- Containing Protein 6	gi|171849090	10.16	KAAXKPKPLSVLK	9.961697	8.678686
B Chain B, Design Of Non-covalent Inhibitors Of Human Cathepsin L. From The 96- Residue Proregion To Optimized Tripeptides	gi|27573940	17.969999	NQGQCGSXWAFSATGALEGQM	4.78891	6.668487
B Chain B, Discovery Of Checkpoint Kinase Inhibitor Azd7762 By Structure Based Design And Optimization Of Thiophene Carboxamide Ureas	gi|374073984	20.57	AVDCPENIKKEIXINK	11.18045	9.851636
B Chain B, High Resolution Structure Of Human Galectin-7	gi|358440052	10.17	FHVNLLXGEEQGSDAALHFNPR	8.430283	7.896791
B Chain B, N-Terminal 40kda Fragment Of Human Pms2	gi|17942781	11.52	LXFDHNGKIIQK	8.861346	9.847198
B Chain B, Pkr Kinase Domain-Eif2alpha Complex	gi|78101187	13.89	IGDFGLVTSLKNDGKRXR	8.340597	13.04407
B Chain B, Structural Basis For The Microtubule Binding Of The Human Kinetochore Ska Complex	gi|575403215	21.370001	SXNSVTR	4.24887	6.697711
B Chain B, Structure Of A Phosphoinositide 3-Kinase Alpha Adaptor- Binding Domain (Abd) In A Complex With The Ish2 Domain From P85 Alpha	gi|157836474	10.64	RXNSIKPDLIQLRK	6.908092	8.500538
B Chain B, Structure Of Cbp Nuclear Coactivator Binding Domain In Complex With P53 Tad	gi|310942529	13.59	PLSQETFSDLWK	7.875166	5.063734
B Chain B, Structure Of Human Placental S-adenosylhomocysteine Hydrolase: Determination Of A 30 Selenium Atom Substructure From Data At A Single Wavelength	gi|4929855	14.97	RATDVXIAGK	8.479804	9.282466
B Chain B, Structure Of The Catalytic Domain Of Usp7 (Hausp)	gi|28373981	12.27	GVKFLTLPPVLHLQLXR	7.869481	8.242063
B Chain B, Structure Of The Roc Domain From The Parkinson's Disease-associated Leucine-rich Repeat Kinase 2 Reveals A Dimeric Gtpase	gi|166007298	16.709999	TTLLQQLXKTKK	9.167204	7.659682
B Chain B, X-Ray Structure Of Homo Sapiens Protein Flj36880	gi|56553712	1.5700001	IPDPHK	0	4.751988
baculoviral IAP repeat-containing protein 2 isoform 2 [Homo sapiens]	gi|390608639	8.5600004	NTSPMR	9.931263	0
BAH and coiled-coil domain-containing protein 1 isoform X3 [Homo sapiens]	gi|578831174	14.55	GPGRPR	7.937927	9.626925
basic proline-rich protein-like [Canis lupus familiaris]	gi|545550731	21.059999	DEGQRRVPPPR	10.08625	10.39936
basic salivary proline-rich protein 1-like [Canis lupus familiaris]	gi|545550625	10.72	LGYLPSGRVLLPLPALPRR	7.343407	7.195932
Bcd orf2 [Homo sapiens]	gi|2745961	11.23	SAANPLTAPR	8.59677	8.461668
B-cell CLL/lymphoma 11A (zinc finger protein), isoform CRA_d [Homo sapiens]	gi|119620443	9.6899996	QEHIAGK	7.014758	6.874184
B-cell CLL/lymphoma 9 protein isoform X1 [Homo sapiens]	gi|578801354	19.719999	TDVGAPFGPQGHR	12.98336	9.918674
B-cell receptor-associated protein 29 isoform X1 [Homo sapiens]	gi|578814412	13.91	AAKKFMEENEK	10.24699	11.14413
B-cell receptor-associated protein 29, isoform CRA_e [Homo sapiens]	gi|119603818	1.41	MSGPP	6.203129	8.496685
BCL2-antagonist/killer 1, isoform CRA_b [Homo sapiens]	gi|119624146	15.91	MVTLPLQPSSTMGQVGR	7.394687	8.358406
bcl-2-like protein 10 isoform X1 [Homo sapiens]	gi|530405270	15.11	EQEGDVAR	8.605927	0
BCORY1 [Canis lupus familiaris]	gi|528889182	9.54	AKQGWTSKGLQPK	6.743138	5.143502
Bence Jones protein HAG=monoclonal IgM lambda VIII light chain {V region} [human, multiple myeloma, Peptide Partial, 117 aa]	gi|632814	24.889999	GDAIPDR	7.817796	8.994445
beta,beta-carotene 15,15'-monooxygenase isoform X1 [Canis lupus familiaris]	gi|57087275	8.04	MSTAYIRGVNWASCMAFHKED	10.37145	9.231381
beta-1-syntrophin isoform X2 [Homo sapiens]	gi|530389259	21.02	DALSSLPR	9.63873	9.191071
beta-galactosidase-1-like protein 3 isoform X1 [Homo sapiens]	gi|578821965	6.4499998	AAPGR	7.569816	6.36529
beta-tubulin cofactor D [Homo sapiens]	gi|3850044	16.08	LVXSLGGLTESTIR	4.002997	7.70427
bicaudal D-related protein 1 isoform X5 [Homo sapiens]	gi|578824469	11.8	VTSEDK	9.765937	8.257589
bifunctional heparan sulfate N-deacetylase/N-sulfotransferase 3 isoform X1 [Homo sapiens]	gi|578809641	19.02	LGLYTFVNLANFVK	3.943508	10.19387
bifunctional methylenetetrahydrofolate dehydrogenase/cyclohydrolase 2 [Canis lupus familiaris]	gi|545521903	15.11	ALAGGPALPLVLSAAAGRVSR	7.230744	5.583521
bifunctional methylenetetrahydrofolate dehydrogenase/cyclohydrolase 2 isoform X3 [Homo sapiens]	gi|530376985	2.8399999	TGIQTFGK	2.989244	5.327536
biogenesis of lysosome-related organelles complex 1 subunit 4 [Canis lupus familiaris]	gi|545493718	12.53	QVYSKIDR	8.498211	7.257259
biorientation of chromosomes in cell division protein 1-like 1 isoform X2 [Homo sapiens]	gi|578808374	4.0599999	KREVSPPGARTR	5.204606	0
blood vessel epicardial substance [Homo sapiens]	gi|22202606	13.62	LSILLKGKMK	6.828243	8.300937
BPI fold-containing family A member 2 isoform 1 [Canis lupus familiaris]	gi|73991578	82.800003	GLETVDNTLESVLQK	7.652531	7.337456
BPI fold-containing family A member 2 isoform X1 [Homo sapiens]	gi|530417837	53.139999	FVNSVINTLK	7.621317	13.0133
BPI fold-containing family B member 1 isoform X2 [Canis lupus familiaris]	gi|545540367	105.71	DHNAIHILQQLPLLSSIR	11.03131	9.049893
BPI fold-containing family B member 2 [Canis lupus familiaris]	gi|345789608	77.620003	LNEAVLSYVSEIGTAPLQQALR	5.805431	7.837678
BPI fold-containing family B member 2 precursor [Homo sapiens]	gi|15055535	36.639999	AGALNLDITGQLR	10.73926	11.55841
BPI fold-containing family C protein isoform X1 [Homo sapiens]	gi|530419838	13.61	VASTSVGLVILGQR	10.27109	9.237129
brain-specific angiogenesis inhibitor 1 isoform X1 [Homo sapiens]	gi|530389224	13.32	YAELDFEKIMHTR	8.089272	12.73201
Brca2 [Canis lupus familiaris]	gi|58801256	16.309999	EQDNSEITNFSHRGAKMSKDR	13.63074	10.97744
breast cancer resistance protein [Canis lupus familiaris]	gi|77812225	13.21	MSEQGRTIIFSIHQPR	10.59297	9.093201
bridging integrator protein-1 [Homo sapiens]	gi|2745974	10.52	GSKGVTAGK	10.48637	9.464052
BRO1 domain-containing protein BROX isoform X3 [Homo sapiens]	gi|578800564	26.43	CGEAIR	7.479988	9.170923
bromo adjacent homology domain-containing 1 protein isoform X5 [Canis lupus familiaris]	gi|545549430	6.1799998	VHGKNYPK	5.27137	7.35991

bromodomain adjacent to zinc finger domain protein 2A isoform X6 [Canis lupus familiaris]	gi|545513777	8.5500002	MLAMQR	7.717048	7.266065
bromodomain and PHD finger-containing protein 3 isoform X2 [Canis lupus familiaris]	gi|545518873	14.96	AETGRGWREALPCPLL	6.696355	6.507096
bromodomain-containing protein 2 isoform X4 [Canis lupus familiaris]	gi|545518263	27.860001	GSRAPRPSQPKK	7.544949	6.812913
bromodomain-containing protein 8 isoform X9 [Homo sapiens]	gi|578810105	15.75	RCAIEADMK	2.803405	7.654949
brorin [Canis lupus familiaris]	gi|345782937	13.46	RLAAER	7.339414	9.351688
BTB (POZ) domain containing 6, isoform CRA_a, partial [Homo sapiens]	gi|119602319	20.049999	MDSSPRPSPAMGAWEPAPDAGI	12.14345	4.528536
BTB/POZ domain-containing protein KCTD8 [Homo sapiens]	gi|38198663	11.57	GAAAAVPSGPGAHGGGGGGGA	9.299357	9.145194
BTF2p44, partial [Homo sapiens]	gi|736404	7.79	KXISS	8.688056	9.942257
BTG3 associated nuclear protein, isoform CRA_e [Homo sapiens]	gi|119615776	2.5999999	AAGASE	5.167519	5.390353
BUD13 homolog isoform 2 [Homo sapiens]	gi|229577273	11.64	RSQPPGK	9.154845	10.12001
C Chain C, Crystal Structure Analysis Of The N-Terminal Bromodomain Of Human Brd2	gi|110590692	16.25	QPXDXGTIK	12.82653	10.80072
C Chain C, Crystal Structure Of The Tyrosine Kinase Binding Domain Of Cbl-c (pl Mutant) In Complex With Phospho-egfr Peptide	gi|459358411	2.54	XSSDPT	1.409086	7.942654
C16orf46 protein [Homo sapiens]	gi|39795541	25.030001	SSPAACIWPRK	2.515088	9.970903
C1orf138 protein [Homo sapiens]	gi|124376654	14.97	GADTRRLPRETRPTK	9.050788	10.37152
C2 domain-containing protein 2 isoform X1 [Homo sapiens]	gi|530419214	9.6599998	GGILR	6.712339	9.782595
C2 domain-containing protein 3 [Canis lupus familiaris]	gi|545537153	11.82	KGQGSAGVR	9.567533	0
C4orf15 protein [Homo sapiens]	gi|19264159	23.799999	MASVTSHK	8.859967	10.05385
CA099_HUMAN RecName: Full=uncharacterized protein C1orf99	gi|125987734	28.139999	AMGQRTPSLAELR	7.505985	7.692084
cadherin-12 isoform 1 preproprotein [Homo sapiens]	gi|16445393	13.26	RNGYSR	2.88882	5.35944
cadherin-18 isoform X4 [Homo sapiens]	gi|578809828	15.63	DATMLK	12.73967	8.025025
cadherin-2 isoform X2 [Homo sapiens]	gi|530413612	12.16	ESAEVEEIVFPR	9.014256	8.726394
cadherin-23 isoform X5 [Homo sapiens]	gi|578819692	14.67	VATVK	8.662038	8.711028
cadherin-24 isoform 2 precursor [Homo sapiens]	gi|32880203	2.03	EPPAP	10.69576	8.678431
cadherin-like and PC-esterase domain-containing protein 1, partial [Canis lupus familiaris]	gi|545523262	9.79	GLPCSPAGPSWGRRLPSEQSCK	11.34952	7.974061
CAGH4 alternate open reading frame [Homo sapiens]	gi|2565087	12.99	CLSPLATSSQPK	9.378551	9.578793
calcineurin-binding protein cabin-1 isoform X11 [Homo sapiens]	gi|578837053	10.56	VSSMLQRTPDQGKK	4.702742	0
calcineurin-binding protein cabin-1 isoform X8 [Homo sapiens]	gi|578837050	10.02	SRPLPNMPK	9.885879	9.372572
calcium signal-modulating cyclophilin ligand [Homo sapiens]	gi|4502559	10.61	GDLTADSVQR	6.888025	8.371128
calcium/calmodulin-dependent protein kinase kinase 2 isoform X1 [Homo sapiens]	gi|530401173	11.26	HIPSLATVILVK	9.059504	9.498912
calcium-transporting ATPase type 2C member 1 isoform X1 [Homo sapiens]	gi|578807269	14.88	KRAIVK	10.09789	10.18144
calcyphosin-2 isoform X10 [Canis lupus familiaris]	gi|545514152	8.8599997	KKQTVAEQVMTDHLSR	8.166203	9.097977
calcyphosin-2 isoform X11 [Homo sapiens]	gi|578824354	12.85	KKQIVAEQVMIDHLSR	6.565572	10.84568
calcyphosin-like protein isoform X2 [Homo sapiens]	gi|578809847	6.0700002	EMAIQAK	0	7.154574
calmin, partial [Canis lupus familiaris]	gi|545508837	6.5500002	ASSTNDPSPESSILSSR	6.677991	6.22083
calmodulin-binding transcription activator 1 isoform 1 [Homo sapiens]	gi|54112401	11.28	TGGYGSHSEVQHNDVSEGK	8.326778	6.523028
calmodulin-binding transcription activator 1 isoform X2 [Homo sapiens]	gi|578798707	5.4899998	EPMSVLMMANR	7.054596	7.530743
calmodulin-binding transcription activator 2 isoform X7 [Homo sapiens]	gi|578829786	5.2800002	TSATLPAR	6.025597	6.518325
calmodulin-like protein 4 [Canis lupus familiaris]	gi|545550697	15.4	GYIMASELR	6.492814	5.537536
calpain-5 [Homo sapiens]	gi|37577157	10.84	GTPGPAVR	8.989006	8.484761
calpastatin isoform X15 [Canis lupus familiaris]	gi|545492664	5	TKDSKKPEDDK	12.19093	8.991482
calponin homology domain-containing protein 2 isoform X3 [Canis lupus familiaris]	gi|545557806	6.29	TGVSSPIK	8.167172	8.701215
calponin-3 isoform X1 [Homo sapiens]	gi|578798339	12.95	RFDEGK	9.569085	9.050504
cAMP-dependent protein kinase type I-alpha regulatory subunit isoform b [Homo sapiens]	gi|443497966	5.5100002	SIPLG	8.323268	6.750664
Can f 1 [Canis lupus familiaris]	gi|2598974	65.620003	DPEQSQEALEDFR	12.47588	8.043153
Can f 2 [Canis lupus familiaris]	gi|2598976	39.82	QQDFLPAFESVCEDIGLHK	11.64865	9.962847
canalicular multispecific organic anion transporter 1 isoform X1 [Homo sapiens]	gi|578818931	22.309999	RLDSVTR	10.66581	11.75423
cancer susceptibility candidate protein 1 [Canis lupus familiaris]	gi|545547118	26.459999	SWMIVEILK	8.955332	6.764091
carbonic anhydrase 4 isoform X1 [Homo sapiens]	gi|530412793	10.33	EQILAFSQKLYYDK	5.216007	12.01861
carbonic anhydrase 6 isoform 1 precursor [Homo sapiens]	gi|70167127	35.009998	NYPENTYYSNFISHLANIK	7.296103	7.907012
carbonic anhydrase VI [Canis lupus familiaris]	gi|28812184	62.639999	SYDIAQHEPDGLAVLAALVK	8.19398	5.811232
carbonyl reductase [NADPH] 2-like isoform X3 [Canis lupus familiaris]	gi|545509004	7.6799998	GAMTMLTKAMASELGPYQIR	11.97233	8.669456
carboxypeptidase B2 isoform X3 [Homo sapiens]	gi|530402101	17.99	AWTHCTEKFVVQKIAVGMK	4.294404	10.25378
carcinoembryonic antigen-related cell adhesion molecule 18 precursor [Homo sapiens]	gi|508083061	15.26	MDLSRPR	8.804919	0
cardiomyopathy-associated protein 5 [Homo sapiens]	gi|62241003	17.309999	TTSNTPPITGAIYK	7.753664	9.731107
carrier family 6, member 8 variant, partial [Homo sapiens]	gi|62088990	15.63	RMWHQALLRSGDKVR	0	6.927011
CASP8 and FADD-like apoptosis regulator isoform X3 [Homo sapiens]	gi|530371133	13.67	SAEVIHQVEEALDTDEKEMLLFI	8.030772	12.0072
CASP8-associated protein 2 [Homo sapiens]	gi|212549782	17.469999	SVDNSNRELLK	6.604506	8.579382
CASP8-associated protein 2 isoform X2 [Canis lupus familiaris]	gi|545519924	7.5100002	ADNGPNEAKNKDLKLSFMEK	10.77245	8.568154
caspase recruitment domain-containing protein 10 isoform X2 [Canis lupus familiaris]	gi|545514914	11.6	SLAIRVSCRSPPGGPEPHDK	5.246434	9.910437
caspase recruitment domain-containing protein 14 isoform 1 [Homo sapiens]	gi|332801087	13.29	TRGKNGAIAFLESLK	8.945422	10.91136
caspase-16 isoform X1 [Canis lupus familiaris]	gi|545502463	10.44	SRPGAK	9.596456	8.990417
cat eye syndrome critical region protein 2 isoform X3 [Homo sapiens]	gi|578836802	9.6700001	KLNGGLYCTK	8.694991	8.788247
catenin delta-1 isoform 1AC [Homo sapiens]	gi|332688210	14.13	RHVSAQLERVR	7.681004	10.87088
cathepsin B isoform X1 [Homo sapiens]	gi|578815061	8.6300001	SGVYQHVTGEMMGGHAIR	11.42915	10.49298
cathepsin S preproprotein [Canis lupus familiaris]	gi|27497538	11.24	KWLVGLLPLCSYAVAQVHK	5.506948	10.32385
cation channel sperm-associated protein subunit gamma isoform X1 [Homo sapiens]	gi|578834722	13.3	GSVVMR	11.75854	9.738206
cation-independent mannose-6-phosphate/insulin-like growth factor 2 receptor protein precursor [Canis lupus familiaris]	gi|63099710	22.57	GLRESADGAGGLVTGDRVR	6.621791	8.06037
caveolin 3, partial [Canis lupus familiaris]	gi|76161571	24.299999	VKDIHFKEIDLVNR	7.116682	3.65325
caveolin-2 isoform X1 [Canis lupus familiaris]	gi|545521927	10.73	ADVQLCMDDDAYSR	10.00846	9.275447
CCCTC-binding factor (zinc finger protein), isoform CRA_c [Homo sapiens]	gi|119603550	5.6799998	AGSPK	9.624306	6.620154
CCDC102B protein [Homo sapiens]	gi|33604112	12.81	MEGKRNAHSFGK	6.406119	7.169292
CCR4-NOT transcription complex subunit 3-like [Homo sapiens]	gi|530436103	10.79	APDARLALGSAR	6.231001	0
CCR4-NOT transcription complex subunit 6-like isoform X2 [Homo sapiens]	gi|530377448	9.8699999	IPPDIAK	5.624435	6.061656

CD2-associated protein [Canis lupus familiaris]	gi|345778489	18.309999	AKVESDDGK	9.362139	12.25291
CD2-associated protein isoform X1 [Homo sapiens]	gi|530381720	7.1100001	AVLSS	0	9.195009
CD93 antigen [Homo sapiens]	gi|119630576	14.18	MLAPSGSSGVWR	5.116819	9.896031
CDK5 regulatory subunit-associated protein 2 isoform X5 [Homo sapiens]	gi|578817654	11.86	AGEVPK	8.557753	8.987853
cell cycle and apoptosis regulator protein 2 isoform X2 [Canis lupus familiaris]	gi|545542486	11.62	NAKPGAAPTEHK	6.634878	7.732092
cell division cycle protein 27 homolog isoform X10 [Homo sapiens]	gi|578831763	4.1900001	AIVIDPK	4.606047	4.992421
centrin-2 [Homo sapiens]	gi|4757902	3.6199999	KTSLY	12.34131	8.659519
centriolin isoform X2 [Canis lupus familiaris]	gi|545518057	12.66	QAFKTAVKKAQLSEGK	12.2984	12.70379
centromere protein F [Homo sapiens]	gi|55770834	18.48	ELQLLNDK	10.61547	8.030972
centromere protein U [Canis lupus familiaris]	gi|345781796	10.03	LLDQKGDPTTKL	7.020434	5.950378
centromere protein X isoform X1 [Homo sapiens]	gi|530413460	17.940001	MEGAGAGSGFRK	8.45584	7.845284
centromere-associated protein E [Canis lupus familiaris]	gi|545552147	16.98	QQFMSTKEITETQEK	9.226456	6.535493
centromere-associated protein E isoform 2 [Homo sapiens]	gi|557878612	18.459999	QQNEQLIKQK	6.364053	8.060466
centrosomal protein of 104 kDa isoform X1 [Canis lupus familiaris]	gi|545499199	14.03	EGPSMEVESQDTQGGK	8.834773	5.734439
centrosomal protein of 120 kDa isoform X2 [Homo sapiens]	gi|530379724	8.4300003	LESATK	6.740823	4.927019
centrosomal protein of 164 kDa [Canis lupus familiaris]	gi|345799725	9.8400002	EEMEASEK	9.319437	11.04429
centrosomal protein of 170 kDa protein B [Canis lupus familiaris]	gi|545508888	12.38	SGVPAPARGTGGSSGPQR	8.124865	6.416887
centrosomal protein of 55 kDa [Canis lupus familiaris]	gi|359323204	14.99	TPTAALNESLVECPK	0	9.44721
centrosomal protein of 57 kDa isoform X2 [Canis lupus familiaris]	gi|545535894	9.71	TTPYVPSARRVK	5.576242	5.296749
centrosomal protein of 63 kDa isoform X12 [Homo sapiens]	gi|578807763	11.51	DITIASTKGSSSDMEKRLR	11.92948	9.111319
centrosomal protein of 63 kDa isoform X2 [Canis lupus familiaris]	gi|545539021	16.02	QALDSIEPMSR	10.17607	8.697241
centrosome and spindle pole-associated protein 1 isoform X18 [Homo sapiens]	gi|578815728	13.48	GNVFGEPPTELQIK	11.95924	10.13653
centrosome-associated protein 350 isoform X10 [Homo sapiens]	gi|578801826	10.37	AVTPPVKDDNEDVFSAR	3.005676	10.31315
centrosome-associated protein 350 isoform X2 [Canis lupus familiaris]	gi|545504467	10.62	EKEFCAGERNGYEPIK	8.852013	11.17983
cerebellin-1 precursor [Homo sapiens]	gi|4757922	24.85	EAASNGVLIQMEK	8.715956	12.47805
CF202_HUMAN RecName: Full=uncharacterized protein C6orf202	gi|190358756	21.75	MGGNRELVGK	7.972558	11.48382
cGMP-inhibited 3',5'-cyclic phosphodiesterase A isoform X1 [Canis lupus familiaris]	gi|545546155	14.79	RRRSSSVVSAEMSGCGSK	4.561929	10.59476
chaperonin 10, partial [Homo sapiens]	gi|4008131	3.8099999	DGXILGK	5.576605	8.03917
chaperonin containing TCP1, subunit 4 (delta), isoform CRA_a [Homo sapiens]	gi|119620390	12.47	NGKGDVTITNDGATILK	10.07986	11.86633
Chloride Channel protein 4, partial [Homo sapiens]	gi|3399663	6.4899998	SLLDAWSGWVVMLLIGLLA	2.108578	8.210997
cholesterol 7-alpha-monooxygenase [Homo sapiens]	gi|166295200	7.3299999	VLCHGK	7.898507	7.606776
choline-phosphate cytidylyltransferase A [Homo sapiens]	gi|31543385	14.74	YDAVQHCR	5.30055	9.484713
cholinephosphotransferase 1 isoform X1 [Canis lupus familiaris]	gi|545524646	14.07	MAAGAGARPGLRWPR	8.725546	8.786474
chondroitin sulfate synthase 1 isoform X2 [Homo sapiens]	gi|578826724	11.75	RVNPMYGAEYILDLLLLYK	7.229869	10.46286
chromatin assembly factor 1 subunit A [Canis lupus familiaris]	gi|545535181	9.8699999	GALAAAGAMLEELECGAPGAR(	8.952174	6.606016
chromatin target of PRMT1 protein isoform X1 [Homo sapiens]	gi|530364556	14.96	GGIGGR	8.035561	9.671812
chromodomain-helicase-DNA-binding protein 1 isoform X3 [Canis lupus familiaris]	gi|545492614	18.51	YSGSDSDSISERK	3.861288	9.134097
chromodomain-helicase-DNA-binding protein 3 isoform X11 [Homo sapiens]	gi|578829662	13.44	GGGNQVSLLNIMMDLKK	8.294891	6.234205
chromodomain-helicase-DNA-binding protein 8 isoform 1 [Homo sapiens]	gi|282165704	12.22	KADLDMDLLNSKNNLVIDTPR	8.613599	7.545957
chromodomain-helicase-DNA-binding protein 9 isoform X2 [Homo sapiens]	gi|578829235	4.0599999	AGGHK	9.197989	8.136305
chromosomal protein [Homo sapiens]	gi|537530	21.049999	YPLWLLIKR	8.145151	9.173075
Chromosome 1 open reading frame 141 [Homo sapiens]	gi|112180778	13.55	TNFILERNCEILK	12.1201	9.120502
chromosome 1 open reading frame 71, isoform CRA_a, partial [Homo sapiens]	gi|119597560	17.49	GWDIIGDEDVKR	4.797008	6.027929
chromosome 10 open reading frame 4, isoform CRA_f [Homo sapiens]	gi|119570446	17.110001	HGHGGYDSDFSDDEHCGESSKF	10.29146	6.751709
chromosome 11 open reading frame 11, isoform CRA_a, partial [Homo sapiens]	gi|119594365	8.04	RASLPGK	4.303953	5.201147
chromosome 11 open reading frame 63, isoform CRA_c [Homo sapiens]	gi|119587939	12.77	EESLPEISLLEILQNR	9.391163	8.804432
chromosome 11 open reading frame 71 [Homo sapiens]	gi|119587649	23.780001	MALNNVSLSSGDQR	4.143584	6.632763
Chromosome 16 open reading frame 78 [Homo sapiens]	gi|18204338	11.63	MSEQQMDLKDLMPTKRK	10.13032	6.780655
chromosome 2 open reading frame 28, isoform CRA_a [Homo sapiens]	gi|119621020	13.65	MRTFPVR	8.436295	8.19236
Chromosome 4 open reading frame 36 [Homo sapiens]	gi|16876936	20.610001	MAYGVPR	3.945106	11.28455
chromosome 6 open reading frame 149, isoform CRA_c, partial [Homo sapiens]	gi|119575582	6.4099998	RTRLFFFLSKMAASSR	8.099142	7.154061
chromosome 6 open reading frame 163, isoform CRA_b [Homo sapiens]	gi|119568991	17.559999	KKTPSNLVIK	8.911407	3.717367
chromosome 7 open reading frame 13 [Homo sapiens]	gi|119624955	10.55	AAGVFGDRLAGVFGSR	12.23309	11.1701
chromosome 8 open reading frame 49, partial [Homo sapiens]	gi|119586026	2.46	GCGEPR	3.952089	6.330807
chromosome 9 open reading frame 19, isoform CRA_a, partial [Homo sapiens]	gi|119578720	10.64	GASGRVR	8.488956	7.823723
chromosome 9 open reading frame 84, isoform CRA_b [Homo sapiens]	gi|119579493	13.39	ASDNIIMR	8.424599	9.701342
chromosome transmission fidelity protein 8 homolog isoform X1 [Canis lupus familiaris]	gi|545500117	8.29	ERPTPWTRAQLPPRGR	12.58584	12.91516
chromosome X open reading frame 43, isoform CRA_c [Homo sapiens]	gi|119618980	15.55	MTSLGSVCR	7.887839	10.84884
CHST13 protein [Homo sapiens]	gi|74355600	18.43	LASAYRNKLARTR	8.841577	10.91425
CHTF18 protein, partial [Homo sapiens]	gi|13623635	3.4400001	AGGSER	4.964489	6.116938
cilia- and flagella-associated protein 44 isoform X9 [Homo sapiens]	gi|578807583	14.79	DMKAKVPSPR	12.3119	9.635426
cingulin isoform X2 [Homo sapiens]	gi|578801321	19.040001	ATIYGILR	8.776669	8.170703
cis-aconitate decarboxylase [Homo sapiens]	gi|385719224	15.59	ANASKMLSWDTVESLIK	7.769724	11.14892
citron Rho-interacting kinase isoform X4 [Homo sapiens]	gi|578823215	9.0100002	SAREEAAHRK	9.189464	10.62183
CKAP4 protein, partial [Homo sapiens]	gi|45501009	4.1799998	RVPTRR	8.853655	9.079124
clarin-2 [Homo sapiens]	gi|150010645	9.2200003	MPGWFKK	6.627691	10.22231
clathrin interactor 1 isoform 1 [Homo sapiens]	gi|307078123	10.11	ASPDQNASTHTPQSSVK	8.054523	5.313434
cleavage stimulation factor subunit 2 tau variant [Homo sapiens]	gi|14149675	14.56	MSSLAVR	0.8441408	7.042975
CLIP-associating protein 2 isoform X15 [Canis lupus familiaris]	gi|545538318	18.1	MDGATSEDGRVRAK	9.732797	8.293219
clustered mitochondria protein homolog isoform X1 [Canis lupus familiaris]	gi|545512203	7.27	QGVCALPR	6.684607	8.729049
C-mannosyltransferase DPY19L2 isoform X5 [Homo sapiens]	gi|578823614	8.5100002	SALGGGK	5.771521	7.167907
coagulation factor VIII isoform X1 [Canis lupus familiaris]	gi|545557151	18.129999	EGSLAKER	7.370575	0
cohesin subunit SA-3 isoform X1 [Canis lupus familiaris]	gi|545500886	12.11	QASEGHPPVGRITGR	9.473022	0
coiled-coil alpha-helical rod protein 1 isoform X9 [Homo sapiens]	gi|578842091	0.2	AGEAKELAEAQREAELLRK	4.698339	6.138115
coiled-coil and C2 domain-containing protein 2A [Canis lupus familiaris]	gi|545547489	11.02	ALTDATKLANENPEIR	9.16828	9.744849

coiled-coil and C2 domain-containing protein 2A isoform X1 [Homo sapiens]	gi|530376338	11.32	QCLTTVIDISGKTVFITR	4.103702	7.242237
Coiled-coil domain containing 13 [Homo sapiens]	gi|23273923	13.43	LLSSEMKTLKSQMGTLVVK	12.42335	8.78922
coiled-coil domain-containing protein 108 [Canis lupus familiaris]	gi|545556260	7.1199999	MLIRAAPSPPMKTR	3.187279	6.300232
coiled-coil domain-containing protein 113 isoform 1 [Homo sapiens]	gi|19923432	8.3299999	TGMDR	9.405934	9.122118
coiled-coil domain-containing protein 13 isoform X6 [Homo sapiens]	gi|530371957	12.9	QELRMAQKVLAR	8.263304	12.84149
coiled-coil domain-containing protein 132 isoform X2 [Homo sapiens]	gi|530386202	15.4	EGFTQASLGLLANQRK	6.809356	9.453078
coiled-coil domain-containing protein 136 isoform X13 [Homo sapiens]	gi|578814522	13.98	GGSVGSLSVNK	8.085492	0
coiled-coil domain-containing protein 141 isoform X1 [Homo sapiens]	gi|578804115	23.219999	SLALLNK	4.222295	3.865073
coiled-coil domain-containing protein 149 isoform X2 [Homo sapiens]	gi|578808444	12.32	ADPKDGEAQK	5.202325	8.635422
coiled-coil domain-containing protein 166 [Homo sapiens]	gi|242332545	17.26	EHEDTRDLAR	7.743993	4.125165
coiled-coil domain-containing protein 168 [Canis lupus familiaris]	gi|545537937	9.3999996	VQTGKGK	6.984772	6.664341
coiled-coil domain-containing protein 168 [Homo sapiens]	gi|226246554	18.290001	MSEIDLVAAK	4.664475	7.191905
coiled-coil domain-containing protein 29-like isoform X1 [Homo sapiens]	gi|578844584	4.2199998	DNMASLNK	5.378469	5.063855
coiled-coil domain-containing protein 37 isoform X1 [Homo sapiens]	gi|578807364	17.9	NVEPENMSGYIK	9.881647	8.850444
coiled-coil domain-containing protein 47 isoform X1 [Homo sapiens]	gi|530412564	14.64	AEKERIMNEEDPEK	11.28872	11.17262
coiled-coil domain-containing protein 60 isoform X3 [Homo sapiens]	gi|578823384	7.6700001	KAPSILSVLKQNK	7.248096	8.130625
coiled-coil domain-containing protein 68 isoform X1 [Canis lupus familiaris]	gi|545485253	7.71	TVGTTPYLMLIRLR	1.230751	5.810845
coiled-coil domain-containing protein 71L [Homo sapiens]	gi|57242766	7.8899999	GGDFR	6.800315	9.016541
coiled-coil domain-containing protein 74B isoform X9 [Homo sapiens]	gi|578805131	22.290001	KPRISPR	7.571958	10.48377
coiled-coil domain-containing protein 89 [Canis lupus familiaris]	gi|57102318	14.73	QAVEQMAEAER	9.869246	12.78559
coiled-coil domain-containing protein KIAA1407 homolog isoform X3 [Canis lupus familiaris]	gi|545552897	3.3399999	AESIR	7.95822	6.179071
coiled-coil domain-containing protein R3HCC1L isoform X1 [Canis lupus familiaris]	gi|73998557	22.709999	KEDGFFQKEIFRDK	5.83853	9.197758
collagen alpha-1(I) chain-like [Canis lupus familiaris]	gi|545489812	8.96	GAGGTAGQQR	4.479076	0
collagen alpha-1(I) chain-like [Canis lupus familiaris]	gi|545560531	13.12	SSPRPAAADTAKR	7.024112	5.618656
collagen alpha-1(III) chain-like isoform X1 [Homo sapiens]	gi|530433770	21.049999	AGEQAVGR	7.941789	6.903253
collagen alpha-1(III) chain-like, partial [Homo sapiens]	gi|530359484	14.56	GPGAELLAGPGGADEAVGSR	5.986816	10.28463
collagen alpha-1(VI) chain isoform X1 [Homo sapiens]	gi|578836441	12.1	GEKGKRGIDGVDGVK	11.56765	11.62033
collagen alpha-1(VII) chain precursor [Homo sapiens]	gi|4502961	8.5799999	EAQASGLNVVMLGMAGADPEQ	9.632802	8.037514
collagen alpha-1(XIII) chain [Canis lupus familiaris]	gi|545495125	12.52	GERGSKGDLGMTGPTGAAGLPG	8.771992	6.517957
collagen alpha-1(XXIII) chain isoform X7 [Homo sapiens]	gi|578811280	10.2	SATTAGSR	7.618797	7.888635
collagen alpha-1(XXVII) chain [Canis lupus familiaris]	gi|545518161	6.6599998	IAPPATLMPPTLAPGSAPTR	7.681376	9.470693
collagen alpha-2(I) chain precursor [Homo sapiens]	gi|48762934	8.4399996	GLHGEFGLPGPAGPR	12.75137	10.51796
collagen alpha-2(I) chain-like [Canis lupus familiaris]	gi|545493065	11.14	ELPAAPHVALVGPAGLR	5.563254	6.475308
colorectal cancer associated two [Homo sapiens]	gi|558026281	7.4000001	GTRESGQK	6.872499	9.051601
COMM domain-containing protein 6 [Canis lupus familiaris]	gi|545537677	16.129999	LGMAVSSDSCR	8.639467	6.316838
complement C4-A [Canis lupus familiaris]	gi|545520262	15.88	GRIVSMNREPR	9.222086	12.49913
complement decay-accelerating factor isoform X1 [Homo sapiens]	gi|530366087	14.41	FIFCR	4.928445	5.233677
conserved nuclear protein NHN1, isoform CRA_g [Homo sapiens]	gi|119587209	15.6	GQTSFSWIQNTAVKPEGK	9.646363	10.3799
contactin-3 isoform X1 [Canis lupus familiaris]	gi|545532933	12.19	ISFLKDGGLK	8.675612	8.817979
copper-transporting ATPase 2 isoform X13 [Homo sapiens]	gi|578824929	5.3800001	HSAAADDDGDKWSLLLNGRDE	8.331319	7.150856
copper-transporting ATPase beta subunit, partial [Canis lupus familiaris]	gi|47680380	6.3499999	QEAALALHMLK	3.694421	7.57499
cornulin [Canis lupus familiaris]	gi|345782802	7.8000002	TQTDVTHTVTQTEVTQVMEQD	0	8.452077
coronin 6, isoform CRA_d [Homo sapiens]	gi|119571592	1.48	GGAHK	4.111305	0
cortactin-binding protein 2 [Canis lupus familiaris]	gi|545523256	13.55	VDNKTLASPPSSGPQGNR	9.293578	8.9663
COX assembly mitochondrial protein 2 homolog isoform X3 [Homo sapiens]	gi|530424132	11.02	EHGIAMR	8.355838	10.10165
creatine kinase M-type isoform X1 [Homo sapiens]	gi|530415665	20.68	VGMVREQGGER	7.25028	4.575381
crystallin, lambda 1, isoform CRA_d [Homo sapiens]	gi|119628668	9.4700003	EGVGEKEGIVSPSDLDLVMSEGI	9.474263	5.664724
CTP synthase 1 isoform X1 [Homo sapiens]	gi|578798479	7.1599998	EMADR	6.787468	11.04497
CUB and sushi domain-containing protein 2, partial [Canis lupus familiaris]	gi|545525026	13	TVGLSSCPEPTVPSNGVK	9.371106	8.949826
CUGBP, Elav-like family member 1 isoform X3 [Canis lupus familiaris]	gi|545530117	7.3200002	MTTSTCAAKVIKEAKAVCICGSI	8.379458	8.008978
cullin-9 isoform X1 [Canis lupus familiaris]	gi|545519208	14.8	VGLQSPPLEAR	9.309792	9.273358
cullin-9 isoform X4 [Homo sapiens]	gi|578811522	22.43	APDFVPR	8.486052	7.093642
C-X-C motif chemokine 14 [Canis lupus familiaris]	gi|545516866	13.3	LLPAALLLLLLALCAAR	4.955002	7.599732
cyclic AMP-dependent transcription factor ATF-6 alpha isoform X2 [Homo sapiens]	gi|578800689	9.5299997	KEYMLGLEAR	5.536813	7.821817
cyclin N-terminal domain-containing protein 2 isoform X1 [Homo sapiens]	gi|578834929	19.709999	AVTPEMR	7.385742	10.38677
cyclin-D-binding Myb-like transcription factor 1 isoform X12 [Homo sapiens]	gi|578814836	10.09	DVEDLVNCH	11.66564	8.950159
cyclin-dependent kinase 4 inhibitor C [Homo sapiens]	gi|4502751	7.9099998	TALQVMKLGNPEIARR	9.72208	7.554085
cyclin-dependent kinase inhibitor 1 [Canis lupus familiaris]	gi|545518904	8.1700001	GGRDDLGGGK	7.006785	0
cyclin-dependent kinase-like 4 isoform X1 [Homo sapiens]	gi|578802866	2.0599999	RKARNEGR	2.828118	2.773466
cyclin-K [Canis lupus familiaris]	gi|545508848	0.9	SLQSMMK	5.97546	4.201636
cystatin-S precursor [Homo sapiens]	gi|4503109	86.730003	IIPGGIYDADLNDEWVQR	5.325364	9.120594
cystatin-SA precursor [Homo sapiens]	gi|4503105	32.310001	IIEGGIYDADLNDER	7.445038	9.390382
cystatin-SN precursor [Homo sapiens]	gi|19882251	19.360001	QLCSFEIYEVPWENRR	4.768847	9.544207
cysteine/serine-rich nuclear protein 3 isoform X1 [Homo sapiens]	gi|530370984	16.24	EGCSNTAGR	5.538695	3.152854
cysteine-rich motor neuron 1 protein isoform X3 [Canis lupus familiaris]	gi|545527556	0.44	AHGDR	6.521739	10.82261
Cysteinyl-tRNA synthetase 2, mitochondrial (putative) [Homo sapiens]	gi|13938199	10.37	SSTTSTWELLDPRTK	7.355276	10.09903
cytochrome b ascorbate-dependent protein 3 isoform 2 [Homo sapiens]	gi|241896864	11.43	QPLLHDGE	7.937069	9.793935
cytochrome b561 domain-containing protein 1 isoform X3 [Homo sapiens]	gi|530362436	8.5100002	AARVSR	10.87969	8.028708
cytochrome b5-like [Canis lupus familiaris]	gi|545529104	10.77	MAINK	9.131281	8.753942
cytochrome c oxidase assembly factor 6 homolog isoform X1 [Homo sapiens]	gi|530366186	9.7299995	QVCWGAR	8.767046	8.558268
cytochrome P450 4Z1 [Homo sapiens]	gi|30023836	11.85	QDTTQK	9.327334	9.578113
cytochrome P-450 IIB [Canis lupus familiaris]	gi|164040	1.78	EIDQVIGPHRLPSLDDR	6.915607	7.534438
cytokine-like protein 1 [Canis lupus familiaris]	gi|73951856	12.82	VAQVDGLKDKVRK	14.33952	10.22799
cytoplasmic dynein 1 heavy chain 1 [Canis lupus familiaris]	gi|73964009	23.74	LRQNLDGLLNQLK	12.69506	9.457417
cytoplasmic dynein 1 light intermediate chain 1 isoform X2 [Canis lupus familiaris]	gi|545538648	10.6	SGLRLHK	7.184484	8.78167

cytoplasmic dynein 2 heavy chain 1 isoform X1 [Homo sapiens]	gi|578822370	19.76	GLGGNLNMK	5.775009	7.578894
cytoskeleton-associated protein 2 isoform X1 [Homo sapiens]	gi|530402273	7.1199999	EEVKEVSIEDTGVDVDPEK	7.386266	4.357527
cytoskeleton-associated protein 5 isoform X5 [Canis lupus familiaris]	gi|545530217	4.54	SMSGHPEAAQIVRR	12.3659	11.66695
cytosolic carboxypeptidase 1 isoform X3 [Homo sapiens]	gi|530390827	15.26	VALDTLAALLK	8.104695	11.84958
cytosolic carboxypeptidase 3 isoform X2 [Canis lupus familiaris]	gi|545525526	9.8100004	ESFPSVWYTRNMIRRLMEK	6.48517	12.97707
cytosolic phospholipase A2 zeta isoform X2 [Homo sapiens]	gi|530405620	6.9099998	ERAGA	7.329871	10.50539
D Chain D, Crystal Structure Of A Hsp47-Collagen Complex	gi|400977338	16.09	XLSPKAATLAER	6.49271	4.499572
D Chain D, Crystal Structure Of Dog (Canis Familiaris) Hemoglobin	gi|227343819	31.299999	LLIVYPWTQR	4.296393	7.099972
D Chain D, Crystal Structure Of Kh1 Domain Of Human Pcbp2 Complexed To Single-Stranded 12-Mer Telomeric Dna	gi|149243359	10.74	LLXHGK	6.664092	6.896702
D Chain D, Crystal Structure Of Moz Double Phd Finger Histone H3k14ac Complex	gi|550544618	13.59	QTARKSTGGXAPR	10.95614	10.94602
D Chain D, Crystal Structure Of The Endophilin Bar Domain Mutant	gi|112490401	12.46	GPLGSMSVAGLK	11.54538	12.80842
D Chain D, Crystal Structure Of The R482w Mutant Of Lamin A/c	gi|255311807	15.17	AQNTWGXGNSLR	8.528456	11.70931
D Chain D, Human Mhf1-mhf2 Complex	gi|568786800	19.809999	TKVSGDALQLXVELLK	6.624675	5.048157
D Chain D, Structural Insights Into Nedd8 Activation Of Cullin-Ring Ligases: Conformational Control Of Conjugation	gi|208435635	29.84	EVLKKLKYVQNK	9.235144	5.130898
D Chain D, The Structure Of The Human Retinoid-X-Receptor Beta Ligand Binding Domain In Complex With The Specific Synthetic Agonist Lg100268	gi|20663786	9.1000004	MPVDR	9.268201	8.633523
DALR anticodon-binding domain-containing protein 3 isoform X3 [Homo sapiens]	gi|578806276	5.8800001	GTKSGTFVMYNCARLATLFESY	7.030166	4.057784
DBH-like monooxygenase protein 2 [Canis lupus familiaris]	gi|73978818	9.9899998	VEPQDKTPAGFR	3.129153	9.146045
DDB1- and CUL4-associated factor 15 [Homo sapiens]	gi|78486540	8.1400003	SERNSGAGSGGGGPGGAGGKR/	10.68903	11.47019
dead end protein homolog 1 [Homo sapiens]	gi|34740339	6.02	AAAMAK	4.5593	6.476393
death-associated protein kinase 1 isoform X4 [Homo sapiens]	gi|578817135	6.1999998	ASAVNMEK	9.893573	6.786979
death-inducer obliterator 1 isoform c [Homo sapiens]	gi|301129165	13.37	ASEDRR	4.988039	6.163579
dedicator of cytokinesis protein 10 isoform X10 [Homo sapiens]	gi|578804561	5.8099999	ANHSVAR	5.58928	0
dehydrogenase/reductase SDR family member 11 isoform X3 [Homo sapiens]	gi|578840409	7.6900001	DMFNGLSSLPHKPTLPRR	9.245209	8.073337
dehydrogenase/reductase SDR family member 7B isoform X1 [Homo sapiens]	gi|578829832	10.35	MSIPFRSAYAASKHATQAFFDCI	10.45958	7.418282
deleted in malignant brain tumors 1 protein isoform X3 [Canis lupus familiaris]	gi|545548198	38.709999	DQTYQSYSQPSPR	9.596298	10.10777
delta and Notch-like epidermal growth factor-related receptor isoform X1 [Homo sapiens]	gi|530371157	19.27	HARFGKK	0	4.845905
delta-like protein 3 isoform 1 precursor [Homo sapiens]	gi|8393264	18.42	AGFAGPR	8.675505	8.868502
delta-type opioid receptor [Canis lupus familiaris]	gi|345793999	9.1000004	AGGLEGR	9.93707	11.40929
dendrin [Homo sapiens]	gi|194272140	15.16	TQQVQLLPSGVTR	8.382022	9.143773
dendrin isoform X3 [Canis lupus familiaris]	gi|545545643	12.36	AGAGPSEGGKGTR	7.797525	10.03622
DENN domain-containing protein 1A isoform X3 [Canis lupus familiaris]	gi|545513419	0.72	SLALPR	8.179542	9.021776
DENN domain-containing protein 3 isoform X10 [Homo sapiens]	gi|530388869	27.16	KSLQIVLPAR	8.957999	9.340576
dermatan-sulfate epimerase isoform X3 [Homo sapiens]	gi|530383641	8.8699999	FGQAR	4.258485	6.111624
desmoplakin [Canis lupus familiaris]	gi|345796694	10.14	KKYPCDKNMPLQR	9.627234	7.299919
development and differentiation enhancing factor 2, isoform CRA_b [Homo sapiens]	gi|119621408	22.209999	NTVAAIEEVR	3.170012	5.577309
developmentally regulated GTP binding protein 2, isoform CRA_b [Homo sapiens]	gi|119576074	20.370001	MMLDATK	10.42949	11.78848
dexamethasone-induced Ras-related protein 1 isoform X1 [Canis lupus familiaris]	gi|73956260	12.37	EKASGGGQAK	7.030478	0
diacylglycerol kinase gamma isoform X5 [Canis lupus familiaris]	gi|545553741	3.4000001	RVVTDPK	7.238596	7.445929
differential display clone 8 isoform X2 [Homo sapiens]	gi|578830238	14.2	CSGWSSSVIWR	8.435204	11.63693
diffuse panbronchiolitis critical region protein 1 precursor [Homo sapiens]	gi|238624188	14.94	TTPFPAEPTENR	9.07327	8.366692
dipeptidase 2 isoform X1 [Canis lupus familiaris]	gi|73957466	6.3899999	GQTSLDR	7.735294	7.924061
disabled homolog 2-interacting protein isoform X5 [Canis lupus familiaris]	gi|545513497	18.4	GLCGLGLLGDNLGK	13.91561	9.783192
disco-interacting protein 2 homolog B isoform X7 [Homo sapiens]	gi|578824032	11.65	QKQPGVGPASVMVGNLVAGKR	7.060447	7.950724
disco-interacting protein 2 homolog C isoform X8 [Homo sapiens]	gi|530392114	11.21	MIVEVSR	8.708969	9.344778
disintegrin and metalloproteinase domain-containing protein 8 [Canis lupus familiaris]	gi|545548488	8.96	VSAAFRPR	10.33356	0
disks large homolog 2 isoform X21 [Canis lupus familiaris]	gi|545536161	13.13	RVTLEGDSEEMGVIPSKR	9.480341	7.559361
disrupted in schizophrenia 1 protein isoform X1 [Canis lupus familiaris]	gi|345798836	19.84	EITAKVCMSER	9.077267	10.43649
dJ549K18.1 [Homo sapiens]	gi|47678483	17.07	ICNLLPIR	7.220173	7.785599
DKFZP564B147, partial [Homo sapiens]	gi|49457450	13.42	SLSPSARPISSPSPETSCVPAT	4.177337	5.211219
DLG1 protein, partial [Homo sapiens]	gi|33870101	18.799999	KPVSEKK	7.546687	10.59956
DNA (cytosine-5)-methyltransferase 3-like isoform 2 [Homo sapiens]	gi|28872780	6.9299998	ESENPLEMFETVPVWR	2.6566	4.454254
DNA damage-induced apoptosis suppressor protein [Homo sapiens]	gi|194239694	11.35	IILVSK	3.916139	0
DNA excision repair protein ERCC-6 isoform X2 [Canis lupus familiaris]	gi|545547251	4.0300002	KITAKQKR	7.569295	3.535235
DNA helicase B isoform X1 [Homo sapiens]	gi|530401009	12.78	TLLQENNLQNAK	12.02927	7.254467
DNA helicase INO80 [Homo sapiens]	gi|38708321	9.5	KRDMGHDGIQEEILR	7.70102	8.450913
DNA ligase 1 isoform X1 [Homo sapiens]	gi|578834471	5.73	LRMVETLSNLLR	2.293926	8.172288
DNA ligase 4 isoform X2 [Canis lupus familiaris]	gi|545538037	8.0600004	ATEIVSSDMYKTGCTLR	7.943816	6.916582
DNA mismatch repair protein Mlh1 isoform X2 [Homo sapiens]	gi|530372510	20.57	KEDLDIVCER	8.933502	7.49466
DNA mismatch repair protein Msh3 [Homo sapiens]	gi|284813531	12.33	MSRRKPASGGLAASSSAPAR	8.375558	8.331661
DNA polymerase delta subunit 2 [Canis lupus familiaris]	gi|545560308	11.13	SLACQPISFSGFGAEDEDLGGLG	6.45081	5.891539
DNA polymerase eta isoform X1 [Canis lupus familiaris]	gi|73973002	12.18	EASVEVMGILSR	7.669682	8.750641
DNA repair protein RAD50 [Homo sapiens]	gi|19924129	8.4099998	ILELDQELIKAERELSKAEK	0	7.983154
DNA replication licensing factor MCM3 isoform 2 [Homo sapiens]	gi|394582099	15.08	KKEKMVSAAFMK	8.08499	6.230598
DNA topoisomerase 3-alpha [Homo sapiens]	gi|10835218	16.35	AAMEMALR	12.4934	9.404268
dnaJ homolog subfamily B member 8 [Canis lupus familiaris]	gi|73984943	15.47	SVMSSTEMVNGHK	7.756338	8.374102
dnaJ homolog subfamily C member 13 isoform X1 [Homo sapiens]	gi|530374184	15.42	IAMDQYGKFNK	9.714116	8.667128
dnaJ homolog subfamily C member 14 [Homo sapiens]	gi|119943096	12.26	VSSGKKPPSRR	9.018605	8.418791
dorsal root ganglia homeobox protein [Homo sapiens]	gi|449784876	21.469999	KTERGASDQEPGAK	8.743899	10.54215
double C2-like domain-containing protein alpha isoform X1 [Canis lupus familiaris]	gi|545501644	9.6400003	KDDTGTIQSQGCCMRGR	7.019424	5.875888
double-stranded RNA-binding protein Staufen homolog 2 isoform f [Homo sapiens]	gi|256419005	9	SNVNNNPGSITPTVELNGLAMK	9.079091	10.81772
DRB1 transplantation antigen, partial [Homo sapiens]	gi|181803	17.17	AAVDTY	9.360019	9.251067
DRIL3 [Homo sapiens]	gi|56799575	13.55	QQQHQHVGGDQR	8.492433	7.910002

D-tyrosyl-tRNA(Tyr) deacylase 1 isoform X2 [Homo sapiens]	gi|530426043	7.6199999	KILNLRVFEDESGK	7.300695	6.078868
dual 3',5'-cyclic-AMP and -GMP phosphodiesterase 11A isoform 3 [Homo sapiens]	gi|116536087	15.31	TKSLLCMPIR	8.487362	5.043788
dual specificity mitogen-activated protein kinase kinase 2 isoform X1 [Homo sapiens]	gi|578833393	14.21	RIPEEILGK	10.61303	11.47527
dual specificity phosphatase 5 variant, partial [Homo sapiens]	gi|62089232	15.39	LLQEGGGGVAAVVVLDQGSRH	5.852589	6.438248
dual specificity protein kinase TTK isoform X2 [Canis lupus familiaris]	gi|545519728	5.5999999	EAEDVGGR	2.857919	3.731814
dual specificity protein phosphatase 5 [Homo sapiens]	gi|62865890	7.6500001	VVLTSLLACLPAGPRVYFLK	10.27413	10.6603
dual specificity tyrosine-phosphorylation-regulated kinase 4 isoform X4 [Canis lupus familiaris]	gi|545546678	14.97	ILEALRRK	9.639788	8.470952
dual-specificity tyrosine-(Y)-phosphorylation regulated kinase 4, isoform CRA_b [Homo sapiens]	gi|119609248	15.65	QKFTSAKGPTLSEIYMVGSK	0	5.64156
dynamin-like 120 kDa protein, mitochondrial isoform 7 [Homo sapiens]	gi|224831251	16	DFFTSGSPEETAFR	9.285388	7.373882
dynein heavy chain 14, axonemal [Canis lupus familiaris]	gi|545506135	6.9699998	QEAFLEDLNNVLNMR	6.346325	8.539474
dynein heavy chain 14, axonemal isoform X4 [Homo sapiens]	gi|578802138	14.87	ETETLMEK	7.15554	7.35544
dynein heavy chain 17, axonemal [Canis lupus familiaris]	gi|545509233	1.4400001	ALMEEAVK	0	4.646714
dynein heavy chain 3, axonemal isoform X2 [Canis lupus familiaris]	gi|545501980	13.06	QHIPILSISCNPGMK	8.108199	8.591034
dynein heavy chain 5, axonemal isoform X1 [Homo sapiens]	gi|530378724	17.9	HGMMTLGPSGAGKTTCIHTLMI	7.438893	9.888762
dynein heavy chain 6, axonemal isoform X1 [Homo sapiens]	gi|578802753	12.79	AAQVIR	0	7.879261
dynein heavy chain 9, axonemal [Canis lupus familiaris]	gi|545498900	22.52	YEDMLSLLEKYEAR	10.55682	6.219501
dynein intermediate chain 1, axonemal isoform X7 [Canis lupus familiaris]	gi|545517363	12.49	FNFSER	8.873829	9.033104
dystonin isoform X17 [Homo sapiens]	gi|530382450	12.79	SHEQLLQNIKAEK	8.643973	6.764445
dystonin isoform X18 [Homo sapiens]	gi|578811869	3.0899999	STIANLMGK	7.56803	7.492119
dystrobrevin beta isoform X7 [Homo sapiens]	gi|578802763	8.54	HGPALYT	0	7.490173
dystrotelin [Canis lupus familiaris]	gi|345797457	14.26	QWLLDXVTPK	1.726564	4.167612
E Chain E, Co-Crystal Structure Of The Sam Domains Of Epha1 And Epha2	gi|269914625	14.35	IAYSLLGLKDQVNT	2.594691	9.597116
E Chain E, Crystal Structure Of Protein Phosphatase 2a (Pp2a) With C-Terminus Truncated Catalytic Subunit	gi|122921206	7.0100002	EKLKXKE	8.039454	8.798056
E Chain E, Crystal Structure Of Trim24 Phd-Bromo Complexed With H3(23-31)k27ac Peptide	gi|315113510	6.6399999	XSAPA	10.22663	10.87495
E3 SUMO-protein ligase EGR2 isoform X2 [Canis lupus familiaris]	gi|545494995	13.56	KPFPCPLDSLR	7.348171	6.125963
E3 SUMO-protein ligase RNF212 isoform b [Homo sapiens]	gi|37059810	5.0100002	RLSSLAAPPSVQFWK	0	7.533463
E3 ubiquitin-protein ligase BRE1A [Homo sapiens]	gi|34878777	9.0200005	AHLDEAR	10.51074	6.365615
E3 ubiquitin-protein ligase BRE1A isoform X2 [Canis lupus familiaris]	gi|545517783	19.42	TAVEDSGTTVETIKLGGVSSTEE	12.46649	7.112717
E3 ubiquitin-protein ligase BRE1B isoform 3 [Homo sapiens]	gi|333440442	13.82	AAGDGGSGPPEKKLSR	11.88445	9.563039
E3 ubiquitin-protein ligase CBL-C isoform X2 [Homo sapiens]	gi|530416089	10.44	GNSPPAALGPQDPAPA	9.800909	8.261024
E3 ubiquitin-protein ligase DTX3L isoform X3 [Canis lupus familiaris]	gi|545553062	21.92	AFEQKLIFTVGESR	8.33124	8.224699
E3 ubiquitin-protein ligase FANCL isoform X4 [Homo sapiens]	gi|578802972	19.83	IVLPEDLQLK	8.673717	12.10538
E3 ubiquitin-protein ligase HECTD3 isoform X1 [Canis lupus familiaris]	gi|545524007	19.59	MAGPGPGAALESPR	10.81192	9.819193
E3 ubiquitin-protein ligase HECW2 isoform X2 [Homo sapiens]	gi|578804638	17.73	NPAVMMGAEGMEGGASGNLHS	8.400354	7.355861
E3 ubiquitin-protein ligase HECW2 isoform X4 [Homo sapiens]	gi|578804640	8.3999996	HMITKVR	8.903304	10.45077
E3 ubiquitin-protein ligase HERC2 isoform X3 [Homo sapiens]	gi|578827509	11.17	DFIPGLMYIR	6.37437	9.33454
E3 ubiquitin-protein ligase HERC4 isoform X10 [Canis lupus familiaris]	gi|545495042	11.2	ELVPDVGR	9.173785	10.03247
E3 ubiquitin-protein ligase MARCH8 isoform X4 [Canis lupus familiaris]	gi|545547284	12.68	TLGHSMSHSSNISK	8.545272	7.651867
E3 ubiquitin-protein ligase MIB1 [Canis lupus familiaris]	gi|359320023	19.700001	KEGVGAR	0	5.992401
E3 ubiquitin-protein ligase MSL2 isoform X1 [Homo sapiens]	gi|578807532	16.280001	IAKLNR	3.321138	8.805425
E3 ubiquitin-protein ligase MYCBP2 isoform X15 [Homo sapiens]	gi|578824804	20.92	IELKGPENTLR	7.017045	6.151437
E3 ubiquitin-protein ligase NHLRC1 [Homo sapiens]	gi|40255283	14.3	TGRVVVVHDGRRR	8.138555	6.448465
E3 ubiquitin-protein ligase PDZRN3 isoform X2 [Homo sapiens]	gi|530373631	8.3699999	ASDGSR	8.378882	8.689096
E3 ubiquitin-protein ligase pellino homolog 2 isoform X1 [Homo sapiens]	gi|578825982	9.0200005	NPDGHMDGLTTNGVLVMHPR	7.412404	9.79045
E3 ubiquitin-protein ligase RNF123 isoform X2 [Canis lupus familiaris]	gi|545533704	24.5	ATIVSVEDWDK	7.407377	9.055611
E3 ubiquitin-protein ligase TRIM37 isoform X5 [Canis lupus familiaris]	gi|545511523	16.889999	KMVTLGANAKGGR	4.483146	8.860353
E3 ubiquitin-protein ligase TRIM56 [Canis lupus familiaris]	gi|545500756	17.719999	AGGDLR	7.874546	6.112765
E3 ubiquitin-protein ligase TRIP12 isoform X11 [Homo sapiens]	gi|578805196	13.7	TCPFFFPFDTR	8.202487	9.695143
E3 ubiquitin-protein ligase UBR1 isoform X4 [Canis lupus familiaris]	gi|545549623	12.33	ENLRCPLNEEVIAQARK	9.201277	7.990449
E3 ubiquitin-protein ligase UBR4 isoform X1 [Canis lupus familiaris]	gi|545492116	11.84	MAGVMAQCGGLECMLNRLAGI	6.796479	8.351676
E3 ubiquitin-protein ligase UBR4 isoform X13 [Homo sapiens]	gi|578798776	10.44	KIDAAR	1.983473	4.326686
E3 ubiquitin-protein ligase XIAP isoform X1 [Homo sapiens]	gi|578838669	12.7	MTFNSFEGSK	5.164847	0
E3 UFM1-protein ligase 1 [Homo sapiens]	gi|24308039	13.6	KSSVTEE	0	7.172923
echinoderm microtubule-associated protein-like 5 isoform X5 [Homo sapiens]	gi|578825578	12.78	KDMISDIRFSPGSGK	8.818854	6.711053
echinoderm microtubule-associated protein-like 6 isoform X1 [Homo sapiens]	gi|578802899	3.26	MLAVR	13.23972	9.980194
ectodysplasin-A receptor-associated adapter protein isoform A [Homo sapiens]	gi|153267418	8.4799995	MGLRTTKQMGRGTK	10.41564	6.804855
ecto-NOX disulfide-thiol exchanger 1 isoform X2 [Canis lupus familiaris]	gi|545537518	12.16	FSPADPSVDIALR	6.835318	7.549021
ectonucleoside triphosphate diphosphohydrolase 4 isoform X2 [Canis lupus familiaris]	gi|545542455	10.54	RTLRSASTATLWLEEGLPSQK	7.981278	11.3476
elastin, partial [Homo sapiens]	gi|1389764	6.8200002	AAKYG	9.314213	7.86052
electron transfer flavoprotein subunit beta isoform 2 [Homo sapiens]	gi|62420877	21.129999	TALAMGADR	10.01064	9.048034
electroneutral sodium bicarbonate exchanger 1 isoform c [Homo sapiens]	gi|385719213	10.08	VTGLMIFVLMGCSVFMTAILK	10.33533	8.811078
ELK1, member of ETS oncogene family variant, partial [Homo sapiens]	gi|62087674	16.43	GAGMAGPGGLAR	9.34305	9.846015
ELL-associated factor 2 [Canis lupus familiaris]	gi|345796220	10.78	MNAAAGPASR	10.1105	9.030386
elongation factor Ts, mitochondrial isoform 4 precursor [Homo sapiens]	gi|291084502	4.71	HTFYAGPRLSASASSK	11.36043	5.9716
elongation of very long chain fatty acids protein 5 isoform X2 [Homo sapiens]	gi|530382330	21.17	ALLGPR	7.329392	7.647023
Em:AC002472.7 [Homo sapiens]	gi|47678215	13.65	CPHYGAPLVKGVLSR	10.07281	8.987778
embigin [Canis lupus familiaris]	gi|545496580	10.05	MYNEFVNIKPGTLK	4.6705	5.319995
embryonal Fyn-associated substrate isoform X3 [Canis lupus familiaris]	gi|545506834	13.28	AAVLAVK	10.06315	8.923232
enamelin isoform X1 [Homo sapiens]	gi|578808699	19.030001	KSSAPKR	10.11894	9.045501
endosulfine alpha, isoform CRA_d [Homo sapiens]	gi|119573916	8.4099998	MKNKQLPILTTWLMWGR	7.497882	7.02633
ensconsin isoform X3 [Homo sapiens]	gi|578813062	12.32	AVSPSNPKARQPARSR	6.644234	8.181945
envoplakin [Homo sapiens]	gi|156104874	11.75	TMQPHLLTK	9.27467	10.8163
envoplakin isoform X2 [Canis lupus familiaris]	gi|545509354	9.6999998	ALHLQEEAKLLSQR	13.25292	10.23997

eosinophil peroxidase isoform X1 [Homo sapiens]	gi|578831504	10.39	LLVDAAYNWTQKSIK	7.449821	10.52363
epidermal growth factor receptor kinase substrate 8-like protein 1 isoform X4 [Canis lupus familiaris]	gi|545487378	14.84	QRDVLEVLDDRR	11.93091	10.04084
epidermal retinol dehydrogenase 2 [Canis lupus familiaris]	gi|73999403	18.969999	EGNEDTCR	13.42071	11.67179
epiplakin [Canis lupus familiaris]	gi|73974852	15.9	MNGHASPPPDALAASSTALTGP	5.298473	11.86545
equilibrative nucleoside transporter 4 isoform X3 [Homo sapiens]	gi|578813358	14.04	GYGYR	9.218885	9.634166
ER membrane protein complex subunit 2 [Homo sapiens]	gi|7661910	6.73	KDNMK	6.803758	12.41388
ERC protein 2 isoform X7 [Homo sapiens]	gi|578805962	26.27	MSVLKEQMR	10.26768	9.250429
ERI1 exoribonuclease 3 isoform X2 [Homo sapiens]	gi|530363292	9.0900002	TLPTS	10.39998	0
estradiol 17-beta-dehydrogenase 1 isoform X5 [Homo sapiens]	gi|578830830	12.64	FLPLLRMR	6.830405	6.219609
ethanolamine-phosphate cytidylyltransferase isoform X1 [Homo sapiens]	gi|578832034	20.540001	RTQGVSTTDLVGR	8.917539	8.695657
ethylmalonyl-CoA decarboxylase isoform X2 [Homo sapiens]	gi|530383866	12.84	CCAGSRLGIGWR	7.189631	11.89427
eukaryotic elongation factor 2 kinase [Homo sapiens]	gi|530437751	15.49	KYESDEDSLGSSGR	9.127988	9.001681
eukaryotic translation initiation factor 2-alpha kinase 1 isoform a [Homo sapiens]	gi|11125768	16.08	SREVALEAQTSR	10.35804	7.736418
exocyst complex component 3-like protein 2 [Canis lupus familiaris]	gi|73948176	15.67	LARPGLACLPR	9.150955	9.024329
exonuclease 3'-5' domain-containing protein 1 isoform X5 [Canis lupus familiaris]	gi|545549483	12.99	YLSFLEERQK	11.79414	0
exonuclease 3'-5' domain-containing protein 2 isoform X5 [Homo sapiens]	gi|578825934	13.6	KVLEKCQGVVDIPFR	11.99119	8.490435
exportin-5 [Homo sapiens]	gi|22748937	20.68	AVRNVRR	4.67806	6.776141
extended synaptotagmin-3 [Canis lupus familiaris]	gi|359322565	10.5	EMGSPYTGPEALKKGPLFIKK	10.37894	10.13909
extracellular serine/threonine protein kinase FAM20C [Canis lupus familiaris]	gi|545501336	11.41	MKMILVR	10.87856	7.450276
Fanconi anemia core complex-associated protein 20 isoform 5 [Homo sapiens]	gi|379643019	14.17	LGLSRRRPPPAGGSPGAAR	9.307537	10.10623
Fanconi anemia group B protein [Canis lupus familiaris]	gi|359324155	11.54	EMTLKLAEVQLR	9.760408	7.965384
Fanconi anemia group D2 protein isoform X2 [Canis lupus familiaris]	gi|545532733	3.1400001	QLVMSK	9.328948	9.685208
Fanconi anemia group G protein [Homo sapiens]	gi|4759336	10.45	VAQNSGLTLRR	9.525065	9.044599
Fanconi anemia group J protein [Canis lupus familiaris]	gi|345805276	10.65	SSLWSEYTIGGVK	8.06442	10.37247
Fanconi anemia-associated protein of 100 kDa [Canis lupus familiaris]	gi|345804746	10.04	MVVQQAAQSCSPPDIR	8.977325	11.32042
FAST kinase domain-containing protein 1 isoform X2 [Homo sapiens]	gi|578804901	19.59	KEFFAKLR	5.206426	8.981271
fast myosin heavy chain 2B [Canis lupus familiaris]	gi|83026764	10.38	KKEESTPGK	10.02638	10.74771
F-BAR and double SH3 domains protein 1 isoform X2 [Canis lupus familiaris]	gi|545490432	11	LAGPFLKK	12.34407	0
F-box only protein 16 isoform 2 [Homo sapiens]	gi|384551665	13.37	MAFAPPK	6.233892	7.246112
F-box only protein 16 isoform X2 [Canis lupus familiaris]	gi|545542355	30.83	LRKAQSLMSR	4.011516	5.853484
F-box protein FBW7 isoform 2 variant, partial [Homo sapiens]	gi|62089030	5.8000002	VWSAVTGK	11.01596	6.485921
F-box/LRR-repeat protein 14 [Homo sapiens]	gi|22748931	10.54	GRAAQVCTAWR	8.802356	9.613356
F-box/LRR-repeat protein 19 isoform 1 [Homo sapiens]	gi|157168349	14.79	ADNGEEGASLGSGWK	9.837845	0
F-box/LRR-repeat protein 19 isoform X7 [Canis lupus familiaris]	gi|545501444	20.719999	MGLEVPGK	7.752769	7.049006
FERM domain-containing protein 4A isoform X14 [Homo sapiens]	gi|578818450	1.63	EQRATNEVVRSDLKK	7.893563	10.18333
fermitin family homolog 3 [Canis lupus familiaris]	gi|359321810	8.9499998	MAGMKTATGDYIDSSWELR	9.152455	6.882276
FGGY carbohydrate kinase domain-containing protein isoform a [Homo sapiens]	gi|164663830	9.7600002	HSVLQYVGGVMSVEMQAPK	9.072002	3.684731
FGGY carbohydrate kinase domain-containing protein isoform X7 [Canis lupus familiaris]	gi|545498666	11.15	IGLEDFVADNYRK	0	7.406353
FH1/FH2 domain-containing protein 1 isoform X6 [Homo sapiens]	gi|578828950	7.2600002	QATYR	8.405515	10.30826
FH2 domain-containing protein 1 isoform X1 [Homo sapiens]	gi|578809572	22.85	RSMNIGIFLK	11.05854	10.11944
FIBA_CANLF RecName: Full=Fibrinogen alpha chain	gi|85701317	12.49	XQSAC	5.949079	7.114679
fibrillin-1 isoform X1 [Canis lupus familiaris]	gi|545549819	8.4700003	CQCPNGMTLDATGR	5.176505	9.540165
fibrinogen silencer-binding protein [Homo sapiens]	gi|371502131	4.3499999	MTSSPSSIPR	10.69117	3.560543
fibroblast growth factor 12 isoform X3 [Homo sapiens]	gi|578807164	15.71	SSGTPTMNGGK	11.75584	10.86712
fibroblast growth factor receptor substrate 3 isoform X1 [Homo sapiens]	gi|578811367	15.56	RHGGGTR	10.00828	9.978507
fibrous sheath-interacting protein 1 isoform X5 [Homo sapiens]	gi|578826684	20.77	KRLVELLK	11.59239	7.951759
fibrous sheath-interacting protein 2 [Homo sapiens]	gi|297206791	20.67	SISDIPVSK	8.912717	8.200903
fibrous sheath-interacting protein 2 isoform X3 [Canis lupus familiaris]	gi|545555445	17.799999	DIKSNGENLAR	4.05051	7.956975
filamin A-interacting protein 1-like isoform 1 [Homo sapiens]	gi|109659845	11.61	GSDTEGSAQKK	4.144905	9.79304
FLJ00200 protein, partial [Homo sapiens]	gi|18676604	6.3699999	CSEWGRR	8.154791	8.8571
FLJ27365 protein, isoform CRA_a [Homo sapiens]	gi|119593804	16.32	SPPPQK	8.20177	9.814656
FLJ41423 protein [Homo sapiens]	gi|119588447	12.74	GENDGGEER	11.03601	9.011539
FMR1, partial [Homo sapiens]	gi|26986715	14.11	RADGEAGGGGDGGAAARGR	6.32647	8.974793
FN5 protein, isoform CRA_c, partial [Homo sapiens]	gi|119587308	10.73	EMREVDACGLSAALMR	4.444703	8.044818
forkhead box F1 [Homo sapiens]	gi|119615830	12.48	AQPPPLRPPPPR	8.080344	7.875647
forkhead box L1-like [Canis lupus familiaris]	gi|545501341	4.52	SPGGEGAR	7.198948	7.207281
forkhead box protein F1 [Canis lupus familiaris]	gi|545499598	14.5	GGGGGGGGGAAMDPVASGPAK	7.357304	8.677297
formimidoyltransferase-cyclodeaminase isoform X2 [Canis lupus familiaris]	gi|545551545	11.04	EGAALASMAGLMTYGR	10.21567	7.861926
formin-1 isoform X6 [Canis lupus familiaris]	gi|545549286	17.440001	HLKAGK	9.433495	9.296986
formin-like protein 16-like [Canis lupus familiaris]	gi|545557054	7.1500001	GLRGGAGR	10.99784	10.0556
FRAP1 variant protein, partial [Homo sapiens]	gi|68533045	11.82	AGKAGGGAR	7.697842	10.98962
FRAS1-related extracellular matrix protein 2 precursor [Homo sapiens]	gi|79749430	17.01	MDSFEFQVTDGR	5.855608	10.16498
frizzled-5 precursor [Homo sapiens]	gi|27894385	15.43	MSCDRLPVLGR	12.83378	10.60321
fructose-1,6-bisphosphatase 1 isoform X1 [Homo sapiens]	gi|578817185	1.39	KSPNGK	5.669764	8.247852
FVSY9334 [Homo sapiens]	gi|37181392	14.94	LLGTPGR	8.566573	7.67294
G Chain G, Structure Of A Complex Of The Golgin-245 Grip Domain With Arl1	gi|39655049	6.6900001	EXRILILGLDGAGK	4.426773	7.727787
G Chain G, The Nucleosome Containing Human H2a.z.2	gi|567755107	9.4499998	GSHMAGGK	8.017683	6.555235
G patch domain-containing protein 2-like isoform X2 [Homo sapiens]	gi|578825940	2.3199999	TAPLP	8.317992	5.350138
G patch domain-containing protein 8 isoform X6 [Canis lupus familiaris]	gi|545510344	10.57	DEGGGGGDSQDHGGRK	8.010332	8.072395
G protein-coupled receptor kinase 6 isoform B [Homo sapiens]	gi|51896035	23.16	EGGGGNRKGKSK	8.14516	5.954678
G protein-regulated inducer of neurite outgrowth 1 isoform X1 [Homo sapiens]	gi|530380833	12.21	EDPGSSRKADPMFTGKAEPEILC	11.73144	7.519032
G2 and S phase-expressed protein 1 isoform X1 [Homo sapiens]	gi|530420136	11.69	EGGGGR	7.838278	8.783562
galactose-3-O-sulfotransferase 2 isoform X3 [Canis lupus familiaris]	gi|545543232	18.969999	VQAELGPR	9.684748	9.13872
galactose-3-O-sulfotransferase 4 [Canis lupus familiaris]	gi|73958010	15.95	SAVSNSGLTTEDMQLTAR	9.099678	9.178793

galectin-12 isoform X2 [Canis lupus familiaris]	gi|545531107	5.23	GMPVTTT	4.09289	6.893265
gamma-aminobutyric acidA receptor gamma 2 short form subunit {intracellular loop} [human, cerebral cortex, Peptide Partial, 85 aa]	gi|266008	0.46	IKMDS	0.5435348	4.439767
gamma-crystallin B [Homo sapiens]	gi|148491074	9.0500002	VMDLY	8.363733	10.74051
gap junction delta-4 protein [Homo sapiens]	gi|145699105	7.4899998	GSGSEEQPSAAPSR	6.403089	0
gasdermin-A [Canis lupus familiaris]	gi|545510858	10.02	SLLTSLSKLLGKK	9.73508	8.802949
gastrin/cholecystokinin type B receptor isoform X1 [Homo sapiens]	gi|530395959	12.38	ATGPAGVGGTEMKVR	9.320771	8.301009
GCN1 general control of amino-acid synthesis 1-like 1 (yeast), isoform CRA_b [Homo sapiens]	gi|119618575	35.720001	LGLLVQFCTSHK	1.762887	6.158792
GDF5, partial [Homo sapiens]	gi|290565905	12.93	APPXAGSVPSSFLLKK	10.60608	10.47599
genethonin 1, isoform CRA_f [Homo sapiens]	gi|119626192	14.5	KAMDI	10.00195	6.199293
genetic suppressor element 1 isoform X9 [Homo sapiens]	gi|578828929	23.07	QKMVSER	4.807312	7.565872
germ cell-less homolog 1 (Drosophila)-like [Homo sapiens]	gi|119574231	0.68	MGSSSSR	6.289197	7.804873
germinal center-associated signaling and motility protein isoform 4 [Homo sapiens]	gi|298231245	17.5	GNSLLRENRR	8.031744	7.867174
GFC1_HUMAN RecName: Full=HERV-F(c)1_Xq21.33 provirus ancestral Gag polyprotein	gi|52000661	0.76	EETAEAQR	7.541878	6.554283
GH3 domain-containing protein isoform X2 [Canis lupus familiaris]	gi|545510586	8.5699997	AAGGTRSR	3.223134	5.890024
gliomedin [Canis lupus familiaris]	gi|345794946	11.76	RGKMGLPGATGAPGEK	9.136494	8.512016
gluconokinase isoform 1 [Homo sapiens]	gi|299758445	15.81	MAAPGALLVMGVSGSGK	9.337427	8.833814
glucose 6-phosphate translocase [Homo sapiens]	gi|3859932	15.22	TKMGRVSK	13.13134	8.037536
GluR-delta2 philic-protein [Homo sapiens]	gi|51094465	7.3600001	AAGVPR	6.317071	9.097255
glutamate receptor ionotropic, kainate 4 isoform 2 precursor [Homo sapiens]	gi|541862236	12.3	GYGIGMPVGMR	8.397135	9.1716
glutamate receptor ionotropic, kainate 5 isoform X5 [Homo sapiens]	gi|578834355	18.76	DPTPLLKEIR	5.410118	6.572353
glutamate-rich protein 6 isoform X1 [Homo sapiens]	gi|530373923	8.29	MSEMSIDR	4.812233	8.215558
glutamine-dependent NAD synthetase [Homo sapiens]	gi|28849201	11.5	VAKMGPYSMFCKLLGMR	10.08497	10.19269
glutathione-specific gamma-glutamylcyclotransferase 1 [Canis lupus familiaris]	gi|545550636	5.0799999	RPPGXVPVPSTMK	4.287635	6.7723
glycerophosphodiester phosphodiesterase domain containing 5, isoform CRA_b, partial [Homo sapiens]	gi|119595374	13.85	ADSSSSEHPKALGKAPRFGACGI	10.09743	10.0936
glycerophosphodiester phosphodiesterase domain-containing protein 1 isoform X2 [Canis lupus familiaris]	gi|545511533	10.2	FLIWLSDTLLMR	8.346213	10.28236
glycine amidinotransferase, mitochondrial [Canis lupus familiaris]	gi|559767210	5.0799999	LGISTIK	9.626878	7.245902
glycogen [starch] synthase, muscle isoform 2 [Homo sapiens]	gi|239049591	10.88	NRTERLSDLLDWK	8.313725	9.306856
glycolipid transfer protein domain-containing protein 2 isoform X2 [Canis lupus familiaris]	gi|545497830	10.11	MAPAAGTGVAMLPLVSR	13.09094	13.88564
glypican-2 precursor [Homo sapiens]	gi|22749459	4.0900002	QVLGAR	6.218651	7.525378
Golgi integral membrane protein 4 isoform X3 [Canis lupus familiaris]	gi|545553997	15.97	KKVEGSLFQSIVFK	11.83357	11.01935
golgin subfamily A member 3 isoform X3 [Canis lupus familiaris]	gi|545543403	9.1000004	AATLELSEVKKELQAKEQLVQK	11.99041	11.19272
golgin subfamily A member 5 [Homo sapiens]	gi|526118265	27.469999	QELIDYK	6.995985	9.915074
golgin subfamily A member 8J isoform X1 [Homo sapiens]	gi|578827316	9.6800003	ATSGGCQPPRDSATGFHR	4.345657	7.027114
G-protein coupled receptor 101 [Homo sapiens]	gi|16876435	9.96	EGSTK	8.81748	8.425448
G-protein coupled receptor-associated sorting protein 1 [Canis lupus familiaris]	gi|545558729	3.04	TEAGGR	7.443904	5.936929
G-protein coupled receptor-associated sorting protein 2 [Homo sapiens]	gi|315259097	14.71	MSLIDDDFSLEPLISAFR	7.676209	3.967117
G-protein-signaling modulator 1 isoform b [Homo sapiens]	gi|525313752	12.11	MNVAQLQLVLGR	4.229452	6.030522
grainyhead-like protein 3 homolog isoform 1 [Homo sapiens]	gi|38049007	23.41	VPVEQLR	8.192849	12.42178
GRAM domain-containing protein 1A isoform X2 [Canis lupus familiaris]	gi|545488941	15.28	GRLTPNLSR	10.753	10.13384
granulocyte-macrophage colony-stimulating factor receptor subunit alpha isoform X10 [Homo sapiens]	gi|578838981	19.719999	AADVR	5.68808	8.885027
granzyme A [Canis lupus familiaris]	gi|545490631	20.99	MPIDVGTAATMR	9.13763	7.302097
GRB2-associated and regulator of MAPK protein-like [Canis lupus familiaris]	gi|359321567	11.61	LGRAGALAGVGGGGGGGGPG	10.20215	10.33165
GRIP1-associated protein 1 isoform X1 [Homo sapiens]	gi|578838047	19.959999	DTVDGQRILEK	5.178834	8.027866
growth hormone-inducible transmembrane protein [Homo sapiens]	gi|118200356	16.389999	TRIGIR	8.147449	7.72462
growth regulation by estrogen in breast cancer-like isoform X5 [Canis lupus familiaris]	gi|545506251	20.610001	KRGTMSMMLTK	11.33245	10.60429
GTPase-activating Rap/Ran-GAP domain-like protein 3 isoform X1 [Homo sapiens]	gi|530391684	20.25	QKRAALRR	8.810857	8.699734
GTP-binding protein 1 isoform X2 [Canis lupus familiaris]	gi|545514814	11.36	GGVQFR	7.667735	7.225266
GTP-binding protein REM 2 isoform X1 [Homo sapiens]	gi|530403202	1.53	GSMPVPYK	8.872429	10.7377
guanine nucleotide exchange factor MCF2L2 [Homo sapiens]	gi|540344580	9.4899998	DEEETATRSTEEERAGASTGR	7.896303	4.619977
guanylate cyclase soluble subunit alpha-3 isoform X1 [Homo sapiens]	gi|578809076	6.7199998	TLAKHK	5.861533	9.89005
guanylate cyclase soluble subunit alpha-3 isoform X2 [Homo sapiens]	gi|578809078	8.6499996	ASGID	8.46225	9.230607
H Chain H, Crystal Structure Of An Unliganded (Native) Fv From A Human Igm Anti-Peptide Antibody	gi|10835403	12.13	QAPGKGLEWVAVISSDGGNK	8.429915	6.353519
H Chain H, Crystal Structure Of The Dbl And Pleckstrin Homology Domains Of Dbs In Complex With Rhoa	gi|21466031	6.8400002	KKSGS	0	8.512319
H(+)/Cl(-) exchange transporter 7 isoform a [Homo sapiens]	gi|14149607	19.459999	EIPHNEKLLSLK	4.339161	7.561756
hCG15585 [Homo sapiens]	gi|119591074	9.2600002	ELAGGILSLYLMPILPVPSSSWEI	8.700808	4.226629
hCG1639925, isoform CRA_a, partial [Homo sapiens]	gi|119572448	13.81	QSSLGAMEAPR	7.402319	5.492742
hCG1640457, isoform CRA_a [Homo sapiens]	gi|119605917	12.39	FHKRYFTLGRAWAGAPT	11.45796	9.350851
hCG1640714, isoform CRA_b, partial [Homo sapiens]	gi|119602762	12.5	ATDKGDAR	8.567423	9.011048
hCG1641872, isoform CRA_b, partial [Homo sapiens]	gi|119627576	20.51	MARPGPDLSRMRR	6.847463	10.50418
hCG1643801 [Homo sapiens]	gi|119593357	10.84	TRRTSCKK	10.2056	8.813941
hCG1644012, partial [Homo sapiens]	gi|119603930	23.950001	HTDCVVMK	11.90221	11.33456
hCG1644145, partial [Homo sapiens]	gi|119611283	4.6799998	AANVLK	0	5.529482
hCG1644435, isoform CRA_b, partial [Homo sapiens]	gi|119620307	5.48	MSGHKGGK	5.696766	6.421496
hCG1644968 [Homo sapiens]	gi|119632110	10.18	MTVSDK	6.838809	8.728823
hCG1645016 [Homo sapiens]	gi|119590907	14.4	SRTRTHRGVPR	10.5836	10.43804
hCG1646042 [Homo sapiens]	gi|119625493	5.4299998	GTSSSDLR	9.539786	9.171619
hCG1647019, isoform CRA_a, partial [Homo sapiens]	gi|119620582	21.18	APEKSLTVNSPR	8.780897	9.491038
hCG1649618 [Homo sapiens]	gi|119600860	8.6599998	AGMDSGSLVKLPSCALHK	12.26202	12.51397

hCG1649732, isoform CRA_b, partial [Homo sapiens]	gi|119615590	6.98	LRASESGRGAESGSQKPVGARM	6.976959	7.719974
hCG1654062 [Homo sapiens]	gi|119627151	19.02	TEANGT	8.209194	0
hCG1656033 [Homo sapiens]	gi|119623507	3.9100001	VQAPQEDLR	3.22523	4.445701
hCG1657257 [Homo sapiens]	gi|119575075	11.46	EPKAANATMAHK	8.24729	4.03449
hCG1658583, isoform CRA_a, partial [Homo sapiens]	gi|119605695	10.96	MMSPKIEVAKMGTPR	3.148332	5.417432
hCG1658793, isoform CRA_b [Homo sapiens]	gi|119581620	20.26	HMIGKPLDGK	8.080997	10.37082
hCG1660224, isoform CRA_b [Homo sapiens]	gi|119601085	19.49	QQKESRSVAR	9.267375	9.074351
hCG1741626 [Homo sapiens]	gi|119628636	1.79	KKPYR	5.912203	7.875817
hCG1742442, isoform CRA_a, partial [Homo sapiens]	gi|119606953	16.059999	LFAHPPPR	9.092247	8.815933
hCG1746338 [Homo sapiens]	gi|119625980	12.77	AGAHK	7.140893	7.119034
hCG1746998 [Homo sapiens]	gi|119600832	14.52	PPDPGLLSYIDK	4.400267	5.553211
hCG1766505 [Homo sapiens]	gi|119573069	2.45	TAGPGR	7.475155	8.318431
hCG1778862, isoform CRA_a [Homo sapiens]	gi|119569169	21.32	SHASGVKTSPDPR	7.776758	9.472324
hCG1779407, partial [Homo sapiens]	gi|119605335	17.09	QAEVAGVGLLAVK	9.637938	10.51784
hCG1781582, partial [Homo sapiens]	gi|119603082	19.059999	QEIAEQLENIGLLLQSQK	7.895525	8.14919
hCG1783157, partial [Homo sapiens]	gi|119630550	13.78	SRGTLQKGVK	7.718078	12.36568
hCG1783243 [Homo sapiens]	gi|119630204	14.32	KLMTVRIIK	11.96748	8.737394
hCG1783746, partial [Homo sapiens]	gi|119621264	7.4299998	SGNDLAS	8.054226	5.674651
hCG1784521, partial [Homo sapiens]	gi|119606618	11.71	HLPADR	7.266215	8.744192
hCG1784882 [Homo sapiens]	gi|119588562	14.2	KKLLLLLSVTKR	5.325499	8.337845
hCG1790262 [Homo sapiens]	gi|119572161	17.629999	INVTSEVPFSK	5.248543	8.454514
hCG1793603, isoform CRA_b, partial [Homo sapiens]	gi|119628600	16.959999	GQALSGAQVSSWR	7.247738	9.439814
hCG1800530, isoform CRA_a [Homo sapiens]	gi|119610396	19.120001	EAAILRLSK	7.180384	10.83155
hCG1806822 [Homo sapiens]	gi|119597552	6.0999999	AIVSGPGKAIVSSPGK	8.111413	11.038
hCG1808712 [Homo sapiens]	gi|119571004	19.35	ATVKQIGRAKTPR	7.859546	5.887413
hCG1811814, isoform CRA_b, partial [Homo sapiens]	gi|119623593	17.08	GIEEQLGK	6.419742	4.618146
hCG1812010 [Homo sapiens]	gi|119626233	14.84	KLDAVSLTLLPR	9.372485	7.519499
hCG1813549 [Homo sapiens]	gi|119599497	18.129999	QHCEQNMEG	11.01017	9.20074
hCG1815277 [Homo sapiens]	gi|119582885	2.52	KSSLAPH	7.274175	5.53426
hCG1815516 [Homo sapiens]	gi|119603171	11.88	MCISK	6.72273	7.588588
hCG1816500 [Homo sapiens]	gi|119607263	8.71	YSLSLKK	7.0211	7.693208
hCG1816973 [Homo sapiens]	gi|119619415	15.13	MFPEVSLSGR	13.98641	9.427387
hCG1817987 [Homo sapiens]	gi|119612015	21.66	LIDDRR	9.700461	9.264044
hCG1820498 [Homo sapiens]	gi|119577805	8.3299999	TTWKIFLNSCLSKIK	8.581983	0
hCG1820678 [Homo sapiens]	gi|119622066	16.17	MHLHRLLDILLKK	4.813256	6.221555
hCG18315, isoform CRA_c [Homo sapiens]	gi|119628610	6.0599999	GPQWSCLVSMGTPHHR	8.942853	7.519039
hCG1979866, partial [Homo sapiens]	gi|119582201	16.84	VLALGK	5.345107	6.541163
hCG1980758, partial [Homo sapiens]	gi|119582059	12.56	SPRGTHPLPGLVER	9.033106	9.205238
hCG1985461, partial [Homo sapiens]	gi|119610677	8.5699997	GEPSARGSPIPPLTAPPPR	10.75084	9.716221
hCG1986453 [Homo sapiens]	gi|119609571	14.09	NTPFLQHRSR	13.21911	7.753577
hCG1986496 [Homo sapiens]	gi|119582162	14.41	KVSVASLNQLGKVVPMVLSSISI	9.132913	6.976363
hCG1987119, isoform CRA_c [Homo sapiens]	gi|119609694	8.3400002	QGGAKAPK	8.520821	9.334228
hCG1987937, isoform CRA_d, partial [Homo sapiens]	gi|119575125	23.33	REIVDSLSVLPR	10.4695	9.126988
hCG1988320, isoform CRA_a [Homo sapiens]	gi|119620880	8.4700003	MLDDLMEK	5.694376	6.131178
hCG1988537, partial [Homo sapiens]	gi|119570893	21.309999	YLTWASR	9.528618	11.30752
hCG1988537, partial [Homo sapiens]	gi|119570893	49.259998	DASGVTFTWTPSSGK	10.04693	12.48865
hCG1988537, partial [Homo sapiens]	gi|119570893	93.290001	QEPSQGTTTFAVTSILR	8.467889	12.60036
hCG1989313, isoform CRA_c [Homo sapiens]	gi|119609749	9.96	VSGLVSRGPL	7.451386	11.70167
hCG1989907 [Homo sapiens]	gi|119628429	5.5799999	SAFWR	7.634225	7.015854
hCG1992315 [Homo sapiens]	gi|119618984	9.71	KVSGYK	11.07782	10.56924
hCG1993711, partial [Homo sapiens]	gi|119574252	15.95	AAAGQVPGEAGARR	10.05041	10.11474
hCG1997148, partial [Homo sapiens]	gi|119577043	12.8	AISNNEYIWVFTGAGR	9.824289	8.525376
hCG2000277, isoform CRA_a, partial [Homo sapiens]	gi|119624427	15.37	HIMKNLLK	10.28569	10.56192
hCG2002607, partial [Homo sapiens]	gi|119623477	16.25	EGLQSQMALASPR	5.921616	7.231204
hCG2002820 [Homo sapiens]	gi|119614845	11.47	SSMFVTPEPIYK	6.863821	10.43653
hCG2004878, isoform CRA_a [Homo sapiens]	gi|119589514	7.25	GPGTL	7.598133	9.423759
hCG2005032 [Homo sapiens]	gi|119623672	10.64	MMNVSLVGK	8.009418	3.427687
hCG2005294, partial [Homo sapiens]	gi|119589859	22.530001	AHGAVDTTGK	9.105767	11.83531
hCG2007771 [Homo sapiens]	gi|119630298	16.77	LGIGGIGRRLWR	4.79344	0
hCG2007967, partial [Homo sapiens]	gi|119593232	25.809999	MRSQNAALSGSGPR	5.587793	0
hCG2008833, partial [Homo sapiens]	gi|119612372	15.29	AAVTPR	10.4116	8.965381
hCG2008892, partial [Homo sapiens]	gi|119612373	22.459999	GEALSPPICINVR	12.56274	9.565553
hCG2010232, isoform CRA_c, partial [Homo sapiens]	gi|119614499	13.2	NGIDQEIIFPPIK	6.248444	10.41211
hCG2011167, partial [Homo sapiens]	gi|119580114	12.99	EGIDLGDLDMR	8.264894	7.673687
hCG2011409 [Homo sapiens]	gi|119579577	12.74	AALIKGR	5.387548	8.989154
hCG2012269, partial [Homo sapiens]	gi|119629636	11.91	LLLVSAR	4.391676	2.97089
hCG2013046 [Homo sapiens]	gi|119629564	19.41	CLIMRR	5.827068	5.37621
hCG2014067, partial [Homo sapiens]	gi|119603821	4.7600002	AGRGH	3.318166	5.465644
hCG2017588 [Homo sapiens]	gi|119617157	2.4000001	MAQIIKPR	7.479019	5.360797
hCG2017590 [Homo sapiens]	gi|119606648	11.36	EYGVKHMPMGNNAGNLEPEKR	8.81289	7.563922
hCG2018311 [Homo sapiens]	gi|119606809	13.97	SPLEMAFRCRFLFHRSGWSPK	8.261642	5.134996
hCG2018444, partial [Homo sapiens]	gi|119595610	18.42	DREKVQAGLGSGPVGSSGR	7.316937	8.6222
hCG2019454, partial [Homo sapiens]	gi|119596033	4.8200002	MRSTFAR	0	8.92604
hCG2019817, isoform CRA_b [Homo sapiens]	gi|119631082	4.5300002	MGCGR	6.607105	6.899353
hCG2021576, partial [Homo sapiens]	gi|119608597	18.139999	QLPLLALCLAAPKAMGESQSHL(	11.53352	6.316931

hCG2021598, isoform CRA_a, partial [Homo sapiens]	gi|119598468	23.379999	IRTPSAMSISKEITF	11.52176	5.064821
hCG2022586, isoform CRA_c [Homo sapiens]	gi|119599705	11.98	AGSPASLSR	3.790576	6.357887
hCG2027994, partial [Homo sapiens]	gi|119626682	18.68	LGVWNVSAPDRK	10.0313	11.13144
hCG2032840, partial [Homo sapiens]	gi|119588096	4.48	MSPQEAPR	5.326039	5.591021
hCG2036543 [Homo sapiens]	gi|119601762	10.6	VRLLAKGK	10.40448	12.11855
hCG2036564, isoform CRA_b [Homo sapiens]	gi|119600045	7.8000002	EMCIKTDQVTSQFLK	0	7.2018
hCG2036642 [Homo sapiens]	gi|119592457	1.67	HQRTHTGEKPYHCSECGK	7.456846	9.767761
hCG2036663, isoform CRA_a, partial [Homo sapiens]	gi|119597543	17.860001	FIVSEPPVSPS	10.16503	9.571538
hCG2036684, partial [Homo sapiens]	gi|119580087	13.17	ERKEKMLSKPEMGSPR	5.404751	10.70517
hCG2036717 [Homo sapiens]	gi|119571781	15.28	KDNKHLLM	3.55149	0
hCG2037010 [Homo sapiens]	gi|119597204	7.9299998	LGNNTLKNMNPVDPFEEARCQI	7.361323	6.653225
hCG2038676, partial [Homo sapiens]	gi|119628504	12.35	ANDMR	9.198213	7.386607
hCG2038787, partial [Homo sapiens]	gi|119569976	4.5599999	QEAVSSR	11.12763	8.127033
hCG2038942, partial [Homo sapiens]	gi|119602344	43.27	QIQVSWLR	12.50879	13.07999
hCG2038968, partial [Homo sapiens]	gi|119569668	9.1999998	KGGVNLK	7.217646	5.374361
hCG2040281, partial [Homo sapiens]	gi|119586289	15.39	ILGPVECTVQGGR	8.815085	6.021474
hCG2040419 [Homo sapiens]	gi|119629662	14.03	AASGSR	6.027202	6.99748
hCG2040430, partial [Homo sapiens]	gi|119608946	4.7199998	GLISTRHLR	8.045298	8.300784
hCG2040565, partial [Homo sapiens]	gi|119573509	15.25	SARLSAKPSSPKPEPKPKK	8.2889	7.019834
hCG2040568, partial [Homo sapiens]	gi|119578190	8.46	VKLGLR	9.009238	7.591938
hCG2040862, partial [Homo sapiens]	gi|119623698	15.54	KMLVVSLSGLR	7.760274	9.06196
hCG2041053, partial [Homo sapiens]	gi|119583844	12.02	METPVTYSLTITNEN	9.998826	5.711788
hCG2041144, partial [Homo sapiens]	gi|119623134	7.73	KVRSSSGK	5.018024	5.693011
hCG2041324 [Homo sapiens]	gi|119597553	2.49	TGLSFHEKK	10.81509	9.980968
hCG2041438, partial [Homo sapiens]	gi|119625555	6.5999999	IGNSLSA	6.913126	6.048418
hCG2041480, partial [Homo sapiens]	gi|119604330	12.41	AGGSAGSRS	9.949605	9.546505
hCG2041505, partial [Homo sapiens]	gi|119621807	10.24	DQNKLQLVPDLTIVPS	5.932722	7.789728
hCG2041581, partial [Homo sapiens]	gi|119597090	8.9799995	AGILAR	6.063549	6.767042
hCG2041707, partial [Homo sapiens]	gi|119632061	15.12	ASIIAFK	8.758875	8.673518
hCG2041727, partial [Homo sapiens]	gi|119620268	12.49	QASMMK	4.265095	6.203955
hCG2041776, partial [Homo sapiens]	gi|119620585	9.9499998	LFIAN	5.945796	5.387939
hCG2042886, isoform CRA_b [Homo sapiens]	gi|119583634	10.38	KGQLPPQAMVAETKAMGR	3.383605	7.509771
hCG2043033, isoform CRA_b [Homo sapiens]	gi|119602706	10.4	MKLQPKPYGVTSARASEGAPMI	6.733867	9.312366
hCG2044057 [Homo sapiens]	gi|119604339	8.5699997	VAGGGGR	7.394664	8.200995
hCG2044971 [Homo sapiens]	gi|119569800	8.7600002	TGLVG	4.993619	6.362442
hCG2045126 [Homo sapiens]	gi|119581988	20.440001	GQSLMAAGYFLMSSVR	7.880809	6.620285
hCG2045127 [Homo sapiens]	gi|119581906	9.8500004	KPSRGK	7.127458	8.151548
hCG2045385 [Homo sapiens]	gi|119600006	11.42	QGLSKPSVEEPSRISAR	10.21801	8.873478
hCG2045453 [Homo sapiens]	gi|119605967	24.629999	QPITER	8.245157	6.931254
hCG2045543 [Homo sapiens]	gi|119612080	4.4099998	TNNNK	4.420627	4.883576
hCG2045753, partial [Homo sapiens]	gi|119626198	0.46	APPGVSR	6.389189	4.353306
hCG2045811 [Homo sapiens]	gi|119629889	8.2299995	MSVLHARTSAEGSARGGVLGKS	8.804158	8.115946
hCG20693, isoform CRA_a [Homo sapiens]	gi|119613433	5.0500002	VDGKPVNLGLWDTAGQEDYDR	10.15131	5.541564
hCG21816, isoform CRA_a, partial [Homo sapiens]	gi|119591985	20.67	KFPGASAAAQGVLGCGPR	11.41829	7.062221
hCG22014 [Homo sapiens]	gi|119610290	12.08	GGISSK	10.71276	10.04249
hCG23738, isoform CRA_c [Homo sapiens]	gi|119616816	6.8200002	AKMASATSSSQRDWDK	8.845498	9.563259
hCG24169, isoform CRA_b [Homo sapiens]	gi|119611496	14.7	TSSPMAPQPPR	11.30952	9.116124
hCG24169, isoform CRA_b [Homo sapiens]	gi|119611496	15.92	TVGPNPEPVLSPR	10.31627	10.70491
hCG32285, isoform CRA_b [Homo sapiens]	gi|119598363	14.68	ADAGGKVQEGNLK	7.889071	9.99524
hCG37332 [Homo sapiens]	gi|119569294	13.5	MAAAVAGLLR	2.699354	7.772023
hCG39586, partial [Homo sapiens]	gi|119575391	8.8800001	LGNLSCAGATGPEPSQKPKGR	3.447418	6.826816
HEAT repeat containing 7A [Canis lupus familiaris]	gi|545521846	27.040001	AATVMVNCLLK	5.766621	7.113186
HEAT repeat-containing protein 1 [Homo sapiens]	gi|73695475	15.17	VAVEDSVFLVFSLK	8.202019	9.34234
HEAT repeat-containing protein 5B isoform X2 [Canis lupus familiaris]	gi|545527576	10.03	KLMTPIECAMTMMSHIPSVIK	0	9.030068
heat shock 70 kDa protein 1-like isoform X1 [Homo sapiens]	gi|530429272	15.07	KFNDPVVQADMK	10.40183	14.45167
heat shock factor protein 1 isoform X3 [Canis lupus familiaris]	gi|545521223	12.2	QESMDSKLLAMK	8.947842	11.09229
heat shock factor-binding protein 1-like protein 1 [Canis lupus familiaris]	gi|345784815	8.7200003	ASLGN	5.955533	4.753221
helicase SKI2W isoform X1 [Homo sapiens]	gi|578840867	17.030001	LTYTMILNLLR	0	7.651626
helicase SRCAP [Homo sapiens]	gi|146219843	18.719999	RTSADVEIR	7.146729	8.596345
hemicentin-2 isoform X2 [Homo sapiens]	gi|578844797	3.3900001	AGTGR	9.751075	8.517797
hepatoma-derived growth factor-related protein 2 [Canis lupus familiaris]	gi|545535131	6.96	GEAPASGGSSGDELGDEDEPVR	9.669904	7.897089
HERV-H LTR-associating 1, isoform CRA_b [Homo sapiens]	gi|119612546	7.0999999	FGAVTR	6.709228	8.921487
heterogeneous nuclear ribonucleoprotein A/B isoform b [Homo sapiens]	gi|55956921	8.1499996	AMAMKK	7.576982	7.382142
heterogeneous nuclear ribonucleoprotein F [Homo sapiens]	gi|148470406	16.940001	MLGPEGGEGFVVKLR	7.021775	10.93734
heterogeneous nuclear ribonucleoprotein L, isoform CRA_a [Homo sapiens]	gi|119577230	22.809999	MVKMAAAGGGGGGGRYYGGG	4.826476	5.24357
HFSE-1 [Homo sapiens]	gi|3265150	9.1099997	FMGFR	10.27642	11.68779
high affinity cAMP-specific and IBMX-insensitive 3',5'-cyclic phosphodiesterase 8A isoform X2 [Homo sapiens]	gi|578827080	18.879999	MGYQSGELIGK	4.522179	5.373597
hippocampus abundant transcript-like protein 1 isoform X2 [Homo sapiens]	gi|578817946	13.42	SVEPPPELEEK	10.22167	9.941194
histatin-1 precursor [Homo sapiens]	gi|4504529	18.43	EFPFYGDYGSNYLYDN	2.42937	10.80119
histocompatibility antigen-related [Homo sapiens]	gi|333360918	23.35	EANQCSRGPGSK	10.67915	10.32262
histone deacetylase 2 [Homo sapiens]	gi|293336691	15.7	MAYSQGGGKK	9.621596	10.05858
histone deacetylase 5 isoform X4 [Homo sapiens]	gi|530411260	7.29	AMPSSMGGGGGGSPSPVELR	7.428119	9.446482
histone H1oo isoform X2 [Canis lupus familiaris]	gi|345786311	5.5999999	KPGEGK	7.615264	6.879961
histone H2A type 2-A [Homo sapiens]	gi|106775678	14.9	VTIAQGGVLPNIQAVLLPK	4.701959	0

histone-lysine N-methyltransferase 2A isoform X1 [Homo sapiens]	gi|578822195	14.21	MLAQADKLPMTDKRVASLLK	7.968744	8.218872
histone-lysine N-methyltransferase ASH1L [Canis lupus familiaris]	gi|73960946	12.99	IVANSTMGLVTK	8.42656	8.737575
histone-lysine N-methyltransferase ASH1L isoform X2 [Homo sapiens]	gi|578801274	14.95	QALAAPLLNLPPK	2.23806	6.184518
histone-lysine N-methyltransferase SETD1A isoform X4 [Homo sapiens]	gi|578828708	23.530001	EEAELAEGK	8.527083	8.525596
histone-lysine N-methyltransferase SETD2 isoform X2 [Canis lupus familiaris]	gi|545533918	9.4399996	EKDLDDTCMLHSK	7.925265	7.517555
histone-lysine N-methyltransferase SETD2 isoform X3 [Homo sapiens]	gi|578806017	6.3299999	RPDDR	7.024362	8.063283
histone-lysine N-methyltransferase SUV420H2 [Homo sapiens]	gi|31543169	17.67	THKMNVSPVPPLR	6.503448	6.577394
histone-lysine N-methyltransferase, H3 lysine-36 and H4 lysine-20 specific isoform X2 [Canis lupus familiaris]	gi|545495666	11.62	FNTPSSISSENSLIK	7.716894	11.88896
histone-lysine N-methyltransferase, H3 lysine-79 specific isoform X4 [Homo sapiens]	gi|578833685	14.97	KMNTANPERKPK	4.480047	3.619159
HLA class I histocompatibility antigen, alpha chain F isoform X4 [Homo sapiens]	gi|578842317	3.8599999	TLAMSGK	7.859496	0
HLA class II histocompatibility antigen, DO alpha chain isoform X2 [Homo sapiens]	gi|578842305	16.15	FDPQGGLAGIAAIK	9.967479	9.622633
HLA class II histocompatibility antigen, DQ beta 2 chain-like isoform X2 [Canis lupus familiaris]	gi|545518650	6.1199999	VRFLAKYIYNR	9.35511	6.860081
homeobox protein GBX-1 [Canis lupus familiaris]	gi|545525932	10.29	LRVPGIQNLSR	9.780797	9.118968
homeobox protein Hox-C4 isoform X1 [Canis lupus familiaris]	gi|545545253	14.7	AGQGAGGGPR	8.311762	7.612343
homeobox protein Meis2 isoform X7 [Homo sapiens]	gi|578826968	18.25	KRGIFPK	9.741442	9.79256
homeobox protein Nkx-6.3 [Canis lupus familiaris]	gi|73979193	13.49	AAGGGGGGER	9.423336	10.1241
HORMA domain-containing protein 1 isoform X3 [Canis lupus familiaris]	gi|545528802	12.47	ILMDK	4.903136	3.256225
hormone-sensitive lipase isoform X4 [Homo sapiens]	gi|578834481	12.71	KSQKMSEPIAEPMR	9.184513	10.43974
Human basement membrane heparan sulfate proteoglycan core protein [Homo sapiens]	gi|29470	12.64	KCKAGFFGDAMK	8.716455	8.932194
human HsGCN1 U77700 (PID:g2282576)	gi|3970973	8.7299995	AIITALGVERRNR	3.183703	6.873118
human HsGCN1 U77700 (PID:g2282576)	gi|3970973	18.219999	QKEMLQAQLDR	10.56496	9.353956
Hyaluronan synthase 1 [Homo sapiens]	gi|23243102	7.1199999	MRQDAPKPTPAARR	9.660211	9.271355
hydrocephalus-inducing protein homolog isoform X5 [Homo sapiens]	gi|578829018	7.4400001	METIERKISVR	2.124614	3.815374
hypermethylated in cancer 2 protein isoform X1 [Canis lupus familiaris]	gi|545544907	13.95	KEPMVGSPFER	0	3.694118
hyperpolarization activated cyclic nucleotide-gated potassium channel [Homo sapiens]	gi|28629108	13.01	AXGDGSPGR	8.676547	8.822974
hypothetical LOC285995 [Homo sapiens]	gi|51094567	19.030001	MAATMMASER	0.904612	7.778962
hypothetical LOC340228 [Homo sapiens]	gi|51094651	5.1999998	MNMPFVK	6.707403	6.677739
hypothetical protein [Homo sapiens]	gi|5262479	15.01	HTLDGAACPLNSNK	4.277163	5.933777
hypothetical protein [Homo sapiens]	gi|30268314	11.47	VSMETGL	8.850487	6.768773
hypothetical protein [Homo sapiens]	gi|51476581	15.98	INIMKMAKLPK	9.158461	8.459775
hypothetical protein [Homo sapiens]	gi|31874062	16.42	MSCSWPDEGTDK	2.736528	11.14601
hypothetical protein [Homo sapiens]	gi|21732475	4.77	DGGPK	10.07526	11.19338
hypothetical protein [Homo sapiens]	gi|51476746	10.59	RAGRWYTHSHCCR	11.19782	11.68032
hypothetical protein FLJ30313, isoform CRA_b, partial [Homo sapiens]	gi|119595763	18.75	AVPSRPPRPDK	7.933068	8.090141
hypothetical protein FLJ32110, partial [Homo sapiens]	gi|119597306	18.9	MKSALLLK	7.580203	6.581708
hypothetical protein FLJ37396, isoform CRA_a [Homo sapiens]	gi|119568743	16.620001	NKNMKDDDLSMK	6.767944	0
hypothetical protein FLJ38723, isoform CRA_c [Homo sapiens]	gi|119598019	9.0799999	QGGGGK	5.539485	5.178702
hypothetical protein FLJ40288, isoform CRA_b [Homo sapiens]	gi|119604204	18.690001	LLPREPAAEDLR	9.443542	8.786503
hypothetical protein LOC90768, isoform CRA_a [Homo sapiens]	gi|119625113	17.42	ACLGLPSECGSLVRAR	5.890545	10.03262
hypothetical protein MGC2752 [Homo sapiens]	gi|119593025	6.0100002	SRPLGQSRR	7.550134	7.183501
hypothetical protein MGC45840, isoform CRA_c [Homo sapiens]	gi|119622804	3.0799999	MPAGMFVGVASAQGANPCR	8.774765	5.17173
hypothetical protein, partial [Homo sapiens]	gi|31873746	19.860001	LQETVTMQ	7.287324	6.09989
hypothetical protein, partial [Homo sapiens]	gi|31873949	23.6	EAAGQSYRAR	8.011723	10.16246
Ig H-chain, partial [Homo sapiens]	gi|185315	17.42	GITGTT	5.635132	0
Ig heavy chain variable region, partial [Homo sapiens]	gi|2808901	11.39	KGLLRVPTISSDGSKK	6.764631	4.887347
Ig light chain variable domain, partial [Homo sapiens]	gi|1864117	13.81	AIQMTQSPSSLSASVGDR	8.011727	7.479158
IgG heavy chain variable region, partial [Homo sapiens]	gi|8250279	17.16	GHAMSWVR	7.382752	8.84109
IgGFc-binding protein [Canis lupus familiaris]	gi|545488517	50.490002	YVVPSLSSQSR	12.95851	11.11428
IgGFc-binding protein-like [Canis lupus familiaris]	gi|545488815	10.28	VAGLCGNFNRDPADDVDGPDPI	11.01805	7.009298
IGH@ protein [Homo sapiens]	gi|41351320	48.360001	DASGATFTWTPSSGK	14.07699	11.33383
IgM heavy chain VH1 region precursor, partial [Homo sapiens]	gi|2344972	18.610001	DIGDYTAK	7.879546	7.184582
imidazolonepropionase [Canis lupus familiaris]	gi|545525099	21.66	SGYGLDLQTELK	11.67014	9.28756
immunoglobulin A heavy chain variable region, partial [Homo sapiens]	gi|121944822	23.99	GLEWLGGINYR	6.495596	11.91756
immunoglobulin alpha heavy chain variable region, partial [Homo sapiens]	gi|62871162	11.33	QVQLVQSGSEVKKPGASVR	6.320035	5.852825
immunoglobulin alpha-2 heavy chain, partial [Homo sapiens]	gi|184761	93.139999	QEPSQGTTTFAVTSILR	8.375312	12.92752
immunoglobulin E heavy chain variable region, partial [Homo sapiens]	gi|268375166	16.16	GPRPLTGK	9.758545	7.168901
immunoglobulin epsilon heavy chain variable region, partial [Homo sapiens]	gi|304563542	15.83	GSTQYNPSLDTRVTISVDTSK	4.955398	7.318833
immunoglobulin epsilon variable region, partial [Homo sapiens]	gi|288189027	20.57	DDSKNMLYLHMNR	2.154232	5.873003
immunoglobulin G heavy chain variable region, partial [Homo sapiens]	gi|371446927	51.25	QMHLVESGGDLVKPGGSLR	10.7962	9.336861
immunoglobulin gamma heavy chain variable region, partial [Homo sapiens]	gi|404249070	9.0299997	LTISMDSSK	7.38604	8.361703
immunoglobulin gamma heavy chain, partial [Homo sapiens]	gi|32765851	22.9	GHSGPGLPGQGLLPR	9.513072	9.104384
immunoglobulin heavy chain constant region CH2, partial [Canis lupus familiaris]	gi|124390009	42.529999	QISVSWFR	10.89176	7.794448
immunoglobulin heavy chain variable region 194-104, partial [Homo sapiens]	gi|13123480	11.37	AVPGDSVSNDGVAWNWIRQSP	7.033633	9.297152
immunoglobulin heavy chain variable region VH 3-53 family, partial [Homo sapiens]	gi|31790070	15.09	LSCASSGFTVTLNYMSWVRQAI	7.97157	5.734007
immunoglobulin heavy chain variable region, partial [Homo sapiens]	gi|112694973	19.26	VQCEVQVLASGGGLAQPGGSLF	11.48097	8.389906
immunoglobulin heavy chain VDJC region, partial [Homo sapiens]	gi|553426	23.24	STXGGTAALGCLVK	8.829389	11.97049
immunoglobulin heavy chain, partial [Homo sapiens]	gi|219566253	9.8400002	SPSLESRLTINK	0	5.384861
immunoglobulin heavy variable 3-30-3*01, partial [Homo sapiens]	gi|371570905	15.99	DKSGNTLYLQMNSLR	9.976668	9.637272
immunoglobulin heavy variable 3-49*03, partial [Homo sapiens]	gi|371570901	4.6700001	GLIQPGRSLR	5.699828	10.00435
immunoglobulin kappa chain variable region, partial [Homo sapiens]	gi|19773365	27.870001	VIIYGASTR	8.813227	9.006892
immunoglobulin kappa light chain variable region, partial [Homo sapiens]	gi|98956312	20.790001	ISRVEAEGVGVYYCMQSLQIPR	7.200797	6.082465
immunoglobulin kappa light chain, partial [Homo sapiens]	gi|3169770	55.610001	DSTYSLSSTLTXSK	13.82618	9.165097
immunoglobulin kappa-chain, partial [Homo sapiens]	gi|642375	10.65	ASQSVGR	9.562269	7.581209

immunoglobulin L/VH3-9/N13/DH2-15/N5/JH4 heavy chain variable region, partial [Homo sapiens]	gi|209165484	14.22	SYVVTAEYYFD	4.608572	10.14993
immunoglobulin lambda 1 light chain, partial [Homo sapiens]	gi|170684540	5.04	LAVLG	10.14357	10.43628
immunoglobulin lambda chain variable region, partial [Homo sapiens]	gi|16075980	22.940001	RPXGLSPR	10.06618	9.702919
immunoglobulin lambda light chain variable region, partial [Homo sapiens]	gi|77378301	18.879999	LIIYEVIK	10.40639	10.28772
immunoglobulin lambda-1 variable region, partial [Homo sapiens]	gi|42760073	14.44	DNQRPSGVSDR	8.821983	11.15207
immunoglobulin lambda-like polypeptide 5 isoform 2 [Homo sapiens]	gi|372466586	15.61	ANPTVTLFPPSSEELQANK	8.142119	8.803743
immunoglobulin lambda-like polypeptide 5-like isoform X1 [Canis lupus familiaris]	gi|545544681	56.549999	ADGSPVTQGVETTKPSK	11.23379	9.430575
immunoglobulin lambda-like polypeptide 5-like isoform X3 [Canis lupus familiaris]	gi|545544685	69.610001	ASPSVTLFPPSSEELGANK	13.17257	11.98997
immunoglobulin light chain variable region, partial [Homo sapiens]	gi|11558186	13.43	SGASASLVISGLR	5.918536	5.509534
immunoglobulin mu heavy chain, partial [Homo sapiens]	gi|54780674	14.91	NNSDISSTRGFHQS	5.192325	9.120022
immunoglobulin superfamily member 10 isoform X2 [Canis lupus familiaris]	gi|545539358	10.4	NRMGDDLILMHVSLR	9.399029	7.108035
immunoglobulin variable region VK-NHL112, partial [Homo sapiens]	gi|584461508	6.5999999	AAGIPDR	3.273458	2.521883
immunoglobulin variable region, partial [Homo sapiens]	gi|323432812	19.219999	TVAXPSVFIFPPSDEQLK	7.408424	12.17746
immunoglobulin VH_3c kappa chain, partial [Homo sapiens]	gi|339272251	99.690002	SGTASVLCLLNNFYPR	9.233213	12.51125
importin subunit alpha-8 isoform X1 [Homo sapiens]	gi|578814254	14.92	KDEQTLKRR	11.69344	10.24079
importin-11 isoform 1 [Homo sapiens]	gi|198041777	11.59	KMLALK	4.748554	5.651091
importin-4 [Canis lupus familiaris]	gi|545506927	22.48	AALLLLLTFLAR	9.947374	5.520702
importin-5 isoform X1 [Homo sapiens]	gi|530423358	7.7399998	CIEVMGDGCLNNEHFEELGGILI	6.383379	7.280657
inactive dual specificity phosphatase 27 isoform X1 [Canis lupus familiaris]	gi|73961411	21.32	QQLQVSSLSRAGRRR	11.85239	9.656174
indoleamine 2,3-dioxygenase 2 [Homo sapiens]	gi|148539554	20.629999	HGKPNHLPGPPQALKDR	11.32338	10.98356
inducible nitric oxide synthase [Homo sapiens]	gi|452488	12.99	FHQYAMNGEKGINNNVEK	6.394583	7.901511
inhibitor of growth protein 4 isoform X3 [Homo sapiens]	gi|530398674	8.1999998	MSSARSLSSEEK	6.208957	7.977306
inner nuclear membrane protein Man1 isoform 2 [Homo sapiens]	gi|264681410	13.79	AVDFLAANESRVR	9.620179	9.492881
inorganic pyrophosphatase [Homo sapiens]	gi|11056044	24.42	VCARGEIIGVK	7.67615	13.34519
inositol 1,4,5-trisphosphate receptor type 3 isoform X3 [Homo sapiens]	gi|578811675	2.72	KGSGK	9.972209	0
inositol-trisphosphate 3-kinase B [Canis lupus familiaris]	gi|73961467	15.26	MGKKDPPGGGGGGGGGGGGC	5.584031	4.559216
insulin receptor substrate BAP2	gi|4454524	4.9299998	KDYDTLSK	11.29095	11.9044
insulin-like growth factor-binding protein complex acid labile subunit [Canis lupus familiaris]	gi|545502603	22.040001	MESGPVGLGWAGRPR	12.51409	10.84871
integrin alpha 4 precursor variant, partial [Homo sapiens]	gi|62087350	13.14	IGFLKPHENKTYLAVGSMK	11.05371	6.857292
integrin alpha-10 isoform X11 [Homo sapiens]	gi|578801641	15.89	AAFDGSGQRLSPR	0	5.83835
Integrin alpha-IIb precursor variant, partial [Homo sapiens]	gi|62088684	9.7600002	VVLCELGNPMKK	7.271574	12.15714
integrin, alpha D [Homo sapiens]	gi|119572524	11.12	ASSSFQHEMSQEGFSTALTMVTI	7.674506	8.261556
integrin-linked kinase-associated serine/threonine phosphatase 2C isoform X2 [Canis lupus familiaris]	gi|345790792	15.7	MDLFGDLPEPERSPRR	9.06027	7.504307
interferon alpha-1/2-like [Canis lupus familiaris]	gi|345806671	27.01	RDPPGSPR	12.09564	9.761398
interferon alpha-8 precursor [Homo sapiens]	gi|42476083	21.42	ALILLAQMRR	11.858	6.152078
interferon regulatory factor 6 [Homo sapiens]	gi|343131263	9.8800001	HATRHSPQQEEENTIFK	8.612372	5.170609
interferon-induced protein with tetratricopeptide repeats 3 [Canis lupus familiaris]	gi|545494542	11.18	AKSTEEGK	9.327511	7.60565
interleukin 12 receptor, beta 1, isoform CRA_c, partial [Homo sapiens]	gi|119605061	34.84	SGDGVAEPR	8.563743	11.72337
interleukin 17 receptor, isoform CRA_c [Homo sapiens]	gi|119578144	24.610001	GAEGVCGIQNGSLRWAEVKR	7.339526	13.46743
Interleukin 27 [Homo sapiens]	gi|38566147	16.73	LLAEVR	7.873831	7.908679
interleukin-10 receptor subunit alpha isoform X1 [Homo sapiens]	gi|578822174	8.9799995	CLVDEAGLHPPALAKGYLK	7.414092	9.524027
interleukin-12 receptor subunit beta-2 isoform X3 [Homo sapiens]	gi|578799050	15.84	EKGIQGHQASEK	7.551229	8.308859
interleukin-18 receptor accessory protein isoform X1 [Homo sapiens]	gi|530368833	7.6300001	TETTGR	7.279478	10.36476
intermediate conductance calcium-activated potassium channel protein 4 isoform X2 [Homo sapiens]	gi|530416474	13.42	AGPAVR	9.142015	9.342375
interstitial collagenase [Canis lupus familiaris]	gi|345800008	11.83	QRNNSLVSEK	7.286867	8.156637
intracellular hyaluronan-binding protein 4 isoform X1 [Homo sapiens]	gi|530390749	3.6700001	AVVIHK	5.226727	0
intraflagellar transport protein 122 homolog isoform X4 [Canis lupus familiaris]	gi|545532616	9.8199997	DMMLDKFR	9.152088	9.706137
intraflagellar transport protein 140 homolog isoform X10 [Homo sapiens]	gi|578828383	18.15	KEPEIMK	0	7.050363
intraflagellar transport protein 172 homolog isoform X3 [Homo sapiens]	gi|578802823	3.8299999	KVICNK	4.463316	4.858567
IQ domain-containing protein H isoform 4 [Homo sapiens]	gi|547235270	7.4400001	KTKCMSALSMPMLATSR	9.46803	6.910401
iron-sulfur cluster assembly enzyme ISCU, mitochondrial isoform X1 [Homo sapiens]	gi|530400015	1.7	AAAGAFR	7.74798	6.864089
iroquois-class homeodomain protein IRX-5 isoform X2 [Canis lupus familiaris]	gi|545491132	14.69	KGHPTLLE	8.861404	0
JmjC domain-containing histone demethylation protein 2C isoform X5 [Canis lupus familiaris]	gi|545495008	5.6700001	MATAVETR	9.417315	7.853185
jouberin isoform X7 [Canis lupus familiaris]	gi|545485426	7.3099999	KGSPVR	6.600658	6.641832
JQ1035 hypothetical 3.2K protein (type I IGFR 5' region) - human	gi|106318	5.3699999	MEADDADSPPR	8.628698	10.38891
junction plakoglobin isoform X1 [Homo sapiens]	gi|578830876	6.0300002	DPMNR	9.361956	9.581933
junctophilin 1, isoform CRA_e [Homo sapiens]	gi|119607436	15.43	EANSGAGETTARGMR	7.539405	7.50565
kalirin isoform X10 [Canis lupus familiaris]	gi|545553113	14.56	DPVGCLNEGMTPPTPPR	9.857014	9.729979
kallikrein-1 isoform X1 [Homo sapiens]	gi|530416509	36.110001	LTEPADTITDAVK	9.054893	9.866377
kallikrein-1 precursor [Canis lupus familiaris]	gi|55741639	35.48	YNLFEHEDTAQFVQVR	8.914849	7.968486
kelch repeat and BTB domain-containing protein 13 [Canis lupus familiaris]	gi|545550208	12.57	LGALSAGGFRTTLQVLR	11.98992	9.256748
kelch-like protein 41 [Canis lupus familiaris]	gi|57110617	15.35	KGDWKDLAPMKTPR	6.842485	10.55236
keratin, type I cytoskeletal 9 [Homo sapiens]	gi|55956899	88.370003	GGSGGSYGGGGSGGGYGGGSG	8.050247	9.864631
keratin, type II cytoskeletal 1 [Homo sapiens]	gi|119395750	48.279999	SLDLDSIIAEVK	4.66882	7.266319
keratin, type II cytoskeletal 1b isoform X1 [Homo sapiens]	gi|530400209	1.1799999	NSKMEIAELNRTVQR	7.880607	8.665694
keratin, type II cytoskeletal 2 epidermal [Homo sapiens]	gi|47132620	21.74	LNDLEEALQQAK	8.743817	9.185357
keratin, type II cytoskeletal 3-like [Canis lupus familiaris]	gi|545547076	46.740002	CLDLDSIIAEVR	8.872511	11.61275
keratin, type II cytoskeletal 4 [Homo sapiens]	gi|331999954	27.879999	VDSLNDEINFLK	10.70391	9.175653
keratin, type II cytoskeletal 6C [Homo sapiens]	gi|5031839	36.759998	ADTLTDEINFLR	9.131142	10.29168
keratin, type II cytoskeletal 72 isoform 2 [Homo sapiens]	gi|226246665	49.450001	DLDLDSIIAEVR	8.479163	10.31177
keratin, type II cytoskeletal 79 [Homo sapiens]	gi|32567786	25.51	NLDLDSIIAEVK	8.362372	9.269594
K-glypican precursor [Homo sapiens]	gi|6652549	19.67	SCSXVRR	5.559922	8.141144

KHDC3-like protein isoform X1 [Homo sapiens]	gi|578812433	16.66	MDAPRR	6.623564	7.596677
KIAA0226-like ortholog, partial [Canis lupus familiaris]	gi|545537990	16.280001	ILSTWDFR	11.01222	7.814908
KIAA0232 [Homo sapiens]	gi|48146437	13.35	ISVFLKK	8.572347	6.777903
KIAA0288 protein, partial [Homo sapiens]	gi|6635127	20.25	AISNHLK	9.456244	7.460025
KIAA0446 protein, partial [Homo sapiens]	gi|58257725	10.87	TGGCGLEVR	9.983419	9.508792
KIAA0556 protein [Homo sapiens]	gi|119576147	11.71	LSSQGNVSGKRKNSTNCR	7.721397	5.288921
KIAA0921 protein, partial [Homo sapiens]	gi|20521694	13.39	GPGGAGAAVGMASGSR	9.024211	9.553451
KIAA0995 protein, partial [Homo sapiens]	gi|4589634	4.1999998	QDGGGSQK	11.07019	4.936292
KIAA1184 protein, partial [Homo sapiens]	gi|6330255	15.67	AATALKGRGARNAR	9.972533	9.22504
KIAA1186 protein, partial [Homo sapiens]	gi|58257684	24.120001	ATMSGRVGDLSPR	7.543788	11.72805
KIAA1299 protein, partial [Homo sapiens]	gi|7242953	14.69	LWLGVGCSLR	10.03201	10.68502
KIAA1430 protein [Homo sapiens]	gi|116283624	14.34	IDKDTKK	9.170331	6.290767
KIAA1809 protein, partial [Homo sapiens]	gi|14017835	6.8299999	AVVRPPPPPPPPPAPQPTMSRR	9.024529	8.454673
kielin/chordin-like protein isoform 1 precursor [Homo sapiens]	gi|209571519	4.6900001	SSPFSR	6.63817	10.06764
kinesin family member 20B [Canis lupus familiaris]	gi|545547379	20.92	KKWLEEKMMLIIQAK	8.990884	9.055482
kinesin-like protein KIF16B isoform X8 [Homo sapiens]	gi|530425802	25.4	SLGANPDDLKDPIK	8.962505	10.24667
kinesin-like protein KIF20B isoform 1 [Homo sapiens]	gi|546231677	24.209999	ETGNINTSLLTLGK	7.269083	7.409537
kinesin-like protein KIF23 isoform X7 [Homo sapiens]	gi|578827638	10.72	NHNMYVAGCTEVEVK	10.61716	9.890998
kinesin-like protein KIF26B [Homo sapiens]	gi|124430752	0.1	AAPIK	9.328606	9.59411
kinesin-like protein KIF2A isoform 1 [Homo sapiens]	gi|148612877	8.3900003	RTVASIKNDPPSRDNR	9.625248	7.390875
kinesin-like protein KIF2C isoform X3 [Homo sapiens]	gi|530361649	18.4	KAQNSEMR	0	7.676169
kinetochore protein Spc24 isoform X2 [Canis lupus familiaris]	gi|545534660	2.6500001	PGGSK	9.464694	11.48614
kinetochore-associated protein 1 isoform X1 [Homo sapiens]	gi|578824490	18.719999	ANDENRR	9.370954	11.03779
kininogen-1 isoform X1 [Canis lupus familiaris]	gi|345796419	17.41	ENYLFLTPDCK	9.415711	10.38424
KN motif and ankyrin repeat domain-containing protein 1 isoform X16 [Homo sapiens]	gi|578816444	14.62	QTSTQTVETRTVAVGEGR	10.37066	9.976047
krev interaction trapped protein 1 isoform X15 [Homo sapiens]	gi|578814707	8.0100002	VIPVYVGVNIKGLHLLNMETK	3.794385	7.037451
L Chain L, Structure Of Reovirus Sigma1 In Complex With Its Receptor Junctional Adhesion Molecule-A	gi|211939412	9.6400003	VKLIVL	9.765276	8.82985
lactation elevated protein 1 isoform X1 [Homo sapiens]	gi|530383503	10.3	TMVMDMFYAYVEMK	4.695522	8.564983
lactoferrin [Homo sapiens]	gi|27438055	18.200001	FQLFGSPSGQK	0	5.899138
lactoperoxidase [Canis lupus familiaris]	gi|345805633	44.889999	FWWENPGVFTEK	8.313517	9.617686
lactoperoxidase isoform 3 preproprotein [Homo sapiens]	gi|231569458	43.32	NGFPLPLAR	9.236872	7.577475
laminin subunit alpha-1 [Canis lupus familiaris]	gi|545506399	10.18	MTERILK	7.701453	8.385277
laminin subunit alpha-4 [Canis lupus familiaris]	gi|545520371	14.51	RVDGALARKSALR	10.97508	8.575265
laminin subunit alpha-5-like, partial [Canis lupus familiaris]	gi|545560951	12.16	NQERLEDALRRR	9.912427	9.309738
laminin, alpha 4, isoform CRA_a, partial [Homo sapiens]	gi|119568651	7.52	EARAPT	10.33877	11.7778
laminin-5 gamma 2 [Canis lupus familiaris]	gi|17998675	21.629999	TLPCGITK	8.968228	9.525259
L-amino-acid oxidase-like [Canis lupus familiaris]	gi|545524998	9.6899996	RVIVVGAGMSGLAAAK	6.230475	4.104725
lanC-like protein 1 isoform X1 [Homo sapiens]	gi|530369695	18.860001	GLKSADPR	10.53327	11.32891
large neutral amino acids transporter small subunit 4 isoform X3 [Homo sapiens]	gi|578829698	21.15	LSVGSSMR	10.26871	10.26604
latent-transforming growth factor beta-binding protein 2 [Canis lupus familiaris]	gi|545508063	15.04	EQDAPVPGLPPTER	5.504041	7.210049
lebercilin isoform X1 [Homo sapiens]	gi|530383030	26.76	LQGEEEERLKREMLLAK	7.639843	3.612801
leiomodin-2 [Canis lupus familiaris]	gi|359321176	7.6599998	KQPNNILKEIKNSLR	8.735965	0
LEM domain-containing protein 1 isoform X1 [Homo sapiens]	gi|578801733	9.4700003	KAVDTYCLDYKPSKGR	8.074434	7.249277
LEM domain-containing protein 2 isoform 1 [Homo sapiens]	gi|31044432	13.24	ARPAAASPR	8.598461	9.056732
leucine zipper protein 4 [Canis lupus familiaris]	gi|545560124	15.12	GLLANXR	9.311789	8.975554
leucine zipper protein 4 isoform X1 [Homo sapiens]	gi|530422291	1.7	NTITT	6.086425	7.121086
leucine zipper transcription factor-like protein 1 [Canis lupus familiaris]	gi|73985994	14.18	QLFSQAEK	6.468669	10.65783
leucine-rich repeat-containing protein 16A isoform X4 [Homo sapiens]	gi|578811788	15.8	YGVQVMGSGLLAEMKAKQEK	7.175752	7.820549
leucine-rich repeat-containing protein 16A isoform X6 [Homo sapiens]	gi|578811792	14.14	NNITAQGFQDIAVAMEK	9.076254	5.166991
leucine-rich repeat-containing protein 29 [Canis lupus familiaris]	gi|345800999	7.6999998	ALGSGRR	6.979372	7.959857
leucine-rich repeat-containing protein 41 isoform X1 [Homo sapiens]	gi|530361606	11.65	AAGKKGARTR	6.726985	7.118426
leucine-rich repeat-containing protein 45 [Canis lupus familiaris]	gi|73964715	10.21	TQVLSK	9.802569	9.468712
leucine-rich repeat-containing protein 58 [Homo sapiens]	gi|153792305	8.9700003	MQKVLLG	9.820108	9.677303
leukocyte elastase inhibitor [Canis lupus familiaris]	gi|545554777	47.68	TYNFLPEFLASTQK	7.404015	7.178727
leukocyte immunoglobulin-like receptor, subfamily B (with TM and ITIM domains), member 5, isoform CRA_a [Homo sapiens]	gi|119592624	23.57	GKSLTGNSLPGLSVGPR	8.852496	10.0098
LIM and calponin homology domains-containing protein 1 isoform X10 [Homo sapiens]	gi|578808499	7.1399999	EHPSSDGAVVAPAPKSEEKDAA	6.566144	7.750498
LIM domain-binding protein 1 isoform X1 [Homo sapiens]	gi|530394577	10.53	RKMSGGSTMSSGGGNTNNSNSI	6.581255	4.307848
LIM domain-binding protein 2 isoform X9 [Homo sapiens]	gi|578808437	19.42	SPVSGCALR	0	8.29304
lipase maturation factor 1 isoform X4 [Homo sapiens]	gi|578828214	13.68	IMLGAGLIKIR	8.228707	8.420665
liprin-beta-2 isoform 2 [Homo sapiens]	gi|375151573	11.31	MSSEQWPR	9.655058	9.701987
LMBR1 domain containing 1, isoform CRA_b [Homo sapiens]	gi|119569220	12.93	KNQNGTFK	7.94936	4.303093
LOC100132686 protein [Homo sapiens]	gi|21595277	6.8899999	KAIEKM	4.915432	3.621178
LOC55908 protein, partial [Homo sapiens]	gi|89243638	30.309999	AAAVGSTGER	7.852687	7.638163
LOC646938 protein, partial [Homo sapiens]	gi|121934142	10.89	WPALMGTGR	9.405845	6.737995
lon protease homolog, mitochondrial isoform 3 [Homo sapiens]	gi|451327636	13.38	LKELVVPK	7.816655	6.901474
long-chain-fatty-acid--CoA ligase 4 isoform X2 [Homo sapiens]	gi|578838329	10.33	KGSKGDCTVLKPTLMAAVPEIM	7.84484	7.06856
long-chain-fatty-acid--CoA ligase ACSBG2 isoform b [Homo sapiens]	gi|574584773	15.54	ASTVTEIVK	6.643591	10.69024
loss of heterozygosity 12 chromosomal region 1 protein isoform X1 [Homo sapiens]	gi|530398989	3.48	TPSPAK	8.416651	11.75263
low-density lipoprotein receptor-related protein 4 isoform X2 [Homo sapiens]	gi|578820535	14.48	IEVANTDGSMR	7.820728	9.748698
LPS-responsive vesicle trafficking, beach and anchor containing isoform X7 [Canis lupus familiaris]	gi|545524786	8.5500002	GLDGPRPNQKEILSLRAFLLMFH	7.432108	8.838064
lymphocyte antigen 6H isoform a precursor [Homo sapiens]	gi|208431838	12.05	LPAAMK	11.60181	11.01699
lysine-specific demethylase 4C isoform X2 [Canis lupus familiaris]	gi|545517031	9.6300001	EPDPPK	7.740005	9.323551

lysine-specific histone demethylase 1A isoform X4 [Homo sapiens]	gi|578798683	8.4300003	QATPGVPAQQSPSM	0	5.94635
lysophosphatidic acid receptor 3 [Canis lupus familiaris]	gi|545503452	12.43	KMICCFSQEK	6.88552	5.336967
lysosomal alpha-mannosidase isoform X1 [Homo sapiens]	gi|530414862	16.57	ANLTWSVK	8.431852	7.753128
lysosomal beta-galactosidase [Canis lupus familiaris]	gi|76470548	13.33	MARPAAVR	10.55254	11.17358
lysosomal-trafficking regulator isoform X5 [Homo sapiens]	gi|578802129	4.71	EIFTYLVEGFK	8.81347	6.96046
lysyl oxidase homolog 1 [Canis lupus familiaris]	gi|545550718	16.99	MGAGAGAGAGAGGGLR	9.58906	9.512841
M Chain M, 2me Modified Human Sod1	gi|399124886	18.620001	DGVADVSIEDSVISLSGDHXIIGI	11.62879	6.45567
m7GpppN-mRNA hydrolase isoform 1 [Homo sapiens]	gi|31542498	10.82	HRQPLQQKPYNNHSEMSDLLK	13.16538	10.95199
macrophage receptor MARCO [Homo sapiens]	gi|5803080	9.1499996	GAMGMPGAPGPPGPPAEK	10.09323	6.621487
maestro heat-like repeat family member 5 isoform X3 [Canis lupus familiaris]	gi|545521024	20.98	WASSTILLSYGQMAVR	12.12123	8.50191
maestro heat-like repeat-containing protein family member 1-like isoform X9 [Canis lupus familiaris]	gi|545546817	8.4799995	GEDIKVVFCSISK	5.692496	11.24526
maestro heat-like repeat-containing protein family member 6 isoform X10 [Homo sapiens]	gi|578839730	11.89	GASGPEPQALAATR	9.220525	10.05755
magnesium transporter NIPA4 [Canis lupus familiaris]	gi|545495952	4.5700002	KKGLQR	3.183318	10.26555
MAGUK p55 subfamily member 4 [Canis lupus familiaris]	gi|545556430	9.0799999	AAEVCK	6.260068	7.714651
MAGUK p55 subfamily member 6 isoform X1 [Homo sapiens]	gi|578813535	10.81	EGGSAGLIPSQFLEEK	7.741416	10.9738
major facilitator superfamily domain-containing protein 2B [Canis lupus familiaris]	gi|545527133	17	ERPDSSAPASGQGLSFLAGLGLT	9.215325	7.276206
major histocompatibility complex, class II, DR beta 5 precursor [Homo sapiens]	gi|18641375	11.43	MVCLKLPGGSYMAK	9.642996	8.204176
male-specific lethal 3 homolog isoform a [Homo sapiens]	gi|212275945	6.9699998	MSASEGMK	10.47353	9.545539
MAM and LDL-receptor class A domain-containing protein 1 precursor [Homo sapiens]	gi|565671710	19.889999	SMGILK	6.092792	4.636738
MAM domain-containing protein 2 isoform X1 [Canis lupus familiaris]	gi|73946831	4.8299999	GVTGK	9.600745	9.304073
mannan-binding lectin serine protease 1 isoform X2 [Canis lupus familiaris]	gi|545553781	6.02	RRSDGNEQHLRVK	7.732864	8.279806
mannose-P-dolichol utilization defect 1 protein isoform X3 [Homo sapiens]	gi|578830104	2.73	KPEIP	0	8.364367
mannosyl-oligosaccharide 1,2-alpha-mannosidase IA [Canis lupus familiaris]	gi|345784526	6.73	SILGHKRMSARR	9.081303	8.105659
MAP kinase-activating death domain protein isoform X31 [Homo sapiens]	gi|578820904	13.82	KLGIPR	8.890832	8.463473
MAP3K12-binding inhibitory protein 1 isoform 2 [Homo sapiens]	gi|222080055	10.27	RKVQPPQNYSLAELDEKISALK	6.553294	7.191903
MAP7 domain-containing protein 3 isoform X7 [Canis lupus familiaris]	gi|545559505	13.62	ERMVIDGSVLKNDVKQR	7.858404	6.703434
MAP9 protein, partial [Homo sapiens]	gi|124504595	6.8800001	EAKKIAA	5.596598	8.419554
MASP1 protein, partial [Homo sapiens]	gi|24981014	10.95	EGADLFGR	8.365315	10.47106
matrix metalloproteinase 3, partial [Homo sapiens]	gi|90287774	21.129999	LDSDT	6.838824	7.28519
matrix-remodeling-associated protein 7 [Canis lupus familiaris]	gi|545509641	12.02	GGGGGAGRARRR	12.11916	9.003059
MAX dimerization protein 4, isoform CRA_e [Homo sapiens]	gi|119602941	14.65	RAEPAGAGRAAAAAVPGR	5.900611	5.901899
MAX gene-associated protein isoform X5 [Canis lupus familiaris]	gi|545549540	16.139999	IPDSSKSSAGDSFLGKEDLGR	8.792417	5.569927
max-interacting protein 1 isoform a [Homo sapiens]	gi|57242782	9.1300001	MERVKMINVQR	6.407922	5.311435
mediator of RNA polymerase II transcription subunit 13-like isoform X2 [Homo sapiens]	gi|578823479	4.6799998	AIAPSR	10.3503	7.888855
megakaryocyte-associated tyrosine-protein kinase isoform X2 [Canis lupus familiaris]	gi|545535223	18.559999	KQGTRSAEEELAK	8.191316	8.334803
meiosis arrest female protein 1 isoform X7 [Homo sapiens]	gi|578840065	16.629999	FEEFISDLPPR	10.67813	8.208329
melanocortin-2 receptor accessory protein isoform c [Homo sapiens]	gi|549806740	8.8900003	MSWSASPQMR	8.51256	7.649363
melanoma inhibitory activity protein 2 precursor [Homo sapiens]	gi|46094009	14.55	SSYSLSDMVSNIELPTR	7.329298	10.35565
melanoma inhibitory activity protein 3 isoform X2 [Homo sapiens]	gi|578800895	8.0699997	AAGNQMNDRK	7.438051	9.005928
melanoma-associated antigen B1 [Homo sapiens]	gi|29171711	3.6300001	EPIMK	10.30044	11.18954
membrane-associated guanylate kinase, WW and PDZ domain-containing protein 2 isoform X1 [Homo sapiens]	gi|530386693	5.7399998	DVLAVIKHCKDPLRLK	8.432658	7.212386
mesoderm posterior protein 1 [Homo sapiens]	gi|14149724	10.13	GDAGSPR	6.698406	8.482805
metallothionein 1B (functional), partial [Homo sapiens]	gi|119603286	22.5	ATSTNLDFFF	5.629381	7.424507
metastasis-associated in colon cancer protein 1 isoform X1 [Canis lupus familiaris]	gi|545522639	6.46	SAPLLPK	7.202953	6.223764
methionyl-tRNA formyltransferase, mitochondrial isoform X1 [Homo sapiens]	gi|530405381	10.61	QSQILLVYCKDGWIGVRSVMLK	11.22155	4.592778
methyl-CpG-binding domain protein 3-like 5 [Canis lupus familiaris]	gi|545534946	13.55	KRQVHLAK	7.514096	5.405846
methyl-CpG-binding protein 2 isoform X1 [Homo sapiens]	gi|530422797	7.29	MVAGMLGLR	9.347273	8.068289
methylthioribose-1-phosphate isomerase [Canis lupus familiaris]	gi|359322226	3.04	AAPGGGK	7.060994	7.926651
methyltransferase TARBP1 isoform X5 [Homo sapiens]	gi|578802318	19.209999	KMTSR	11.07545	8.109976
methyltransferase-like protein 8 isoform X2 [Canis lupus familiaris]	gi|545555183	14.23	LHSSYR	0	7.399732
MGN3_HUMAN RecName: Full=protein mago nashi homolog 3	gi|205810123	9.7700005	KEAYVHKSLMEELK	9.329284	5.611165
MHC class I antigen, partial [Homo sapiens]	gi|4378522	10.37	AVMAPRTLVLLLSGALALTQTW	5.799252	4.304173
MHC class II antigen, partial [Homo sapiens]	gi|3320136	16.85	AITPWCRAP	9.381946	4.139297
MHC HLA-SX-alpha, partial [Homo sapiens]	gi|386952	7.9899998	RSXQHPRVQGLL	6.148387	8.635506
MICAL-like protein 2 isoform X1 [Homo sapiens]	gi|578813627	18.74	ALAEPRAGEAPR	6.957334	7.416631
microcephalin, partial [Homo sapiens]	gi|46358767	11.54	GTLFADQPXMFVSPASSPPVAK	8.990965	8.175637
microtubule-actin cross-linking factor 1 isoform X29 [Homo sapiens]	gi|578798838	15.18	NIEPTHAPFIEKSR	5.843664	9.853815
microtubule-associated protein 2 isoform X7 [Canis lupus familiaris]	gi|545556050	12.83	HSAGGGNVSR	5.204757	6.188562
midasin [Canis lupus familiaris]	gi|545519916	16.290001	QWFELVAASDHL	13.60283	10.86824
midasin isoform X3 [Homo sapiens]	gi|578812559	20.139999	RPTLEGR	9.797832	7.130838
midnolin isoform X2 [Homo sapiens]	gi|530425485	7.1999998	AGGGGFR	8.008775	9.499216
MIG7 [Homo sapiens]	gi|102863546	11.52	KTDRGERGUCVSTTK	6.502363	7.224863
mitochondrial 10-formyltetrahydrofolate dehydrogenase precursor [Homo sapiens]	gi|238814322	10.35	EESFGPIMVISK	6.758795	0
mitochondrial chaperone BCS1 isoform X1 [Homo sapiens]	gi|578804710	16.620001	GIPYRR	1.634583	7.341162
mitochondrial intermediate peptidase, partial [Canis lupus familiaris]	gi|545542014	9.1199999	EMLAHGGGK	4.74375	5.754751
mitochondrial ribonuclease P protein 1 isoform X2 [Canis lupus familiaris]	gi|545552673	19.91	NLTLDQMMRILLCLK	9.91444	9.159417
mitochondrial ribonuclease P protein 3 [Canis lupus familiaris]	gi|545507127	6.4899998	MMTFCFTGIR	6.324375	8.351433
mitochondrial ribosomal protein S9, isoform CRA_c, partial [Homo sapiens]	gi|119622174	6.5100002	LLNLEK	8.650172	8.682488
mitofusin 1, isoform CRA_e [Homo sapiens]	gi|119598818	13.51	IYKSLPR	8.806623	8.001677
mitofusin-2 isoform X1 [Homo sapiens]	gi|530360745	8.7200003	MSLLFSRCNSIVTVK	10.23977	10.49863
mitogen-activated protein kinase 8 isoform X3 [Homo sapiens]	gi|578819637	14.16	EVMDLEER	10.74008	10.12904
mitogen-activated protein kinase kinase kinase 15 [Canis lupus familiaris]	gi|345806826	6.1700001	ESGGGAPPAGALGAAAESVR	9.041015	9.573878
mitogen-activated protein kinase kinase kinase 6 [Canis lupus familiaris]	gi|545492222	20.879999	SRSPGSPR	9.084064	0

mitogen-activated protein kinase kinase kinase kinase 4 isoform X20 [Homo sapiens]	gi|578805234	16.82	MLQALQLR	8.624534	9.505912
mitotic spindle assembly checkpoint protein MAD1 isoform X1 [Canis lupus familiaris]	gi|345801374	5.04	QQLQEEVR	9.751994	8.536591
moesin isoform X2 [Homo sapiens]	gi|530421753	10.88	KTANDMIHAENMR	9.86694	11.03889
molybdenum cofactor sulfurase isoform X2 [Canis lupus familiaris]	gi|545505863	13.03	KTYFGGGTAAAYLAGEDFYIPR	9.1706	8.634424
mono-ADP-ribosyltransferase 3 [Homo sapiens]	gi|48525610	0.61	KTRSLKTMV	8.017282	5.850946
monocarboxylate transporter 7 isoform X2 [Homo sapiens]	gi|578831622	8.6099997	AFYSCAAGMALAAVCLALVRP(	9.193033	5.841489
mothers against decapentaplegic homolog 1 isoform X1 [Homo sapiens]	gi|578809121	3.6500001	KGAMEELEKALSCPGQPSNCVT	9.559896	4.414095
MRDS1 protein [Homo sapiens]	gi|22770616	13.1	EGSIPK	0	3.536848
msx2-interacting protein [Homo sapiens]	gi|14790190	15.99	KIGGNK	6.311049	7.900648
MTG8-related protein 2, partial [Homo sapiens]	gi|2967833	14.9	SLTVVR	10.58058	8.691435
MUCB_HUMAN RecName: Full=Ig mu heavy chain disease protein	gi|127506	21.65	QIEVSWLR	12.31704	10.10288
mucin-16 [Canis lupus familiaris]	gi|545535651	10.52	VALGLAGISMDPK	7.018259	8.793417
mucin-19-like [Canis lupus familiaris]	gi|545547107	19.42	DCLCTIFGNYVK	10.63257	7.677674
mucin-5AC [Canis lupus familiaris]	gi|545531528	65.559998	ALSGVVEGTAAAFANTWK	8.734108	8.182912
mucin-7-like [Canis lupus familiaris]	gi|545560548	47.900002	NNVQQYTVDR	11.25891	9.926558
muellerian-inhibiting factor precursor [Homo sapiens]	gi|157266298	8.9499998	NPRYGNHVVLLLKMQAR	6.091302	10.65508
multidrug resistance protein 1 [Canis lupus familiaris]	gi|545522646	3.6800001	AANIHSFIEDLPK	5.099253	4.835429
Multidrug resistance protein 1 variant, partial [Homo sapiens]	gi|62087520	10.2	GAIGSR	9.626546	9.488186
multidrug resistance protein 3 isoform X4 [Canis lupus familiaris]	gi|545522239	15.86	MDLEAGR	8.414763	12.6445
multidrug resistance-associated protein 4 isoform X1 [Canis lupus familiaris]	gi|545537293	8.71	EGKMVQK	7.991978	7.387451
multidrug resistance-associated protein 5 isoform X1 [Homo sapiens]	gi|530373795	14.14	AEGLSLDASMHSQLR	6.200748	7.60009
multidrug resistance-associated protein 7 isoform X7 [Homo sapiens]	gi|578812050	17.35	ADAVLLMEAGR	6.511395	6.039957
multidrug resistance-related protein, isoform CRA_a [Homo sapiens]	gi|119592315	15.09	WISGLPR	9.755282	8.161138
multimerin-2 isoform X1 [Homo sapiens]	gi|578819775	13.83	ARAATSR	8.208286	7.067598
multiple myeloma tumor-associated protein 2 [Canis lupus familiaris]	gi|73975460	7.7800002	KDKAHRSR	9.788903	9.198125
musculin [Homo sapiens]	gi|116805332	5.0900002	KPLPAK	8.177192	8.832329
myelin regulatory factor-like protein [Canis lupus familiaris]	gi|545514199	13.63	FTLGNLCFHGQRGAR	13.67172	9.210065
myeloperoxidase, isoform CRA_b [Homo sapiens]	gi|119614877	16.91	WGTRHCPALGK	2.466876	7.649912
myomesin-1 isoform a [Homo sapiens]	gi|140560919	8.1000004	MAALESLK	9.029105	9.230065
myomesin-3 [Homo sapiens]	gi|155969693	19.84	QTSAVELEER	5.822983	11.90451
myosin-15 [Canis lupus familiaris]	gi|545552775	13.57	KAATEAANVAEELKK	7.414551	7.310352
myosin-15 precursor [Homo sapiens]	gi|150010558	18.57	ALDAKLSR	9.350615	8.889556
myosin-4 isoform X1 [Homo sapiens]	gi|530410339	15.26	QQLNEKLKKK	9.081515	7.845564
myosin-6 isoform X4 [Homo sapiens]	gi|578825824	16.35	LTQESIMDLENDK	11.69536	10.65251
myotubularin-related protein 10 isoform X3 [Canis lupus familiaris]	gi|545493194	14.04	SISGTPLSKFLSGAK	7.520732	0
N(G),N(G)-dimethylarginine dimethylaminohydrolase 1 isoform X6 [Homo sapiens]	gi|578798850	7.0500002	MVDMMK	9.525119	7.360625
Na(+)/H(+) exchange regulatory cofactor NHE-RF2 isoform X1 [Canis lupus familiaris]	gi|545502654	7.6399999	DVGGPPR	4.92411	7.553907
N-acetylated-alpha-linked acidic dipeptidase 2 isoform X3 [Canis lupus familiaris]	gi|545536024	6.5500002	DPSSENTNFPR	5.253235	7.977749
N-acetylated-alpha-linked acidic dipeptidase isoform X1, partial [Homo sapiens]	gi|578822016	8.8100004	MMNDQLMFLER	4.967588	8.967006
N-acetyl-D-glucosamine kinase isoform X1 [Homo sapiens]	gi|530367804	5.1199999	KAGVDPLVPLRSLGLSLSGGDQI	7.357635	6.239855
N-acetyllactosaminide alpha-1,3-galactosyltransferase isoform X3 [Canis lupus familiaris]	gi|545512408	12.01	EADDEEKIMNVKGK	8.828266	8.915752
N-acetyltransferase 8B isoform X3 [Canis lupus familiaris]	gi|545528209	10.12	MAPYHIRKYQERDRPR	6.633216	10.12499
N-acetyltransferase ESCO1 isoform X4 [Canis lupus familiaris]	gi|545506264	4.2800002	AASSDK	5.914617	9.978014
NACHT, LRR and PYD domains-containing protein 4 isoform X4 [Homo sapiens]	gi|578834018	7.1399999	KDLCMK	10.8091	9.30906
NADH dehydrogenase [ubiquinone] 1 alpha subcomplex assembly factor 5 isoform X2 [Homo sapiens]	gi|578835541	7.3600001	RPWAARVPAENLGRREVTSGV!	7.269555	6.999101
NADH dehydrogenase [ubiquinone] 1 beta subcomplex subunit 3 [Canis lupus familiaris]	gi|545555828	11.38	YAGGFANNVSFVGALLK	11.94144	10.89456
NADH dehydrogenase subunit 4 (mitochondrion) [Homo sapiens]	gi|545770183	10.64	LGGXGMMR	11.24796	8.121742
NADPH oxidase 1 isoform 3 [Homo sapiens]	gi|425854820	16.35	VQFYFNK	8.519134	8.341523
NADPH--cytochrome P450 reductase [Homo sapiens]	gi|127139033	12.47	LIEGGAHIYVCGDAR	8.156591	6.463743
nasal embryonic LHRH factor variant 4 [Homo sapiens]	gi|32478640	9.5	VMVRPSPGSAPLHPEQDSAPTR	8.030636	7.211262
natural resistance-associated macrophage protein 1, partial [Homo sapiens]	gi|21739135	23.610001	IPIPDTKP	7.521296	8.46538
nebulin isoform X18 [Homo sapiens]	gi|578804369	9.2200003	MVGFRSLEDDPK	10.08852	9.718697
nebulin-related-anchoring protein isoform X2 [Homo sapiens]	gi|578819506	11.34	GKAMGTADSR	9.107162	7.710284
NEDD4 binding protein 2-like 1 [Canis lupus familiaris]	gi|545542132	16.059999	RNIHGVPR	8.815413	9.027326
NEDD4-like E3 ubiquitin-protein ligase WWP1 isoform X2 [Homo sapiens]	gi|530388695	9.79	ADALLGK	5.103145	5.115468
NEDD8-activating enzyme E1 regulatory subunit [Canis lupus familiaris]	gi|359319592	13.42	GTIPDMIADSSK	8.835585	8.605809
NEDD8-activating enzyme E1 regulatory subunit isoform X1 [Homo sapiens]	gi|530424459	18.24	ALKEFVAK	0	10.32912
negative elongation factor E isoform X1 [Homo sapiens]	gi|578842391	0.86	MLVIPPGLSEEEEALQK	8.504142	8.032969
nephrocystin-4 isoform X15 [Homo sapiens]	gi|578798919	11.19	GPQDVSR	3.621735	7.406237
nesprin-1 [Canis lupus familiaris]	gi|545485667	3.7	MSTIKMK	7.756065	7.971046
nesprin-2 isoform X7 [Homo sapiens]	gi|530403364	10.52	QFRAVR	3.30997	2.297957
nesprin-3 isoform X6 [Homo sapiens]	gi|578825565	12.88	KLLDLQVR	7.598905	10.12162
neugrin precursor [Homo sapiens]	gi|75677385	12.86	QKQAIRFQK	14.34587	10.90825
neurabin-2 [Homo sapiens]	gi|140972063	9.2799997	EEMDKLLDKISELEGNLQTLR	9.426294	5.955542
neuregulin 1 isoform 4, partial [Homo sapiens]	gi|28883520	12.68	CVNGGECFMVK	8.557261	8.215548
neurobeachin-like 1 isoform X2 [Canis lupus familiaris]	gi|545555920	7.8400002	AIVPK	7.982841	9.128747
neuroblast differentiation-associated protein AHNAK isoform X10 [Homo sapiens]	gi|578821732	17.52	ISMQDVDLSLGSPKLK	12.66511	10.28174
neuroblastoma breakpoint family member 10, partial [Homo sapiens]	gi|385139449	6.5500002	QAEELG	7.411877	8.738033
neuroblastoma breakpoint family member 20 isoform X11 [Homo sapiens]	gi|578842723	4.6799998	SSAPR	7.121814	9.004488
neuroblastoma-amplified sequence [Homo sapiens]	gi|41393547	18.450001	LATVMLTR	5.951152	9.547279
neurolysin, mitochondrial isoform X2 [Homo sapiens]	gi|578810467	11.38	KEGIMNPEVGMK	6.098081	7.264217
neurolysin, mitochondrial isoform X3 [Canis lupus familiaris]	gi|545490837	4.4400001	GIMNPEVGMKYR	4.950469	9.299114
neuron navigator 1 isoform X3 [Homo sapiens]	gi|578801670	16.18	APGGGGGMAK	6.492546	7.972927
neuron navigator 2 isoform X16 [Homo sapiens]	gi|578820917	10.06	SATLGK	5.80277	6.716741

neuron navigator 2 isoform X7 [Canis lupus familiaris]	gi|545537057	13.36	KEAAAPSHSGIPKPGMK	11.45589	14.81798
neuron navigator 3 isoform X5 [Canis lupus familiaris]	gi|545524307	10.4	KLIEMEIER	4.400383	4.128621
neuronal tyrosine-phosphorylated phosphoinositide-3-kinase adapter 2 isoform X1 [Canis lupus familiaris]	gi|545542624	9.5299997	GHEGSYAGKHFR	7.539809	5.826526
neuropathy target esterase, isoform CRA_b [Homo sapiens]	gi|119589436	13.56	DGHLLMDGGYINNLPGNMGAK	5.249249	8.27661
neuropeptide FF receptor 2 isoform 1 [Homo sapiens]	gi|221316654	21.18	FIMNEK	5.696169	6.140661
neuroplastin isoform X1 [Homo sapiens]	gi|530405684	4.8800001	KKENGMPMDIVNTSGR	10.41	7.72915
neurosecretory protein VGF isoform X1 [Homo sapiens]	gi|530386359	12.05	APLPPPAPSQFQAR	8.788937	8.235501
neutrophil gelatinase-associated lipocalin precursor [Homo sapiens]	gi|38455402	35.91	SYPGLTSYLVR	8.361621	8.762851
niban-like protein 1 isoform X1 [Homo sapiens]	gi|530391419	9.5200005	AELGPR	9.046432	9.826352
nibrin isoform X2 [Homo sapiens]	gi|530389034	12.12	KLSSAVVFGGGEAR	5.51483	0
nicotinamide mononucleotide adenylyltransferase 2 isoform X2 [Canis lupus familiaris]	gi|545504566	10.99	KRRPLNQREGPTR	7.516202	5.250817
ninein isoform X6 [Homo sapiens]	gi|578825861	12.29	EKEPGNSALEEREQEKFNLK	10.20304	10.06454
ninjurin-2 isoform 1 [Homo sapiens]	gi|39725946	15.41	AAETQTAEPGGAHAVCSR	4.668653	7.118515
NMDA receptor synaptonuclear signaling and neuronal migration factor isoform X3 [Homo sapiens]	gi|530426699	14.59	VATFAKVEKEEDMIHFWK	3.082238	5.842653
nodal homolog precursor [Homo sapiens]	gi|222352098	7.4400001	HPGALEK	8.79866	11.33884
noelin-2, partial [Canis lupus familiaris]	gi|545535635	13.87	SMETLMRSLDAR	9.467372	10.17679
non-histone chromosomal protein HMG-17-like [Canis lupus familiaris]	gi|545520510	12.92	QIRHRKLK	6.783366	0
notch related protein, partial [Homo sapiens]	gi|1749368	4.79	GDSPGPR	8.093087	0
N-terminal asparagine amidase [Homo sapiens]	gi|119575136	4.6900001	TLAGGPR	5.081599	5.063367
nuclear chloride ion channel protein [Homo sapiens]	gi|15277274	11.47	AGSDGAK	11.49618	10.55337
nuclear envelope pore membrane protein POM 121C [Canis lupus familiaris]	gi|545500657	20	QRPLHASPPAK	8.694243	7.390553
nuclear export mediator factor NEMF isoform X1 [Homo sapiens]	gi|530404901	14.37	AVLAELNASLLGMR	6.339572	12.06729
nuclear factor 1 X-type isoform X2 [Canis lupus familiaris]	gi|545534498	15.77	TRPVSGHEAAPARLACASSR	5.934745	11.20633
nuclear factor erythroid 2-related factor 3 [Canis lupus familiaris]	gi|73976513	11.11	NEESKIANNPDWEAEKPTESSNI	8.408094	9.263986
nuclear factor of activated T-cells, cytoplasmic 2 isoform X1 [Homo sapiens]	gi|530418109	9.8800001	SSSPGAKR	8.050275	6.295987
nuclear factor of kappa light polypeptide gene enhancer in B-cells inhibitor-like 1 isoform X2 [Canis lupus familiaris]	gi|545518288	19.49	TSMASGSRR	9.292451	6.970824
nuclear fragile X mental retardation-interacting protein 2 isoform X1 [Canis lupus familiaris]	gi|545511991	17.91	RTSPQVLSSILK	7.222783	5.517711
nuclear mitotic apparatus protein 1 [Canis lupus familiaris]	gi|545536505	13.65	MAATSKEVARLEALVRK	12.36356	12.04225
nuclear mitotic apparatus protein 1 isoform X9 [Homo sapiens]	gi|578821456	9.8999996	QAASPLEPKELEELRDK	12.70616	10.76314
nuclear pore complex protein Nup214 isoform X3 [Homo sapiens]	gi|578817905	9.6199999	KRLNHLVDSLQQLRLYK	7.082883	11.4995
nuclear pore complex protein Nup93 isoform X1 [Homo sapiens]	gi|530424559	5.52	KPGVIDK	6.772432	9.664492
nuclear pore complex-interacting protein family member A8 isoform X2 [Homo sapiens]	gi|578840001	18.01	KLSMKEREHR	12.27617	8.925385
nuclear pore-associated protein 1 [Homo sapiens]	gi|112421134	22.559999	EATPQPK	8.868686	11.84254
nuclear receptor coactivator 6 isoform X5 [Canis lupus familiaris]	gi|545540437	9.0299997	QRMPMPLNTPLGSNSR	9.254717	5.542045
nuclear receptor coactivator 6 isoform X9 [Homo sapiens]	gi|578835897	12.8	MNGPMGAGNSVR	6.986142	8.118963
nuclear receptor corepressor 1 isoform X7 [Homo sapiens]	gi|578830118	8.3800001	TGVPARRMMKNQVMR	5.038948	7.040531
nuclear receptor subfamily 1 group I member 2 isoform X4 [Canis lupus familiaris]	gi|545552973	8.6099997	LGRFPDNNSEDFVVK	9.498339	9.94495
nuclear receptor subfamily 3, group C, member 1 (glucocorticoid receptor), partial [Homo sapiens]	gi|76057156	3.9100001	SSAST	8.644103	8.554751
nuclear receptor-interacting protein 2 isoform X2 [Homo sapiens]	gi|530398901	14.08	LVEGNPNWLQGEPPR	7.856394	9.767656
nuclear RNA export factor 2 [Homo sapiens]	gi|153791282	6.0100002	MCSTLK	7.412493	7.42287
nuclear RNA export factor 2-like [Canis lupus familiaris]	gi|545558743	11.63	MLCFSVSGVFREVEGK	8.334831	9.266696
nuclear RNA export factor 5 isoform X4 [Homo sapiens]	gi|530422124	7.3699999	LKPGQMEMLKLTMNK	8.138683	6.08378
nuclear speckle splicing regulatory protein 1 isoform 2 [Homo sapiens]	gi|387849036	9.4799995	MRNMAK	12.16984	12.29102
nuclear transcription factor Y subunit alpha isoform 2 [Homo sapiens]	gi|11496974	9.75	HAMARK	8.171779	10.36178
Nucleoside Diphosphate Kinase	gi|2935619	11.2	SCAHDWVYE	12.15331	8.893692
nyctalopin isoform X1 [Homo sapiens]	gi|530421507	5.6500001	LFSVPER	8.057825	9.421053
O-acetyl-ADP-ribose deacetylase MACROD2 isoform X2 [Homo sapiens]	gi|578835353	10.23	MYPSNKK	9.54576	7.12718
obscurin-like protein 1 isoform 3 precursor [Homo sapiens]	gi|291084513	6.0100002	TAGTE	5.05163	7.398203
occludin/ELL domain-containing protein 1 [Canis lupus familiaris]	gi|545534258	14.45	MRADVTGAAPFR	10.66248	10.34965
odorant-binding protein 2b isoform X2 [Homo sapiens]	gi|578817408	17.26	KLMYLQELPR	0	5.510025
olfactomedin-like protein 3 isoform X1 [Homo sapiens]	gi|578799479	17.09	SMKILKR	8.084929	4.754941
olfactory marker protein [Canis lupus familiaris]	gi|545536254	10.05	MAEDGPKQPQLSMPLVLDPDLT	10.58517	5.923497
olfactory receptor 10T2 [Homo sapiens]	gi|52218846	9.4300003	VLGMPVATK	0	8.263318
olfactory receptor 1M1 [Homo sapiens]	gi|52218828	1.47	ITSSS	9.94841	9.849752
olfactory receptor 226 [Canis lupus familiaris]	gi|545548494	6.04	MLLGLAAPR	5.324189	6.294446
olfactory receptor 2A12 [Homo sapiens]	gi|51921273	11.17	RVLWKQRSM	7.939412	7.446901
olfactory receptor 2AT4 [Homo sapiens]	gi|52627217	9.5100002	AAITK	5.060242	5.302423
olfactory receptor 4S2 [Canis lupus familiaris]	gi|545525059	6.2399998	EKXEG	8.193206	6.643983
olfactory receptor 5AP2 [Homo sapiens]	gi|50979290	6.9499998	DVKKALK	8.181358	11.47464
olfactory receptor 5B12 [Homo sapiens]	gi|52317108	6.3099999	MRSPEGR	9.248818	5.210751
olfactory receptor 5H14 [Homo sapiens]	gi|53933276	4.27	SVKGMR	3.80298	2.675793
olfactory receptor 5J2 [Homo sapiens]	gi|53828705	13.71	VVSMFYTLGIPMLNLLIHSLRNK	8.083844	8.828582
olfactory receptor family 3 subfamily A, partial [Canis lupus familiaris]	gi|37623761	15.4	AFSTCGSHLTVVCIFYGTGIFSYI	5.558491	8.556334
oligophrenin-1 isoform X2 [Canis lupus familiaris]	gi|545558331	8.7399998	KSPSIRPVSDGK	10.21915	10.49439
oligoribonuclease, mitochondrial [Canis lupus familiaris]	gi|545497341	12.36	AVKESTMTXQQAEYEFLSFVR	8.389802	7.158574
ornithine aminotransferase, mitochondrial isoform X4 [Canis lupus familiaris]	gi|545548244	29.049999	GLLNAIVIR	6.078594	10.91147
ornithine aminotransferase, partial [Homo sapiens]	gi|238721	2.24	MAIAA	6.481888	6.99111
otolin-1 [Canis lupus familiaris]	gi|359323777	3.27	TGLKGEAGDMGIPGPPGVVGPQ	9.156883	6.797674
OTU domain-containing protein 6B isoform X1 [Canis lupus familiaris]	gi|545549056	18.33	MGPRVK	7.328536	11.52273
OTU domain-containing protein 7B isoform X5 [Homo sapiens]	gi|578801304	13.46	FIFVGTLKMGHR	9.568319	10.60202
outer dense fiber protein 2-like isoform X4 [Homo sapiens]	gi|530363026	13.2	KKVFEK	9.030416	11.62144

ovochymase-1 precursor [Homo sapiens]	gi|110815798	5.6300001	VSASSMAK	7.652804	7.315682
ovostatin homolog 2-like [Canis lupus familiaris]	gi|545546410	41.509998	QVESLLQILDQSATK	9.991201	9.309699
oxidative stress-responsive serine-rich protein 1 isoform X3 [Homo sapiens]	gi|578836017	7.3099999	KMSHMAEMMYT	2.657881	9.585528
oxygen-regulated protein 1 [Homo sapiens]	gi|5454016	18.120001	ATNKSSETLALLEILK	8.790784	5.393997
oxygen-regulated protein 1 isoform X1 [Canis lupus familiaris]	gi|545548506	20.91	GISSLGLEEENNLK	9.764371	9.070399
P Chain P, 14-3-3 Isoform Sigma In Complex With A Phosphorylated C-raf Peptide	gi|545719804	19.280001	RSAXEPSL	4.549859	9.210189
P1 Cdc21 protein, partial [Homo sapiens]	gi|940536	12	AGTPSTMSSPASTPSR	13.93361	10.66696
P21 protein (Cdc42/Rac)-activated kinase 4 [Homo sapiens]	gi|12804135	20.040001	QENGMPEKPPGPR	11.64118	10.00492
P2X purinoceptor 3 [Homo sapiens]	gi|28416925	17.52	GNLLPNLTARDMK	9.094287	8.26745
P2X5a [Homo sapiens]	gi|7239180	12.8	MKAFLMAR	6.274813	8.748713
P2Y purinoceptor 2-like [Canis lupus familiaris]	gi|545554153	10.36	MEMLDTNASREQTVCKFSEDYI	8.962749	7.407291
p300/CBP-associated factor, isoform CRA_b, partial [Homo sapiens]	gi|119584710	16.4	EGVCCGGGGSGSAGGGGSARIA	4.712652	7.583516
p97 homologous protein, partial [Canis lupus familiaris]	gi|5441567	12.84	QGGFSLEEEEEEDA	7.66136	8.442485
pachytene checkpoint protein 2 homolog [Canis lupus familiaris]	gi|74003129	5.73	LSSCI	6.966166	5.129601
pantothenate kinase 4 [Homo sapiens]	gi|8922665	9.2600002	LKVDKEDVMTCLIK	7.869347	7.243705
parkin coregulated gene protein [Canis lupus familiaris]	gi|73946224	10.63	QILPILNIFK	4.307658	5.596184
parkin variant SV5DEL [Homo sapiens]	gi|284468411	4.46	IVFVR	8.907808	9.283569
partner and localizer of BRCA2 [Homo sapiens]	gi|27436910	11.43	SGNIKAVLGLTK	9.154323	9.409541
PAX-9, partial [Homo sapiens]	gi|4097409	22.17	LRQQDPGAIQR	8.039116	9.221569
PDZ domain-containing protein 2 isoform X8 [Homo sapiens]	gi|578809880	7.27	RSPHAIVVTQVK	7.133624	7.94261
pecanex-like protein 2 [Canis lupus familiaris]	gi|345798831	19.67	ASQEDMGLDDTASQQSASDEL	8.768238	11.32153
peptidyl-glycine alpha-amidating monooxygenase isoform X2 [Homo sapiens]	gi|578810405	61.369999	GNAILVR	8.920865	7.904503
peptidyl-prolyl cis-trans isomerase A-like [Homo sapiens]	gi|410172829	16.110001	ALSTGEKGFGYK	7.617615	7.678608
peptidyl-prolyl cis-trans isomerase E isoform X1 [Homo sapiens]	gi|578798221	15.36	SQKVESHTI	10.57344	11.53973
peptidyl-prolyl cis-trans isomerase-like 2 [Canis lupus familiaris]	gi|545544946	4.4200001	GDEVLAATMR	5.941223	5.039643
PERB11.1 protein, partial [Homo sapiens]	gi|1764036	9.5699997	AQTLXMNVRNLLK	4.184494	8.059481
perforin, partial [Homo sapiens]	gi|1247449	9.46	ALRALPR	8.667415	8.908373
pericentrin isoform X7 [Homo sapiens]	gi|530419256	21.08	AAVTLR	9.775983	8.8066
perilipin-4 isoform X22 [Canis lupus familiaris]	gi|545535177	14.08	GTVQTGLDTTK	8.447396	7.873967
peripherin isoform X2 [Homo sapiens]	gi|578823986	14.1	SGGRAGPLPSRR	5.162609	7.908004
peroxiredoxin 3 isoform a precursor, partial [Homo sapiens]	gi|134254696	7.9699998	NGGLGHMNIXLLSD	2.869867	8.465134
peroxisomal bifunctional enzyme isoform 1 [Homo sapiens]	gi|68989263	17.23	AVIDHTIKAIVICGAEGK	8.47317	10.9207
peroxisome proliferator-activated receptor gamma coactivator 1-beta isoform X1 [Canis lupus familiaris]	gi|545496109	9.1099997	HLTSVPSCPQTKARSPDR	7.528784	6.116677
persulfide dioxygenase ETHE1, mitochondrial [Canis lupus familiaris]	gi|73948241	22.959999	MAGATLRVAGR	10.67425	11.05757
PGC-1 and ERR-induced regulator in muscle 1 [Homo sapiens]	gi|523545115	15.06	TALLANVGTISAIR	5.782502	8.781119
PH and SEC7 domain-containing protein 4 isoform X5 [Homo sapiens]	gi|578804003	26.530001	TAWTLPR	10.39982	10.09569
PHD and RING finger domain-containing protein 1 isoform X3 [Homo sapiens]	gi|578839865	8.6700001	KKVPGRK	12.19271	10.67457
PHD finger protein 10 isoform b [Homo sapiens]	gi|194328736	13.98	RDLSHK	6.918173	0
PHD finger protein 20-like 1 isoform X7 [Canis lupus familiaris]	gi|545520880	17.26	AIADGRGAPTASGISKTEK	9.814461	12.04565
PHD finger protein 3 isoform X1 [Homo sapiens]	gi|578812606	24.07	QNMTTDAPKKIVAAK	5.181479	0
PHD finger protein 3 isoform X5 [Homo sapiens]	gi|578812608	9.3800001	VEKGVLNVHPAASASKPSADQI	7.248994	9.387173
Phenol-sulfating phenol sulfotransferase 1 variant, partial [Homo sapiens]	gi|62087878	8.4700003	TTFTVAQNERFDADYAEKMAG	12.5782	5.815874
phosphatase and actin regulator 1 isoform X4 [Homo sapiens]	gi|578811493	18.91	GEDAR	7.332702	11.14589
phosphatidate phosphatase LPIN3 isoform X3 [Canis lupus familiaris]	gi|545540728	13.31	EPLVPVDLDALA	12.08239	9.368643
phosphatidate phosphatase LPIN3 isoform X8 [Homo sapiens]	gi|578836161	13.35	GLNPATLSGGIDVLVVK	6.138118	9.800576
phosphatidylinositol 3,4,5-trisphosphate 3-phosphatase and dual-specificity protein phosphatase PTEN isoform X1 [Homo sapiens]	gi|578819653	7.7800002	ADNDK	7.3343	9.638001
phosphatidylinositol 3,4,5-trisphosphate-dependent Rac exchanger 1 protein isoform X3 [Homo sapiens]	gi|530418228	18.200001	TTPSVGRSFSIRFGR	10.90178	10.13415
phosphatidylinositol-glycan biosynthesis class W protein isoform X1 [Homo sapiens]	gi|578840336	28.559999	LPFLKILEK	9.548439	6.633392
phosphatidylinositol-glycan-specific phospholipase D isoform X1 [Homo sapiens]	gi|530381810	7.0500002	MYALTSDAQPLLLSTFSGDRRFS	6.942526	9.326467
phosphatidylserine decarboxylase proenzyme isoform X2 [Homo sapiens]	gi|530419773	11.36	MATSVGHR	12.57941	13.20727
phosphoglycerate kinase 1 [Homo sapiens]	gi|4505763	8.2299995	MSLSNKLTLDKLDVKGK	6.950219	7.478419
phosphoinositide 3-kinase [Homo sapiens]	gi|2143260	6.8000002	VTMVNADPLGEEINVMFKVGEI	10.6121	8.807673
phosphoinositide-3-kinase, regulatory subunit 5, p101, isoform CRA_b, partial [Homo sapiens]	gi|119610443	9.2600002	ESLGMPGR	10.7123	9.115014
phospholipase A2, group IVB (cytosolic), isoform CRA_a [Homo sapiens]	gi|119612926	11.28	MMPAER	9.89873	11.9993
phospholipase D2 isoform X5 [Homo sapiens]	gi|578829958	13.22	CSSYRQAR	8.515697	12.28385
phospholipid-transporting ATPase IB isoform X2 [Canis lupus familiaris]	gi|545541992	8.7799997	VQELNPTMFKMR	6.461188	3.441635
Pig3, partial [Homo sapiens]	gi|2415310	13.41	IGDTAMALLPGGGQAQYVTVPF	9.503186	8.800226
PIGP_HUMAN RecName: Full=Phosphatidylinositol N-acetylglucosaminyltransferase subunit P	gi|425906062	6.75	STSLALIVFLFHRLSK	0	4.887387
pikachurin isoform X4 [Canis lupus familiaris]	gi|545496336	11.92	HGSSPR	8.241648	8.860712
pim-3 oncogene, partial [Homo sapiens]	gi|119593891	6.54	GAAAQNPR	8.547479	9.167701
pituitary adenylate cyclase-activating polypeptide type I receptor isoform X4 [Canis lupus familiaris]	gi|545522923	14	EFDFPDTNSLDLSDMR	7.190134	7.058738
PKD1L2 protein, partial [Homo sapiens]	gi|33988793	12.47	LQAVSVPSAGTR	6.273199	11.25041
plakophilin-3, partial [Canis lupus familiaris]	gi|545531425	13	GLDSYGGHR	0	10.14926
platelet endothelial cell adhesion molecule isoform X5 [Canis lupus familiaris]	gi|545509952	7.4299998	IISGTHMETSRAIKSELVTVTESF	1.956754	7.233762
platelet-activating factor acetylhydrolase IB subunit alpha [Homo sapiens]	gi|4557741	16.25	VMELESKLNEAK	7.013926	10.36485
platelet-derived growth factor receptor alpha isoform X3 [Homo sapiens]	gi|578808641	4.1700001	QATREVKEMKK	9.532102	9.944596
pleckstrin homology domain containing, family H (with MyTH4 domain) member 3, isoform CRA_b [Homo sapiens]	gi|119581267	10.02	SPSTVSAMAMWPPAS	8.969141	10.05046
pleckstrin homology domain-containing family A member 1 isoform X2 [Homo sapiens]	gi|530394084	9.3100004	GPGRSASSMRQAR	5.855463	7.338499
pleckstrin homology domain-containing family A member 4 isoform X8 [Homo sapiens]	gi|578834700	0.26	QPDVEQR	6.097875	5.804333

pleckstrin homology domain-containing family A member 6 isoform X6 [Homo sapiens]	gi|578800665	10.74	GRMLSVQALAEANAVK	6.447249	7.488708
pleckstrin homology domain-containing family A member 7 isoform X2 [Homo sapiens]	gi|578820348	6.3600001	QDSSGMR	2.428803	10.12951
pleckstrin homology domain-containing family M member 3 [Canis lupus familiaris]	gi|345797470	9.9399996	GTAPDNLSWMEQK	7.812507	5.836295
plectin [Homo sapiens]	gi|1296662	13.77	LPVTDAVNKGLVDK	8.644919	7.418335
plexin-D1 precursor [Homo sapiens]	gi|157694524	27.66	KGFAELQTDMTDLTK	12.55298	10.50755
PMS1 protein homolog 1 isoform X7 [Canis lupus familiaris]	gi|545555609	15.07	KSNVVDNK	11.0311	10.75413
PNMA-like protein 2 [Canis lupus familiaris]	gi|545488058	8.3500004	AGPLXR	6.391097	7.512635
PO113_HUMAN RecName: Full=Endogenous retrovirus group K member 113 Pol protein	gi|52000770	11.43	AAYTGLK	6.58615	6.418214
podocan-like protein 1 isoform X4 [Homo sapiens]	gi|578833638	13.23	SLDLAGNQLTRLPMGLPTGLR	6.956185	5.886564
poliovirus receptor-related protein 3 isoform X1 [Homo sapiens]	gi|530374342	17.02	GPLLPR	5.404915	9.931771
polo-like kinase 3 variant, partial [Homo sapiens]	gi|62089336	12.51	NHAQER	9.146515	8.067474
polo-like kinase variant, partial [Homo sapiens]	gi|62087938	3.1500001	SASNR	9.920992	10.35075
poly(A) RNA polymerase GLD2 isoform X2 [Canis lupus familiaris]	gi|545492929	19.93	RDLNSILPLR	5.840222	5.270335
poly(A)-specific ribonuclease PARN-like domain-containing protein 1 isoform 2 precursor [Homo sapiens]	gi|27735155	16.559999	DIWKEMNFPR	8.534607	8.060173
polyadenylate-binding protein 1-like isoform X1 [Homo sapiens]	gi|530418415	21.860001	RPPAHISSVR	9.023748	7.605659
polyadenylate-binding protein 4-like [Homo sapiens]	gi|291084655	4.73	EAELRSK	4.052745	5.544621
polymerase delta-interacting protein 2 isoform 1 [Homo sapiens]	gi|7661672	7.0900002	GRGVVGREPVLSK	6.749978	0
polymeric immunoglobulin receptor isoform X1 [Homo sapiens]	gi|530366266	55.889999	DGSFSVVITGLR	10.41512	7.154752
polymeric immunoglobulin receptor, partial [Canis lupus familiaris]	gi|19715659	60.98	SNVDLQVLKPEPNLLYGDLR	6.719744	8.786472
polypeptide N-acetylgalactosaminyltransferase 13 isoform X2 [Homo sapiens]	gi|578803575	20.74	NQEGPGEMGKAVLIPK	5.099987	8.226254
polypeptide N-acetylgalactosaminyltransferase 2 precursor [Homo sapiens]	gi|4758412	23.32	RAAEVWMDEYK	12.58158	8.100752
polypeptide N-acetylgalactosaminyltransferase 9 [Canis lupus familiaris]	gi|545544592	13.02	NALRAAEVWMDSFK	9.796371	8.386049
POM121-like protein 1-like [Homo sapiens]	gi|578810772	14.72	GGYPR	6.938128	7.676544
POM121-like protein 2 [Homo sapiens]	gi|253683544	12.26	LEEPLFPESLDSKRR	9.553732	9.755756
porphobilinogen deaminase isoform X4 [Canis lupus familiaris]	gi|545497150	12.03	SGNGTAAAAAEGNGPKMR	8.468019	9.179763
post-GPI attachment to proteins factor 3 [Canis lupus familiaris]	gi|545510883	12.68	LVLLAGAAALASASQGDR	8.623145	10.69658
potassium voltage-gated channel subfamily G member 2 [Canis lupus familiaris]	gi|545484957	6.3000002	AXSLGLR	8.103065	8.660789
potassium voltage-gated channel subfamily H member 1 isoform 1 [Homo sapiens]	gi|27437001	7.6799998	TMAGGR	10.10849	7.794284
potassium voltage-gated channel subfamily H member 6 isoform X2 [Canis lupus familiaris]	gi|545509878	1.54	SAMSR	9.09593	8.683201
potassium voltage-gated channel subfamily S member 3 [Homo sapiens]	gi|539846921	19.02	FDTLRFGQLR	8.807417	10.71427
POTE ankyrin domain family member F [Homo sapiens]	gi|153791352	17.02	ILTEHGYR	5.389313	9.514524
PRAME family member 19 [Homo sapiens]	gi|194306635	15.54	YLSQMKNLR	9.284836	9.761498
pre-miRNA 5'-monophosphate methyltransferase [Canis lupus familiaris]	gi|345791865	19.74	QAAGGVEEAAEEPR	7.765585	7.845332
pre-mRNA 3' end processing protein WDR33 isoform X2 [Homo sapiens]	gi|530369258	17.75	MATEIGSPPRFFHMPR	9.856583	9.614566
presequence protease, mitochondrial isoform 3 precursor [Homo sapiens]	gi|334085252	19.799999	VTSVPELFLTAVK	11.45543	5.928952
prickle-like protein 3 isoform X2 [Canis lupus familiaris]	gi|74006940	24.77	SPSPQLPR	7.102504	8.820665
programmed cell death 2, isoform CRA_c [Homo sapiens]	gi|119567800	12.93	AVPQQGGRAAGMAGRGR	8.848637	8.141124
programmed cell death protein 7 [Canis lupus familiaris]	gi|545550687	22.27	AAGAAARTLR	6.393333	7.163574
pro-interleukin-16 isoform X2 [Canis lupus familiaris]	gi|545493612	12.84	GNEVLSINGKSLKGATHNDALA	6.65198	8.223002
prolactin-induced protein, partial [Homo sapiens]	gi|116642859	51.560001	TYLISSIPLXGAFNYK	4.770256	7.993619
prolactin-inducible protein [Canis lupus familiaris]	gi|73978762	55.400002	KAAPNEEISVTVR	8.93924	8.040951
prolactin-inducible protein precursor [Homo sapiens]	gi|4505821	54.91	ELGICPDDAAVIPIK	8.003637	8.460135
proline-rich protein 25 [Homo sapiens]	gi|61966733	10.44	GTPPTPNPWGPEQPQNR	9.905784	9.515381
proline-rich protein 36 [Canis lupus familiaris]	gi|545535659	16.18	SVSSPPQHGPAEPSPATR	6.696017	9.093012
proline-rich proteoglycan 2-like [Canis lupus familiaris]	gi|545556419	0.89	RAGPRAPLQGIPGPQGIPR	9.408558	12.51899
prominin-2 [Canis lupus familiaris]	gi|545527761	10.4	VDEVVR	9.629622	4.546239
propionyl-CoA carboxylase alpha chain, mitochondrial isoform X2 [Homo sapiens]	gi|530423370	4.5100002	EIGYPVMIKASAGGGGKGMR	2.431233	6.9498
proprotein convertase subtilisin/kexin type 9 preproprotein [Homo sapiens]	gi|31317307	4.1300001	MEAQGGK	7.04421	9.424653
prostaglandin reductase 1 isoform 1 [Homo sapiens]	gi|226059133	13.2	MEAFVVYR	8.208415	9.373308
proteasome (prosome, macropain) subunit, alpha type, 3, isoform CRA_g [Homo sapiens]	gi|119601127	23.85	AVAGLLADARSLADIAREEASN	6.373388	4.044662
proteasome subunit beta type-9 precursor [Homo sapiens]	gi|4506205	12.34	MLRAGAPTGDLPR	7.795496	5.097155
protein AHNAK2 isoform X1 [Homo sapiens]	gi|530403029	12.92	EVATK	7.442744	6.166994
protein AKNAD1 [Homo sapiens]	gi|91754185	1.46	GPGDK	6.776213	5.979533
protein ALEX isoform Alex [Homo sapiens]	gi|117938768	6.1300001	AAAPGWR	7.080414	8.999566
protein arginine N-methyltransferase 3 isoform 1 [Homo sapiens]	gi|44771198	17.01	MCSLASGATGGR	9.534612	9.322538
protein artemis isoform X3 [Canis lupus familiaris]	gi|545489929	3.1199999	HIPQEK	8.688091	9.165092
protein BEX1-like [Canis lupus familiaris]	gi|345806410	13.64	WDVFQRLEEPQGR	0	7.707167
protein C, partial [Homo sapiens]	gi|265668	11.54	SAATSTGSMGTSETRKPPRR	4.637686	8.159034
protein C19orf12 homolog isoform X2 [Canis lupus familiaris]	gi|545489119	14.99	MLPSRLAKMPLVVEDVMR	9.841034	5.095496
protein C21orf2 isoform X3 [Canis lupus familiaris]	gi|545551397	10.03	KMVLSR	7.9468	4.772134
protein cramped-like [Homo sapiens]	gi|223972612	5.9200001	NNHAWAR	7.285309	8.308795
protein Daple isoform X3 [Homo sapiens]	gi|530403839	14.91	ASDPPAIGGQPGPPAK	10.36371	9.219697
protein DENND6A isoform X2 [Homo sapiens]	gi|578805769	12.21	YVASLMPLQK	8.967007	8.0243
protein diaphanous homolog 2 isoform 12C [Homo sapiens]	gi|6382071	10.7	EQYEKLSTMHNNMMK	9.446681	8.63926
protein dispatched homolog 1 isoform X7 [Homo sapiens]	gi|578801639	15.88	QMNKPKAIGMIDGQMIIMK	4.665881	10.79705
protein disulfide-isomerase [Canis lupus familiaris]	gi|73964749	19.360001	AAGTLK	6.321062	8.364559
protein ECT2 isoform X6 [Canis lupus familiaris]	gi|545554082	27.02	TSLADSSIFDCK	7.307772	13.65413
protein ELYS isoform X2 [Homo sapiens]	gi|578802192	13.26	LSSYPK	4.796399	3.067194
protein enabled homolog isoform X6 [Homo sapiens]	gi|578802282	11.96	VTATQDSTNLRCIFCGPTLPR	8.339695	8.964616
protein eyes shut homolog [Canis lupus familiaris]	gi|345778944	15.28	NGFQGCIFTMQVR	9.877044	7.701892
protein FAM110A [Homo sapiens]	gi|46391104	11.34	GPADGGARKPSAVER	3.615547	0
protein FAM149A isoform X4 [Homo sapiens]	gi|530377521	10.15	VLSAMPDGTERSRLR	12.4918	8.778696
protein FAM161B [Canis lupus familiaris]	gi|545508021	19.83	GKPQVHSPPSPR	7.719611	6.506101
protein FAM161B [Homo sapiens]	gi|333609271	13.68	LGFLHTNFR	7.026428	7.347002

protein FAM167A isoform X1 [Homo sapiens]	gi|530387464	11	IEHTCR	3.133744	3.450585
protein FAM171A2 precursor [Homo sapiens]	gi|222831660	32.619999	NGTGVIR	5.526131	7.17956
protein FAM171B isoform X1 [Homo sapiens]	gi|530369954	10.64	RDQTTSTTHINHISTVKVALK	11.19718	5.329621
protein FAM179A isoform X1 [Homo sapiens]	gi|578802744	1.4299999	GSGPR	9.680412	7.878798
protein FAM183A isoform X1 [Homo sapiens]	gi|530362646	2.53	AGHPK	5.737935	8.207311
protein FAM184B [Homo sapiens]	gi|210032790	12.32	HTVEIKSVRSSVEAER	12.56942	9.73188
protein FAM186A [Canis lupus familiaris]	gi|545545518	15.33	IDNIGKPLDK	8.188441	12.05738
protein FAM188B isoform X3 [Canis lupus familiaris]	gi|545522914	10.63	SGLIVRGMMAGPIASSPQDSVRI	12.25207	12.49349
protein FAM193A isoform X3 [Homo sapiens]	gi|578808301	8.8999996	AQTESK	9.208439	9.451943
protein FAM193B isoform X9 [Homo sapiens]	gi|578811158	30.26	QELPEPVSSGGKPQK	9.382264	9.49981
protein FAM194B isoform X2 [Canis lupus familiaris]	gi|545537455	18.379999	KLGILLKK	11.47506	9.336673
protein FAM208A isoform X5 [Homo sapiens]	gi|578805903	10.33	FLYSAPR	7.511267	5.108909
protein FAM214A isoform X7 [Homo sapiens]	gi|578827203	9.2299995	EKIINENYNPKFFGNLQSDDSK	10.60391	6.666265
protein FAM3D isoform X2 [Canis lupus familiaris]	gi|545533257	11.02	GLNIALVNGTTGMVLTQK	9.544139	6.688869
protein FAM65C isoform X3 [Homo sapiens]	gi|578835791	5.25	MVTTMSVR	10.48321	6.7836
protein FAM92B isoform X2 [Homo sapiens]	gi|530423868	11.79	AAVDSSR	8.35367	8.029363
protein FRG1 isoform X3 [Homo sapiens]	gi|578840732	12.38	DGFLHETLLDR	8.334109	9.776297
protein furry homolog isoform X1 [Homo sapiens]	gi|578824707	9.2700005	KFMAELK	9.899318	5.511146
protein HEATR9 isoform X6 [Homo sapiens]	gi|578840327	20.799999	METLTGLR	10.77258	9.64397
protein HEXIM2 isoform X2 [Homo sapiens]	gi|578830381	19.799999	RAGVTSSRRLLK	10.54384	9.733209
protein HGH1 homolog [Homo sapiens]	gi|13124773	10.9	KMLVEAIMLLTATAPGR	9.745188	7.373736
protein HIRA isoform X2 [Canis lupus familiaris]	gi|545544833	3.74	SRGGH	9.02586	8.647711
protein KIAA2022 [Homo sapiens]	gi|56711286	9.5100002	LFSFDSMAPLSVSSSNYCSLSLK	11.94249	13.69507
protein kinase C, zeta, isoform CRA_b [Homo sapiens]	gi|119576535	12	HMDLKPVIDGMDGIK	8.025087	7.657712
protein KTI12 homolog [Homo sapiens]	gi|19923939	12.9	RAEELRVALAAEGR	12.32244	10.40949
protein LTV1 homolog [Canis lupus familiaris]	gi|545485529	5.3400002	QVERMQMINGSDLPKVSTQPRS	7.724605	10.6051
protein LYRIC isoform X4 [Homo sapiens]	gi|530389397	12.25	ASLLKSQEPIPDDQK	6.566615	9.112394
protein MSS51 homolog, mitochondrial [Canis lupus familiaris]	gi|345799109	8.1700001	VDGGV	6.174039	2.940312
protein naked cuticle homolog 1 isoform X3 [Canis lupus familiaris]	gi|545491211	10.21	GLQAPLAVGSSVVGRDR	7.724929	11.86216
protein NDRG3 isoform X4 [Homo sapiens]	gi|578836095	14.75	HQTMEVSC	7.969818	9.731564
protein phosphatase 1 regulatory subunit 14C [Homo sapiens]	gi|19311006	12.78	SVATGSSETAGGASGGGAR	7.027766	9.242814
protein phosphatase 1 regulatory subunit 16A isoform X3 [Homo sapiens]	gi|578816221	10.09	MLDDIR	9.87394	7.803605
protein phosphatase 1 regulatory subunit 1C isoform 2 [Homo sapiens]	gi|387528000	13.81	GPNTQGELQNASPK	9.832305	8.932956
protein phosphatase 1 regulatory subunit 3D [Canis lupus familiaris]	gi|545541256	5.6300001	VHFALR	10.14019	8.632564
protein phosphatase 1, regulatory (inhibitor) subunit 13B, isoform CRA_a [Homo sapiens]	gi|119602251	23.4	KIQACEK	11.52319	12.02308
protein phosphatase 1N [Canis lupus familiaris]	gi|545488138	11.66	SLLDAPR	9.028709	11.1189
protein piccolo isoform X1 [Homo sapiens]	gi|578814114	21.4	KDAAPK	4.029744	4.058362
protein PRRC2A isoform X2 [Homo sapiens]	gi|578842365	13.73	GVPSR	9.350012	9.818809
protein PRRC2B [Canis lupus familiaris]	gi|345805923	12.61	KELAKRSFSSQRPLADR	7.777759	0
protein prune homolog 2 isoform X5 [Canis lupus familiaris]	gi|545486507	14.26	KMPGLGWMK	9.782856	7.65034
protein RD3-like [Canis lupus familiaris]	gi|545508665	12.29	QVLKDFLNSTDK	5.5947	7.913571
protein RER1 [Canis lupus familiaris]	gi|545499141	13.29	GKEDGGKTFAS	4.026446	6.321088
protein RUFY3 isoform X8 [Canis lupus familiaris]	gi|545521746	19.059999	RLQNNRSIPGKGSQKPEPK	8.260886	10.80438
protein S100-A8 isoform d [Homo sapiens]	gi|21614544	61.41	ALNSIIDVYHK	7.585237	7.673948
protein S100-A9 [Homo sapiens]	gi|4506773	23.92	NIETIINTFHQYSVK	6.894409	13.28543
protein sel-1 homolog 1 isoform X2 [Homo sapiens]	gi|530404461	10.42	EAYRYLQKAASMNHTK	11.01574	7.747264
protein SET isoform X1 [Canis lupus familiaris]	gi|545513094	11.52	KPPSLGR	10.5805	7.56512
protein shisa-9 isoform X2 [Canis lupus familiaris]	gi|545502150	9.7799997	QAYGNK	0	11.37733
protein SHQ1 homolog isoform X3 [Canis lupus familiaris]	gi|545532943	16.15	SGVFQR	7.780016	7.277837
protein Shroom2 isoform X1 [Homo sapiens]	gi|578837883	8.3299999	EGAEPR	2.365711	4.629308
protein SOGA3 precursor [Homo sapiens]	gi|59806361	3.71	TPGTGSR	9.148387	8.360332
protein spinster homolog 1 isoform X2 [Homo sapiens]	gi|578828681	23.27	ITGLSPGR	7.702745	9.319436
protein spire homolog 2 isoform X6 [Homo sapiens]	gi|578829294	7.8400002	LTDPRGAQAHYQAVCR	4.372893	7.050013
protein strawberry notch homolog 2 isoform X3 [Canis lupus familiaris]	gi|545535456	12.74	RVLQELQLMDADVK	0	6.180917
protein SZT2 isoform X3 [Homo sapiens]	gi|578798759	13.55	GLGGAGGGSSPSK	10.01839	7.906884
protein TBATA [Canis lupus familiaris]	gi|545495163	9.29	MATDVK	7.786519	9.905578
protein tilB homolog isoform X1 [Homo sapiens]	gi|578815913	15.29	DLKILYLQNNLIGK	4.91075	4.984389
protein unc-119 homolog B [Homo sapiens]	gi|122937500	6.6900001	MSGSNPK	8.870388	9.05184
protein unc-80 homolog isoform X9 [Homo sapiens]	gi|578804139	10.95	DSGMLKYIRLQVMSLSPAPLSLI	7.877096	5.114181
protein Wnt-10b precursor [Homo sapiens]	gi|16936522	15.77	AVGAALRERLGR	9.996707	12.21643
protein ZNF720 [Canis lupus familiaris]	gi|545487551	7.6900001	RVTIHPGK	8.544958	8.079046
protein-associating with the carboxyl-terminal domain of ezrin isoform X3 [Homo sapiens]	gi|578801312	13.48	GSENSALK	9.356513	11.01869
protein-glutamine gamma-glutamyltransferase 5 isoform X1 [Canis lupus familiaris]	gi|545549630	9.9300003	QIQANLRSNKFK	6.740897	8.615051
protein-glutamine gamma-glutamyltransferase K isoform X2 [Canis lupus familiaris]	gi|545506684	12.38	KEVVLAPGATER	6.020097	11.34271
protein-methionine sulfoxide oxidase MICAL3 isoform X15 [Homo sapiens]	gi|578836826	18.43	AQIIQR	5.338539	0
proteoglycan 4 [Canis lupus familiaris]	gi|545506100	10.16	TAPPSPGASQTIKSTAKR	5.883	4.835173
protocadherin alpha 4 isoform 1 precursor variant, partial [Homo sapiens]	gi|62087430	16.049999	VRLLENVPNGTLVIK	8.172131	6.552337
protocadherin beta 5 precursor variant, partial [Homo sapiens]	gi|62088366	4.8699999	TAEIR	9.092994	7.601665
protocadherin Fat 1 isoform X1 [Homo sapiens]	gi|578808907	4.5900002	SSTTR	4.822361	6.612826
protocadherin Fat 3 precursor [Homo sapiens]	gi|148886692	4.71	ASGAIR	10.47359	8.710365
protocadherin gamma-A8 isoform 1 precursor [Homo sapiens]	gi|14270484	12.36	GYLVTKVVAVDR	6.431595	6.342057
protocadherin gamma-C4 isoform 2 precursor [Homo sapiens]	gi|14277682	12.1	LSSNEHFALDVKK	5.503319	7.4475
protocadherin-18 isoform X1 [Homo sapiens]	gi|530377844	3.8	KGPLPAK	8.147829	7.169141
protocadherin-23 isoform 3 [Homo sapiens]	gi|216548395	8.29	KMGEGR	5.886738	7.970014
protocadherin-23-like [Canis lupus familiaris]	gi|545525131	16.370001	HTLDREAR	9.933716	7.145289

proto-oncogene DBL isoform X5 [Homo sapiens]	gi|578838679	4.1199999	GATKMK	6.871601	7.677527
proto-oncogene Wnt-1 precursor [Homo sapiens]	gi|4885655	12.06	AVGDVLR	7.184056	7.482087
PSD-95/SAP90-associated protein-2, partial [Homo sapiens]	gi|2454510	8.8599997	IRHLVHSVQKXFTKSHSLEGSSK	7.514798	6.898877
pseudopodium-enriched atypical kinase 1 isoform X7 [Homo sapiens]	gi|578827415	16.34	KMIIR	7.302127	3.765358
pseudouridylate synthase 7 homolog isoform X4 [Homo sapiens]	gi|578814362	20.82	EMLTADNLDIDNMR	7.405872	9.247056
PT0205 insulin-like growth factor-binding protein, bone - human (fragment)	gi|87947	14.57	LGFFVXVEPDDKAAL	7.550006	12.14347
pterin-4-alpha-carbinolamine dehydratase 2 [Homo sapiens]	gi|134133240	19.870001	MAAVLGALGATRRLLAALR	7.269856	8.512965
PTK XI Eph/Elk/Eck orphan receptor family, partial [Homo sapiens]	gi|38502059	34.630001	HSLALSPR	9.805895	8.571264
PTTG1 protein, partial [Homo sapiens]	gi|89243589	7.5700002	MPSPPWESNLLQSPSSILSTLDV	13.03536	8.556599
purinergic receptor P2Y2, partial [Homo sapiens]	gi|241044069	13.52	PDXSAPELFSR	7.000254	4.917084
PWWP domain-containing protein 2A isoform X3 [Homo sapiens]	gi|530380846	9.6700001	QVLCDK	9.290144	7.605086
pyroglutamyl-peptidase 1-like protein isoform X1 [Homo sapiens]	gi|578826639	9.1199999	MDTAAK	8.708681	8.253585
pyruvate kinase PKLR isoform X4 [Homo sapiens]	gi|578839334	26.83	SAQLLSR	9.917811	8.57458
R Chain R, Structure Of Uhrf1 In Complex With Histone Tail	gi|374074050	16.67	QTARXSTGG	10.80304	9.701705
R3H and coiled-coil domain-containing protein 1 isoform X1 [Homo sapiens]	gi|530387620	2.9400001	AAATR	7.647768	7.773917
rab GDP dissociation inhibitor beta isoform 2 [Homo sapiens]	gi|169646441	3.0999999	KTTMR	7.400682	5.370831
rab11 family-interacting protein 1 isoform 1 [Homo sapiens]	gi|289547526	19.16	KEGLSFLGGLR	8.624218	9.116162
rab11 family-interacting protein 1 isoform X2 [Canis lupus familiaris]	gi|545526225	9.3199997	GGSMTSAIK	9.814958	8.678248
RAB6A-GEF complex partner protein 1 isoform X1 [Homo sapiens]	gi|530390030	17.65	TVGEQLLK	7.560114	8.935002
rabankyrin-5 isoform 3 [Homo sapiens]	gi|384367970	12.33	CRDAGGK	7.802736	8.781734
rabenosyn-5 isoform X1 [Homo sapiens]	gi|530372982	8.6700001	KAKDRLLK	0	10.06725
rabenosyn-5 isoform X4 [Homo sapiens]	gi|530372986	16.82	QKGGTD	9.215682	12.06605
rab-interacting lysosomal protein isoform X1 [Homo sapiens]	gi|578840161	11.59	MLGTPEEAESSFGLWYRGK	3.370456	9.289872
RAB-R protein [Homo sapiens]	gi|4102709	9.2299995	VMAAXK	5.799301	5.347797
Rad51, partial [Homo sapiens]	gi|38017105	15.63	VVGTTG	6.015412	10.43851
RAD51-associated protein 1 isoform X4 [Homo sapiens]	gi|578822547	14.94	LGLSRLAR	9.390214	10.82289
radial spoke head 1 homolog isoform X2 [Homo sapiens]	gi|530419417	8.6400003	MSDLGSEELEEEGENDIGVR	7.626627	4.204196
ran-binding protein 17 [Homo sapiens]	gi|12597633	13.57	RDVAEALR	7.006416	9.725665
rapamycin-insensitive companion of mTOR isoform X2 [Homo sapiens]	gi|578809893	13.4	TIPDDTPMCR	4.017378	6.440796
ras association domain-containing protein 1 isoform B [Homo sapiens]	gi|332701155	9.3400002	TSFYLPKDAVKHLHVLSR	5.115802	3.342285
ras GTPase-activating-like protein IQGAP1 isoform X1 [Homo sapiens]	gi|530407289	11.45	YLDELMK	5.659623	7.071146
ras-associated and pleckstrin homology domains-containing protein 1 isoform X1 [Homo sapiens]	gi|578804759	7.7199998	SGSPGK	5.362089	6.757307
ras-associating and dilute domain-containing protein [Homo sapiens]	gi|148612825	8.25	MFYGTHFIMSPPTKSK	0	7.692249
ras-GEF domain-containing family member 1C isoform X1 [Homo sapiens]	gi|578811061	8.3599997	GAAHR	10.73929	10.44947
ras-related protein Rab-15 isoform X1 [Homo sapiens]	gi|530403647	5.8499999	SYQHIMK	4.63197	7.943341
ras-related protein Rab-8B isoform X1 [Homo sapiens]	gi|578827085	13.18	ERGEKLAIDYGIK	5.146407	7.35569
RBPJ-interacting and tubulin-associated protein 1 isoform X1 [Homo sapiens]	gi|530401496	16.1	TRGVGK	6.446119	8.706676
rcNIP30, partial [Homo sapiens]	gi|82802820	11.96	DGGDDGNLVIK	8.2896	8.081914
RD RNA binding protein, isoform CRA_b [Homo sapiens]	gi|119623957	8.0900002	DRSHEDRERDR	9.801406	9.922295
RE1-silencing transcription factor variant E1b/E2/E3/N3c/E4 [Homo sapiens]	gi|429336190	7.6500001	ATQVMGQSSGGGGLFTSSGNIG	3.555012	9.820274
receptor-type tyrosine-protein phosphatase S isoform X9 [Homo sapiens]	gi|578833433	14.15	GPGPFSPPVR	8.342138	9.456954
receptor-type tyrosine-protein phosphatase T isoform X4 [Homo sapiens]	gi|578835721	0.89	MVNSSGR	10.66022	9.694985
receptor-type tyrosine-protein phosphatase T isoform X5 [Homo sapiens]	gi|578835723	12.51	VADLLQHITQMKR	2.689536	6.867982
recombining binding protein suppressor of hairless [Canis lupus familiaris]	gi|545494264	11.65	GEKSLTQASELSSILSPSPVSR	9.243988	7.951772
regulator of G-protein signaling 14 [Canis lupus familiaris]	gi|545495645	8.0699997	PGKPKHLGVPNGRMVLAVSDGI	6.71041	8.744281
regulator of G-protein signaling 6 isoform X7 [Canis lupus familiaris]	gi|545507938	6.9899998	KGSQGE	11.12622	9.951525
regulator of G-protein signaling 9 [Homo sapiens]	gi|3284012	8.2399998	YGDQSKVK	8.314817	7.931222
RelA associated inhibitor [Homo sapiens]	gi|4679034	9.7399998	HGGPGPGGR	10.79335	5.904088
relA-associated inhibitor [Canis lupus familiaris]	gi|545488152	12.29	RSLGSTGPSGTLPR	9.906085	9.23278
replication factor C subunit 1 isoform 2 [Homo sapiens]	gi|325296984	2.8399999	MVASK	8.241573	7.976101
retinoblastoma-associated protein [Canis lupus familiaris]	gi|545537983	7.71	MLGRGTAGGACGGHR	8.723348	10.05764
retinoblastoma-like protein 2 isoform X2 [Homo sapiens]	gi|530424182	17.07	LQDVANDR	0	4.789761
retinoic acid-induced protein 1 [Canis lupus familiaris]	gi|545498438	8.7700005	AAVPPGR	7.72267	8.45199
retinoic acid-induced protein 1 [Homo sapiens]	gi|40807477	20.950001	AAVPQK	10.07406	7.731163
retinol-binding protein 3 [Canis lupus familiaris]	gi|73953972	15.7	VELPGR	7.794278	11.15019
retrotransposon gag domain-containing protein 1 [Canis lupus familiaris]	gi|545559000	8.9499998	ASVSGIMTTPLR	8.836455	8.915927
RGS12 protein, partial [Homo sapiens]	gi|78070480	12.98	AVGPLCASNSEPLK	9.282669	8.963124
Rh blood group D antigen, partial [Homo sapiens]	gi|336092033	4.79	RGSQVPA	6.017047	8.249844
Rhesus CcEe antigen, partial [Homo sapiens]	gi|60656598	5.3600001	AMLGA	7.692308	6.285959
rho GTPase-activating protein 11A isoform X3 [Homo sapiens]	gi|578841031	18.280001	SSMELPSKSFLKMR	3.142738	5.615314
rho GTPase-activating protein 20-like isoform X1 [Canis lupus familiaris]	gi|545560212	2.24	AADSGQK	8.104246	8.192935
rho GTPase-activating protein 21 isoform X9 [Homo sapiens]	gi|578818522	7.3899999	AYRMEIQVPPSPTDVAK	9.981709	11.24261
rho GTPase-activating protein 23 isoform X6 [Homo sapiens]	gi|578840371	20.379999	ALAVGGQGR	5.268754	5.28277
rho GTPase-activating protein 24 isoform X2 [Homo sapiens]	gi|530378251	2.02	GSGIVTNGSFSSSNAEGLEK	6.782196	8.676351
rho GTPase-activating protein 27-like [Canis lupus familiaris]	gi|545510099	7.9400001	AAVAPPAGLLGSAGSFK	0	6.468294
rho GTPase-activating protein 32 isoform X2 [Homo sapiens]	gi|578822484	16.65	TGLLSVAEGKESR	8.75046	12.09743
rho GTPase-activating protein 39 [Canis lupus familiaris]	gi|545521864	9.5699997	QTRAGGGAGDTARKSRPAAR	5.682444	11.56795
rho GTPase-activating protein 40 isoform X1 [Homo sapiens]	gi|578835971	17.1	DFYAGLFSWDEVHHNDASDLLF	10.06319	8.121028
rho GTPase-activating protein SYDE2 [Canis lupus familiaris]	gi|545503798	11.5	REPARRPAVGARASGGGGGAA	9.850204	7.155661
rho guanine nucleotide exchange factor 12 isoform X2 [Canis lupus familiaris]	gi|545497086	13.01	QVGETPASGDCIDNTPR	7.854491	6.768183
rho guanine nucleotide exchange factor 12 isoform X2 [Homo sapiens]	gi|578822090	15.4	TGTGDIATCYSPR	9.688317	9.769969
rho guanine nucleotide exchange factor 28 isoform X2 [Homo sapiens]	gi|578810490	6.9200001	SAAEK	8.004735	7.800756
rho guanine nucleotide exchange factor 33 [Homo sapiens]	gi|290463441	29.530001	APPADGVAPR	10.03757	8.361071
rho guanine nucleotide exchange factor 37 isoform X4 [Homo sapiens]	gi|578810882	14.67	HGADEPSSR	8.604163	8.854249

rho guanine nucleotide exchange factor 7 isoform X6 [Canis lupus familiaris]	gi|545538064	11.78	GASSPGILVLTTGLSRPFMR	9.376763	9.072858
rho guanine nucleotide exchange factor 9 isoform X7 [Homo sapiens]	gi|578838334	7.27	QAAMTVRKVPKQK	3.108998	6.844081
rho-associated protein kinase 2 isoform X1 [Homo sapiens]	gi|530366978	6.6300001	GNDTDVRRK	3.952352	7.273541
rhophilin associated tail protein 1-like [Canis lupus familiaris]	gi|345796304	10.58	NGMIGLSDFFVLTRKVXETSR	6.859889	10.38035
rho-related GTP-binding protein RhoN isoform X1 [Homo sapiens]	gi|530412930	7.9499998	MGMGTR	0	6.494822
rho-related GTP-binding protein RhoQ isoform X4 [Homo sapiens]	gi|530367411	10.33	CINCCLIT	5.70298	7.958072
ribonuclease 3 isoform X4 [Homo sapiens]	gi|530378791	12.76	VHHMHMR	9.596526	9.813989
ribonuclease H2 subunit B isoform X1 [Homo sapiens]	gi|578825007	18.52	GNPEIDNKKYYK	7.908504	8.754545
ribosomal protein S6 kinase alpha-2 isoform X2 [Homo sapiens]	gi|578812928	14.94	FFSVYLR	9.879951	8.363642
ribosomal protein S6 kinase-like 1 isoform X16 [Homo sapiens]	gi|578826178	13.3	ARSQAHVYLEQIR	10.47478	10.51619
ribosome biogenesis protein BMS1 homolog isoform X9 [Homo sapiens]	gi|578844679	23.4	LTGFPYK	11.25823	8.452851
ribosome-binding protein 1 [Homo sapiens]	gi|110611220	12.9	VTVKHLEEIVEKLK	7.723821	4.734947
RIMS-binding protein 2 [Homo sapiens]	gi|140561070	4.9299998	AARYK	4.129493	7.30556
RIMS-binding protein 3A-like [Canis lupus familiaris]	gi|345791573	8.1999998	LKEDTGHSLLPWK	3.430883	10.50299
RING finger protein 219 isoform X1 [Homo sapiens]	gi|578825001	15.61	LENGGLVR	7.717427	7.677902
RNA polymerase II elongation factor ELL2 isoform X1 [Homo sapiens]	gi|578810253	13.46	NRSTKVIKPGGPYVGKR	8.607916	8.741629
RNA polymerase II subunit B1 CTD phosphatase RPAP2 isoform X1 [Homo sapiens]	gi|578799784	4.02	AGAPR	7.01861	4.244205
RNA polymerase II subunit B1 CTD phosphatase RPAP2 isoform X2 [Canis lupus familiaris]	gi|545503257	10.62	KAGQKANSK	0	6.43417
RNA polymerase II transcription factor SIII subunit A2 [Homo sapiens]	gi|45439357	17.65	KKDNHALVR	10.0327	9.434211
RNA polymerase II-associated factor 1 homolog isoform X2 [Canis lupus familiaris]	gi|545488561	7.5999999	TVAVMGVASGAGAAAPVPSPV	10.40687	5.069788
RNA polymerase-associated protein LEO1 isoform 2 [Homo sapiens]	gi|556695389	8.0500002	RKNAIASDSEADSDTEVPK	5.750716	8.858758
RNA pseudouridylate synthase domain-containing protein 2 isoform 2 [Homo sapiens]	gi|556695367	3.3499999	NTVHR	5.160336	4.667418
RNA-binding motif protein, X chromosome isoform 1 [Homo sapiens]	gi|56699409	6.25	SRGPPRGLR	8.13002	0
RNA-binding motif, single-stranded-interacting protein 1 isoform X10 [Homo sapiens]	gi|578804703	16.389999	AVSALKASGVQAQMAK	8.410133	6.930494
RNA-binding protein 10 isoform X3 [Homo sapiens]	gi|530421602	0.39	DMDYR	6.393559	7.431452
RNA-binding protein 12B [Canis lupus familiaris]	gi|545549096	16.23	NLSLSINK	5.244123	11.78399
RNA-binding protein 12B isoform X5 [Homo sapiens]	gi|578815970	8.0100002	MAVVIR	8.748471	11.86705
RNA-binding protein 41 isoform X6 [Homo sapiens]	gi|578838428	8.6999998	MMTGRMR	7.154968	5.511506
RNA-binding protein 5 isoform X4 [Homo sapiens]	gi|578805520	21.77	KDLVLSDGNR	10.20275	10.31704
RNA-binding protein 6 isoform X2 [Homo sapiens]	gi|578805512	16.360001	ESWSGETRQDGESKTIMLKR	7.810242	8.878651
RNA-binding protein EWS isoform X4 [Homo sapiens]	gi|578837007	5.6100001	GGPGGMRGGR	4.897143	5.035775
RNA-binding protein EWS isoform X4 [Homo sapiens]	gi|578837007	12.65	GGFDRGGMSRGGR	10.9775	9.353531
RNA-binding protein FUS isoform X2 [Homo sapiens]	gi|530407875	12.5	GGFGPGKMDSR	5.178518	9.189282
RNF103-CHMP3 protein [Homo sapiens]	gi|312147344	11.33	VAGSLQKSTEVMKAMQSLVK	4.966844	6.841728
rootletin [Canis lupus familiaris]	gi|359319094	6.4299998	AEXAEALSKAEAGR	3.087352	5.460652
rootletin-like [Homo sapiens]	gi|578843302	24.799999	AGGLLGMPEDRR	6.435694	10.87331
roundabout homolog 3 isoform X2 [Canis lupus familiaris]	gi|545496959	17.969999	AVPYR	7.299198	9.310877
RRBP1_CANLF RecName: Full=Ribosome-binding protein 1	gi|23822071	14.47	AEGAPNQGKKGEGTPNQGKK	6.558872	9.879037
rRNA 2'-O-methyltransferase fibrillarin isoform X1 [Homo sapiens]	gi|530415994	8.2700005	GGGFHSGGNRGRGR	2.980217	8.355592
rRNA-processing protein EBP2 isoform 2 [Homo sapiens]	gi|237649015	11.63	KAGAK	8.908319	6.19523
RRP12-like protein isoform 3 [Homo sapiens]	gi|547234776	13.41	AEARAK	8.366889	8.422298
RRP12-like protein isoform X3 [Canis lupus familiaris]	gi|545547546	19.15	KKVKLQGQFK	8.333795	8.655828
RUN and FYVE domain-containing protein 2 isoform X14 [Homo sapiens]	gi|578819619	3.1600001	ANLLNMAK	7.336002	6.684384
RUN and SH3 domain-containing protein 1 [Canis lupus familiaris]	gi|345802607	7.6799998	SPGLDSNR	6.24709	6.734877
runt-related transcription factor 3 [Canis lupus familiaris]	gi|345794032	17.41	AVGGAAARGLR	6.481514	8.669632
ryanodine receptor 1 isoform X3 [Homo sapiens]	gi|578834746	6.3600001	QAPPSPPPKK	7.796855	11.88697
S Chain S, 14-3-3 ZetaPHOSPHOPEPTIDE COMPLEX (MODE 1)	gi|157879766	12.67	ARSHXYPA	6.728881	5.301864
S Chain S, Structure Of Camp Dependent Protein Kinase A In Complex With High Ba2+ Concentration, Adp And Phosphorylated Peptide Psp20	gi|512125581	8.5100002	AXIHD	7.099659	10.4797
S phase cyclin A-associated protein in the endoplasmic reticulum isoform X1 [Homo sapiens]	gi|530405927	16.32	LKEYEQRLNELQEER	7.766108	11.96064
S10812 phosphatidylserine-binding phosphoprotein - human (fragments)	gi|107288	20.35	QXAQVKR	9.2677	8.803028
S65716 prostaglandin-D synthase (EC 5.3.99.2) - human (fragments)	gi|2135996	11.21	AALSMXK	0	8.226482
S71083_1 Unknown, partial [Homo sapiens]	gi|4261775	13.05	PSSLSASVGERVTITCR	11.45865	10.38285
S71519_1 immunoglobulin light chain variable region, partial [Homo sapiens]	gi|4261788	9.7399998	DIQMTQSPDSLTVSLGER	6.564265	9.569649
SAA protein partial (AA 1-52), partial [Homo sapiens]	gi|825714	9.6300001	AEAISDARENIQR	6.743839	8.412275
SAFB-like transcription modulator isoform X12 [Homo sapiens]	gi|578827413	12.45	ARPTARR	0	6.306786
SAGA-associated factor 29 [Homo sapiens]	gi|19923935	10.3	IAGLYNDSEPPRKTMRR	10.04555	10.11688
SAM domain and HD domain 1 [Homo sapiens]	gi|22209036	22.790001	FVEQLIR	0	6.034351
SAM domain-containing protein SAMSN-1 isoform 3 [Homo sapiens]	gi|557357765	18.629999	AISWTMK	9.916738	8.66761
schlafen family member 12 isoform X3 [Homo sapiens]	gi|578831122	10.35	GYSTQTALTLK	10.18292	8.046916
sciellin isoform X12 [Canis lupus familiaris]	gi|545537749	13.52	VATSLQK	6.853614	5.074838
SCRL protein [Homo sapiens]	gi|119592243	12.65	MGMEGPFFR	6.976064	7.921656
SEC23-interacting protein isoform X2 [Canis lupus familiaris]	gi|545548137	18.93	QKAVKLEQKK	9.604817	0
SEC31-like 2 (S. cerevisiae), isoform CRA_d [Homo sapiens]	gi|119570209	17.629999	QVTLEQDSRMK	8.495163	11.00081
secretogranin II [Homo sapiens]	gi|338051	11.38	ENIGKNEQINDEMKR	4.593132	4.013719
secretogranin-3 isoform 2 [Homo sapiens]	gi|259089433	5.25	QADAYVEK	7.761466	5.98806
segment polarity protein dishevelled homolog DVL-2 isoform X2 [Homo sapiens]	gi|578829758	23.950001	MAGSSTGGGGVGETK	7.975156	8.362932
selenocysteine insertion sequence-binding protein 2 isoform X1 [Homo sapiens]	gi|578817876	10.67	RMSQMKTPHNPLDSSAPLMK	7.255975	6.263138
selenoprotein O [Homo sapiens]	gi|32880229	10.73	AVYRAALGASLAAAR	8.447899	9.714581
semaphorin-4A isoform X5 [Canis lupus familiaris]	gi|545505398	13.09	HAVLLPAAAPAEPR	8.765307	7.447306
sentrin-specific protease 6 isoform X4 [Canis lupus familiaris]	gi|545519677	11.57	MKDQFGNSIVSTPLK	9.315833	12.31629
septin-2 isoform X1 [Canis lupus familiaris]	gi|545543197	16.969999	HLPGGERGGGGGGSRPRPR	7.527994	10.40653
serine hydroxymethyltransferase, cytosolic isoform X1 [Homo sapiens]	gi|530410579	16.219999	SKGTDGGRAEK	5.046425	7.579045
serine/arginine repetitive matrix protein 2 isoform X7 [Homo sapiens]	gi|578828053	13.82	SRSGSSSK	8.617452	9.223814
serine/arginine repetitive matrix protein 2-like [Canis lupus familiaris]	gi|545506591	6.4299998	GPEGGSAASGSGAAAPAR	6.013758	9.313928

serine/arginine-rich splicing factor 1 isoform X1 [Homo sapiens]	gi|578831236	13.95	SGRGTGRGGGGGGGGGAPR	9.871583	10.3342
serine/threonine kinase-like domain-containing protein STKLD1 [Homo sapiens]	gi|525345385	10.33	VVVQEEGGSGLSLIKETYQLHR	6.53074	10.63164
serine/threonine-protein kinase 16 [Homo sapiens]	gi|57165436	24.780001	YLFIQK	4.479151	8.138786
serine/threonine-protein kinase 3 isoform 3 [Homo sapiens]	gi|372622371	17.030001	SKLKKLSEDSLTK	9.712188	8.526371
serine/threonine-protein kinase 32C isoform X1 [Homo sapiens]	gi|578819297	13.53	LYICEMALALDYLR	8.021841	12.21594
serine/threonine-protein kinase BRSK1 [Homo sapiens]	gi|24308326	5.9699998	MSSGAK	10.90521	9.983898
serine/threonine-protein kinase Chk2 isoform X4 [Homo sapiens]	gi|578836887	25.82	VVGNKRLKEATCK	8.081441	10.16949
serine/threonine-protein kinase H1 [Homo sapiens]	gi|27901803	15.55	YDIKALIGR	6.632448	10.25729
serine/threonine-protein kinase MRCK alpha-like isoform X4 [Canis lupus familiaris]	gi|545486807	12.82	KEMSRMFKVTASQLSAPSNR	10.92475	9.356412
serine/threonine-protein kinase MRCK beta isoform X3 [Homo sapiens]	gi|530404961	13.51	SMSDPDQDFDK	9.219642	9.048421
serine/threonine-protein kinase Nek11 isoform X6 [Homo sapiens]	gi|578807732	14.24	KLQAADEKARK	13.52799	13.16263
serine/threonine-protein kinase Nek8 [Homo sapiens]	gi|30039692	5.9400001	IMSGTFAPISDR	0	6.634113
serine/threonine-protein kinase PINK1, mitochondrial [Canis lupus familiaris]	gi|545492239	13.12	SYQEAQLPAMPESVPLDAR	10.24654	7.677415
serine/threonine-protein kinase PLK4 isoform X2 [Homo sapiens]	gi|578808733	10.22	MTVFPK	9.929951	9.947659
serine/threonine-protein kinase Sgk1 isoform 1 [Homo sapiens]	gi|25168263	9.4700003	MTVKTEAAK	8.740792	8.239804
serine/threonine-protein kinase SMG1 isoform X3 [Homo sapiens]	gi|530407765	10.78	SLGQELR	8.242403	10.17325
serine/threonine-protein kinase TNNI3K isoform X2 [Canis lupus familiaris]	gi|545501528	16.83	SMTKEKADVLLLR	8.511613	9.36938
serine/threonine-protein kinase tousled-like 2 isoform X11 [Homo sapiens]	gi|578830286	21.200001	SSPQHSLSNPLPR	10.11303	11.3944
serine/threonine-protein kinase ULK4 isoform X7 [Homo sapiens]	gi|578806260	5.3499999	LLDSPSTCIRAK	6.707405	6.187267
serine/threonine-protein kinase WNK1 isoform X18 [Homo sapiens]	gi|578822664	7.8099999	TMAKAIKDRVSLIK	13.20038	10.55006
serine/threonine-protein kinase WNK2 [Canis lupus familiaris]	gi|545489201	5.4099998	DPGSPDK	9.645875	7.275546
serine/threonine-protein kinase/endoribonuclease IRE1 isoform X1 [Homo sapiens]	gi|578830598	13.08	DLKPHNILISMPNAHGK	7.999281	10.3923
serine/threonine-protein phosphatase 2A activator isoform b [Homo sapiens]	gi|30065643	3.3800001	MAEGER	4.193203	4.385029
serine/threonine-protein phosphatase 6 regulatory ankyrin repeat subunit C [Canis lupus familiaris]	gi|545514167	11.24	TVGGCGALPHGASCPYSQER	8.137968	6.897422
serine/threonine-protein phosphatase 6 regulatory subunit 2 isoform X17 [Homo sapiens]	gi|578837746	14.33	NGPERGGQDGKASLEAHR	9.37109	12.25332
serine/threonine-protein phosphatase 6 regulatory subunit 3 isoform X18 [Canis lupus familiaris]	gi|545530528	11.27	ASASEGR	9.533467	11.31104
serine-protein kinase ATM isoform X6 [Homo sapiens]	gi|578822209	14.83	DILSVR	8.149681	7.831971
serine-protein kinase ATM isoform X7 [Homo sapiens]	gi|578822213	10.42	VAERVLMRLQEKLK	14.53237	8.236193
serotonin N-acetyltransferase isoform 2 [Homo sapiens]	gi|4501845	8.79	GPILLWR	7.832949	8.401661
serpin B7 isoform X1 [Homo sapiens]	gi|578832747	10.52	QLPQSTLFR	9.097426	7.838232
serum albumin [Canis lupus familiaris]	gi|22531688	101.85	LGEYGFQNALLVR	9.02206	7.254401
serum albumin isoform X1 [Canis lupus familiaris]	gi|545520919	51.040001	DVFLGTFLYEYSR	11.80181	6.271523
serum response factor-binding protein 1 [Homo sapiens]	gi|103471995	16.870001	KEQQSNIAVFQGK	9.773294	9.678097
sesquipedalian-1 [Canis lupus familiaris]	gi|545544608	10.3	DDPGPGGAAMKLNER	11.92043	9.386868
SET-binding protein isoform X3 [Homo sapiens]	gi|578832440	24.93	AGKNSK	8.807778	7.456433
seven transmembrane helix receptor [Homo sapiens]	gi|27348191	14.05	MSSLKGKSRAAEVWK	6.068025	8.70611
SFRS8 protein, partial [Homo sapiens]	gi|27371125	9.0699997	MGDHK	7.817279	9.006603
SH2B adapter protein 3 isoform X1 [Canis lupus familiaris]	gi|545543838	8.8999996	AGLARK	8.842726	0
SH3 domain-binding protein 5 isoform X1 [Homo sapiens]	gi|578806777	11.62	YYVQLEQLKKTVDDLQAK	7.097704	6.724601
SH3 domain-containing protein 19 isoform X10 [Homo sapiens]	gi|578808810	15.89	AEGRR	11.83088	9.085673
SH3 domain-containing protein 21 isoform X7 [Canis lupus familiaris]	gi|545523621	7.9099998	DPVAKEPK	7.687429	7.455871
SHC SH2 domain-binding protein 1 [Homo sapiens]	gi|190358504	20.190001	ADGSLTGGGLEAAAMAPER	8.352014	9.909964
SHC-transforming protein 4 isoform X2 [Homo sapiens]	gi|530405840	15.22	HLLLVDPEGK	9.0805	9.050496
short-chain dehydrogenase/reductase 10d [Homo sapiens]	gi|32396160	0.72	AAPGPK	5.6958	8.417229
shugoshin-like 2 isoform X2 [Homo sapiens]	gi|530369919	9.4300003	MECPVMETGSLFTSGIK	8.436821	6.921148
signal recognition particle 54 kDa protein isoform X2 [Homo sapiens]	gi|530404536	3.21	LVDPGVK	7.019653	6.313505
signal-induced proliferation-associated 1-like protein 1 isoform X1 [Homo sapiens]	gi|578825709	6.3200002	QNTQSDIGGSGKSTPSWQR	6.221921	8.035487
signal-induced proliferation-associated 1-like protein 3 isoform X2 [Homo sapiens]	gi|530416037	10.25	NEIGGECER	4.331312	7.405923
single Ig IL-1-related receptor [Canis lupus familiaris]	gi|545531427	14.06	GPIFGEPLAPPHASGVSLGEGR	8.196969	5.614464
sirtuin (silent mating type information regulation 2 homolog) 3 (S. cerevisiae), isoform CRA_a, partial [Homo sapiens]	gi|119581643	7.8099999	EAGAGR	11.30224	0
SKI family transcriptional corepressor 2 isoform X1 [Homo sapiens]	gi|578832646	18.110001	FIFHSHR	5.802037	4.595206
slit homolog 3 protein isoform 1 precursor [Homo sapiens]	gi|429836873	14.37	KAFRGITDVK	9.417358	9.693621
SLIT-ROBO Rho GTPase-activating protein 1 isoform X1 [Homo sapiens]	gi|530400607	15.14	GEPPPPVR	9.464196	8.154198
small conductance calcium-activated potassium channel protein 2 isoform a [Homo sapiens]	gi|25777645	14.16	MSSCRYNGGVMRPLSNLSASRF	8.634477	6.142186
smoothelin-like 1 isoform X5 [Canis lupus familiaris]	gi|545530028	17.389999	EDGGK	7.658849	8.421926
sodium- and chloride-dependent GABA transporter 3 isoform 1 [Homo sapiens]	gi|7657587	12.72	GKLGVSPR	10.77507	8.294638
sodium channel protein type 7 subunit alpha isoform X2 [Canis lupus familiaris]	gi|545555090	12	KRSPTSIITSLNMLDDAAAAIR	8.069038	5.302587
sodium/hydrogen exchanger 7 isoform X4 [Canis lupus familiaris]	gi|545557941	20.799999	EPGDAAR	8.485203	9.236897
sodium/potassium/calcium exchanger 1 isoform X8 [Homo sapiens]	gi|530406679	13.17	AESKPEGR	3.291	9.032393
sodium/potassium-transporting ATPase subunit beta-3 [Homo sapiens]	gi|4502281	7.9899998	MTKNEK	6.634793	6.564034
sodium-dependent noradrenaline transporter isoform X1 [Homo sapiens]	gi|578829177	13.7	ERNGVQCLLAPR	10.17124	9.97796
sodium-driven chloride bicarbonate exchanger isoform X6 [Homo sapiens]	gi|578804626	10.9	ADSGK	4.935324	3.568337
solute carrier family 12 (potassium/chloride transporters), member 4, isoform CRA_a [Homo sapiens]	gi|119603597	21.690001	QLGGG	9.347181	8.609757
solute carrier family 15 member 1 [Homo sapiens]	gi|4827008	4.8600001	MGMSK	7.058002	8.841955
solute carrier family 22 member 6 isoform X3 [Canis lupus familiaris]	gi|545531139	11.94	ALQRVAWINGKQEEGTK	7.852726	6.856302
solute carrier family 25 member 41 [Homo sapiens]	gi|149274607	10.82	MQAQDTVEGSNPTMRGVLQR	5.308721	10.71415
solute carrier family 25 member 52 [Homo sapiens]	gi|393193618	15.89	IWLERDRK	7.176512	6.618404
solute carrier family 28 member 3 [Homo sapiens]	gi|11545853	7.1199999	KEGGPK	8.572227	7.754977
solute carrier organic anion transporter family member 2B1 isoform 3 [Homo sapiens]	gi|223634014	8.9399996	EARTK	4.016809	9.872215
solute carrier organic anion transporter family member 4A1 isoform X2 [Canis lupus familiaris]	gi|545541302	11.48	AAPDMHYVAAGPQGAACGWR	8.56382	8.796834
solute carrier organic anion transporter family member 4C1 [Canis lupus familiaris]	gi|359319147	6.5	AYPVR	10.06407	9.46674

sorbin and SH3 domain-containing protein 2 isoform 7 [Homo sapiens]	gi|224586853	13.3	QEMDPGLSK	2.626882	4.529185
sortilin isoform X2 [Canis lupus familiaris]	gi|545502966	7.96	DFGGK	7.447508	6.981199
sorting nexin-18 isoform X2 [Canis lupus familiaris]	gi|545496184	10.49	SDLSLGARGGAAPQHPPSGAK	3.839071	8.078999
sorting nexin-25 isoform X4 [Homo sapiens]	gi|578809437	18.02	RVVISHNMDK	7.611645	0
SPAG6 [Homo sapiens]	gi|49065530	7.6900001	AVVDAGAVPLLVLCIQEPEIALK	8.191728	7.5158
sparc/osteonectin, cwcv and kazal-like domains proteoglycan precursor variant, partial [Homo sapiens]	gi|62088982	16.73	ELGPKDKEGK	8.364473	6.554161
spastin isoform X1 [Homo sapiens]	gi|530368010	13.13	APSYSGLSMVSGVKQGSGPAPT	5.805287	7.360558
spatacsin isoform X1 [Homo sapiens]	gi|578827438	17.389999	DGHQLK	8.644232	7.312675
specifically androgen-regulated gene protein [Canis lupus familiaris]	gi|545506078	15.93	LPEPQGLGPRSGSYSLPR	7.808653	9.528255
spectrin beta chain, non-erythrocytic 1 isoform X1 [Homo sapiens]	gi|578803120	14.59	ANNQKVYMPR	5.361625	7.40663
spectrin beta chain, non-erythrocytic 5 [Canis lupus familiaris]	gi|545550640	14.76	EKATSLPSDVAPDLR	7.412769	9.023736
spectrin beta chain, non-erythrocytic 5 isoform X1 [Homo sapiens]	gi|530405965	16.360001	HTFSLR	1.137884	9.708331
sperm flagellar protein 2 isoform X4 [Homo sapiens]	gi|578809999	19.5	QEHLIEFFFR	10.17041	9.187054
sperm-associated antigen 8 isoform X4 [Homo sapiens]	gi|578816456	14.14	HGHRGLLTMQLK	8.769353	8.943472
spermatogenesis-associated protein 1 isoform X1 [Homo sapiens]	gi|410171200	11.42	YHAYNGWKKKYLETK	7.298378	9.429222
spermatogenesis-associated protein 24 isoform X2 [Canis lupus familiaris]	gi|545490280	9.6800003	QQENYMAQVLDQK	4.181625	5.737551
spermatogenesis-associated protein 31D3 [Homo sapiens]	gi|223633944	3.01	RKSVTFKDR	0	7.178272
spermatogenesis-associated protein 31E1 [Canis lupus familiaris]	gi|545486799	8.1899996	EKDGSIGK	7.747999	5.780239
sperm-tail PG-rich repeat-containing protein 2 isoform X1 [Homo sapiens]	gi|578809052	28.139999	KSCPIPLFVK	10.64379	9.771464
spidroin-1-like [Homo sapiens]	gi|578798093	10.57	ITPATALLLLSHRGRAEXAAAAK	7.736678	8.451076
SPINLW1-WFDC6 fusion protein precursor [Homo sapiens]	gi|312176446	16.67	KASLST	9.402096	8.250783
splicing factor U2AF 35 kDa subunit-like isoform X3 [Homo sapiens]	gi|578846884	25.969999	GGGGGGGGGGGGRERDR	10.74801	8.309655
splicing regulatory glutamine/lysine-rich protein 1 isoform X2 [Homo sapiens]	gi|578810144	21.77	MAGDETQPTR	8.559505	9.435917
SPRY domain-containing SOCS box protein 3 isoform X2 [Homo sapiens]	gi|530409358	13.35	APVSDPQAATSAHPSSREPRPCC	7.986148	11.07419
SRY-box 9, partial [Homo sapiens]	gi|556559930	11.96	AGSSRTSTR	6.867368	5.773663
Sry-related HMG box gene [Homo sapiens]	gi|1881852	16.65	MPALRINSGAGPLK	9.578435	10.01346
ST8 alpha-N-acetyl-neuraminide alpha-2,8-sialyltransferase 3 variant, partial [Homo sapiens]	gi|62087572	3.1300001	GPAALR	8.825348	9.709059
stAR-related lipid transfer protein 7, mitochondrial isoform X1 [Canis lupus familiaris]	gi|545527727	11.83	RPPALAAWLAGAAAAGRAGAR	12.28396	7.241366
sterile alpha motif domain-containing protein 3 [Canis lupus familiaris]	gi|345784807	14.75	IGYQSVLMDLIKK	9.402789	8.617411
sterol regulatory element-binding protein 1 isoform X2 [Homo sapiens]	gi|578830023	2.1900001	AGPGGK	9.07324	8.902847
STIP1 protein [Homo sapiens]	gi|73909112	17.639999	AAALELLNR	6.606299	6.193454
stomatin-like protein 1 isoform X8 [Homo sapiens]	gi|578827623	17.26	IWDPVLSVMTVK	7.660284	8.84415
structure-specific endonuclease subunit SLX1 isoform X1 [Canis lupus familiaris]	gi|73958514	11.26	NSVLWGDLIWLCR	7.820021	7.900209
submaxillary gland androgen regulated protein 3A isoform X4 [Canis lupus familiaris]	gi|545521728	39.07	IPPPPPPYGPGR	10.3875	10.65685
submaxillary mucin-like protein [Canis lupus familiaris]	gi|545547103	31.93	EAILNCPGGSTIPYR	10.75848	10.18902
succinate dehydrogenase [ubiquinone] flavoprotein subunit, mitochondrial isoform X3 [Homo sapiens]	gi|530378875	12.27	TSELRLSMQK	4.584366	6.90924
succinate dehydrogenase complex, subunit A, flavoprotein precursor variant, partial [Homo sapiens]	gi|62087562	13.35	AAATADMSGVR	10.49274	9.930948
sulfotransferase 1A2 isoform X2 [Homo sapiens]	gi|578828630	6.8099999	GVEEDR	10.00509	9.084874
supervillin isoform X15 [Homo sapiens]	gi|578818557	7.0700002	QSIGQFHEGDAYVVKWK	12.72575	10.91852
supervillin isoform X8 [Canis lupus familiaris]	gi|545489761	17.1	KRKVLTR	6.977159	7.814438
suppressin [Homo sapiens]	gi|3293442	7.5100002	IHADAKR	5.585447	0
surfeit locus protein 6 [Canis lupus familiaris]	gi|545512726	15.05	APTAYAAPRRAR	9.545834	9.289369
SURP and G-patch domain-containing protein 2 [Homo sapiens]	gi|224282117	12.52	AQDLLRAVPRSRAEMYDDVHSI	7.016129	6.56879
sushi, nidogen and EGF-like domain-containing protein 1 isoform X4 [Homo sapiens]	gi|578804038	8.3500004	RTGLCIRRR	9.997515	10.75483
SWI/SNF complex subunit SMARCC2 isoform X4 [Homo sapiens]	gi|530400737	27.629999	QVDADTKAGR	7.743437	0.8279464
SWI/SNF related, matrix associated, actin dependent regulator of chromatin, subfamily a, member 3, isoform CRA_b [Homo sapiens]	gi|119599296	9.3000002	KMKLILSSGSDEE	5.159911	7.12356
synaptophysin-like protein 2 isoform X2 [Homo sapiens]	gi|530362428	12.95	SSTESAGRTADK	4.596101	7.96409
synaptotagmin-7 isoform X11 [Homo sapiens]	gi|530397577	23.559999	GTWTHHTTVEPPPSR	7.55456	10.34989
synaptotagmin-like 1, isoform CRA_d, partial [Homo sapiens]	gi|119628167	6.52	REGGDPGGR	8.382148	11.68573
synaptotagmin-like protein 2 isoform X13 [Homo sapiens]	gi|578821513	26.59	SVPAFLQDESDDR	9.70179	8.684337
SYNGR1 [Homo sapiens]	gi|47678711	8.3599997	MEGGAYGAGK	0	8.055223
synovial sarcoma, X breakpoint 2, isoform CRA_c [Homo sapiens]	gi|119576267	15.45	RKYEAMTKLGIMPK	8.867019	7.223428
syntaxin binding protein 2 variant, partial [Homo sapiens]	gi|62897515	21.74	KLGDIALP	4.977469	5.174895
syntaxin-16 isoform X8 [Canis lupus familiaris]	gi|545541225	11.61	MALVSGISLDPEAAIGVTKR	8.500758	7.004117
syntaxin-2 isoform X3 [Homo sapiens]	gi|578823407	18.16	TQHSVLSR	9.366776	9.793102
syntaxin-binding protein 5 isoform X3 [Canis lupus familiaris]	gi|545485595	15.2	SPAKPMQTITPHGK	9.648636	9.749835
T cell receptor beta chain CDR3, partial [Homo sapiens]	gi|3859246	13.04	TGGQFFG	9.095382	9.0105
T cell receptor beta chain, partial [Homo sapiens]	gi|29647033	14.98	QAYNE	8.981292	9.608153
T cell receptor beta variable 28, partial [Homo sapiens]	gi|161729036	10.47	KAAMGIR	5.144397	8.304602
T cell receptor delta chain variable region CDR3 domain {clone B-ST-6} [human, LCL-responsive T cells, Peptide Partial, 23 aa]	gi|861563	17.690001	CALGK	5.195375	5.035005
T Chain T, Crystal Structure Of Anthrax Edema Factor (Ef) In Complex With Calmodulin In The Presence Of 10 Millimolar Exogenously Added Calcium Chloride	gi|66360504	12.1	KXKDTDSEEEIR	9.971284	9.737789
T Chain T, Crystal Structure Of The Complex Of Human Chromobox Homolog 3 (cbx3)	gi|379318422	28.58	KARXSAGAAKR	6.07288	12.31076
T Chain T, Monoclinic Form Of Human C-reactive Protein	gi|371927385	5.9200001	QTDMSR	9.71903	8.898749
T54 protein [Homo sapiens]	gi|1663764	8.2799997	GAISGGK	3.960407	4.773088
TACC2 protein [Homo sapiens]	gi|219519155	12.62	SAKDIGLWKAMMPSLDTDTLDV	11.85273	5.752947
talin-2 isoform X14 [Homo sapiens]	gi|578827489	14.38	AAGNAVK	8.931836	9.046158
talin-2 isoform X15 [Homo sapiens]	gi|578827491	13.94	MVALSLK	11.63062	6.835671
target of Myb protein 1 isoform X2 [Canis lupus familiaris]	gi|545514991	19.809999	DGLAGALDARQQSTGAMGGSSI	10.32366	5.254966
taste receptor type 2 member 41 [Homo sapiens]	gi|116268093	12.12	EWLRYGRLLPLDMILISLGASR	7.23126	6.644675

TATA binding protein interacting protein 49 kDa variant, partial [Homo sapiens]	gi|62896685	15.5	EACGVTVELIKSKKMAGR	8.482612	10.40493
TBC1 domain family member 1 isoform X4 [Canis lupus familiaris]	gi|545494234	16.120001	LKASENDLLNK	8.580474	7.225362
TBC1 domain family member 30 isoform X2 [Homo sapiens]	gi|578823469	14.41	KSATARNLGLYGPTER	9.634089	8.865027
T-box transcription factor TBX21 [Homo sapiens]	gi|7019549	15.99	AEGSMPGNR	10.58481	11.79553
T-cell activation Rho GTPase-activating protein [Canis lupus familiaris]	gi|73946195	15.31	SPGSPPSYEEAIR	10.49581	8.28259
T-cell receptor alpha chain, partial [Homo sapiens]	gi|170816726	9.96	SMDFKSNS	6.928805	8.857492
T-cell receptor beta chain VJ region, partial [Homo sapiens]	gi|13276408	6.1700001	CVLVQQVLGQGPQFIFQYYEKE	9.865776	11.45003
T-cell receptor beta chain, partial [Homo sapiens]	gi|8217283	18.139999	CASSLLPR	7.949934	8.574015
T-cell receptor beta VJ region, partial [Homo sapiens]	gi|311902884	3.6300001	TVTSAQK	8.720495	9.136148
T-cell receptor delta chain, partial [Homo sapiens]	gi|2239878	7.4099998	TPINSL	6.181555	6.012452
T-cell receptor, partial [Canis lupus familiaris]	gi|303542	18.1	TQKATLVCLAR	13.86451	13.39622
T-cell surface glycoprotein CD3 gamma chain isoform X1 [Homo sapiens]	gi|578822471	5.0900002	DGKMIGFLTEDK	5.820132	8.447078
T-complex protein 1 subunit epsilon isoform 1 [Canis lupus familiaris]	gi|359323746	13.11	QMAEIAVNAVLTVADMQRR	7.172412	6.301468
TCR alpha chain [Homo sapiens]	gi|431831546	11.17	AADTASYFCATPR	5.430779	7.410462
TCR delta chain (VJ), partial [Homo sapiens]	gi|535907	5.0799999	FFLPR	10.44958	10.70519
TCR junctional sequence, partial [Homo sapiens]	gi|1770561	17.66	QLTFGXCLVGR	9.959045	8.67974
TDRD5 protein, partial [Homo sapiens]	gi|59807731	8.8900003	SLLMLK	0	2.93225
teashirt homolog 3 [Homo sapiens]	gi|127138957	7.9699998	AVTDEKPK	8.930591	8.413692
telomerase Cajal body protein 1 [Canis lupus familiaris]	gi|545497990	7.8299999	SAPEAPR	4.612062	7.693794
tenascin isoform X8 [Canis lupus familiaris]	gi|545516486	6.0999999	CVAGR	5.639643	7.316102
tenascin XB, isoform CRA_h [Homo sapiens]	gi|119623984	18.639999	VCRWMARPGPR	9.077137	8.817536
teneurin-1 isoform X1 [Homo sapiens]	gi|578838599	4.9400001	GNRGTESMDTTYSPIGGK	7.958992	8.663863
teneurin-2 isoform X4 [Homo sapiens]	gi|578811189	7.6900001	TDIDKQGRIVSR	4.350222	7.519607
teneurin-4 isoform X1 [Homo sapiens]	gi|578821286	15.73	CGDGGK	8.544878	10.34679
terminal uridylyltransferase 7 isoform X4 [Canis lupus familiaris]	gi|545486398	8.7600002	AAVDLGR	11.31463	10.15307
testicular haploid expressed gene protein isoform X1 [Homo sapiens]	gi|530425265	13.85	RPLASMSLPPPK	8.641155	9.925581
testis spermatogenesis apoptosis-related protein 7 [Homo sapiens]	gi|46241188	15.51	LYMKSLLKIFAWATLR	0	9.028374
testis-specific gene 13 protein [Homo sapiens]	gi|16418411	6.1300001	ATVIG	4.201623	6.924745
testis-specific serine kinase substrate isoform X4 [Canis lupus familiaris]	gi|545487729	7.9400001	KEAEGVGGGEK	7.937842	7.778344
tetratricopeptide repeat protein 16 isoform X4 [Homo sapiens]	gi|578817094	12.33	QDAGILAVQGK	6.988397	4.117603
tetratricopeptide repeat protein 19, mitochondrial isoform 2 [Homo sapiens]	gi|406601116	11.84	ATMSYLLGGGMKQEDNAIIEISI	5.383244	6.631966
tetratricopeptide repeat protein 28 isoform X3 [Canis lupus familiaris]	gi|545544292	19.32	DGTSSLPR	8.936701	7.788469
tetratricopeptide repeat protein 33 isoform X1 [Homo sapiens]	gi|530378752	12.11	MASFGWKRKIGEK	8.632651	9.361495
tetratricopeptide repeat protein 7A isoform X1 [Canis lupus familiaris]	gi|545515512	10.2	RKMFQLKMFR	9.293739	10.0764
TGF-beta-activated kinase 1 and MAP3K7-binding protein 2 isoform X1 [Homo sapiens]	gi|578812557	11.17	SSGPR	6.066811	8.580528
TGF-beta-activated kinase 1 and MAP3K7-binding protein 3 isoform X8 [Canis lupus familiaris]	gi|545557801	20.969999	RVSCTTAIPTSI	6.059915	9.507792
THAP domain containing 4, isoform CRA_a [Homo sapiens]	gi|119591682	18.129999	DASKATGGVRGHSSAATGR	8.406614	9.978382
thiamine transporter 1 isoform 2 [Canis lupus familiaris]	gi|345803276	11.45	QEDAESSSHVTTS	6.949061	4.557734
thioredoxin reductase 1 [Homo sapiens]	gi|119618150	10.09	AAVVLAK	5.856117	6.00856
thioredoxin reductase 2, mitochondrial isoform X1 [Canis lupus familiaris]	gi|545544811	11.42	EAGQQNYDLLVIGGGSGGLACA	8.786232	9.787162
thymocyte nuclear protein 1 isoform X3 [Homo sapiens]	gi|578822161	5.3000002	TSATKNCLK	6.887853	7.189893
tissue-type plasminogen activator [Canis lupus familiaris]	gi|345781617	20.08	CTSRHLFNK	4.127762	7.272387
titin [Canis lupus familiaris]	gi|545555517	29.059999	KTAVTK	9.893085	8.373018
titin isoform novex-3 [Homo sapiens]	gi|110349721	6.3600001	EGEDKELK	9.718513	9.136943
titin isoform X6 [Homo sapiens]	gi|578804834	12.87	KVFEEKIR	9.945238	9.709468
titin isoform X7 [Homo sapiens]	gi|578804836	14.78	EREEEPLFDIDSEMRK	8.751872	8.916549
TLD domain-containing protein 1 isoform X1 [Homo sapiens]	gi|578829106	12.3	LYDGMR	12.16337	11.83805
TMEM72 protein [Homo sapiens]	gi|121933983	11.96	LIITGLAYFLLSKRKK	8.783357	10.1553
TNFAIP3 interacting protein 2, isoform CRA_b, partial [Homo sapiens]	gi|119602921	8.9399996	GYTGN	4.126691	5.313575
TNS4 protein, partial [Homo sapiens]	gi|33878245	8.5	WGLGLYR	5.050239	5.496563
toll-like receptor 2 isoform X1 [Homo sapiens]	gi|530378107	1.14	AAIKS	6.329711	10.6031
tolloid-like 2 [Canis lupus familiaris]	gi|345792616	12.11	LHGKFCGSEMPEVITSQSNNMR	7.03496	4.953493
tomoregulin-1 isoform X2 [Canis lupus familiaris]	gi|545517745	7.77	AACKHQKEITVVARGPCYSDNC	2.594871	6.720161
torsin A interacting protein 2, isoform CRA_d, partial [Homo sapiens]	gi|119611468	20.98	AVWSGENTRRALLILTVLR	7.72855	5.956347
trace amine-associated receptor 5 [Homo sapiens]	gi|223633986	10.25	QAQQITTLSK	11.395	10.97431
trafficking kinesin-binding protein 2 isoform X4 [Canis lupus familiaris]	gi|545555869	12.78	TPDAQENGR	0	7.468112
trans-2-enoyl-CoA reductase, mitochondrial isoform X3 [Canis lupus familiaris]	gi|545491533	15.86	KHEMKNFFK	9.22962	10.28622
transacylase, partial [Homo sapiens]	gi|579832	4.1100001	AAVGM	6.811336	7.46716
transcription elongation factor A (SII), 1, isoform CRA_b [Homo sapiens]	gi|119607146	7.8699999	MSTRIGMSVNAIRK	9.122945	5.368148
transcription elongation factor A N-terminal and central domain-containing protein 2 [Homo sapiens]	gi|23308505	13.34	KDSGGK	7.276161	9.796996
transcription elongation factor A protein-like 4 isoform X1 [Homo sapiens]	gi|530422174	11.03	PAGGQAPQLTPVIPAFWEAR	10.29499	10.43668
transcription elongation factor A protein-like 5 [Canis lupus familiaris]	gi|545558799	8.6199999	KQKMGGFHWMPR	6.117252	0
transcription factor AP-2-beta isoform X3 [Homo sapiens]	gi|578811891	13.47	SVTSLMMNK	8.135431	4.663631
transcription factor AP-2-epsilon isoform X1 [Homo sapiens]	gi|578799017	13.22	SMLLAAKQICK	13.19605	13.07747
transcription factor E2F1 isoform X1 [Canis lupus familiaris]	gi|545540387	6.8600001	ALLSS	8.62412	9.385813
transcription factor HIVEP3 isoform X5 [Homo sapiens]	gi|578799553	12.64	KAEGSPR	9.565201	9.615613
transcription factor RelB isoform X2 [Homo sapiens]	gi|530416980	18.440001	DQQGQMR	7.593767	6.805068
transcription initiation factor TFIID subunit 3 [Canis lupus familiaris]	gi|73949156	6.98	QIQTPPDAGKLNNENQPK	5.571043	8.236434
transcription initiation factor TFIID subunit 4B [Canis lupus familiaris]	gi|345802806	18.040001	AVIAPVQIK	5.97263	6.236519
transcription initiation factor TFIID subunit 4-like [Canis lupus familiaris]	gi|545537239	7.4899998	SRPPTPPPLATGSRPPGRLFR	7.941526	3.551062
transcription termination factor 3, mitochondrial isoform X4 [Homo sapiens]	gi|578815995	3.1700001	MLTANK	9.242715	9.092957
transcriptional repressor p66-alpha isoform X11 [Homo sapiens]	gi|578833351	11.69	SSPEERERMIKQLK	6.433212	9.502103
transcriptional repressor p66-beta isoform X1 [Homo sapiens]	gi|578801317	13.17	SSSRMEER	10.05485	8.427462

transcriptional repressor scratch 1 [Homo sapiens]	gi|525507392	19.969999	SLGSGPGGRGGTR	7.418153	6.169886
transducin beta-like protein 2 isoform X1 [Homo sapiens]	gi|578814101	8.5699997	QDPYLLK	8.395465	10.03529
transforming acidic coiled-coil-containing protein 3 [Canis lupus familiaris]	gi|545494490	18.629999	EAQPPESPQSRPAGPMAKK	10.23414	8.409436
transforming growth factor beta 1 induced transcript 1, isoform CRA_c, partial [Homo sapiens]	gi|119572514	15.28	RQALLPALLPEALRLTAR	11.43702	11.47267
transient receptor potential cation channel, subfamily M, member 4, isoform CRA_d [Homo sapiens]	gi|119572848	8.3900003	LDLLGLL	9.6397	10.34015
transient receptor potential cation channel, subfamily M, member 6, isoform CRA_b [Homo sapiens]	gi|119582958	6.1999998	AVLKGHK	8.552854	4.368316
transketolase isoform X1 [Homo sapiens]	gi|578806532	7.8800001	ESWHGKPLPK	0	3.902407
translation initiation factor eIF-2B subunit gamma isoform 3 [Homo sapiens]	gi|387527981	11.33	EFQAVVMAVGGGSR	9.739508	9.588758
translational activator GCN1 [Canis lupus familiaris]	gi|73994683	17.879999	GMGFLMK	9.106346	8.926552
translin-associated factor X-interacting protein 1 isoform c [Homo sapiens]	gi|573014750	13.04	AAAHQR	5.630942	8.283507
translin-associated factor X-interacting protein 1 isoform X7 [Homo sapiens]	gi|530424123	16.77	KEKMNLLKLIDK	10.7098	10.25395
transmembrane and coiled-coil domains protein 1, partial [Canis lupus familiaris]	gi|545557046	3.24	KXGLPAVR	7.971712	6.393968
transmembrane protease serine 6 [Canis lupus familiaris]	gi|345777094	10.03	QEPVVEVLASGAVMAVVWK	9.592492	8.540242
transmembrane protease serine 9 [Canis lupus familiaris]	gi|545508770	14.52	KMKTGFK	6.893675	6.760015
transmembrane protein 100-like [Canis lupus familiaris]	gi|545503698	4.3400002	EPGAA	8.743398	8.272692
transmembrane protein 101 isoform X2 [Homo sapiens]	gi|578831530	14.43	TVAIIGGFLVLASGAGELYR	10.30567	10.47616
transmembrane protein 155 [Canis lupus familiaris]	gi|545531819	20.16	XNLPR	7.896449	9.711702
transmembrane protein 161B isoform X5 [Homo sapiens]	gi|578810193	5.4499998	KEAGRISTVELQKMVAR	9.925729	8.129492
transmembrane protein 200A [Canis lupus familiaris]	gi|545486029	9.3500004	TGGPK	8.34869	9.253297
transmembrane protein 200B [Homo sapiens]	gi|284520904	7.8000002	DLETRR	9.801056	7.704585
transmembrane protein 214 isoform X5 [Homo sapiens]	gi|578802964	14.82	MATKTAGVGR	11.70875	11.53493
transmembrane protein 25 isoform 3 precursor [Homo sapiens]	gi|221139834	6.3499999	HTLLLLPALLSSVKPEIAQVGAK	0	7.400016
transmembrane protein 253 isoform X1 [Homo sapiens]	gi|578826063	2.0999999	AALLPP	11.70308	10.02573
transmembrane protein 53 [Canis lupus familiaris]	gi|545524043	22.129999	KEVGAR	0	7.77826
transmembrane protein 68 [Canis lupus familiaris]	gi|545548664	6.25	IPGNIMSALLERFHKKQK	8.082745	3.987427
transmembrane protein 86B, isoform CRA_b [Homo sapiens]	gi|119592757	9.1199999	LVIMTTYYAAQLLITLSALR	9.885503	10.10698
transmembrane protein 87B isoform X1 [Homo sapiens]	gi|578805044	9.0799999	AVPELGLWLETVNDKSGPLIFR	7.541448	5.085006
transmembrane protein adipocyte-associated 1 isoform 2 [Homo sapiens]	gi|217416360	4.77	LGAQDPLLLQMPSGRDRGAR	4.248357	5.952809
tricarboxylate transport protein, mitochondrial isoform c [Homo sapiens]	gi|568786339	18.559999	GFFHGVR	4.152285	4.237885
trichohyalin-like, partial [Homo sapiens]	gi|578845873	10	GGVGR	8.907821	9.817756
trimeric intracellular cation channel type A [Homo sapiens]	gi|13129060	9.8500004	KAKKAD	7.546568	8.40651
trinucleotide repeat-containing gene 18 protein isoform X2 [Homo sapiens]	gi|578813686	12.67	SVHGPPPPLLSGLAMDSHR	9.113305	10.41644
tripartite motif-containing protein 49D1-like isoform X2 [Homo sapiens]	gi|578796508	13.11	NLKTNIRLKK	5.732518	7.568249
tripartite motif-containing protein 67 isoform X2 [Homo sapiens]	gi|530366216	15.9	VPPVPLLQLEK	9.17387	10.33078
tripartite motif-containing protein 7 isoform X2 [Canis lupus familiaris]	gi|545516086	10.69	RTGERGESGADLGPR	6.132023	7.257922
triple functional domain protein isoform X2 [Homo sapiens]	gi|578809984	11.87	CVTAVDK	7.498837	9.681934
tRNA (guanine(10)-N2)-methyltransferase homolog isoform X1 [Homo sapiens]	gi|578812921	5.0599999	HFIGNTSMDAGLSFIMANHGKV	5.959513	5.574644
tRNA methyltransferase 10 homolog B isoform X8 [Homo sapiens]	gi|578816380	19.43	HWEKIVAAK	5.271495	0
tropomyosin alpha-1 chain isoform X20 [Homo sapiens]	gi|578827357	9.6499996	ALMAAEDK	7.794186	7.160794
truncated aniridia protein, partial [Homo sapiens]	gi|7690062	1.91	GGLYER	5.351627	6.963859
truncated ganglioside differentiation associated protein 1 [Homo sapiens]	gi|320461577	9.7799997	QGRRGG	5.849613	0
T-type calcium channel alpha H1, partial [Canis lupus familiaris]	gi|27228316	13.78	DPMQPAPVTDTAGQGSTRQR	7.726994	11.47979
tubulin polymerization-promoting protein family member 2 isoform X4 [Canis lupus familiaris]	gi|545524221	8.3299999	ATTVGGVSR	9.218656	8.820701
tubulin-folding cofactor B [Canis lupus familiaris]	gi|545488789	8.6899996	IGRVGSRGR	0	7.614667
tudor domain containing 6 [Homo sapiens]	gi|119624705	11.3	PAPGASLALR	9.09698	11.11233
tudor domain-containing protein 3 [Canis lupus familiaris]	gi|345805859	27.9	SVLEGSGLPRNRGSK	5.342618	5.131607
tumor necrosis factor (ligand) superfamily, member 13b, isoform CRA_c, partial [Homo sapiens]	gi|119629506	15.56	NKVAALQGDLASLR	5.887737	9.985959
tumor necrosis factor alpha-induced protein 8-like protein 2 [Homo sapiens]	gi|157389001	26.030001	ICDGLRKLLDEGK	10.53957	13.20911
tumor protein D55 [Canis lupus familiaris]	gi|545517011	9.6300001	HTLAAKERRCTELK	4.772785	5.982351
tumor protein D55 isoform 2 [Homo sapiens]	gi|49574212	11.89	SATLRSFEGLIFNK	9.344196	8.555862
tumor protein p53-inducible nuclear protein 2 isoform X2 [Homo sapiens]	gi|578836103	15.32	ESRPR	9.145411	6.825304
tVEGFR-1, partial [Homo sapiens]	gi|114186835	3.4200001	EGATA	8.328597	8.695352
type I hair keratin 5 [Homo sapiens]	gi|3724107	9.6700001	MYSSSSCKLPSLSPVAR	6.246099	7.614481
type I keratin K16, partial [Homo sapiens]	gi|1000377	24.530001	LASYPDK	8.476689	8.881016
type II inositol 3,4-bisphosphate 4-phosphatase isoform X1 [Homo sapiens]	gi|578809591	8.6899996	ALDCMRREGCRIENVLK	5.839501	10.77308
type-1 angiotensin II receptor isoform 2 [Homo sapiens]	gi|395455058	16.83	YIPPK	3.698745	3.69049
type-2 angiotensin II receptor [Canis lupus familiaris]	gi|545559094	13.19	KSNITLATISK	6.753979	7.063962
tyrosine-protein kinase CSK isoform X1 [Homo sapiens]	gi|530405396	26.58	EGIIPANYVQK	10.93365	8.923843
tyrosine-protein kinase Fer isoform X3 [Homo sapiens]	gi|578810247	10.61	NCIHRDLAAR	7.39593	6.703129
tyrosine-protein kinase STYK1 [Canis lupus familiaris]	gi|359323112	12.72	RDVMTMNSLLYDLTEK	3.187009	10.1346
Tyrosine-protein kinase transmembrane receptor ROR2 precursor variant, partial [Homo sapiens]	gi|62087888	13.03	VAGNPPPNVRWLK	6.50267	11.46821
tyrosine-protein phosphatase non-receptor type 12 isoform X3 [Homo sapiens]	gi|578814469	8.5100002	GHAIKIKSASPCIADK	6.307364	7.845595
tyrosine-protein phosphatase non-receptor type 3 isoform X4 [Homo sapiens]	gi|578817687	20.120001	MLRGGYSAIVMTSR	6.316581	7.294774
tyrosine-protein phosphatase non-receptor type 7 isoform 3 [Homo sapiens]	gi|315221143	14.84	GSNVALMLDVR	2.876029	6.607784
tyrosyl-DNA phosphodiesterase 2 [Canis lupus familiaris]	gi|545554799	20.950001	FKSQEIIPFPNTKMMR	9.547032	10.58704
U3 small nucleolar RNA-associated protein 14 homolog A isoform X2 [Canis lupus familiaris]	gi|545559307	8.5500002	TSQALSK	7.905251	6.276468
U6 snRNA-associated Sm-like protein LSm4 isoform 2 [Homo sapiens]	gi|355477275	15.43	GMGGAGRGVFGGR	6.849439	6.20161
U621B_HUMAN RecName: Full=UPF0621 protein B	gi|206557954	1.66	AGTAA	8.791888	6.659128
UAP56-interacting factor isoform 1 [Homo sapiens]	gi|42716295	19.74	IDMSLDDIIK	11.0794	10.28216
ubiquitin carboxyl-terminal hydrolase 16 isoform b [Homo sapiens]	gi|50312664	2.71	MSGTIVK	8.432477	8.861503

ubiquitin carboxyl-terminal hydrolase 24 isoform X3 [Homo sapiens]	gi|578798779	9.5500002	CSTANSR	0	4.169628
ubiquitin carboxyl-terminal hydrolase 36 isoform X3 [Homo sapiens]	gi|530412598	13.37	ADVLSGENAYMCAKCKKK	6.827418	6.939277
ubiquitin carboxyl-terminal hydrolase 37 isoform X10 [Homo sapiens]	gi|578804665	18.07	SLGFLPQPVPLSVKKLR	10.35886	11.38855
ubiquitin carboxyl-terminal hydrolase 42 isoform X1 [Homo sapiens]	gi|578813668	8.04	AAQSVNK	4.897939	6.34091
ubiquitin carboxyl-terminal hydrolase 47 [Canis lupus familiaris]	gi|545536825	8.96	MMDLEEVIPMDCCR	9.393353	9.413874
ubiquitin specific peptidase 28, isoform CRA_e [Homo sapiens]	gi|119587639	3.1300001	YINDK	6.075485	9.855385
ubiquitin thioesterase OTU1 isoform X1 [Canis lupus familiaris]	gi|57089291	21.389999	MSGPAKGGHFGVPR	9.607099	9.588213
ubiquitin-conjugating enzyme E2 O isoform X2 [Homo sapiens]	gi|530412648	14.61	IGNTEDGAPHK	9.802917	9.780581
ubiquitin-like modifier-activating enzyme 5 isoform X1 [Homo sapiens]	gi|530375300	13.46	VELANMNR	0	5.707277
ubiquitin-protein ligase E3A isoform X1 [Homo sapiens]	gi|578827377	2.27	GFGML	7.825876	9.134865
ubiquitously transcribed tetratricopeptide repeat protein Y-linked transcript variant 13 [Homo sapiens]	gi|148733150	13.5	CYXNAAR	6.444706	0
ubiquitously transcribed tetratricopeptide repeat protein Y-linked transcript variant 139 [Homo sapiens]	gi|157829419	29.9	AVRCYESLILKXEGK	6.428362	9.100398
ubiquitously transcribed tetratricopeptide repeat protein Y-linked transcript variant 200 [Homo sapiens]	gi|151946827	13.78	ACRNPGKNGLSNSXILLDK	10.62211	10.11009
ubiquitously transcribed tetratricopeptide repeat protein Y-linked transcript variant 276 [Homo sapiens]	gi|151946727	17.370001	TKTLLGXLLINQK	8.607717	8.014705
ubiquitously transcribed tetratricopeptide repeat protein Y-linked transcript variant 38 [Homo sapiens]	gi|148733166	10.64	CPPPRPPTSPXPPLPKDK	9.856647	9.40736
ubiquitously transcribed tetratricopeptide repeat protein Y-linked transcript variant 73 [Homo sapiens]	gi|157829431	10.24	AVRCYESLILXAEGK	9.360467	4.360274
UBX domain-containing protein 1 isoform X1 [Homo sapiens]	gi|530396858	18.43	RMLELVAQK	5.689117	7.402222
UDP-GalNAc:beta-1,3-N-acetylgalactosaminyltransferase 1 isoform X7 [Canis lupus familiaris]	gi|545553942	17.030001	QDFRFTLR	4.629203	11.25493
UDP-glucose:glycoprotein glucosyltransferase 2 isoform X3 [Canis lupus familiaris]	gi|345788594	18.26	ARAADVVR	6.973867	6.096572
UDP-glucuronosyltransferase 3A2 isoform 2 precursor [Homo sapiens]	gi|270132420	13.75	KVKET	7.596592	8.240274
UHRF1-binding protein 1-like isoform X3 [Homo sapiens]	gi|578823458	15.11	MSSSVVVR	8.533066	8.187737
uncharacterized aarF domain-containing protein kinase 2 isoform X2 [Homo sapiens]	gi|578814728	17.469999	CSGAAGAGPAESLPR	10.3271	9.687325
uncharacterized protein C10orf118 isoform X1 [Canis lupus familiaris]	gi|545548008	14.31	VKIVDQIQEQHQRGK	8.004777	9.175117
uncharacterized protein C10orf95 isoform X2 [Canis lupus familiaris]	gi|545536765	6.5500002	LGSPQPRGPGDGRLRAR	7.642849	8.585621
uncharacterized protein C11orf80 [Canis lupus familiaris]	gi|545531556	13.19	QLVISMEALNSK	7.454957	6.566903
uncharacterized protein C16orf59 homolog isoform X3 [Canis lupus familiaris]	gi|545502569	11.61	AASQNSR	8.929684	9.430717
uncharacterized protein C16orf95 isoform 1 [Homo sapiens]	gi|304376255	13.22	FGGRLPMPR	5.429144	7.335696
uncharacterized protein C16orf96 isoform X3 [Homo sapiens]	gi|530408022	15.14	KDGAPK	9.655204	11.40239
uncharacterized protein C17orf104 homolog isoform X4 [Canis lupus familiaris]	gi|545510331	20.07	IQSVNHMEGLTK	2.762115	9.040833
uncharacterized protein C1orf168 isoform X3 [Homo sapiens]	gi|530362046	1.63	ENLRT	7.853916	7.828383
uncharacterized protein C1orf50 homolog [Canis lupus familiaris]	gi|545523354	5.54	HDAKINIVDK	9.666845	9.223343
uncharacterized protein C20orf195 homolog isoform X1 [Canis lupus familiaris]	gi|545541363	22.610001	TMNLQVTGLGLDK	11.66976	10.14228
uncharacterized protein C20orf203 [Canis lupus familiaris]	gi|545541496	13.06	EVGRGSQGPAGRAQGRK	9.228613	8.627321
uncharacterized protein C22orf46 homolog isoform X1 [Canis lupus familiaris]	gi|345776802	13.06	VHVGGENDMQTQVSR	8.73424	7.928503
uncharacterized protein C2orf16-like isoform X2 [Canis lupus familiaris]	gi|545560721	12.95	TSSLGQPMGIVQNDSSSKGKK	9.990901	5.267558
uncharacterized protein C2orf82 homolog [Canis lupus familiaris]	gi|359322809	25.110001	KPELHLFAVEKKTRGGTVCAPV	10.02592	4.343577
uncharacterized protein C5orf66 [Homo sapiens]	gi|474451706	5.8099999	MGITPWPK	8.630628	3.412777
uncharacterized protein C6orf99 isoform X3 [Homo sapiens]	gi|530383294	6.6199999	LGLQNRWRGMK	4.113334	6.523807
uncharacterized protein C7orf26 homolog isoform X3 [Canis lupus familiaris]	gi|545501083	12.86	QILSASPR	0	6.840656
uncharacterized protein C8orf31 isoform X1 [Homo sapiens]	gi|578839747	12.13	GSCTHRAQGLLAARTTALQR	5.416099	10.05856
uncharacterized protein C9orf129 isoform X1 [Homo sapiens]	gi|578817493	4.77	LSAPG	3.913077	3.802728
uncharacterized protein CXorf30 [Homo sapiens]	gi|167003762	12.37	EMTCIALDSTCIEIPLSNPK	7.897051	5.23745
uncharacterized protein CXorf58 isoform X3 [Canis lupus familiaris]	gi|545557713	16.17	QALMNVEKMKK	6.79816	11.34279
uncharacterized protein encoded by ZNF503-AS2 [Canis lupus familiaris]	gi|545495396	19.27	RNRIEGVTRR	5.608667	6.162318
uncharacterized protein KIAA1210 [Canis lupus familiaris]	gi|545560126	15.3	ERSSPVEPKPPR	8.855711	11.92147
uncharacterized protein KIAA1328 homolog isoform X5 [Canis lupus familiaris]	gi|545505859	13.47	EAGAWNHSTRR	8.152962	7.217762
uncharacterized protein LOC101060085 [Homo sapiens]	gi|410171184	4.1799998	GSTAQKVR	4.10394	1.936212
uncharacterized protein LOC101927371 [Homo sapiens]	gi|530394743	7.0799999	TQGMNSASLLKIK	2.455578	6.929416
uncharacterized protein LOC101928498 [Homo sapiens]	gi|530437126	8.0600004	AAVESQRKEAQGAGEAR	8.861401	10.17749
uncharacterized protein LOC101928941 [Homo sapiens]	gi|530439024	11	ELDSIF	6.031442	6.012901
uncharacterized protein LOC102151348 [Canis lupus familiaris]	gi|545552424	18.15	GPRMEAATPERGPR	7.295507	8.282363
uncharacterized protein LOC102151755 isoform X4 [Canis lupus familiaris]	gi|545554011	18.66	ATDAR	7.063337	11.37931
uncharacterized protein LOC102153217 [Canis lupus familiaris]	gi|545535552	27.1	GAATWLPGR	10.6747	10.64576
uncharacterized protein LOC102154085 [Canis lupus familiaris]	gi|545530402	12.94	AGSMAGSVHQGNGRAPGMR	12.16488	9.849541
uncharacterized protein LOC102155181 isoform X1 [Canis lupus familiaris]	gi|545546427	8.71	EGEERCGKNYSSHRGAAHGPLS	0	7.681248
uncharacterized protein LOC102156775 [Canis lupus familiaris]	gi|545525114	13.79	ADAGSAPPPASGAPR	8.754478	9.694098
uncharacterized protein LOC284861-like [Canis lupus familiaris]	gi|545520333	15.42	AGRGAASSRER	11.27734	9.969927
uncharacterized protein LOC284861-like [Canis lupus familiaris]	gi|545537217	18.66	AGGPWAR	11.28883	10.8115
uncharacterized protein LOC475957 [Canis lupus familiaris]	gi|545529939	6.1799998	IFENGK	4.815017	6.214525
uncharacterized protein LOC607427 [Canis lupus familiaris]	gi|545508906	15.55	QDIDED	10.78351	8.858729
unconventional myosin-Ia [Homo sapiens]	gi|4885503	14.85	LQQLATLIQK	3.470926	4.844531
unconventional myosin-IXa isoform X5 [Canis lupus familiaris]	gi|74000953	17.75	LPLRFNDALVLR	7.881278	8.624962
unconventional myosin-X isoform X1 [Homo sapiens]	gi|578809923	11.83	LMKAYISMIVK	4.251488	5.150735
unconventional myosin-XV isoform X1 [Homo sapiens]	gi|530410390	17.98	KDGGK	4.655076	4.929631
unconventional myosin-XVI [Canis lupus familiaris]	gi|545538166	18.41	DGNGNVALR	6.307831	8.641362
Unknown (protein for IMAGE:30530665), partial [Homo sapiens]	gi|51858976	9.6099997	GPVSICLSAGNGFSR	8.602327	8.62775
Unknown (protein for MGC:176400) [Homo sapiens]	gi|223461298	10	AVESVARLGYLFR	11.53227	6.999742
unknown [Homo sapiens]	gi|82791473	25.74	MSGQTERLQK	8.657335	9.532929

unknown [Homo sapiens]	gi|763429	9.2299995	LSPGV	10.91566	9.88755
unknown, partial [Homo sapiens]	gi|2345091	16.059999	QAEGRSSGTAGDAGSLPR	0	7.526418
unknown, partial [Homo sapiens]	gi|2852640	12.84	ETSGPAPGGSLPR	10.15174	7.683823
unnamed protein product [Homo sapiens]	gi|32458	11.2	YDGLVGMFDPQR	3.743165	0
unnamed protein product [Homo sapiens]	gi|194389262	4.2399998	GNNICEGGEEMDNK	8.16553	0
unnamed protein product [Homo sapiens]	gi|194381768	12.38	KEDGTPAATGGSQPPSMGR	8.303342	0
unnamed protein product [Homo sapiens]	gi|221043208	10.35	LSMQKSTQNHAAVFRVGSLLQE	7.609986	3.128098
unnamed protein product [Homo sapiens]	gi|28801461	11.68	CAALGAR	6.884367	3.143029
unnamed protein product [Homo sapiens]	gi|27900563	5.6399999	DGDGKIGVDEFSTLVAEXKKH	8.045392	3.190825
unnamed protein product [Homo sapiens]	gi|15141749	19.07	AMDNFHAELHPR	8.172777	3.432998
unnamed protein product [Homo sapiens]	gi|194381714	12.07	AVTGMNGRIVATKPLYVALAQI	4.844627	3.596842
unnamed protein product [Homo sapiens]	gi|189053793	7.6599998	KMAVKEGK	5.864945	4.475274
unnamed protein product [Homo sapiens]	gi|14042326	5.1799998	QEDSVTELTVEDSGESLEDLMA	8.447515	4.668263
unnamed protein product [Homo sapiens]	gi|28800826	4.0300002	GEEGGK	4.447067	4.702351
unnamed protein product [Homo sapiens]	gi|221044848	8.6400003	GSAGA	5.706018	5.074562
unnamed protein product [Homo sapiens]	gi|28802547	11.44	GGRQAPEAESEATCKQMSR	4.512051	5.391256
unnamed protein product [Homo sapiens]	gi|221045510	19.67	IALLEEARKK	7.855272	5.468361
unnamed protein product [Homo sapiens]	gi|194378860	8.1300001	SPVGL	6.098703	5.689691
unnamed protein product [Homo sapiens]	gi|189053342	4.6300001	ERLAPFRQREGKPYLIDLAGSED	10.61361	5.764686
unnamed protein product [Homo sapiens]	gi|194375714	3.1300001	MAAGT	7.294455	5.876397
unnamed protein product [Homo sapiens]	gi|28800997	10.43	LLTSGDLPTSASR	7.009426	5.901131
unnamed protein product [Homo sapiens]	gi|40047126	7.1100001	EXMGCR	7.82548	5.993106
unnamed protein product [Homo sapiens]	gi|21757148	14.12	KSLLAGR	6.619235	6.092591
unnamed protein product [Homo sapiens]	gi|40034003	15.06	EQPGAPAGSQR	1.62982	6.195254
unnamed protein product [Homo sapiens]	gi|21439064	20.530001	VARKNTEGALDLLKK	5.976847	6.215657
unnamed protein product [Homo sapiens]	gi|221041362	7.27	VATVAVLWVGKALR	7.547393	6.229263
unnamed protein product [Homo sapiens]	gi|158259651	10.45	MSLTFRRPK	3.116883	6.309964
unnamed protein product [Homo sapiens]	gi|189065178	14.83	DFGAEPMGEKPVGSLAGIGEVL(	6.768698	6.372082
unnamed protein product [Homo sapiens]	gi|22761752	5.5799999	NNLAA	8.335007	6.37285
unnamed protein product [Homo sapiens]	gi|194381302	10.62	ASVSPTRASQAWSPSMSACWST	8.355234	6.406793
unnamed protein product [Homo sapiens]	gi|194374141	7.7199998	DQKLRRMMGVPEIR	0	6.612126
unnamed protein product [Homo sapiens]	gi|194388768	6.52	EREVMESTPSRGLNR	9.663218	6.646873
unnamed protein product [Homo sapiens]	gi|21752739	16.290001	LSCTVSGFAR	7.645381	6.717435
unnamed protein product [Homo sapiens]	gi|40047082	15.83	XSVALQVGDVR	7.00488	6.752004
unnamed protein product [Homo sapiens]	gi|194386076	27.780001	GVEKAKAMPSPR	6.666403	6.756675
unnamed protein product [Homo sapiens]	gi|194374659	4.7600002	GERIT	6.503002	6.892608
unnamed protein product [Homo sapiens]	gi|40035592	17.879999	CLASQER	6.360956	6.961637
unnamed protein product [Homo sapiens]	gi|21752110	6.6199999	QVGGVH	8.360374	6.964904
unnamed protein product [Homo sapiens]	gi|193784115	8.6599998	MEPLQGHATPALPFK	7.473066	6.998749
unnamed protein product [Homo sapiens]	gi|34534467	9.0799999	QIEGRTILSEGYGPLASAAC	5.83698	7.012789
unnamed protein product [Homo sapiens]	gi|194378372	11.8	MWTKRMSLVTAGVSCVFVMHI	8.953802	7.032908
unnamed protein product [Homo sapiens]	gi|194386834	7.8099999	RTLSLAPSSSKCTSCM	9.080769	7.08223
unnamed protein product [Homo sapiens]	gi|32693508	8.9700003	SPEALLRGR	7.202861	7.294229
unnamed protein product [Homo sapiens]	gi|80747843	22.07	MALSAVSELITSK	6.759064	7.300524
unnamed protein product [Homo sapiens]	gi|158260387	10.31	FESLTMGSKKK	5.250507	7.393853
unnamed protein product [Homo sapiens]	gi|34527143	12.24	MSLDSGALK	8.693608	7.402154
unnamed protein product [Homo sapiens]	gi|27900099	10.23	GAAALHK	6.47821	7.492566
unnamed protein product [Homo sapiens]	gi|28802584	6.6799998	FAVMDLEAGARTLPLQMVLRG/	9.191859	7.5115
unnamed protein product [Homo sapiens]	gi|28799780	11.35	GEEXAGAGTGTTEGK	9.352797	7.576602
unnamed protein product [Homo sapiens]	gi|194380318	18.77	MIFCTVR	10.74491	7.659054
unnamed protein product [Homo sapiens]	gi|221043988	30.48	KRQTVKQGPK	8.257215	7.697203
unnamed protein product [Homo sapiens]	gi|221040750	5.3699999	GALPP	8.128165	7.717695
unnamed protein product [Homo sapiens]	gi|194374963	10.33	EQQDLESAKEMAEDDDDSFP	10.20563	7.720922
unnamed protein product [Homo sapiens]	gi|40046684	16.66	XNLMVNMMSLDVK	10.59687	7.764204
unnamed protein product [Homo sapiens]	gi|194380728	9.8599997	MIIDASGESGLTQLLMTEVMK	7.239505	7.797579
unnamed protein product [Homo sapiens]	gi|34526326	19.02	AINYIEMER	6.153626	7.82316
unnamed protein product [Homo sapiens]	gi|34527000	8.9099998	VRVLAPVFRICSPLPR	0	7.836419
unnamed protein product [Homo sapiens]	gi|221045664	14.23	SKGDTGVEGQEFIVRGWLHKEV	9.332981	7.87414
unnamed protein product [Homo sapiens]	gi|193786271	13.5	FGEVHLEFTMSK	6.313444	7.999937
unnamed protein product [Homo sapiens]	gi|28802185	10.74	AGVGGGGGFXWGGGLGACR	9.430457	8.108011
unnamed protein product [Homo sapiens]	gi|40038769	13.07	TGGPSLFGGPGGER	7.114525	8.10953
unnamed protein product [Homo sapiens]	gi|193785872	16.469999	MKTDGATVK	7.512987	8.126534
unnamed protein product [Homo sapiens]	gi|4587129	9.3000002	AVVLQSQNQMSEAHK	10.57559	8.133846
unnamed protein product [Homo sapiens]	gi|40038656	12.64	SASPGPASCFPR	8.4401	8.156178
unnamed protein product [Homo sapiens]	gi|59669020	15.04	MQLXGK	7.959928	8.183835
unnamed protein product [Homo sapiens]	gi|34535506	11.69	MLDLTMTR	10.98545	8.261659
unnamed protein product [Homo sapiens]	gi|194384984	10.91	GNSKGQEELGRGAR	9.453139	8.314813
unnamed protein product [Homo sapiens]	gi|194375512	11.52	KLHCEVEAISR	4.439966	8.320183
unnamed protein product [Homo sapiens]	gi|194384718	9.96	GVLTGLR	8.707096	8.339869
unnamed protein product [Homo sapiens]	gi|194375249	6.0900002	AEAGT	6.869369	8.390342
unnamed protein product [Homo sapiens]	gi|194379222	15.44	ERKTFALPGIIKK	12.27561	8.407093
unnamed protein product [Homo sapiens]	gi|34531724	71.010002	YLTWASWQEPSQGTTTFAVTSI	7.396816	8.593779
unnamed protein product [Homo sapiens]	gi|28800899	27.389999	LSLALSPR	8.129061	8.598239
unnamed protein product [Homo sapiens]	gi|28801736	20.23	KGAGKGTDAGGPMLLK	10.32014	8.68594
unnamed protein product [Homo sapiens]	gi|14036124	16.059999	MEILWLMVK	4.527676	8.793058

unnamed protein product [Homo sapiens]	gi|13397236	10.86	VSVAIAFVGGSR	0	8.802056
unnamed protein product [Homo sapiens]	gi|34531961	9.8100004	MRPKSHGLR	9.339775	8.82845
unnamed protein product [Homo sapiens]	gi|34527453	11.11	TMKQLWFFLLLVAAPR	10.21324	8.91445
unnamed protein product [Homo sapiens]	gi|40046586	7.9499998	MXMSVSK	6.614644	8.972735
unnamed protein product [Homo sapiens]	gi|28590	25.41	VPEVSTPTLVEVSR	9.368199	8.979859
unnamed protein product [Homo sapiens]	gi|21758373	12.27	MWICPGGGGGGGGGGGGGGGI	8.046529	8.983075
unnamed protein product [Homo sapiens]	gi|23342725	10.96	MDGDR	9.254786	9.02756
unnamed protein product [Homo sapiens]	gi|34531311	23.700001	SLGSRCHR	8.63822	9.040223
unnamed protein product [Homo sapiens]	gi|40046848	7.5	DCAXIVTQKK	0	9.07577
unnamed protein product [Homo sapiens]	gi|194382934	10.22	GLSPARPRVGEGR	4.480992	9.083976
unnamed protein product [Homo sapiens]	gi|194385778	30.76	CDGTGTGDVDCR	6.767526	9.097828
unnamed protein product [Homo sapiens]	gi|40046456	14.55	KXIER	6.910034	9.143977
unnamed protein product [Homo sapiens]	gi|22760608	7.6199999	EALCCTA	9.678313	9.15463
unnamed protein product [Homo sapiens]	gi|34529082	10.39	LHHPVDQLPHQVLQPTTGQR	10.97422	9.184176
unnamed protein product [Homo sapiens]	gi|21899852	5.8899999	HGAGPK	10.47611	9.362375
unnamed protein product [Homo sapiens]	gi|194374613	16.15	MVGAAFMKK	9.776047	9.37466
unnamed protein product [Homo sapiens]	gi|40046696	14.92	KGTANAXPSG	7.924633	9.383229
unnamed protein product [Homo sapiens]	gi|28799739	7	MYFFVSK	8.725801	9.384457
unnamed protein product [Homo sapiens]	gi|194385500	13.53	RSLATSPCTSLQR	11.01682	9.4477
unnamed protein product [Homo sapiens]	gi|194377938	18.719999	SSNAYGGGRGLNSSNNSHGR	9.852042	9.468964
unnamed protein product [Homo sapiens]	gi|7023699	26.93	MKLLLDMYRSAPK	9.289468	9.482976
unnamed protein product [Homo sapiens]	gi|21749880	16.66	STGMLHGGR	4.823947	9.503403
unnamed protein product [Homo sapiens]	gi|194386298	18.139999	MDEPTITDLNTR	9.25742	9.552408
unnamed protein product [Homo sapiens]	gi|194379666	19.43	QIAITTAK	8.785848	9.639712
unnamed protein product [Homo sapiens]	gi|28071068	14.31	LRLTSMPRPSLTR	11.30491	9.675048
unnamed protein product [Homo sapiens]	gi|40047156	21.139999	KSLLSXALAKSGER	8.799406	9.756112
unnamed protein product [Homo sapiens]	gi|7020591	4.6799998	ASDLS	7.288626	9.777794
unnamed protein product [Homo sapiens]	gi|194382260	12.64	SPPACFLEAGLPSVPR	9.11072	9.860614
unnamed protein product [Homo sapiens]	gi|56675551	14.16	EREKGGXR	7.909086	9.870098
unnamed protein product [Homo sapiens]	gi|34535739	26.790001	KSLALSPR	10.09849	9.904718
unnamed protein product [Homo sapiens]	gi|194380266	11.82	RAGIKSTAFGMNGLGGIAAKLFA	9.285568	9.919839
unnamed protein product [Homo sapiens]	gi|40037698	13.19	SPGQAPDTCSPSPR	8.668337	9.947954
unnamed protein product [Homo sapiens]	gi|221046168	16.700001	MALPLRDR	12.43182	9.974185
unnamed protein product [Homo sapiens]	gi|21758011	11.48	ILGLLQPLPPRFK	12.44221	9.993072
unnamed protein product [Homo sapiens]	gi|10438313	6.21	MLVMR	9.547646	10.0385
unnamed protein product [Homo sapiens]	gi|221040858	9.1000004	AHWPAASR	8.06998	10.05765
unnamed protein product [Homo sapiens]	gi|10433842	15.48	MDMSWQLR	11.29911	10.12122
unnamed protein product [Homo sapiens]	gi|34526765	19.629999	SSLSYGLEAR	8.642011	10.23577
unnamed protein product [Homo sapiens]	gi|221046284	13.8	MPSVFGK	11.18762	10.30503
unnamed protein product [Homo sapiens]	gi|28800595	15.7	ALENKTGGLCPR	11.09736	10.47778
unnamed protein product [Homo sapiens]	gi|327516055	14.7	GGGGSGGGGSGGGGSR	9.385662	10.50352
unnamed protein product [Homo sapiens]	gi|221042308	8.2399998	ESECTLLASCLQFSTPAP	1.48016	10.65679
unnamed protein product [Homo sapiens]	gi|193785963	24.15	LSDDHVNEIIIQK	13.76339	10.78991
unnamed protein product [Homo sapiens]	gi|194374837	27.92	CGPAGEIR	11.38682	10.98254
unnamed protein product [Homo sapiens]	gi|218468898	26.879999	IATAXMLGIRK	6.230194	11.08599
unnamed protein product [Homo sapiens]	gi|40047104	13.1	TSTLQXVRIER	8.66895	11.1038
unnamed protein product [Homo sapiens]	gi|3980130	11.14	MSKQQASQVLVR	8.528152	11.24582
unnamed protein product [Homo sapiens]	gi|221042242	9.6999998	VAFSNTS	9.05153	11.27123
unnamed protein product [Homo sapiens]	gi|27900061	17.9	KVNXLRAFVK	10.91619	11.33667
unnamed protein product [Homo sapiens]	gi|194375574	6.27	MKTLYLADTFPTNFRDSAGAMI	10.50775	11.60764
unnamed protein product [Homo sapiens]	gi|193785003	13.27	KRGAAGSPGAAPSASR	9.401218	11.95864
unnamed protein product [Homo sapiens]	gi|194376146	16.99	MLRASGLTQK	11.42534	12.14389
unnamed protein product [Homo sapiens]	gi|21439424	20.959999	GDVTITAVR	10.18362	12.3897
unnamed protein product [Homo sapiens]	gi|158259413	9.79	TMWSRGDKAIMEGSGR	5.323667	12.40918
unnamed protein product, partial [Canis lupus familiaris]	gi|21538359	7.1399999	HHIVW	5.294636	6.447469
unnamed protein product, partial [Homo sapiens]	gi|40042734	8.6499996	GPAGXGK	5.424545	0
unnamed protein product, partial [Homo sapiens]	gi|218347496	4.9699998	TCGPRSMAAAR	5.492964	0
unnamed protein product, partial [Homo sapiens]	gi|40039260	14.42	VVYGSR	6.95277	0
unnamed protein product, partial [Homo sapiens]	gi|40042882	7.9899998	YDIFDPR	8.709718	0
unnamed protein product, partial [Homo sapiens]	gi|40979160	14.45	HSRXWDFR	10.12257	0
unnamed protein product, partial [Homo sapiens]	gi|40975830	16.139999	DAIDGLXGMR	7.235257	2.361636
unnamed protein product, partial [Homo sapiens]	gi|40981438	14.6	AGVACK	4.284656	4.009387
unnamed protein product, partial [Homo sapiens]	gi|40040118	6.6500001	AMAGS	4.14176	4.193988
unnamed protein product, partial [Homo sapiens]	gi|40043588	13.38	MSDSXEQNYGER	7.998518	4.308456
unnamed protein product, partial [Homo sapiens]	gi|29333343	6.4499998	DSLTCPQSLVCVGLR	4.186405	4.41538
unnamed protein product, partial [Homo sapiens]	gi|40044542	16.549999	VAAQNLIXNTNIDNGQK	8.034026	4.517948
unnamed protein product, partial [Homo sapiens]	gi|40978340	6.3299999	VQAAXK	0	4.548312
unnamed protein product, partial [Homo sapiens]	gi|40982368	9.3500004	MGGGTR	4.492209	4.639047
unnamed protein product, partial [Homo sapiens]	gi|40981908	14.87	GGRVIAPXKAR	3.974415	4.758547
unnamed protein product, partial [Homo sapiens]	gi|40977584	5.7399998	MTQQSF	3.461926	4.813849
unnamed protein product, partial [Homo sapiens]	gi|40975592	6.3299999	EPSXHPV	6.78215	4.980352
unnamed protein product, partial [Homo sapiens]	gi|194374517	11.6	KPLSPP	5.838747	5.072031
unnamed protein product, partial [Homo sapiens]	gi|40041292	3.45	XTMEGR	3.478396	5.212604
unnamed protein product, partial [Homo sapiens]	gi|40041854	14.94	KMGVXYCIIKGK	2.840344	5.281646
unnamed protein product, partial [Homo sapiens]	gi|40980298	5.3299999	MSDLGR	2.473865	5.347681

unnamed protein product, partial [Homo sapiens]	gi|40039242	13.96	KCSESSDSGSGFWKALTFMAVG	12.13526	5.360297
unnamed protein product, partial [Homo sapiens]	gi|40982314	12.92	HMDLSGKRXGNK	7.762156	5.824393
unnamed protein product, partial [Homo sapiens]	gi|40045132	1.24	XGEVNA	0	5.866741
unnamed protein product, partial [Homo sapiens]	gi|40045840	8.6700001	NMTXAEK	0	5.972366
unnamed protein product, partial [Homo sapiens]	gi|40042598	9.5799999	GXRGNTE	6.455666	6.061171
unnamed protein product, partial [Homo sapiens]	gi|40043468	12.42	MAASLXGK	9.489565	6.063656
unnamed protein product, partial [Homo sapiens]	gi|40044658	13.2	MIIXIK	2.264309	6.1125
unnamed protein product, partial [Homo sapiens]	gi|40980998	4.4000001	TIAPP	7.83357	6.244129
unnamed protein product, partial [Homo sapiens]	gi|40982020	9.9700003	XDGKEPSDKPQKAVQDHK	0	6.248456
unnamed protein product, partial [Homo sapiens]	gi|40045962	16.469999	QGGSGAGPAK	2.096714	6.365237
unnamed protein product, partial [Homo sapiens]	gi|40041814	21.73	GEIFXLK	9.401396	6.441391
unnamed protein product, partial [Homo sapiens]	gi|40043472	15.58	RVXCLVGAGISTSAGNP	9.299544	6.527078
unnamed protein product, partial [Homo sapiens]	gi|40982366	6.4899998	NYAKSILPVLYK	6.421844	6.560477
unnamed protein product, partial [Homo sapiens]	gi|40980440	11.51	MVMNGLNAMSPR	11.12005	6.599104
unnamed protein product, partial [Homo sapiens]	gi|40981084	10.49	VDASGSVASLSVGEXTGVRAPV	7.783064	6.612871
unnamed protein product, partial [Homo sapiens]	gi|40044008	20.49	LPGSLHSPASXSR	9.719105	6.615141
unnamed protein product, partial [Homo sapiens]	gi|40981466	16.83	VGXGK	6.037601	6.677905
unnamed protein product, partial [Homo sapiens]	gi|40041782	10.96	MDEDGLPLMGSGIDLTXVPAIQ	8.280145	6.779037
unnamed protein product, partial [Homo sapiens]	gi|40045308	23.889999	MPLXPLR	1.869836	6.798043
unnamed protein product, partial [Homo sapiens]	gi|40977350	4.7800002	AVSTD	7.469928	6.802789
unnamed protein product, partial [Homo sapiens]	gi|40044566	8.7700005	DLGAEKAPXPSR	0	6.883411
unnamed protein product, partial [Homo sapiens]	gi|40043646	12.59	DGLDTRMTGAPXRTSRHSQEPF	9.056563	6.928973
unnamed protein product, partial [Homo sapiens]	gi|40040634	12.24	QXLAEHK	0	6.94314
unnamed protein product, partial [Homo sapiens]	gi|40043438	12.06	MXMREAR	7.176615	6.973684
unnamed protein product, partial [Homo sapiens]	gi|40045330	16.09	MKETLQNVXTR	3.002764	6.982403
unnamed protein product, partial [Homo sapiens]	gi|40042374	6.0599999	MHVDTILE	8.237352	7.10876
unnamed protein product, partial [Homo sapiens]	gi|40044636	10.37	KLDIGFR	8.538481	7.134776
unnamed protein product, partial [Homo sapiens]	gi|40982590	10.97	ANILMR	3.681005	7.182169
unnamed protein product, partial [Homo sapiens]	gi|40045700	4.9699998	GTSGPR	5.699925	7.20365
unnamed protein product, partial [Homo sapiens]	gi|40979690	16.450001	KIVTK	6.747243	7.322968
unnamed protein product, partial [Homo sapiens]	gi|40979844	15.66	MLFISAPNIR	6.221047	7.38119
unnamed protein product, partial [Homo sapiens]	gi|40979300	4.2800002	KQNQNTXK	9.130058	7.419115
unnamed protein product, partial [Homo sapiens]	gi|40980048	5.4000001	XTVAWAR	7.917501	7.445517
unnamed protein product, partial [Homo sapiens]	gi|40980166	9.7700005	ESIRKNS	8.611845	7.451317
unnamed protein product, partial [Homo sapiens]	gi|40977148	8.1899996	MASIXSK	6.159455	7.513461
unnamed protein product, partial [Homo sapiens]	gi|40981350	8.4799995	XGMVFSSLR	0	7.633719
unnamed protein product, partial [Homo sapiens]	gi|40039348	9.8599997	QXLMGSFSIT	8.474216	7.783753
unnamed protein product, partial [Homo sapiens]	gi|40045466	15.04	AGPLSPSFRAALCR	11.47199	7.789621
unnamed protein product, partial [Homo sapiens]	gi|32592	8.0600004	ATPIE	10.13347	7.814607
unnamed protein product, partial [Homo sapiens]	gi|221042958	6.8899999	TPSDRTV	8.056579	7.858781
unnamed protein product, partial [Homo sapiens]	gi|40043048	13.35	SFMPNLXPPKIPDGER	5.731937	8.018826
unnamed protein product, partial [Homo sapiens]	gi|40981358	24.07	MDTTTGLGTLLKXPRTEQCGRA	7.562616	8.023751
unnamed protein product, partial [Homo sapiens]	gi|56675591	15.35	GLQEVTPGQGRLQXSLK	6.197662	8.163304
unnamed protein product, partial [Homo sapiens]	gi|40040084	6.8600001	MEAAT	8.012753	8.226183
unnamed protein product, partial [Homo sapiens]	gi|148354350	13.46	ATCNCNLHAR	4.567582	8.263086
unnamed protein product, partial [Homo sapiens]	gi|194387736	12.34	EQTFDSQLEKVHSPFRR	8.404073	8.280603
unnamed protein product, partial [Homo sapiens]	gi|40978314	18.48	GEIRKMAR	8.86865	8.474819
unnamed protein product, partial [Homo sapiens]	gi|40980638	20.08	AAAMALKVTGK	10.22531	8.497166
unnamed protein product, partial [Homo sapiens]	gi|40041984	4.7399998	KSSLVXSK	7.214154	8.551494
unnamed protein product, partial [Homo sapiens]	gi|39103793	10.1	GMNNSR	9.374084	8.558514
unnamed protein product, partial [Homo sapiens]	gi|40042654	13.71	MXLAAR	8.123222	8.571327
unnamed protein product, partial [Homo sapiens]	gi|40040128	12.23	TPTTXAPSASVPWPR	9.266997	8.573084
unnamed protein product, partial [Homo sapiens]	gi|40977704	11.21	XIVEK	9.183154	8.641938
unnamed protein product, partial [Homo sapiens]	gi|40043624	21.620001	MELYXIGGGQDVR	9.905847	8.678626
unnamed protein product, partial [Homo sapiens]	gi|40980872	19.969999	KISSINIM	9.119942	8.689896
unnamed protein product, partial [Homo sapiens]	gi|40976334	4.9099998	MAGKGGK	7.33411	8.692135
unnamed protein product, partial [Homo sapiens]	gi|40041664	8.4700003	ANIQAVXLK	7.703359	8.792919
unnamed protein product, partial [Homo sapiens]	gi|221044110	19.08	MVASFNRGAR	10.29857	8.832087
unnamed protein product, partial [Homo sapiens]	gi|56675572	7.4499998	MXDFDRFKVMK	7.147532	8.862551
unnamed protein product, partial [Homo sapiens]	gi|40977764	18.860001	EVVEAENXR	9.149624	8.893101
unnamed protein product, partial [Homo sapiens]	gi|40041104	7.77	AISVLAKWQXSYSIK	9.721121	8.895468
unnamed protein product, partial [Homo sapiens]	gi|40975714	9.4300003	EKPDDPLNYFLGGCAGXLTLGA	9.830174	8.923165
unnamed protein product, partial [Homo sapiens]	gi|10434221	10.08	LATSMAGKTDRKEFVR	11.6601	8.980346
unnamed protein product, partial [Homo sapiens]	gi|40045198	10.86	MAXIKKSGT	10.10354	8.989308
unnamed protein product, partial [Homo sapiens]	gi|40039948	11.82	RGFGXCTAQKGEACMLLR	8.469915	8.999727
unnamed protein product, partial [Homo sapiens]	gi|40040998	14.91	FAMXSRRGPHVPVGHNAPKDLI	11.11554	9.048692
unnamed protein product, partial [Homo sapiens]	gi|40043138	6.0300002	QKFTRASSTSCAPSISPAMKLQL	7.448972	9.16419
unnamed protein product, partial [Homo sapiens]	gi|40040872	11.62	MDKMAAAXVQGGR	12.59885	9.314695
unnamed protein product, partial [Homo sapiens]	gi|40981212	2.05	EXVES	9.066428	9.332495
unnamed protein product, partial [Homo sapiens]	gi|28799498	10.2	VCAGGPR	7.529383	9.334846
unnamed protein product, partial [Homo sapiens]	gi|40041740	13.61	GTQAPRLAENFCVCHLATGXMI	10.45348	9.33972
unnamed protein product, partial [Homo sapiens]	gi|40976674	15.06	MSPRRGTWLPR	8.973367	9.344552
unnamed protein product, partial [Homo sapiens]	gi|40982142	20.77	PLILQFR	10.70194	9.398717
unnamed protein product, partial [Homo sapiens]	gi|56675599	15.98	KIMGGSGTEXTLEK	9.1769	9.506326
unnamed protein product, partial [Homo sapiens]	gi|40045318	5.4099998	GADGKTKIAXPRGAAPPGQK	6.486	9.567777

unnamed protein product, partial [Homo sapiens]	gi|40042832	17.889999	MASVXLSEAEK	0	9.606595
unnamed protein product, partial [Homo sapiens]	gi|40980524	9.2200003	ALKNGVPSPIMATKIPXK	10.45649	9.611301
unnamed protein product, partial [Homo sapiens]	gi|40977868	7.0700002	HMVQDCTAVKTXLLK	10.52336	9.624654
unnamed protein product, partial [Homo sapiens]	gi|40043700	13.25	VVGMAPGLPR	9.080249	9.682762
unnamed protein product, partial [Homo sapiens]	gi|40981164	16.629999	QMLKLDKENXNRPR	6.937353	10.04802
unnamed protein product, partial [Homo sapiens]	gi|56675582	8.5799999	TRCGDXSSPGLGAAGRESFPLGI	7.067648	10.18183
unnamed protein product, partial [Homo sapiens]	gi|40040280	21.219999	WSLSLSPR	9.656178	10.2015
unnamed protein product, partial [Homo sapiens]	gi|21438519	7.54	VLGAA	10.27694	10.20493
unnamed protein product, partial [Homo sapiens]	gi|40040884	15.77	MDXLVSECSAR	8.820166	10.21378
unnamed protein product, partial [Homo sapiens]	gi|40975950	13.39	XSSDPAWAVEWIELPR	11.73052	10.2167
unnamed protein product, partial [Homo sapiens]	gi|40040624	6.0799999	XAINK	6.489874	10.46968
unnamed protein product, partial [Homo sapiens]	gi|40979490	11.86	KPXPAPSPMRAANR	6.012524	10.92982
unnamed protein product, partial [Homo sapiens]	gi|40039390	4.6999998	YGIVLDAGSSHTSLYIYKWPAXI	8.856164	11.04268
unnamed protein product, partial [Homo sapiens]	gi|40045662	11	XKLGV	9.338819	11.22106
unnamed protein product, partial [Homo sapiens]	gi|40041064	14.2	GDEVMVELA	10.97337	11.34289
unnamed protein product, partial [Homo sapiens]	gi|40042380	11.5	MHLVTNSGKMVVLDK	9.988729	11.55289
unnamed protein product, partial [Homo sapiens]	gi|40976542	18.18	DCKMADGK	10.06952	11.56743
unnamed protein product, partial [Homo sapiens]	gi|40978172	14.53	MAGVAATYGTKPLSCR	11.19989	11.66482
unnamed protein product, partial [Homo sapiens]	gi|40982792	18.620001	MTGLSMDGGGXPR	10.34571	11.71068
UPF0249 protein ydjC homolog isoform X1 [Homo sapiens]	gi|530419550	21.959999	AAVGPFSRHGLR	9.452125	8.558155
UPF0378 protein KIAA0100 isoform X2 [Homo sapiens]	gi|578831699	13.12	KQQEDVSVVR	9.901053	12.33349
UPF0538 protein C2orf76 homolog isoform X1 [Canis lupus familiaris]	gi|545532010	10.03	QDIPLRTSLPPPFR	11.09591	12.68258
UPF0598 protein C8orf82 [Homo sapiens]	gi|49169841	16.59	MWPPCGTLRTLALARSR	7.527273	10.98478
UPF0607 protein ENSP00000383144 [Homo sapiens]	gi|578845458	12.2	WDRDEGPPPAK	9.498836	9.680073
UPF0609 protein C4orf27 homolog isoform X5 [Canis lupus familiaris]	gi|545542241	21.66	SASLNFNLHWR	7.992389	14.13247
UPF0688 protein C1orf174 homolog [Canis lupus familiaris]	gi|73956697	18.25	SELQKLAPQSDR	11.98202	9.054404
UPF0696 protein C11orf68 homolog [Canis lupus familiaris]	gi|359321918	11.3	MAAAAAVAGAGRGGGGGGG	6.33585	10.83696
UPF0762 protein C6orf58 isoform X2 [Canis lupus familiaris]	gi|545486007	20.33	IFEDRLEYYSK	9.829614	9.44048
up-regulated during skeletal muscle growth protein 5-like [Canis lupus familiaris]	gi|545485620	14.7	MNCVLATYGGIALK	10.36764	9.904879
up-regulator of cell proliferation-like isoform X7 [Canis lupus familiaris]	gi|545560590	16.4	VRSIVAHVAR	4.144619	8.571869
upstream binding transcription factor, RNA polymerase I, isoform CRA_b [Homo sapiens]	gi|119572003	18.139999	MTMRMKIMSPR	9.890233	10.16247
upstream regulatory element binding protein 1 [Homo sapiens]	gi|3694922	10.06	MIISREMFNPMYALFR	7.189957	8.472289
urocanate hydratase isoform X3 [Canis lupus familiaris]	gi|545532420	5.0300002	AALMK	9.387424	11.78925
uroplakin-1a isoform 2 [Homo sapiens]	gi|527498307	17.889999	AASNTSATPSTATRGVSR	7.939535	7.334447
uroplakin-2 [Canis lupus familiaris]	gi|73955054	7.6599998	GSSTESSR	6.818764	7.560986
Usher syndrome type-1C protein-binding protein 1 isoform X1 [Homo sapiens]	gi|530415234	10.34	KMDGGSGR	8.73058	9.323584
USP6 N-terminal like isoform X3 [Canis lupus familiaris]	gi|545490012	16.540001	YPSPLDGEDR	10.3568	9.231441
utrophin isoform X8 [Homo sapiens]	gi|578812959	14.65	TSEISIPADLDK	1.26188	9.123588
UV-stimulated scaffold protein A isoform X1 [Homo sapiens]	gi|578808194	12.21	ARIGRKVFAK	12.27637	10.42246
vacuolar protein sorting-associated protein 13B isoform X8 [Homo sapiens]	gi|578815845	8.8500004	TEEMQPTVEANQAAKEDTVVL	8.499849	4.312826
vacuolar protein sorting-associated protein 13C isoform 2A [Homo sapiens]	gi|66347828	12.62	KEGAAGFFKGIGK	9.871762	10.5245
vacuolar protein sorting-associated protein 41 homolog isoform 1 [Homo sapiens]	gi|114199475	17.639999	DSLVKILQDYNLQILLREGCK	7.642828	7.775274
vacuolar protein sorting-associated protein 4B [Canis lupus familiaris]	gi|545485143	4.8699999	AVDLASK	7.569554	3.783888
vacuolar protein sorting-associated protein 54 isoform X1 [Homo sapiens]	gi|578802939	10.87	LVAIMDSLFDK	8.377737	5.12462
vascular endothelial growth factor (VEGF) receptor, partial [Homo sapiens]	gi|1155009	12.89	MQSKVLLAVALWLCVETRAAS	11.0271	8.041545
VCY2 interacting protein-1 [Homo sapiens]	gi|22002953	9.25	TPRELR	7.876482	9.156637
ventricular zone-expressed PH domain-containing protein homolog 1 isoform 1 [Homo sapiens]	gi|269847558	22.23	RDRSLPR	8.697051	10.97667
very-long-chain (3R)-3-hydroxyacyl-[acyl-carrier protein] dehydratase 2 [Canis lupus familiaris]	gi|345796233	26.809999	TDAALEPSRTLGR	6.152948	9.41327
vinculin isoform X2 [Homo sapiens]	gi|530394330	6.6599998	VAMANIQPQMLVAGATSIAR	8.226592	7.648032
visinin-like peptide 1 [Homo sapiens]	gi|755161	10.45	VDKIFSKMDXNKDDQITLGEFR	8.730279	12.32654
visual system homeobox 2 [Homo sapiens]	gi|34365783	19.48	AGEALSKPK	7.52013	8.451673
voltage-dependent calcium channel subunit alpha-2/delta-3 isoform X2 [Homo sapiens]	gi|530372847	5.8099999	MAGPGSPR	8.285152	8.877593
voltage-dependent N-type calcium channel subunit alpha-1B [Canis lupus familiaris]	gi|545513521	8.6800003	LFFVMGEDKVIXK	6.651	6.012222
voltage-dependent P/Q type calcium channel alpha 1A subunit, partial [Homo sapiens]	gi|3873285	7.1399999	SPRMERR	2.038694	6.218893
voltage-dependent R-type calcium channel subunit alpha-1E isoform 1 [Homo sapiens]	gi|329663504	10.25	SVQPSNHGIYLPSDTQEHAGSGI	10.34509	14.60224
voltage-dependent T-type calcium channel subunit alpha-1H isoform X8 [Homo sapiens]	gi|578828330	17.74	AADEVR	9.510847	9.557041
voltage-gated calcium channel, partial [Homo sapiens]	gi|951207	9.04	YIPKNXHQYK	8.634529	7.613376
von Willebrand factor A domain-containing protein 3B isoform X3 [Homo sapiens]	gi|578803895	18.02	AEDGR	8.701795	10.03186
von Willebrand factor A domain-containing protein 8 [Canis lupus familiaris]	gi|545537547	9.9700003	SLMEWRNMIGQGDR	6.293311	9.637741
V-type proton ATPase subunit S1 [Canis lupus familiaris]	gi|74008854	10.51	AAMAAARVLAGTRRAPALR	10.68179	10.05879
V-type proton ATPase subunit S1 precursor [Homo sapiens]	gi|17136148	7.46	MAAMATARVRMGPR	11.50717	8.481939
WAS/WASL-interacting protein family member 3-like [Homo sapiens]	gi|530380345	5	ASFGR	10.1116	0
WD repeat and HMG-box DNA-binding protein 1 [Canis lupus familiaris]	gi|545507415	14.85	KAWASKAKGGTTSDGAEAK	7.79838	8.823853
WD repeat domain 16, isoform CRA_d [Homo sapiens]	gi|119610433	12.13	GVTMK	10.28503	9.573118
WD repeat domain 17 [Homo sapiens]	gi|119625128	10.49	FGGGIGVPTK	7.307991	8.055099
WD repeat-containing protein 11 isoform X2 [Homo sapiens]	gi|530393966	1.92	QGAVLFASKAGAAGK	3.937109	4.847311
WD repeat-containing protein 6 [Homo sapiens]	gi|197927448	13.24	MGSAAR	6.591753	8.771203
WD repeat-containing protein 60 isoform X1 [Homo sapiens]	gi|578814386	9.8199997	AACQVMAVLLEEDR	2.201826	8.833588
WD repeat-containing protein 65 [Canis lupus familiaris]	gi|359321386	19.459999	DILGLK	9.999066	8.921988
WD repeat-containing protein 78 [Canis lupus familiaris]	gi|345800386	15.06	EPGTEEPADFLK	6.184595	6.022718
WD repeat-containing protein 87 [Canis lupus familiaris]	gi|545489346	9.75	KVISVALGK	9.157825	4.648842
WD repeat-containing protein 90 isoform X6 [Homo sapiens]	gi|578828026	19.379999	GAVTGLTATPDGR	6.493262	9.859622
wiskott-Aldrich syndrome protein family member 2-like [Homo sapiens]	gi|530434484	12.42	EPSLAARGTAPPAPPR	3.086064	7.860154
WIZ protein, partial [Homo sapiens]	gi|38566138	4.96	TPLNLSSRAEPVRDIR	0	6.97999

WWP3, partial [Homo sapiens]	gi|2072505	6.3899999	IEDPSPGI	8.226409	7.770788
xin actin-binding repeat-containing protein 1 isoform X1 [Canis lupus familiaris]	gi|545538481	5.5599999	DGVKGDVK	8.314963	6.434156
xin actin-binding repeat-containing protein 2 isoform X3 [Canis lupus familiaris]	gi|545555096	3.95	EAATPR	9.428456	9.311019
X-linked retinitis pigmentosa GTPase regulator isoform X2 [Homo sapiens]	gi|578838049	10.28	ETKLAEIAGMKDLR	8.4911	5.806266
ZFAT-3 [Homo sapiens]	gi|45504126	11.27	TRDEFLVMKR	9.177878	8.607675
zinc finger and BTB domain-containing protein 17 [Canis lupus familiaris]	gi|545492318	13.4	GEVREDEGAGAAEVK	9.357942	10.00388
zinc finger and BTB domain-containing protein 2 [Canis lupus familiaris]	gi|545485643	8.3500004	LASAPEKLGREPRPQAPR	2.879349	10.0565
zinc finger and BTB domain-containing protein 26 [Homo sapiens]	gi|18141299	4.9200001	QPMDSK	5.275258	8.897193
zinc finger and BTB domain-containing protein 45 isoform X1 [Homo sapiens]	gi|578835051	16.360001	HLLAARPPGHPGAAHSRK	9.555382	11.35469
zinc finger and BTB domain-containing protein 5 isoform X1 [Canis lupus familiaris]	gi|545517622	14.93	RVTASSSEQGGGARLR	10.4496	10.13009
zinc finger and SCAN domain-containing protein 1 isoform X7 [Canis lupus familiaris]	gi|545487095	13.88	ALASPR	8.937281	7.208872
zinc finger and SCAN domain-containing protein 2 isoform X1 [Homo sapiens]	gi|530407183	4.9699998	MAADIPR	7.977919	7.777999
zinc finger and SCAN domain-containing protein 26 isoform c [Homo sapiens]	gi|566006125	14.77	MATALDPDQPKK	10.70459	9.946372
zinc finger and SCAN domain-containing protein 30 [Canis lupus familiaris]	gi|545505886	16.66	QQDTAHGQGMIWK	8.445221	11.55046
zinc finger and SCAN domain-containing protein 5A isoform X1 [Homo sapiens]	gi|578834884	13.32	KRGLESR	6.922016	7.505662
zinc finger CCCH domain-containing protein 13 isoform X1 [Homo sapiens]	gi|530402208	14.8	KKKEDDVGIER	10.2092	11.96744
zinc finger CCCH domain-containing protein 14 isoform X3 [Canis lupus familiaris]	gi|545508292	13.94	TRTSQEELLAEMVQGQSRTPR	12.55374	6.927826
zinc finger CCCH domain-containing protein 18 isoform X2 [Homo sapiens]	gi|530424666	11.53	KKLGVSVSPSR	5.838509	5.504948
zinc finger CCCH domain-containing protein 3 isoform X8 [Homo sapiens]	gi|578815910	21.5	ELRYGLGGPQVSPLAK	8.769132	8.171711
zinc finger CCCH domain-containing protein 4 isoform X1 [Homo sapiens]	gi|578834188	14.56	EGSSRGRGSR	6.488416	10.86472
zinc finger CCCH-type antiviral protein 1 isoform X1 [Homo sapiens]	gi|530386233	5.9400001	AYVESICSNNFDSFLHETHENK	9.732483	10.54034
zinc finger CCHC domain-containing protein 14 isoform X1 [Homo sapiens]	gi|530423713	10.6	QPSMDFNRPGKRAPWPRPPGSR	3.619616	6.935681
zinc finger CCHC domain-containing protein 4 isoform X4 [Homo sapiens]	gi|530376298	1.46	LTASGDKK	0	7.272091
zinc finger CCHC domain-containing protein 7 [Canis lupus familiaris]	gi|545517615	15.42	DAEAQIVNNRSSGR	6.731128	6.495229
zinc finger FYVE domain-containing protein 21 isoform X1 [Canis lupus familiaris]	gi|73964171	9.8500004	MSSEVAARRDAK	8.037059	9.29903
zinc finger homeobox protein 3 isoform X1 [Homo sapiens]	gi|530423920	5.4299998	NTPREVSPLLPK	10.90225	10.16238
zinc finger homeobox protein 4 [Canis lupus familiaris]	gi|345793145	16.98	LLMTVPVPDVMMPNSLLLPAAA	5.320685	9.65159
zinc finger homeobox protein 4 [Homo sapiens]	gi|291167749	14.87	CNVVFPR	9.219391	6.969781
zinc finger MYM-type protein 6 [Homo sapiens]	gi|115511046	9.2799997	KKPVVTIYTKSISTKCSMCQK	7.355228	4.830184
zinc finger protein 101 isoform X1 [Homo sapiens]	gi|578833738	11	KLIVEK	7.946619	7.989845
zinc finger protein 106 isoform X2 [Homo sapiens]	gi|530406294	6.98	MVRERK	8.54966	8.087876
zinc finger protein 112 [Canis lupus familiaris]	gi|545489313	9.6899996	KEPVMFK	7.512623	9.453387
zinc finger protein 138, isoform CRA_e [Homo sapiens]	gi|119598389	3.6199999	MTSSGR	10.33172	8.823575
zinc finger protein 14 [Homo sapiens]	gi|38045952	13.58	SFSWSISLRLHER	9.122633	8.229146
zinc finger protein 14 homolog isoform X1 [Homo sapiens]	gi|530416944	25	EPGMVVR	5.894667	0
zinc finger protein 157 isoform X1 [Canis lupus familiaris]	gi|545557913	3.1400001	PANGK	2.855112	9.789964
zinc finger protein 181 isoform X4 [Homo sapiens]	gi|578834388	5.9000001	VNGGK	0	5.670215
zinc finger protein 232 isoform X3 [Homo sapiens]	gi|578830032	8.2799997	KEPSH	10.74827	10.30542
zinc finger protein 280C isoform X2 [Homo sapiens]	gi|578838699	16.360001	GITLVCLKCDFLADSSGLDR	13.82591	9.993841
zinc finger protein 318 isoform X1 [Canis lupus familiaris]	gi|345778461	10.34	TVVSLGK	7.492252	6.415061
zinc finger protein 319 isoform X1 [Homo sapiens]	gi|530424154	17.93	QQYALMR	9.422258	8.378456
zinc finger protein 32 isoform X2 [Homo sapiens]	gi|530392695	4.6900001	MTEAHHK	10.54553	10.60463
zinc finger protein 384 isoform X6 [Homo sapiens]	gi|578822575	10.22	VASTLTEEGGGGGGGGSVAPK	11.66172	11.58252
zinc finger protein 426 isoform X3 [Canis lupus familiaris]	gi|545534806	10.34	EKPSEFNQRDK	4.325573	7.384063
zinc finger protein 438 isoform X2 [Canis lupus familiaris]	gi|545489740	19.290001	GGSGDPPPPATSDR	10.46723	8.809987
zinc finger protein 462 isoform X4 [Homo sapiens]	gi|578817729	8.1499996	RERWCDHMMK	6.204179	8.059709
zinc finger protein 511, isoform CRA_b, partial [Homo sapiens]	gi|119581735	15.56	GGPATRLPR	4.740906	5.508292
zinc finger protein 516 [Homo sapiens]	gi|7662010	11.07	SGSSPLGVVTK	13.54	10.1024
zinc finger protein 518A isoform a [Homo sapiens]	gi|57242807	19.360001	WEDFSNVDSPMMPR	5.985323	6.419568
zinc finger protein 532 [Canis lupus familiaris]	gi|545485190	9.0500002	SPESQSLIDGTK	6.405616	2.835267
zinc finger protein 546-like isoform X2 [Canis lupus familiaris]	gi|545487549	11.43	IRIHK	9.408395	10.63603
zinc finger protein 568 isoform 2 [Homo sapiens]	gi|325651956	6.7600002	ASLSIHK	7.419601	0
zinc finger protein 569 isoform X6 [Homo sapiens]	gi|578834032	13.15	IASLALHMRSHTGEKPYKCDK	9.073823	9.263625
zinc finger protein 598 [Homo sapiens]	gi|409264581	10.51	CSTKMR	7.007197	0
zinc finger protein 609 [Homo sapiens]	gi|71725360	9.2700005	MSLSSGASGGK	8.445717	0
zinc finger protein 613 isoform X1 [Homo sapiens]	gi|530417276	24.6	GNLLIHRR	8.876867	8.645325
zinc finger protein 638 isoform X9 [Canis lupus familiaris]	gi|545528278	17.6	KIEDSSLGK	11.45772	9.471617
zinc finger protein 644 isoform X7 [Homo sapiens]	gi|578799886	17.07	TGAGMVEVTSLLK	2.298436	5.992652
zinc finger protein 654 isoform X4 [Homo sapiens]	gi|578806342	15.46	QTISLPVSTSK	12.4528	9.42693
zinc finger protein 683 isoform X5 [Canis lupus familiaris]	gi|545491715	10.81	AGSIDEKLKAKYPASR	8.128524	8.047789
zinc finger protein 687 isoform X3 [Homo sapiens]	gi|578801323	15.84	THGMAFIR	7.038654	7.883351
zinc finger protein 728 isoform X1 [Homo sapiens]	gi|578833255	15.54	RIHTGEKPCK	8.370886	3.166065
zinc finger protein 761-like [Homo sapiens]	gi|530392636	11.21	MKIGNAGK	7.723122	8.213308
zinc finger protein 831 isoform X1 [Homo sapiens]	gi|578835748	7.71	EAMAGKGRAGGR	0	6.189744
zinc finger protein 836 [Homo sapiens]	gi|156627573	13.16	MHTGDKPYK	7.766491	0
zinc finger protein 862 isoform X1 [Homo sapiens]	gi|578814504	16.42	VAEAGGQIGHRAK	8.52667	8.005153
zinc finger protein 91 isoform X4 [Homo sapiens]	gi|578833582	13.58	HTIRHTGK	5.472088	4.105297
zinc finger protein 92 homolog isoform X1 [Homo sapiens]	gi|530422737	10.72	HRIIHSGEKPYACPECGKLFRR	7.5281	4.90617
zinc finger protein Aiolos isoform 14 [Homo sapiens]	gi|548962174	6.6199999	MGSER	9.362218	7.509937
zinc finger protein GLIS1 isoform X1 [Homo sapiens]	gi|530361876	6.4200001	TPGSEK	7.076053	8.077594
zinc finger protein Helios isoform X7 [Homo sapiens]	gi|578803924	12.65	LTGNMGK	10.98328	9.363226
zinc finger protein PLAGL1 isoform X1 [Canis lupus familiaris]	gi|57032122	13.66	MATYPCQLCGK	5.495391	7.634895
zinc finger protein, partial [Homo sapiens]	gi|2522510	3.76	EFILKKDLMNVMNAGKPS	4.615253	7.508847
zinc finger protein, partial [Homo sapiens]	gi|2522502	14.95	GINVMSVGRRSVR	5.07368	8.294606
zinc finger SWIM domain-containing protein 4 isoform X1 [Homo sapiens]	gi|530415089	26.280001	SIHCAPMLSDILR	8.609653	8.842832

zinc finger SWIM domain-containing protein 6 [Homo sapiens]	gi|210147522	9.21	MLTLITEQFMADPRLSLWR	7.36231	8.747474
zinc finger SWIM domain-containing protein 6 isoform X1 [Homo sapiens]	gi|578810477	17.030001	IATLMDSLPDITLLK	8.361967	12.3774
zinc finger SWIM domain-containing protein 8 isoform X4 [Homo sapiens]	gi|578819165	19.700001	GLGEGVPSSQRGPR	9.489726	8.193486
zinc finger transcription factor snail 2 [Canis lupus familiaris]	gi|146231220	12.5	MARSFLVK	8.86643	8.416326
zinc finger X-linked protein ZXDB [Canis lupus familiaris]	gi|545558276	19.27	AASRGPGLSPLAPR	6.776846	10.68276
zinc finger ZZ-type and EF-hand domain-containing protein 1 isoform X1 [Canis lupus familiaris]	gi|545497646	14	LGVQGLSISGYVRPMR	7.421321	8.701254
zinc finger ZZ-type and EF-hand domain-containing protein 1 isoform X4 [Homo sapiens]	gi|578829796	7.6300001	WNSMTGVK	4.618971	5.128797
zinc finger, MYND-type containing 19, partial [Homo sapiens]	gi|119608808	22.65	TAAGNSLRR	9.359644	9.982458
zinc transporter 7 isoform X1 [Homo sapiens]	gi|530361871	6.6900001	FNLFGK	4.647623	7.111493
zinc-alpha-2-glycoprotein precursor [Homo sapiens]	gi|4502337	63.32	HVEDVPAFQALGSLNDLQFFR	3.767911	5.942929
zona pellucida 2 glycoprotein [Canis lupus familiaris]	gi|633050	18.35	QPIYLEVK	9.816149	8.720595
zonadhesin [Canis lupus familiaris]	gi|545501225	7.0599999	QPMTPTKK	12.32014	9.347647
zymogen granule protein 16 homolog B [Canis lupus familiaris]	gi|73959451	26.030001	VATSTFGLMK	11.06256	9.692552
zymogen granule protein 16 homolog B precursor [Homo sapiens]	gi|94536866	51	LGALGGNTQEVTLQPGEYITK	9.435779	8.720604
ZZ-type zinc finger-containing protein 3 isoform X4 [Homo sapiens]	gi|530362339	13.32	LVFDKVGLPARPK	3.83621	5.418766
