# Supplementary material for: Comparative proteomic study of dog and human saliva
Source: PLoS One. 2018 Dec 4;13(12):e0208317. doi: 10.1371/journal.pone.0208317 (PMC6279226; doi:10.1371/journal.pone.0208317)
Supplement: S2 Table — (PDF) [file pone.0208317.s002.pdf]

Supplementary data 2 : Proteins found only in dogs

Protein name	Accession number	ID Score	Peptide	DOG	HUMAN
hCG2036717 [Homo sapiens]	gi|119571781	15.28	KDNKHLLM	3.55149	0
protein FAM110A [Homo sapiens]	gi|46391104	11.34	GPADGGARKPSAVER	3.615547	0
unnamed protein product [Homo sapiens]	gi|32458	11.2	YDGLVGMFDPQR	3.743165	0
armadillo repeat-containing protein 2 isoform X2 [Homo sapiens]	gi|578812995	4.3699999	IILALK	3.775933	0
DNA damage-induced apoptosis suppressor protein [Homo sapiens]	gi|194239694	11.35	IILVSK	3.916139	0
coronin 6, isoform CRA_d [Homo sapiens]	gi|119571592	1.48	GGAHK	4.111305	0
alternative protein ZNF644 [Homo sapiens]	gi|440576095	12.25	KSLMPR	4.173955	0
collagen alpha-1(I) chain-like [Canis lupus familiaris]	gi|545489812	8.96	GAGGTAGQQR	4.479076	0
histone H2A type 2-A [Homo sapiens]	gi|106775678	14.9	VTIAQGGVLPNIQAVLLPK	4.701959	0
calcineurin-binding protein cabin-1 isoform X11 [Homo sapiens]	gi|578837053	10.56	VSSMLQRTPDQGKK	4.702742	0
hCG2007771 [Homo sapiens]	gi|119630298	16.77	LGIGGIGRRLWR	4.79344	0
E3 ubiquitin-protein ligase XIAP isoform X1 [Homo sapiens]	gi|578838669	12.7	MTFNSFEGSK	5.164847	0
PHD finger protein 3 isoform X1 [Homo sapiens]	gi|578812606	24.07	QNMTTDAPKKIVAAK	5.181479	0
biorientation of chromosomes in cell division protein 1-like 1 isoform X2 [Homo sapiens]	gi|578808374	4.0599999	KREVSPPGARTR	5.204606	0
intracellular hyaluronan-binding protein 4 isoform X1 [Homo sapiens]	gi|530390749	3.6700001	AVVIHK	5.226727	0
tRNA methyltransferase 10 homolog B isoform X8 [Homo sapiens]	gi|578816380	19.43	HWEKIVAAK	5.271495	0
protein-methionine sulfoxide oxidase MICAL3 isoform X15 [Homo sapiens]	gi|578836826	18.43	AQIIQR	5.338539	0
unnamed protein product, partial [Homo sapiens]	gi|40042734	8.6499996	GPAGXGK	5.424545	0
unnamed protein product, partial [Homo sapiens]	gi|218347496	4.9699998	TCGPRSMAAAR	5.492964	0
nibrin isoform X2 [Homo sapiens]	gi|530389034	12.12	KLSSAVVFGGGEAR	5.51483	0
suppressin [Homo sapiens]	gi|3293442	7.5100002	IHADAKR	5.585447	0
hCG2007967, partial [Homo sapiens]	gi|119593232	25.809999	MRSQNAALSGSGPR	5.587793	0
dedicator of cytokinesis protein 10 isoform X10 [Homo sapiens]	gi|578804561	5.8099999	ANHSVAR	5.58928	0
Ig H-chain, partial [Homo sapiens]	gi|185315	17.42	GITGTT	5.635132	0
truncated ganglioside differentiation associated protein 1 [Homo sapiens]	gi|320461577	9.7799997	QGRRGG	5.849613	0
zinc finger protein 14 homolog isoform X1 [Homo sapiens]	gi|530416944	25	EPGMVVR	5.894667	0
A Chain A, Dbl And Pleckstrin Homology Domains From Hsos1	gi|6729800	12.6	KXNEIQK	5.926031	0
transcription elongation factor A protein-like 5 [Canis lupus familiaris]	gi|545558799	8.6199999	KQKMGGFHWMPR	6.117252	0
CCR4-NOT transcription complex subunit 3-like [Homo sapiens]	gi|530436103	10.79	APDARLALGSAR	6.231001	0
gap junction delta-4 protein [Homo sapiens]	gi|145699105	7.4899998	GSGSEEQPSAAPSR	6.403089	0
ubiquitously transcribed tetratricopeptide repeat protein Y-linked transcript variant 13 [Homo sapiens]	gi|148733150	13.5	CYXNAAR	6.444706	0
polymerase delta-interacting protein 2 isoform 1 [Homo sapiens]	gi|7661672	7.0900002	GRGVVGREPVLSK	6.749978	0

mitochondrial 10-formyltetrahydrofolate dehydrogenase precursor [Homo sapiens]	gi|238814322	10.35	EESFGPIMVISK	6.758795	0
hypothetical protein FLJ37396, isoform CRA_a [Homo sapiens]	gi|119568743	16.620001	NKNMKDDDLSMK	6.767944	0
non-histone chromosomal protein HMG-17-like [Canis lupus familiaris]	gi|545520510	12.92	QIRHRKLK	6.783366	0
PHD finger protein 10 isoform b [Homo sapiens]	gi|194328736	13.98	RDLSHK	6.918173	0
unnamed protein product, partial [Homo sapiens]	gi|40039260	14.42	VVYGSR	6.95277	0
cyclin-dependent kinase inhibitor 1 [Canis lupus familiaris]	gi|545518904	8.1700001	GGRDDLGGGK	7.006785	0
zinc finger protein 598 [Homo sapiens]	gi|409264581	10.51	CSTKMR	7.007197	0
dexamethasone-induced Ras-related protein 1 isoform X1 [Canis lupus familiaris]	gi|73956260	12.37	EKASGGGQAK	7.030478	0
AMP deaminase 2 isoform X2 [Homo sapiens]	gi|578798942	3.98	FIKRAMK	7.158061	0
coagulation factor VIII isoform X1 [Canis lupus familiaris]	gi|545557151	18.129999	EGSLAKER	7.370575	0
zinc finger protein 568 isoform 2 [Homo sapiens]	gi|325651956	6.7600002	ASLSIHK	7.419601	0
myotubularin-related protein 10 isoform X3 [Canis lupus familiaris]	gi|545493194	14.04	SISGTPLSKFLSGAK	7.520732	0
sorting nexin-25 isoform X4 [Homo sapiens]	gi|578809437	18.02	RVVISHNMDK	7.611645	0
zinc finger protein 836 [Homo sapiens]	gi|156627573	13.16	MHTGDKPYK	7.766491	0
protein PRRC2B [Canis lupus familiaris]	gi|345805923	12.61	KELAKRSFSSQRPLADR	7.777759	0
39S ribosomal protein L11, mitochondrial isoform X1 [Homo sapiens]	gi|578821681	5.3200002	AGLAMPGPPLGPVLGQR	7.77847	0
HLA class I histocompatibility antigen, alpha chain F isoform X4 [Homo sapiens]	gi|578842317	3.8599999	TLAMSGK	7.859496	0
AF119917_31 PRO2249 [Homo sapiens]	gi|7770175	11.59	MDLPTCGAR	7.994611	0
coiled-coil domain-containing protein 136 isoform X13 [Homo sapiens]	gi|578814522	13.98	GGSVGSLSVNK	8.085492	0
notch related protein, partial [Homo sapiens]	gi|1749368	4.79	GDSPGPR	8.093087	0
RNA-binding motif protein, X chromosome isoform 1 [Homo sapiens]	gi|56699409	6.25	SRGPPRGLR	8.13002	0
unnamed protein product [Homo sapiens]	gi|194389262	4.2399998	GNNICEGGEEMDNK	8.16553	0
hCG1654062 [Homo sapiens]	gi|119627151	19.02	TEANGT	8.209194	0
unnamed protein product [Homo sapiens]	gi|194381768	12.38	KEDGTPAATGGSQPPSMGR	8.303342	0
zinc finger protein 609 [Homo sapiens]	gi|71725360	9.2700005	MSLSSGASGGK	8.445717	0
hCG1820498 [Homo sapiens]	gi|119577805	8.3299999	TTWKIFLNSCLSKIK	8.581983	0
bcl-2-like protein 10 isoform X1 [Homo sapiens]	gi|530405270	15.11	EQEGDVAR	8.605927	0
unnamed protein product, partial [Homo sapiens]	gi|40042882	7.9899998	YDIFDPR	8.709718	0
leiomodin-2 [Canis lupus familiaris]	gi|359321176	7.6599998	KQPNNILKEIKNSLR	8.735965	0
carcinoembryonic antigen-related cell adhesion molecule 18 precursor [Homo sapiens]	gi|508083061	15.26	MDLSRPR	8.804919	0
SH2B adapter protein 3 isoform X1 [Canis lupus familiaris]	gi|545543838	8.8999996	AGLARK	8.842726	0
iroquois-class homeodomain protein IRX-5 isoform X2 [Canis lupus familiaris]	gi|545491132	14.69	KGHPTLLE	8.861404	0
mitogen-activated protein kinase kinase kinase 6 [Canis lupus familiaris]	gi|545492222	20.879999	SRSPGSPR	9.084064	0
cohesin subunit SA-3 isoform X1 [Canis lupus familiaris]	gi|545500886	12.11	QASEGHPPVGRITGR	9.473022	0
60S ribosomal protein L13 isoform 2 [Homo sapiens]	gi|341604768	20.120001	TIGISVDPR	9.561173	0
C2 domain-containing protein 3 [Canis lupus familiaris]	gi|545537153	11.82	KGQGSAGVR	9.567533	0

SEC23-interacting protein isoform X2 [Canis lupus familiaris]	gi|545548137	18.93	QKAVKLEQKK	9.604817	0
F-box/LRR-repeat protein 19 isoform 1 [Homo sapiens]	gi|157168349	14.79	ADNGEEGASLGSGWK	9.837845	0
baculoviral IAP repeat-containing protein 2 isoform 2 [Homo sapiens]	gi|390608639	8.5600004	NTSPMR	9.931263	0
inositol 1,4,5-trisphosphate receptor type 3 isoform X3 [Homo sapiens]	gi|578811675	2.72	KGSGK	9.972209	0
WAS/WASL-interacting protein family member 3-like [Homo sapiens]	gi|530380345	5	ASFGR	10.1116	0
unnamed protein product, partial [Homo sapiens]	gi|40979160	14.45	HSRXWDFR	10.12257	0
disintegrin and metalloproteinase domain-containing protein 8 [Canis lupus familiaris]	gi|545548488	8.96	VSAAFRPR	10.33356	0
ERI1 exoribonuclease 3 isoform X2 [Homo sapiens]	gi|530363292	9.0900002	TLPTS	10.39998	0
sirtuin (silent mating type information regulation 2 homolog) 3 (S. cerevisiae), isoform CRA_a, partial [Homo sapiens]	gi|119581643	7.8099999	EAGAGR	11.30224	0
exonuclease 3'-5' domain-containing protein 1 isoform X5 [Canis lupus familiaris]	gi|545549483	12.99	YLSFLEERQK	11.79414	0
F-BAR and double SH3 domains protein 1 isoform X2 [Canis lupus familiaris]	gi|545490432	11	LAGPFLKK	12.34407	0
