# Supplementary material for: Comparative proteomic study of dog and human saliva
Source: PLoS One. 2018 Dec 4;13(12):e0208317. doi: 10.1371/journal.pone.0208317 (PMC6279226; doi:10.1371/journal.pone.0208317)
Supplement: S3 Table — (PDF) [file pone.0208317.s003.pdf]

Supplementary data 3 : Proteins found only in human

Protein name	Accession number	ID Score	Peptide	DOG	HUMAN
2112198A Na channel:SUBUNIT=beta	gi|1096608	12.24	KEKLESMK	0	8.369205
5-methylcytosine rRNA methyltransferase NSUN4 isoform a [Homo sapiens]	gi|40316918	16.139999	VDLATVPR	0	10.60365
A Chain A, Crystal Structure Of Dual Specificity Protein Phosphatase 23 From Homo Sapiens In Complex With Ligand Malate Ion	gi|118138468	4.21	TGTXLACYLVK	0	7.140872
A Chain A, Crystal Structure Of Truncated (delta 1-89) Human Methionine Aminopeptidase Type 1 In Complex With 2-((5-chloro-6-methyl-2- (pyridin-2-yl)pyrimidin-4-yl)amino)-3-phenylpropanamide	gi|564731025	9.8599997	GSHMLEDPYR	0	4.47196
acidic leucine-rich nuclear phosphoprotein 32 family member B [Homo sapiens]	gi|5454088	7.8499999	MDMKRR	0	7.080008
actin-related protein 8 isoform X1 [Homo sapiens]	gi|578806774	10.37	MYSSILVVGGGLMFHK	0	7.903813
AF163258_1 MSTP067 [Homo sapiens]	gi|33337920	22.85	SLKISGKEKNLK	0	6.739758
AF164613_1 Gag-Pro-Pol protein [Homo sapiens]	gi|5802819	12.34	GPELMGPSESKPR	0	10.50061
alternative protein DENND1B [Homo sapiens]	gi|444738091	10.41	KLLLISMRVMTLLK	0	6.577109
anti-tetanus toxoid immunoglobulin light chain variable region, partial [Homo sapiens]	gi|353255046	14.4	APILLIYR	0	6.866848
ATP-dependent DNA helicase Q1 isoform X6 [Canis lupus familiaris]	gi|545546127	12.22	LGIQAGAYHANMEPEDK	0	5.296707
B Chain B, Crystal Structure Analysis Of The Fgf10-fgfr2b Complex	gi|28949025	6.5500002	XEKRLHAVPAANTVKFR	0	6.88173
B Chain B, Crystal Structure Of Monomeric Variant Of Human Alpha-defensin 5, Hd5 (glu21eme Mutant)	gi|387766361	9.04	ESLSGVCXISGRLYR	0	6.802151
B Chain B, X-Ray Structure Of Homo Sapiens Protein Flj36880	gi|56553712	1.5700001	IPDPHK	0	4.751988
calcyphosin-like protein isoform X2 [Homo sapiens]	gi|578809847	6.0700002	EMAIQAK	0	7.154574
carrier family 6, member 8 variant, partial [Homo sapiens]	gi|62088990	15.63	RMWHQALLRSGDKVR	0	6.927011
CD2-associated protein isoform X1 [Homo sapiens]	gi|530381720	7.1100001	AVLSS	0	9.195009
centrosomal protein of 55 kDa [Canis lupus familiaris]	gi|359323204	14.99	TPTAALNESLVECPK	0	9.44721
cornulin [Canis lupus familiaris]	gi|345782802	7.8000002	TQTDVTHTVTQTEVTQVMEQDR	0	8.452077
delta and Notch-like epidermal growth factor-related receptor isoform X1 [Homo sapiens]	gi|530371157	19.27	HARFGKK	0	4.845905
DNA repair protein RAD50 [Homo sapiens]	gi|19924129	8.4099998	ILELDQELIKAERELSKAEK	0	7.983154
dual-specificity tyrosine-(Y)-phosphorylation regulated kinase 4, isoform CRA_b [Homo sapiens]	gi|119609248	15.65	QKFTSAKGPTLSEIYMVGSK	0	5.64156
dynein heavy chain 17, axonemal [Canis lupus familiaris]	gi|545509233	1.4400001	ALMEEAVK	0	4.646714
dynein heavy chain 6, axonemal isoform X1 [Homo sapiens]	gi|578802753	12.79	AAQVIR	0	7.879261
dystrobrevin beta isoform X7 [Homo sapiens]	gi|578802763	8.54	HGPALYT	0	7.490173
E3 SUMO-protein ligase RNF212 isoform b [Homo sapiens]	gi|37059810	5.0100002	RLSSLAAPPSVQFWK	0	7.533463
E3 ubiquitin-protein ligase MIB1 [Canis lupus familiaris]	gi|359320023	19.700001	KEGVGAR	0	5.992401
E3 UFM1-protein ligase 1 [Homo sapiens]	gi|24308039	13.6	KSSVTEE	0	7.172923
FGGY carbohydrate kinase domain-containing protein isoform X7 [Canis lupus familiaris]	gi|545498666	11.15	IGLEDFVADNYRK	0	7.406353
H Chain H, Crystal Structure Of The Dbl And Pleckstrin Homology Domains Of Dbs In Complex With Rhoa	gi|21466031	6.8400002	KKSGS	0	8.512319
hCG1644145, partial [Homo sapiens]	gi|119611283	4.6799998	AANVLK	0	5.529482
hCG2019454, partial [Homo sapiens]	gi|119596033	4.8200002	MRSTFAR	0	8.92604
hCG2036564, isoform CRA_b [Homo sapiens]	gi|119600045	7.8000002	EMCIKTDQVTSQFLK	0	7.2018

HEAT repeat-containing protein 5B isoform X2 [Canis lupus familiaris]	gi|545527576	10.03	KLMTPIECAMTMMSHIPSVIK	0	9.030068
helicase SKI2W isoform X1 [Homo sapiens]	gi|578840867	17.030001	LTYTMILNLLR	0	7.651626
hypermethylated in cancer 2 protein isoform X1 [Canis lupus familiaris]	gi|545544907	13.95	KEPMVGSPFER	0	3.694118
immunoglobulin heavy chain, partial [Homo sapiens]	gi|219566253	9.8400002	SPSLESRLTINK	0	5.384861
integrin alpha-10 isoform X11 [Homo sapiens]	gi|578801641	15.89	AAFDGSGQRLSPR	0	5.83835
intraflagellar transport protein 140 homolog isoform X10 [Homo sapiens]	gi|578828383	18.15	KEPEIMK	0	7.050363
kinesin-like protein KIF2C isoform X3 [Homo sapiens]	gi|530361649	18.4	KAQNSEMR	0	7.676169
lactoferrin [Homo sapiens]	gi|27438055	18.200001	FQLFGSPSGQK	0	5.899138
LIM domain-binding protein 2 isoform X9 [Homo sapiens]	gi|578808437	19.42	SPVSGCALR	0	8.29304
lysine-specific histone demethylase 1A isoform X4 [Homo sapiens]	gi|578798683	8.4300003	QATPGVPAQQSPSM	0	5.94635
mannose-P-dolichol utilization defect 1 protein isoform X3 [Homo sapiens]	gi|578830104	2.73	KPEIP	0	8.364367
methyltransferase-like protein 8 isoform X2 [Canis lupus familiaris]	gi|545555183	14.23	LHSSYR	0	7.399732
MRDS1 protein [Homo sapiens]	gi|22770616	13.1	EGSIPK	0	3.536848
NEDD8-activating enzyme E1 regulatory subunit isoform X1 [Homo sapiens]	gi|530424459	18.24	ALKEFVAK	0	10.32912
odorant-binding protein 2b isoform X2 [Homo sapiens]	gi|578817408	17.26	KLMYLQELPR	0	5.510025
olfactory receptor 10T2 [Homo sapiens]	gi|52218846	9.4300003	VLGMPVATK	0	8.263318
PIGP_HUMAN RecName: Full=Phosphatidylinositol N-acetylglucosaminyltransferase subunit P	gi|425906062	6.75	STSLALIVFLFHRLSK	0	4.887387
plakophilin-3, partial [Canis lupus familiaris]	gi|545531425	13	GLDSYGGHR	0	10.14926
protein BEX1-like [Canis lupus familiaris]	gi|345806410	13.64	WDVFQRLEEPQGR	0	7.707167
protein shisa-9 isoform X2 [Canis lupus familiaris]	gi|545502150	9.7799997	QAYGNK	0	11.37733
protein strawberry notch homolog 2 isoform X3 [Canis lupus familiaris]	gi|545535456	12.74	RVLQELQLMDADVK	0	6.180917
rabenosyn-5 isoform X1 [Homo sapiens]	gi|530372982	8.6700001	KAKDRLLK	0	10.06725
ras-associating and dilute domain-containing protein [Homo sapiens]	gi|148612825	8.25	MFYGTHFIMSPPTKSK	0	7.692249
retinoblastoma-like protein 2 isoform X2 [Homo sapiens]	gi|530424182	17.07	LQDVANDR	0	4.789761
rho GTPase-activating protein 27-like [Canis lupus familiaris]	gi|545510099	7.9400001	AAVAPPAGLLGSAGSFK	0	6.468294
rho-related GTP-binding protein RhoN isoform X1 [Homo sapiens]	gi|530412930	7.9499998	MGMGTR	0	6.494822
RNA polymerase II subunit B1 CTD phosphatase RPAP2 isoform X2 [Canis lupus familiaris]	gi|545503257	10.62	KAGQKANSK	0	6.43417
S65716 prostaglandin-D synthase (EC 5.3.99.2) - human (fragments)	gi|2135996	11.21	AALSMXK	0	8.226482
SAFB-like transcription modulator isoform X12 [Homo sapiens]	gi|578827413	12.45	ARPTARR	0	6.306786
SAM domain and HD domain 1 [Homo sapiens]	gi|22209036	22.790001	FVEQLIR	0	6.034351
serine/threonine-protein kinase Nek8 [Homo sapiens]	gi|30039692	5.9400001	IMSGTFAPISDR	0	6.634113
spermatogenesis-associated protein 31D3 [Homo sapiens]	gi|223633944	3.01	RKSVTFKDR	0	7.178272
SYNGR1 [Homo sapiens]	gi|47678711	8.3599997	MEGGAYGAGK	0	8.055223
TDRD5 protein, partial [Homo sapiens]	gi|59807731	8.8900003	SLLMLK	0	2.93225
testis spermatogenesis apoptosis-related protein 7 [Homo sapiens]	gi|46241188	15.51	LYMKSLLKIFAWATLR	0	9.028374
trafficking kinesin-binding protein 2 isoform X4 [Canis lupus familiaris]	gi|545555869	12.78	TPDAQENGR	0	7.468112
transketolase isoform X1 [Homo sapiens]	gi|578806532	7.8800001	ESWHGKPLPK	0	3.902407

transmembrane protein 25 isoform 3 precursor [Homo sapiens]	gi|221139834	6.3499999	HTLLLLPALLSSVKPEIAQVGAK	0	7.400016
transmembrane protein 53 [Canis lupus familiaris]	gi|545524043	22.129999	KEVGAR	0	7.77826
tubulin-folding cofactor B [Canis lupus familiaris]	gi|545488789	8.6899996	IGRVGSRGR	0	7.614667
ubiquitin carboxyl-terminal hydrolase 24 isoform X3 [Homo sapiens]	gi|578798779	9.5500002	CSTANSR	0	4.169628
ubiquitin-like modifier-activating enzyme 5 isoform X1 [Homo sapiens]	gi|530375300	13.46	VELANMNR	0	5.707277
uncharacterized protein C7orf26 homolog isoform X3 [Canis lupus familiaris]	gi|545501083	12.86	QILSASPR	0	6.840656
uncharacterized protein LOC102155181 isoform X1 [Canis lupus familiaris]	gi|545546427	8.71	EGEERCGKNYSSHRGAAHGPLSR	0	7.681248
unknown, partial [Homo sapiens]	gi|2345091	16.059999	QAEGRSSGTAGDAGSLPR	0	7.526418
unnamed protein product [Homo sapiens]	gi|194374141	7.7199998	DQKLRRMMGVPEIR	0	6.612126
unnamed protein product [Homo sapiens]	gi|34527000	8.9099998	VRVLAPVFRICSPLPR	0	7.836419
unnamed protein product [Homo sapiens]	gi|13397236	10.86	VSVAIAFVGGSR	0	8.802056
unnamed protein product [Homo sapiens]	gi|40046848	7.5	DCAXIVTQKK	0	9.07577
unnamed protein product, partial [Homo sapiens]	gi|40045132	1.24	XGEVNA	0	5.866741
unnamed protein product, partial [Homo sapiens]	gi|40044566	8.7700005	DLGAEKAPXPSR	0	6.883411
unnamed protein product, partial [Homo sapiens]	gi|40040634	12.24	QXLAEHK	0	6.94314
unnamed protein product, partial [Homo sapiens]	gi|40981350	8.4799995	XGMVFSSLR	0	7.633719
unnamed protein product, partial [Homo sapiens]	gi|40042832	17.889999	MASVXLSEAEK	0	9.606595
unnamed protein product, partial [Homo sapiens]	gi|40978340	6.3299999	VQAAXK	0	4.548312
unnamed protein product, partial [Homo sapiens]	gi|40045840	8.6700001	NMTXAEK	0	5.972366
unnamed protein product, partial [Homo sapiens]	gi|40982020	9.9700003	XDGKEPSDKPQKAVQDHK	0	6.248456
WIZ protein, partial [Homo sapiens]	gi|38566138	4.96	TPLNLSSRAEPVRDIR	0	6.97999
zinc finger CCHC domain-containing protein 4 isoform X4 [Homo sapiens]	gi|530376298	1.46	LTASGDKK	0	7.272091
zinc finger protein 181 isoform X4 [Homo sapiens]	gi|578834388	5.9000001	VNGGK	0	5.670215
zinc finger protein 831 isoform X1 [Homo sapiens]	gi|578835748	7.71	EAMAGKGRAGGR	0	6.189744
